# Supplementary material for: Anthropometric indices and cut-off points in the diagnosis of metabolic disorders
Source: PLoS One. 2020 Jun 22;15(6):e0235121. doi: 10.1371/journal.pone.0235121 (PMC7307766; doi:10.1371/journal.pone.0235121)
Supplement: S1 File — (PDF) [file pone.0235121.s001.pdf]

	B_MASS	B_HEIGHT	BMI	WC	%BF	CUN_BAE	BRI	ABSI
1	50,00	168,00	17,715	75,00	8,80	14,634	3,65903	0,085148
2	43,50	160,00	16,992	64,00	10,10	15,503	3,36356	0,076552
3	54,10	172,00	18,287	71,00	10,60	14,283	3,17576	0,077995
4	52,90	174,00	17,473	73,00	10,70	14,251	3,19679	0,082188
5	52,00	170,50	17,888	74,00	11,10	15,329	3,44910	0,082858
6	55,20	174,00	18,232	79,00	13,10	16,692	3,56888	0,086455
7	55,00	173,00	18,377	76,00	23,10	16,906	3,43743	0,082974
8	63,00	172,00	21,295	70,00	5,30	18,697	3,11234	0,069472
9	59,80	170,00	20,692	77,00	6,90	20,438	3,67228	0,078355
10	55,00	168,00	19,487	68,00	11,50	17,181	3,19331	0,072448
11	55,00	168,00	19,487	76,00	12,00	16,023	3,72561	0,080971
12	60,50	176,50	19,421	79,00	12,10	17,467	3,43103	0,082302
13	65,10	181,00	19,871	76,00	12,30	18,710	3,02546	0,077000
14	54,80	163,00	20,626	71,00	12,40	18,187	3,68717	0,073943
15	74,00	193,00	19,866	79,00	12,40	16,440	2,65244	0,077524
16	55,30	168,00	19,593	75,00	13,00	18,494	3,65903	0,079616
17	62,00	177,50	19,679	79,00	13,10	15,948	3,37753	0,081351
18	60,00	176,00	19,370	76,00	13,20	17,973	3,27629	0,079428
19	58,20	172,00	19,673	79,00	13,30	17,083	3,68355	0,082658
20	57,00	174,00	18,827	79,00	13,40	16,765	3,56888	0,084626
21	59,80	176,00	19,305	75,00	13,50	17,682	3,21570	0,078558
22	57,10	174,00	18,860	74,00	13,60	17,617	3,25878	0,079177
23	57,00	171,00	19,493	79,00	13,80	18,348	3,74242	0,083408
24	60,80	170,00	21,038	78,00	13,90	20,923	3,73730	0,078499
25	64,70	176,00	20,887	79,00	13,90	18,767	3,45813	0,078515
26	66,20	178,00	20,894	78,00	14,00	20,368	3,29187	0,077068
27	49,70	163,00	18,706	73,00	14,00	17,998	3,82865	0,081141
28	69,80	182,50	20,957	75,00	14,20	18,345	2,89793	0,073037
29	58,70	171,00	20,075	76,00	14,60	18,262	3,54965	0,078684
30	56,80	170,50	19,539	78,00	14,80	18,799	3,70760	0,082344
31	66,60	170,00	23,045	73,00	14,90	22,291	3,41229	0,069138
32	58,90	172,00	19,909	74,00	15,10	18,390	3,36610	0,076812
33	66,00	171,00	22,571	73,00	15,20	20,973	3,35698	0,069897
34	58,60	169,50	20,397	73,00	15,20	20,022	3,44031	0,075110
35	61,10	170,00	21,142	78,00	15,30	19,502	3,73730	0,078242
36	57,90	164,00	21,527	70,00	15,40	20,246	3,55627	0,070634
37	54,20	163,50	20,275	79,00	15,60	19,666	4,21926	0,083092
38	68,50	176,00	22,114	76,00	15,70	20,287	3,27629	0,072713
39	68,40	180,00	21,111	77,00	15,70	19,630	3,13185	0,075136
40	62,50	172,00	21,126	78,00	15,70	19,305	3,62004	0,077824
41	73,00	176,50	23,433	79,00	15,80	21,949	3,43103	0,072616
42	62,00	175,00	20,245	79,00	15,80	18,702	3,51302	0,080395
43	63,40	172,00	21,431	72,00	15,90	18,907	3,23920	0,071156
44	67,10	181,00	20,482	79,00	15,90	19,053	3,19726	0,078441

45	54,90	164,00	20,412	78,00	15,90	18,768	4,11545	0,081549
46	67,00	176,00	21,630	75,00	16,00	20,396	3,21570	0,072824
47	69,80	171,00	23,871	75,00	16,20	23,322	3,48541	0,069181
48	60,00	173,00	20,047	77,00	16,30	18,408	3,50018	0,079328
49	56,70	164,00	21,081	76,00	16,50	20,809	3,97557	0,077767
50	58,00	168,50	20,428	74,00	16,80	18,974	3,56328	0,076286
51	74,20	176,50	23,819	70,00	16,90	24,997	2,88892	0,063648
52	56,80	164,00	21,118	73,00	17,10	21,558	3,76586	0,074609
53	66,10	170,00	22,872	75,00	17,30	20,954	3,54226	0,071390
54	55,90	164,00	20,784	75,00	17,30	20,567	3,90566	0,077474
55	69,60	169,50	24,225	79,00	17,40	23,966	3,83270	0,072476
56	65,70	172,00	22,208	79,00	17,50	22,377	3,68355	0,076241
57	64,60	165,00	23,728	78,00	17,70	22,533	4,04952	0,073538
58	67,80	171,00	23,187	76,00	17,80	21,429	3,54965	0,071476
59	67,40	174,50	22,134	79,00	17,80	20,807	3,54083	0,075861
60	61,60	164,00	22,903	70,00	17,90	21,622	3,55627	0,067777
61	58,20	157,00	23,612	76,00	18,20	24,727	4,46034	0,073697
62	61,60	167,00	22,088	78,00	18,20	21,884	3,92123	0,076672
63	69,40	169,00	24,299	78,00	18,30	24,210	3,79751	0,071520
64	73,20	175,00	23,902	77,00	19,40	23,367	3,39038	0,070148
65	80,20	181,00	24,480	79,00	19,50	24,462	3,19726	0,069648
66	65,70	165,00	24,132	79,00	19,60	23,693	4,11862	0,073647
67	42,80	139,00	22,152	73,00	19,60	20,344	5,77152	0,078501
68	57,30	159,00	22,665	77,00	19,80	20,483	4,38968	0,076246
69	69,30	174,00	22,889	78,00	20,00	23,154	3,50684	0,073349
70	65,20	166,00	23,661	79,00	20,40	22,876	4,05305	0,074396
71	49,40	162,00	18,823	74,00	20,60	17,363	3,96428	0,082163
72	68,20	167,00	24,454	79,00	21,30	25,681	3,98866	0,072561
73	65,00	167,00	23,307	78,00	21,30	24,327	3,92123	0,073975
74	72,30	173,00	24,157	70,00	21,40	25,580	3,06117	0,063686
75	67,00	173,00	22,386	76,00	22,00	22,783	3,43743	0,072745
76	56,00	168,00	19,841	74,00	23,30	16,778	3,59246	0,077899
77	75,50	185,00	22,060	78,00	29,30	20,861	2,94894	0,072908
78	69,00	176,00	22,275	79,00	32,00	21,015	3,45813	0,075218
79	70,50	170,00	24,394	67,00	34,40	23,503	3,02266	0,061093
80	69,80	165,80	25,391	72,00	21,10	27,147	3,58737	0,064727
81	77,80	173,00	25,995	74,00	21,20	25,649	3,31197	0,064114
82	71,60	164,00	26,621	68,00	27,10	27,834	3,41662	0,059558
83	55,80	174,00	18,430	82,00	12,70	15,751	3,75507	0,089094
84	54,00	174,00	17,836	80,00	17,70	15,036	3,63093	0,088842
85	60,70	186,00	17,545	81,00	18,60	14,366	3,06575	0,087960
86	78,20	177,00	24,961	84,00	11,30	24,451	3,70396	0,073924
87	69,20	173,00	23,121	85,00	11,50	21,943	4,00257	0,079626
88	65,00	178,00	20,515	85,00	12,50	18,016	3,70682	0,085015
89	64,70	176,00	20,887	86,00	12,90	19,120	3,88276	0,085472
90	58,20	172,00	19,673	80,00	13,20	17,465	3,74708	0,083704
91	62,80	176,00	20,274	85,00	13,20	18,193	3,82207	0,086173
92	68,70	185,00	20,073	82,00	13,40	16,771	3,16819	0,081624
93	60,80	174,00	20,082	82,00	13,40	18,459	3,75507	0,084140
94	72,50	186,00	20,956	84,00	13,60	18,344	3,22846	0,081030

95	62,40	181,00	19,047	80,00	13,60	16,702	3,25455	0,083374
96	68,10	179,00	21,254	83,00	13,70	19,151	3,53338	0,080852
97	66,80	166,00	24,242	80,00	14,10	23,706	4,12132	0,074130
98	59,40	167,00	21,299	82,00	14,30	18,875	4,19103	0,082582
99	59,00	174,00	19,487	80,00	14,40	17,953	3,63093	0,083749
100	61,50	173,00	20,549	80,00	14,50	18,972	3,68850	0,081073
101	77,40	180,00	23,889	81,00	14,70	22,623	3,36356	0,072787
102	66,70	174,00	22,031	81,00	14,70	21,476	3,69300	0,078137
103	59,40	172,00	20,078	83,00	14,70	19,198	3,93772	0,085670
104	62,50	175,00	20,408	84,00	14,80	18,033	3,81981	0,085027
105	73,50	179,00	22,939	85,00	14,90	21,985	3,65065	0,078693
106	62,20	169,00	21,778	80,00	14,90	20,446	3,92917	0,078911
107	69,70	178,00	21,998	84,00	14,90	21,430	3,64751	0,080194
108	61,50	170,00	21,280	81,00	15,00	18,846	3,93245	0,080899
109	62,30	176,50	19,999	82,00	15,00	16,839	3,61191	0,083774
110	67,00	180,00	20,679	85,00	15,00	20,241	3,59542	0,084094
111	59,70	174,00	19,719	84,00	15,00	16,013	3,87925	0,087248
112	64,50	170,00	22,318	85,00	15,20	20,272	4,19281	0,082241
113	71,90	181,00	21,947	87,00	15,20	19,539	3,65581	0,082496
114	62,00	174,00	20,478	83,00	15,20	19,411	3,81715	0,084064
115	58,00	161,50	22,237	81,00	15,30	20,635	4,50228	0,080602
116	78,60	189,00	22,004	83,00	15,40	21,437	3,03246	0,076886
117	77,00	187,00	22,020	83,00	15,40	20,966	3,12620	0,077260
118	64,80	172,00	21,904	84,00	15,40	19,803	4,00129	0,081816
119	67,80	176,00	21,888	87,00	15,40	20,774	3,94347	0,083810
120	73,40	184,00	21,680	84,00	15,50	19,798	3,32807	0,079646
121	69,90	183,00	20,873	87,00	15,50	19,807	3,54735	0,084835
122	82,20	183,00	24,545	85,00	15,60	23,443	3,43519	0,074396
123	68,60	170,00	23,737	80,00	15,70	22,399	3,86739	0,074287
124	71,90	176,00	23,212	82,00	15,70	21,619	3,64005	0,075961
125	70,00	174,00	23,121	84,00	15,70	21,483	3,87925	0,078464
126	72,70	180,00	22,438	83,00	15,80	22,054	3,47947	0,077765
127	65,70	174,00	21,700	80,00	15,80	19,660	3,63093	0,077954
128	71,30	179,00	22,253	81,00	15,90	21,467	3,41615	0,076525
129	61,90	167,00	22,195	80,00	15,90	21,059	4,05610	0,078384
130	63,00	168,50	22,189	83,00	15,90	21,213	4,15924	0,080975
131	63,40	174,00	20,941	82,00	15,90	20,258	3,75507	0,081824
132	67,40	177,00	21,514	85,00	15,90	20,395	3,76395	0,082596
133	58,50	165,00	21,488	86,00	15,90	21,208	4,60269	0,086623
134	65,00	171,00	22,229	80,00	16,00	20,947	3,80670	0,077383
135	67,90	176,50	21,796	83,00	16,00	19,472	3,67223	0,080067
136	61,50	170,00	21,280	85,00	16,00	20,916	4,19281	0,084894
137	76,10	188,00	21,531	80,00	16,10	19,742	2,91975	0,075387
138	70,10	178,00	22,125	80,00	16,20	20,793	3,41038	0,076084
139	67,10	178,00	21,178	84,00	16,20	20,250	3,64751	0,082252
140	67,00	172,00	22,647	80,00	16,30	21,560	3,74708	0,076204
141	76,00	183,50	22,571	85,00	16,30	20,972	3,40925	0,078567
142	60,40	167,00	21,657	80,00	16,30	21,447	4,05610	0,079676
143	66,00	176,00	21,307	80,00	16,40	20,265	3,51876	0,078461
144	66,10	172,00	22,343	83,00	16,40	21,758	3,93772	0,079778

145	69,50	175,00	22,694	85,00	16,50	22,571	3,88120	0,080161
146	64,40	170,00	22,284	83,00	16,50	22,966	4,06261	0,080389
147	73,00	175,00	23,837	85,00	16,60	22,983	3,88120	0,077577
148	59,30	171,00	20,280	80,00	16,70	20,224	3,80670	0,082265
149	65,00	168,00	23,030	80,00	16,80	22,885	3,99206	0,076250
150	70,00	181,00	21,367	81,00	16,80	19,666	3,31184	0,078190
151	73,90	176,00	23,857	82,00	16,90	23,012	3,64005	0,074584
152	71,60	176,00	23,115	84,00	16,90	23,615	3,76139	0,078031
153	55,60	160,00	21,719	82,00	16,90	20,862	4,68609	0,083278
154	63,50	173,00	21,217	84,00	16,90	21,692	3,93974	0,083330
155	56,40	163,00	21,228	84,00	16,90	21,015	4,60781	0,085819
156	74,00	179,00	23,095	83,00	17,00	21,445	3,53338	0,076495
157	69,60	175,50	22,597	82,00	17,00	22,120	3,66843	0,077441
158	66,70	175,50	21,656	83,00	17,00	20,939	3,72945	0,080641
159	72,00	185,50	20,924	84,00	17,00	19,529	3,25306	0,081223
160	58,10	168,00	20,585	81,00	17,00	20,108	4,05871	0,083200
161	59,00	175,00	19,265	80,00	17,00	17,623	3,57436	0,084150
162	70,20	182,00	21,193	87,00	17,00	21,140	3,60113	0,084208
163	65,00	176,00	20,984	86,00	17,00	20,320	3,88276	0,085209
164	69,30	176,00	22,372	82,00	17,10	20,514	3,64005	0,077849
165	74,40	180,00	22,963	86,00	17,10	21,555	3,65341	0,079343
166	70,30	174,00	23,220	86,00	17,10	23,908	4,00347	0,080104
167	69,90	174,00	23,088	86,00	17,10	21,587	4,00347	0,080409
168	62,00	166,00	22,500	83,00	17,10	21,822	4,32620	0,080830
169	79,00	181,00	24,114	84,00	17,20	22,953	3,48379	0,074804
170	77,20	182,00	23,306	86,00	17,20	21,760	3,54442	0,078129
171	70,30	171,00	24,042	87,00	17,20	23,134	4,25698	0,079869
172	63,20	169,00	22,128	85,00	17,20	20,798	4,25851	0,082956
173	73,10	175,50	23,734	84,00	17,30	22,541	3,79047	0,076777
174	68,00	171,00	23,255	82,00	17,30	23,804	3,93530	0,076967
175	70,40	174,00	23,253	85,00	17,30	23,498	3,94136	0,079097
176	72,00	175,00	23,510	87,00	17,30	23,999	4,00400	0,080136
177	58,20	164,50	21,508	81,00	17,30	19,027	4,29095	0,081660
178	61,30	168,00	21,719	83,00	17,30	22,372	4,19203	0,082261
179	74,80	179,50	23,215	81,00	17,40	21,776	3,38975	0,074291
180	76,20	185,00	22,264	83,00	17,40	22,293	3,22302	0,077105
181	62,00	163,00	23,335	82,00	17,40	22,256	4,46602	0,078652
182	64,40	169,00	22,548	85,00	17,40	21,733	4,25851	0,081922
183	62,00	172,00	20,957	84,00	17,40	21,163	4,00129	0,084261
184	56,00	163,00	21,077	83,00	17,40	20,629	4,53691	0,085201
185	71,80	175,50	23,311	81,00	17,50	23,729	3,60743	0,074926
186	65,30	173,00	21,818	82,00	17,50	21,839	3,81409	0,079844
187	74,60	178,00	23,545	83,00	17,60	23,600	3,58821	0,075730
188	72,80	178,00	22,977	82,00	17,60	22,039	3,52892	0,076046
189	73,40	183,00	21,918	84,00	17,60	21,314	3,37912	0,079285
190	73,00	180,00	22,531	85,00	17,60	20,595	3,59542	0,079420
191	66,60	170,00	23,045	84,00	17,60	23,981	4,12770	0,079556
192	73,60	183,50	21,858	86,00	17,60	21,561	3,46502	0,081210
193	72,00	180,00	22,222	87,00	17,60	21,261	3,71141	0,082040
194	69,90	172,00	23,628	80,00	17,70	23,419	3,74708	0,074082

195	69,70	173,50	23,154	82,00	17,70	23,516	3,78445	0,076632
196	73,90	176,00	23,857	85,00	17,70	24,321	3,82207	0,077313
197	65,00	165,00	23,875	83,00	17,70	24,635	4,39515	0,077930
198	71,00	173,00	23,723	85,00	17,70	22,378	4,00257	0,078274
199	66,40	171,50	22,576	82,00	17,70	20,980	3,90459	0,078389
200	68,10	178,20	21,445	84,00	17,70	21,661	3,63634	0,081521
201	59,20	163,00	22,282	84,00	17,70	21,670	4,60781	0,083091
202	62,00	172,00	20,957	86,00	17,70	20,810	4,12846	0,086267
203	76,30	176,00	24,632	83,00	17,80	24,120	3,70071	0,073902
204	68,10	171,00	23,289	82,00	17,80	23,397	3,93530	0,076892
205	67,00	174,00	22,130	80,00	17,80	20,147	3,63093	0,076942
206	65,20	169,00	22,828	82,00	17,80	21,512	4,06087	0,078383
207	70,00	177,00	22,344	86,00	17,80	21,920	3,82395	0,081485
208	67,70	177,00	21,609	80,00	17,90	21,042	3,46410	0,077507
209	64,40	172,00	21,769	80,00	17,90	20,433	3,74708	0,078242
210	75,90	183,00	22,664	87,00	17,90	20,796	3,54735	0,080303
211	78,60	178,00	24,807	82,00	18,00	24,505	3,52892	0,072257
212	70,20	171,00	24,007	82,00	18,00	24,524	3,93530	0,075350
213	69,70	171,00	23,836	84,00	18,00	23,565	4,06394	0,077557
214	71,00	177,00	22,663	83,00	18,00	23,316	3,64398	0,077902
215	81,00	188,00	22,918	87,00	18,00	22,418	3,29132	0,078643
216	60,30	167,00	21,621	82,00	18,00	20,721	4,19103	0,081758
217	76,10	181,00	23,229	85,00	18,10	21,645	3,54112	0,077606
218	65,30	176,00	21,081	80,00	18,10	21,507	3,51876	0,079021
219	69,50	170,00	24,048	86,00	18,10	23,288	4,25793	0,079168
220	72,70	174,00	24,012	87,00	18,10	23,092	4,06560	0,079242
221	63,80	173,00	21,317	80,00	18,20	20,968	3,68850	0,079113
222	69,30	173,50	23,022	86,00	18,20	21,642	4,03431	0,080679
223	81,10	182,00	24,484	82,00	18,30	25,441	3,31765	0,072087
224	66,40	166,00	24,096	86,00	18,30	25,502	4,53121	0,080010
225	66,70	169,00	23,354	86,00	18,30	23,034	4,32442	0,080969
226	74,70	176,00	24,115	82,00	18,40	22,813	3,64005	0,074050
227	68,00	167,00	24,382	82,00	18,40	25,588	4,19103	0,075464
228	73,10	173,00	24,424	85,00	18,40	24,245	4,00257	0,076768
229	83,20	190,00	23,047	86,00	18,40	21,373	3,14260	0,077039
230	64,80	172,00	21,904	80,00	18,40	20,963	3,74708	0,077920
231	66,90	168,00	23,703	85,00	18,40	22,349	4,32540	0,079474
232	75,40	183,00	22,515	86,00	18,40	21,366	3,49127	0,079731
233	59,40	161,00	22,916	86,00	18,40	21,950	4,90219	0,084010
234	63,60	166,00	23,080	80,00	18,50	23,875	4,12132	0,076596
235	70,00	172,00	23,661	85,00	18,50	22,877	4,06487	0,078637
236	64,00	173,00	21,384	80,00	18,50	20,891	3,68850	0,078948
237	74,80	173,00	24,992	83,00	18,60	25,704	3,87691	0,073821
238	67,60	167,00	24,239	82,00	18,60	24,835	4,19103	0,075761
239	72,30	176,00	23,341	84,00	18,60	23,167	3,76139	0,077526
240	65,00	162,00	24,768	84,00	18,60	24,040	4,68158	0,077672
241	75,30	179,00	23,501	87,00	18,60	22,944	3,76795	0,079256
242	62,40	164,00	23,200	84,00	18,60	22,362	4,53540	0,080635
243	64,40	167,00	23,092	85,00	18,60	21,899	4,39351	0,081113
244	56,00	162,00	21,338	87,00	18,70	19,107	4,89705	0,088850

245	67,00	167,00	24,024	84,00	18,80	24,690	4,32600	0,078072
246	68,20	173,00	22,787	83,00	18,80	22,545	3,87691	0,078510
247	76,90	185,00	22,469	86,00	18,80	22,895	3,38756	0,079407
248	70,00	176,00	22,598	84,00	18,90	21,330	3,76139	0,079215
249	72,00	178,00	22,724	80,00	19,00	22,928	3,41038	0,074740
250	67,30	170,00	23,287	82,00	19,00	24,452	3,99752	0,077122
251	69,70	171,00	23,836	84,00	19,00	24,147	4,06394	0,077557
252	75,80	177,50	24,059	87,00	19,00	25,310	3,85459	0,078356
253	71,40	177,00	22,790	84,00	19,00	21,925	3,70396	0,078546
254	69,30	174,00	22,889	84,00	19,00	22,999	3,87925	0,078992
255	61,00	165,50	22,271	84,00	19,00	21,655	4,42926	0,082488
256	77,00	176,00	24,858	86,00	19,10	24,576	3,88276	0,076108
257	67,00	172,00	22,647	82,00	19,10	21,560	3,87416	0,078110
258	69,40	173,00	23,188	84,00	19,10	21,736	3,93974	0,078538
259	63,10	166,00	22,899	84,00	19,10	22,546	4,39453	0,080851
260	71,40	169,00	24,999	85,00	19,20	25,847	4,25851	0,076476
261	71,40	174,00	23,583	83,00	19,20	23,060	3,81715	0,076513
262	66,00	167,50	23,524	83,00	19,20	23,274	4,22512	0,078114
263	72,90	174,50	23,941	86,00	19,20	23,277	3,97290	0,078375
264	75,30	182,50	22,608	86,00	19,20	22,294	3,51773	0,079620
265	61,40	165,00	22,553	83,00	19,20	20,469	4,39515	0,080947
266	76,40	176,50	24,525	85,00	19,30	24,940	3,79289	0,075796
267	69,20	171,00	23,665	82,00	19,30	23,619	3,93530	0,076075
268	66,30	168,00	23,491	82,00	19,30	23,674	4,12536	0,077131
269	80,10	183,00	23,918	87,00	19,30	24,259	3,54735	0,077471
270	66,50	169,00	23,283	84,00	19,30	22,482	4,19262	0,079245
271	59,70	156,00	24,532	84,00	19,30	24,255	5,15476	0,079659
272	62,20	168,50	21,907	81,00	19,30	22,128	4,02672	0,079700
273	63,50	167,50	22,633	83,00	19,30	23,118	4,22512	0,080151
274	67,90	171,50	23,086	87,00	19,30	23,422	4,22438	0,081939
275	72,00	174,00	23,781	87,00	19,40	23,926	4,06560	0,079755
276	65,00	165,00	23,875	86,00	19,40	23,038	4,60269	0,080747
277	64,00	166,00	23,225	85,00	19,40	24,220	4,46286	0,081044
278	78,30	181,00	23,900	84,00	19,50	23,075	3,48379	0,075249
279	79,20	181,00	24,175	86,00	19,50	24,323	3,59846	0,076456
280	71,20	179,00	22,222	87,00	19,60	22,883	3,76795	0,082270
281	69,30	178,00	21,872	86,00	19,60	20,253	3,76614	0,082419
282	79,30	181,50	24,072	81,00	19,70	23,752	3,28631	0,072116
283	78,00	181,50	23,678	87,00	19,70	22,017	3,62836	0,078317
284	60,80	167,00	21,801	82,00	19,70	22,148	4,19103	0,081310
285	64,10	165,00	23,545	86,00	19,70	23,005	4,60269	0,081501
286	77,20	176,00	24,923	85,00	19,80	24,666	3,82207	0,075093
287	69,20	168,00	24,518	87,00	19,80	24,931	4,45882	0,079532
288	67,10	170,00	23,218	85,00	19,80	23,906	4,19281	0,080102
289	69,30	173,50	23,022	86,00	19,80	23,950	4,03431	0,080679
290	68,10	171,00	23,289	85,00	19,90	23,850	4,12827	0,079705
291	66,50	171,00	22,742	85,00	19,90	21,071	4,12827	0,080978
292	81,20	181,00	24,786	82,00	20,00	23,793	3,36915	0,071698
293	71,50	171,00	24,452	84,00	20,00	24,144	4,06394	0,076250
294	69,70	167,00	24,992	86,00	20,00	25,704	4,46103	0,077853

295	70,90	175,00	23,151	85,00	20,00	21,376	3,88120	0,079102
296	66,00	168,00	23,384	86,00	20,00	22,777	4,39211	0,081138
297	64,80	164,00	24,093	87,00	20,00	24,925	4,74556	0,081440
298	64,10	171,00	21,921	84,00	20,10	21,485	4,06394	0,082011
299	62,50	170,00	21,626	86,00	20,10	22,247	4,25793	0,084974
300	57,60	172,00	19,470	81,00	20,10	16,961	3,81061	0,085338
301	68,00	165,00	24,977	81,00	20,20	26,625	4,25686	0,073799
302	70,00	174,00	23,121	87,00	20,20	23,623	4,06560	0,081267
303	66,80	171,50	22,712	86,00	20,20	22,596	4,16040	0,081884
304	73,00	173,00	24,391	83,00	20,30	24,338	3,87691	0,075030
305	84,00	189,00	23,516	86,00	20,30	22,964	3,19002	0,076213
306	69,80	169,00	24,439	84,00	20,30	25,522	4,19262	0,076727
307	65,90	168,00	23,349	86,00	20,30	23,780	4,39211	0,081221
308	67,60	172,00	22,850	86,00	20,30	23,567	4,12846	0,081434
309	71,20	169,00	24,929	83,00	20,40	24,136	4,12674	0,074816
310	73,40	172,00	24,811	85,00	20,50	26,006	4,06487	0,076190
311	73,70	175,50	23,928	84,00	20,50	24,128	3,79047	0,076360
312	70,60	170,00	24,429	86,00	20,50	25,649	4,25793	0,078343
313	64,30	162,00	24,501	87,00	20,50	25,186	4,89705	0,081029
314	76,40	175,00	24,947	85,00	20,60	24,700	3,88120	0,075258
315	63,70	166,00	23,117	80,00	20,60	22,088	4,12132	0,076516
316	58,50	154,00	24,667	84,00	20,60	25,682	5,32506	0,079881
317	66,90	168,00	23,703	80,00	20,70	23,965	3,99206	0,074799
318	74,50	177,50	23,646	83,00	20,70	23,887	3,61598	0,075620
319	70,00	172,00	23,661	87,00	20,70	22,729	4,19206	0,080487
320	69,40	168,00	24,589	82,00	20,80	25,441	4,12536	0,074817
321	73,80	180,00	22,778	82,00	20,80	20,812	3,42151	0,076062
322	69,70	169,50	24,260	85,00	20,80	23,025	4,22552	0,077906
323	64,50	161,50	24,729	85,00	20,80	23,985	4,79124	0,078799
324	66,00	164,00	24,539	87,00	20,80	24,959	4,74556	0,080450
325	54,80	159,00	21,676	82,00	20,80	20,633	4,76226	0,083648
326	75,60	179,00	23,595	82,00	20,90	22,633	3,47476	0,074504
327	70,80	172,00	23,932	86,00	20,90	23,988	4,12846	0,078962
328	68,00	168,50	23,950	87,00	20,90	24,158	4,42442	0,080664
329	74,00	181,00	22,588	83,00	21,00	22,265	3,42646	0,077207
330	67,00	165,00	24,610	85,00	21,00	25,193	4,53349	0,078212
331	63,70	166,50	22,978	82,00	21,00	23,584	4,22431	0,078626
332	70,00	168,00	24,802	81,00	21,10	26,130	4,05871	0,073481
333	68,10	170,00	23,564	84,00	21,10	23,775	4,12770	0,078383
334	66,00	166,00	23,951	85,00	21,10	22,859	4,46286	0,079399
335	76,80	176,00	24,793	83,00	21,20	23,940	3,70071	0,073581
336	67,00	165,00	24,610	83,00	21,20	24,641	4,39515	0,076372
337	70,90	171,00	24,247	84,00	21,20	24,987	4,06394	0,076679
338	66,90	167,00	23,988	86,00	21,20	23,057	4,46103	0,080010
339	75,70	175,00	24,718	86,00	21,30	26,160	3,94260	0,076612
340	70,00	171,00	23,939	86,00	21,30	23,709	4,19262	0,079177
341	75,70	177,50	24,027	87,00	21,40	23,544	3,85459	0,078425
342	67,00	171,00	22,913	86,00	21,40	23,186	4,19262	0,081523
343	71,90	175,00	23,478	85,00	21,60	23,209	3,88120	0,078367
344	71,00	171,00	24,281	87,00	21,60	24,327	4,25698	0,079343

345	75,10	173,50	24,948	87,00	21,70	25,106	4,09680	0,077359
346	68,70	170,00	23,772	85,00	21,70	24,205	4,19281	0,078854
347	65,70	167,00	23,558	87,00	21,70	24,953	4,52856	0,081923
348	83,30	186,00	24,078	86,00	21,80	23,473	3,33697	0,075625
349	70,90	172,00	23,966	87,00	21,80	22,880	4,19206	0,079805
350	65,00	164,00	24,167	82,00	21,90	24,312	4,39536	0,076602
351	80,00	181,00	24,419	87,00	21,90	23,818	3,65581	0,076829
352	77,70	178,00	24,523	87,00	21,90	23,966	3,82546	0,077254
353	74,10	175,00	24,196	86,00	21,90	25,203	3,94260	0,077711
354	65,20	169,00	22,828	80,00	22,00	23,694	3,92917	0,076471
355	69,00	176,00	22,275	85,00	22,00	20,691	3,82207	0,080931
356	71,00	170,00	24,567	83,00	22,10	25,551	4,06261	0,075326
357	69,00	168,00	24,447	87,00	22,10	25,672	4,45882	0,079685
358	60,10	156,00	24,696	81,00	22,20	24,623	4,92224	0,076473
359	65,00	164,00	24,167	82,00	22,20	24,027	4,39536	0,076602
360	74,00	172,50	24,869	86,00	22,20	25,133	4,09680	0,076854
361	65,50	164,00	24,353	83,00	22,20	24,286	4,46537	0,077141
362	65,20	166,00	23,661	85,00	22,20	24,497	4,46286	0,080047
363	66,40	164,00	24,688	83,00	22,30	24,748	4,46537	0,076443
364	72,00	172,00	24,337	87,00	22,30	25,108	4,19206	0,078990
365	78,50	179,00	24,500	83,00	22,40	24,072	3,53338	0,073543
366	73,00	172,00	24,676	87,00	22,40	26,105	4,19206	0,078267
367	64,00	166,00	23,225	86,00	22,50	23,461	4,53121	0,081998
368	67,70	174,50	22,233	87,00	22,60	21,277	4,03467	0,083296
369	78,60	178,00	24,807	87,00	22,70	25,458	3,82546	0,076663
370	67,60	167,00	24,239	86,00	22,80	24,410	4,46103	0,079457
371	72,70	173,00	24,291	87,00	22,90	24,199	4,12828	0,078862
372	66,40	168,00	23,526	84,00	23,00	23,128	4,25871	0,078933
373	71,00	171,00	24,281	87,00	23,00	24,186	4,25698	0,079343
374	63,80	162,00	24,310	85,00	23,00	24,508	4,75339	0,079579
375	76,70	179,00	23,938	85,00	23,10	23,274	3,65065	0,076489
376	68,30	169,00	23,914	83,00	23,10	24,832	4,12674	0,076920
377	60,00	166,00	21,774	85,00	23,20	22,612	4,46286	0,084607
378	73,50	175,00	24,000	87,00	23,40	23,650	4,00400	0,079042
379	67,71	165,00	24,871	81,00	23,70	25,677	4,25686	0,074009
380	66,60	164,00	24,762	84,00	23,70	26,216	4,53540	0,077209
381	65,80	164,50	24,316	83,00	23,80	25,361	4,43010	0,077102
382	74,70	173,00	24,959	86,00	24,00	25,794	4,06542	0,076558
383	69,00	171,00	23,597	87,00	24,00	23,080	4,25698	0,080869
384	76,00	175,50	24,675	87,00	24,80	24,868	3,97360	0,077483
385	69,20	171,50	23,528	86,00	24,80	24,022	4,16040	0,079980
386	69,00	169,00	24,159	87,00	24,90	25,155	4,39034	0,080080
387	62,20	166,00	22,572	82,00	25,30	22,084	4,25790	0,079685
388	72,50	174,00	23,946	85,00	27,90	24,008	3,94136	0,077562
389	71,70	170,00	24,810	80,00	32,00	24,916	3,86739	0,072130
390	60,10	161,00	23,186	82,00	32,40	24,320	4,61135	0,079479
391	70,00	170,00	24,221	83,00	33,00	24,812	4,06261	0,076042
392	73,00	179,00	22,783	84,00	36,00	21,133	3,59201	0,078122
393	85,10	181,00	25,976	84,00	17,10	25,748	3,48379	0,071186
394	76,10	173,00	25,427	86,00	18,80	25,108	4,06542	0,075616

395	76,00	172,00	25,690	85,00	19,20	25,219	4,06487	0,074442
396	82,10	181,00	25,060	84,00	19,30	24,458	3,48379	0,072909
397	75,00	166,00	27,217	85,00	19,30	27,682	4,46286	0,072912
398	81,40	174,00	26,886	86,00	19,50	26,769	4,00347	0,072645
399	74,60	172,00	25,216	86,00	19,70	26,001	4,12846	0,076257
400	76,10	170,00	26,332	87,00	19,80	27,584	4,32307	0,075388
401	70,00	167,00	25,100	86,00	20,10	26,246	4,46103	0,077630
402	71,00	168,00	25,156	84,00	20,30	24,328	4,25871	0,075486
403	81,30	166,00	29,504	87,00	20,40	30,560	4,59957	0,070721
404	75,70	173,50	25,148	80,00	20,40	26,840	3,65959	0,070758
405	73,20	167,00	26,247	87,00	20,40	26,001	4,52856	0,076227
406	78,00	175,00	25,469	83,00	20,80	25,037	3,75843	0,072479
407	79,20	177,00	25,280	86,00	20,80	25,428	3,82395	0,075046
408	76,00	172,00	25,690	86,00	20,80	25,475	4,12846	0,075318
409	74,60	172,50	25,070	87,00	20,80	25,808	4,16003	0,077331
410	70,30	166,00	25,512	87,00	20,80	24,967	4,59957	0,077918
411	68,50	165,00	25,161	84,00	20,90	26,193	4,46431	0,076159
412	74,00	171,00	25,307	86,00	21,00	25,990	4,19262	0,076297
413	73,00	167,50	26,019	87,00	21,40	26,806	4,49353	0,076557
414	92,30	184,00	27,263	84,00	21,50	27,514	3,32807	0,068364
415	80,20	178,50	25,171	87,00	21,80	25,145	3,79659	0,075817
416	77,20	168,00	27,353	86,00	21,90	29,445	4,39211	0,073088
417	69,80	167,00	25,028	82,00	21,90	26,153	4,19103	0,074161
418	77,00	173,00	25,728	87,00	21,90	27,567	4,12828	0,075898
419	64,10	158,50	25,515	84,00	21,90	27,173	4,95099	0,076984
420	77,10	169,50	26,836	84,00	22,00	28,227	4,16002	0,071981
421	77,40	174,00	25,565	86,00	22,10	26,719	4,00347	0,075127
422	78,60	176,50	25,231	87,00	22,10	26,813	3,91359	0,076125
423	75,40	169,50	26,244	87,00	22,20	26,243	4,35655	0,075668
424	68,20	165,00	25,051	85,00	22,20	26,718	4,53349	0,077292
425	69,50	165,00	25,528	86,00	22,30	26,284	4,60269	0,077223
426	71,60	163,00	26,949	86,00	22,40	28,487	4,74965	0,074940
427	78,50	177,00	25,057	85,00	22,50	26,324	3,76395	0,074613
428	80,50	172,00	27,211	87,00	22,70	28,701	4,19206	0,073327
429	72,70	170,00	25,156	85,00	22,70	26,452	4,19281	0,075934
430	77,20	170,00	26,713	84,00	22,80	27,952	4,12770	0,072096
431	67,90	158,50	27,028	82,00	23,00	28,702	4,80089	0,072320
432	85,00	180,00	26,235	86,00	23,20	27,950	3,65341	0,072602
433	72,20	158,00	28,922	86,00	23,30	31,117	5,14209	0,072614
434	71,60	168,00	25,368	87,00	23,30	25,679	4,45882	0,077744
435	85,00	178,00	26,827	87,00	23,50	27,746	3,82546	0,072765
436	74,50	172,00	25,183	87,00	23,50	25,824	4,19206	0,077213
437	78,70	173,00	26,296	85,00	23,60	26,069	4,00257	0,073082
438	72,20	168,50	25,429	86,00	23,60	26,934	4,35811	0,076614
439	68,90	165,00	25,308	86,00	23,60	25,597	4,60269	0,077670
440	74,70	169,00	26,155	83,00	23,70	26,985	4,12674	0,072461
441	79,70	173,00	26,630	86,00	23,70	26,771	4,06542	0,073321
442	68,50	163,00	25,782	83,00	23,80	26,619	4,53691	0,074492
443	67,10	158,00	26,879	86,00	23,80	28,750	5,14209	0,076248
444	70,90	168,00	25,120	86,00	23,90	26,407	4,39211	0,077356

445	72,90	170,00	25,225	82,00	24,10	26,673	3,99752	0,073120
446	77,00	172,00	26,028	87,00	24,10	27,565	4,19206	0,075532
447	75,50	169,50	26,279	86,00	24,20	27,638	4,29103	0,074732
448	83,90	176,00	27,085	85,00	24,40	27,505	3,82207	0,071040
449	71,90	167,00	25,781	85,00	24,40	26,998	4,39351	0,075370
450	78,30	175,00	25,567	87,00	24,60	27,238	4,00400	0,075778
451	67,70	163,00	25,481	85,00	24,60	26,610	4,67872	0,076886
452	77,50	163,00	29,169	86,00	24,70	30,821	4,74965	0,071086
453	64,00	160,00	25,000	86,00	24,90	26,251	4,98064	0,079520
454	91,00	186,00	26,304	86,00	25,10	26,203	3,33697	0,071296
455	71,60	167,00	25,673	82,00	25,10	27,243	4,19103	0,072913
456	85,00	177,00	27,131	86,00	25,40	28,027	3,82395	0,071592
457	64,00	154,00	26,986	80,00	25,40	28,534	5,00681	0,071653
458	72,40	167,00	25,960	86,00	25,50	27,229	4,46103	0,075905
459	91,80	176,00	29,636	86,00	25,90	30,539	3,88276	0,067691
460	66,10	161,50	25,343	86,00	26,40	26,824	4,86352	0,078435
461	81,40	171,00	27,838	87,00	26,50	29,377	4,25698	0,072432
462	76,20	171,00	26,059	85,00	26,60	26,735	4,12827	0,073951
463	64,60	151,00	28,332	81,00	33,30	29,047	5,34500	0,070927
464	73,00	170,00	25,260	82,00	35,80	25,400	3,99752	0,073053
465	72,80	164,00	27,067	86,00	37,20	26,903	4,67549	0,074493
466	85,70	170,00	29,654	84,00	53,70	31,224	4,12770	0,067246
467	92,40	172,50	31,052	87,00	29,00	32,493	4,16003	0,067049
468	88,00	171,00	30,095	86,00	40,00	31,307	4,19262	0,067974
469	73,00	173,00	24,391	94,00	11,90	25,880	4,56860	0,084974
470	77,90	182,00	23,518	91,00	14,00	23,860	3,82807	0,082176
471	75,00	177,00	23,939	92,00	14,60	23,709	4,18416	0,083251
472	72,90	180,00	22,500	88,00	15,10	21,663	3,76942	0,082298
473	70,50	178,00	22,251	90,00	15,10	20,655	4,00350	0,085271
474	74,30	174,00	24,541	90,00	15,30	24,546	4,25205	0,080793
475	69,60	170,00	24,083	90,00	15,70	24,196	4,51853	0,082771
476	65,90	169,00	23,073	90,00	15,80	24,172	4,58816	0,085420
477	76,00	175,00	24,816	92,00	16,00	24,109	4,31121	0,081742
478	76,10	180,00	23,488	88,00	16,10	22,179	3,76942	0,079975
479	66,80	171,00	22,845	88,00	16,30	22,314	4,32136	0,083585
480	72,50	186,00	20,956	90,00	16,30	19,576	3,55409	0,086818
481	70,30	171,00	24,042	90,00	16,40	25,287	4,45014	0,082623
482	74,50	177,00	23,780	91,00	16,60	22,316	4,12410	0,082714
483	70,80	180,00	21,852	98,00	16,60	21,220	4,35001	0,093454
484	68,10	175,50	22,110	89,00	16,70	21,916	4,09574	0,085282
485	73,40	178,00	23,166	89,00	16,90	23,380	3,94415	0,082087
486	73,20	181,00	22,344	88,00	16,90	21,115	3,71317	0,082453
487	72,90	185,00	21,300	88,00	16,90	20,083	3,49730	0,084199
488	77,00	180,00	23,765	91,00	17,00	23,319	3,94350	0,082056
489	75,30	178,00	23,766	91,00	17,00	23,612	4,06287	0,082514
490	73,30	179,00	22,877	92,00	17,10	22,360	4,06138	0,085329
491	70,20	176,00	22,663	89,00	17,20	22,213	4,06490	0,083771
492	86,70	187,00	24,793	90,00	17,30	24,213	3,50197	0,077404
493	69,00	175,00	22,531	88,00	17,30	22,025	4,06542	0,083390
494	70,70	180,00	21,821	90,00	17,40	20,676	3,88546	0,085906

495	56,00	162,00	21,338	88,00	17,50	21,342	4,96890	0,089871
496	76,00	181,00	23,198	97,00	17,60	23,120	4,22981	0,088640
497	67,20	169,00	23,529	88,00	17,70	22,239	4,45626	0,082441
498	69,80	172,50	23,457	90,00	17,70	24,674	4,34979	0,083624
499	77,60	188,00	21,956	90,00	17,70	21,864	3,45069	0,083714
500	73,30	178,00	23,135	91,00	17,80	21,962	4,06287	0,084008
501	70,20	170,00	24,291	90,00	18,00	23,352	4,51853	0,082298
502	69,00	171,00	23,597	89,00	18,00	23,376	4,38574	0,082728
503	65,90	163,00	24,803	92,00	18,00	25,044	5,17553	0,084727
504	65,00	171,00	22,229	88,00	18,00	21,433	4,32136	0,085121
505	77,60	178,00	24,492	91,00	18,10	23,504	4,06287	0,080875
506	67,20	172,00	22,715	88,00	18,10	22,915	4,25568	0,083659
507	72,20	171,00	24,691	93,00	18,10	23,930	4,64339	0,083873
508	73,00	177,00	23,301	88,00	18,30	22,054	3,94398	0,081080
509	71,00	176,00	22,921	88,00	18,30	22,423	4,00418	0,082206
510	73,50	181,00	22,435	90,00	18,30	21,250	3,82791	0,084098
511	75,60	179,00	23,595	93,00	18,30	22,485	4,12010	0,084498
512	74,50	179,00	23,251	93,00	18,30	23,648	4,12010	0,085328
513	69,50	180,50	21,332	90,00	18,30	20,817	3,85657	0,087093
514	57,40	163,00	21,604	91,00	18,30	21,372	5,10451	0,091888
515	80,00	180,00	24,691	90,00	18,40	24,891	3,88546	0,079112
516	73,80	174,00	24,376	89,00	18,40	24,037	4,18989	0,080256
517	85,30	186,00	24,656	96,00	18,40	24,567	3,88003	0,083093
518	78,50	180,00	24,228	94,00	18,40	24,112	4,11766	0,083677
519	77,50	180,50	23,787	88,00	18,50	22,766	3,74117	0,079192
520	78,00	182,00	23,548	88,00	18,50	23,604	3,65785	0,079399
521	71,70	173,00	23,957	88,00	18,50	22,578	4,19115	0,080508
522	82,00	184,50	24,089	92,00	18,50	23,489	3,74429	0,081204
523	71,90	175,00	23,478	90,00	18,50	22,164	4,18830	0,082976
524	70,40	179,00	21,972	90,00	18,50	21,557	3,94398	0,085751
525	73,50	178,00	23,198	97,00	18,50	24,335	4,41928	0,089385
526	68,40	169,00	23,949	88,00	18,60	25,311	4,45626	0,081474
527	80,70	180,00	24,907	90,00	18,80	24,780	3,88546	0,078654
528	78,60	182,00	23,729	89,00	18,80	22,534	3,71458	0,079892
529	74,60	174,00	24,640	90,00	18,80	25,509	4,25205	0,080576
530	74,00	177,00	23,620	93,00	18,80	24,443	4,24423	0,084913
531	77,00	179,00	24,032	95,00	18,80	23,695	4,23756	0,085266
532	62,40	164,00	23,200	94,00	18,80	23,579	5,23641	0,090235
533	75,70	175,00	24,718	90,00	18,90	25,749	4,18830	0,080176
534	80,60	181,00	24,602	92,00	18,90	26,149	3,94270	0,080841
535	73,50	177,00	23,461	89,00	18,90	23,484	4,00401	0,081629
536	74,60	177,00	23,812	96,00	18,90	24,114	4,42449	0,087181
537	78,30	179,00	24,437	91,00	19,00	24,822	4,00268	0,080769
538	72,00	176,00	23,244	88,00	19,00	22,122	4,00418	0,081443
539	72,40	172,00	24,473	94,00	19,00	25,984	4,63760	0,085031
540	89,00	190,00	24,654	89,00	19,20	24,564	3,29856	0,076224
541	76,00	181,00	23,198	92,00	19,20	21,751	3,94270	0,084071
542	70,80	178,00	22,346	91,00	19,20	21,279	4,06287	0,085974
543	73,60	175,00	24,033	88,00	19,40	23,696	4,06542	0,079878
544	70,00	172,00	23,661	88,00	19,40	22,729	4,25568	0,081412

545	66,00	166,00	23,951	89,00	19,40	23,870	4,73633	0,083135
546	87,30	189,00	24,439	97,00	19,40	23,847	3,76836	0,083781
547	68,70	174,50	22,561	89,00	19,40	21,593	4,15823	0,084382
548	68,00	171,00	23,255	91,00	19,40	22,744	4,51454	0,085415
549	74,60	176,00	24,083	95,00	19,40	25,198	4,42946	0,085867
550	75,40	181,00	23,015	95,00	19,40	22,710	4,11494	0,087272
551	75,70	176,00	24,438	99,00	19,40	24,823	4,67270	0,088613
552	76,30	175,00	24,914	89,00	19,50	24,790	4,12685	0,078869
553	69,90	169,00	24,474	90,00	19,50	25,010	4,58816	0,082129
554	69,30	168,00	24,554	92,00	19,50	24,148	4,79259	0,084021
555	61,30	165,00	22,516	92,00	19,50	23,119	5,01812	0,089822
556	78,00	180,00	24,074	92,00	19,60	22,752	4,00154	0,082247
557	81,60	186,00	23,587	93,00	19,60	22,473	3,71702	0,082912
558	84,00	186,00	24,280	97,00	19,60	23,055	3,93438	0,084823
559	84,40	185,50	24,528	90,00	19,70	23,695	3,58047	0,078277
560	76,60	178,00	24,176	88,00	19,70	23,044	3,88480	0,078888
561	75,30	175,50	24,448	89,00	19,70	23,998	4,09574	0,079755
562	73,70	176,00	23,793	89,00	19,70	25,110	4,06490	0,081097
563	75,00	176,00	24,212	91,00	19,70	23,948	4,18638	0,081959
564	68,50	171,00	23,426	88,00	19,70	23,286	4,32136	0,082196
565	73,90	177,00	23,588	91,00	19,70	23,216	4,12410	0,083162
566	77,70	182,00	23,457	95,00	19,70	23,927	4,05516	0,085935
567	78,70	179,00	24,562	89,00	19,80	24,714	3,88530	0,078726
568	79,20	180,00	24,444	90,00	19,80	24,273	3,88546	0,079644
569	75,50	174,00	24,937	90,00	19,80	25,496	4,25205	0,079935
570	69,40	170,00	24,014	88,00	19,80	24,676	4,38821	0,081087
571	65,00	162,00	24,768	88,00	19,80	26,087	4,96890	0,081371
572	73,00	174,00	24,112	91,00	19,80	25,521	4,31422	0,082658
573	75,00	178,00	23,671	91,00	19,80	22,449	4,06287	0,082734
574	74,60	175,00	24,359	92,00	19,80	23,873	4,31121	0,082761
575	63,70	168,00	22,569	88,00	19,80	22,080	4,52555	0,085012
576	69,90	173,00	23,355	92,00	19,80	22,886	4,44274	0,085607
577	59,80	157,00	24,261	90,00	19,80	25,571	5,53155	0,085709
578	67,40	170,50	23,185	91,00	19,80	23,710	4,54897	0,085711
579	76,20	181,00	23,259	95,00	19,80	24,416	4,11494	0,086660
580	78,80	180,00	24,321	91,00	19,90	24,382	3,94350	0,080801
581	74,30	175,00	24,261	91,00	20,00	23,451	4,24975	0,082082
582	70,10	175,00	22,890	88,00	20,00	22,534	4,06542	0,082516
583	70,80	172,00	23,932	90,00	20,00	23,554	4,38294	0,082634
584	74,60	175,00	24,359	92,00	20,00	25,277	4,31121	0,082761
585	82,00	186,00	23,702	95,00	20,00	24,699	3,82568	0,084419
586	86,90	187,00	24,851	100,00	20,00	26,329	4,03948	0,085873
587	62,70	162,00	23,891	92,00	20,00	24,947	5,25645	0,087138
588	74,00	172,50	24,869	98,00	20,00	25,268	4,85629	0,087578
589	66,10	165,00	24,279	90,00	20,10	25,595	4,87959	0,083563
590	70,90	179,00	22,128	90,00	20,10	21,451	3,94398	0,085347
591	76,20	176,00	24,600	107,00	20,10	25,594	5,15969	0,095354
592	77,50	177,00	24,737	92,00	20,20	24,680	4,18416	0,081451
593	78,90	180,00	24,352	93,00	20,20	25,127	4,05959	0,082507
594	69,00	169,00	24,159	94,00	20,20	25,439	4,85209	0,086523

595	64,30	164,00	23,907	94,00	20,20	23,953	5,23641	0,088448
596	76,10	177,00	24,291	88,00	20,30	24,199	3,94398	0,078863
597	77,60	181,00	23,687	89,00	20,30	23,060	3,77054	0,080207
598	69,10	167,00	24,777	89,00	20,30	25,007	4,66366	0,081034
599	74,00	176,50	23,754	90,00	20,30	24,035	4,09471	0,081980
600	77,20	183,00	23,052	90,00	20,30	22,455	3,71567	0,082137
601	71,50	170,00	24,740	94,00	20,30	25,231	4,77932	0,084911
602	73,00	176,00	23,567	93,00	20,30	24,520	4,30790	0,085283
603	74,10	176,00	23,922	95,00	20,30	23,684	4,42946	0,086253
604	78,20	182,00	23,608	88,00	20,40	24,871	3,65785	0,079263
605	71,80	171,50	24,412	88,00	20,40	25,626	4,28837	0,079852
606	77,00	182,00	23,246	88,00	20,40	23,337	3,65785	0,080084
607	73,70	176,00	23,793	88,00	20,40	24,818	4,00418	0,080186
608	76,60	176,00	24,729	94,00	20,40	24,121	4,36867	0,083477
609	70,60	176,00	22,792	90,00	20,40	23,333	4,12564	0,084392
610	79,20	180,00	24,444	96,00	20,40	24,273	4,23381	0,084954
611	73,70	177,00	23,525	93,00	20,40	23,126	4,24423	0,085143
612	80,50	180,00	24,846	90,00	20,50	25,644	3,88546	0,078784
613	70,70	171,80	23,954	88,00	20,50	23,151	4,26872	0,080796
614	64,80	161,00	24,999	89,00	20,50	26,250	5,12048	0,082040
615	65,60	166,00	23,806	89,00	20,50	24,543	4,73633	0,083473
616	77,00	182,00	23,246	98,00	20,50	23,337	4,22556	0,089185
617	86,60	188,00	24,502	89,00	20,60	25,604	3,39756	0,076944
618	74,00	173,00	24,725	91,00	20,60	25,348	4,37982	0,081519
619	70,50	174,00	23,286	89,00	20,60	23,695	4,18989	0,082741
620	76,50	176,70	24,501	96,00	20,60	25,047	4,44410	0,085611
621	72,00	171,00	24,623	96,00	20,60	23,832	4,83675	0,086739
622	74,00	182,00	22,340	94,00	20,60	21,432	3,99837	0,087841
623	69,60	175,00	22,727	94,00	20,60	22,774	4,43416	0,088563
624	72,00	170,00	24,913	90,00	20,70	25,734	4,51853	0,080921
625	73,40	178,00	23,166	88,00	20,70	22,770	3,88480	0,081165
626	73,50	180,50	22,560	89,00	20,70	22,225	3,79887	0,082972
627	70,70	169,00	24,754	89,00	20,80	25,796	4,52221	0,080603
628	77,50	179,50	24,053	91,00	20,80	23,008	3,97296	0,081513
629	76,30	176,00	24,632	92,00	20,80	24,396	4,24714	0,081915
630	71,90	175,00	23,478	89,00	20,80	24,253	4,12685	0,082055
631	88,00	189,00	24,635	96,00	20,80	24,401	3,71575	0,082477
632	76,30	176,00	24,632	92,00	20,90	24,396	4,24714	0,081915
633	80,40	179,50	24,953	96,00	20,90	26,056	4,26492	0,083911
634	70,10	168,00	24,837	93,00	20,90	25,633	4,85938	0,084287
635	78,10	179,00	24,375	95,00	20,90	24,456	4,23756	0,084463
636	73,00	174,00	24,112	94,00	20,90	24,378	4,50079	0,085383
637	78,80	180,00	24,321	98,00	20,90	24,382	4,35001	0,087017
638	68,00	170,00	23,529	94,00	20,90	23,281	4,77932	0,087800
639	76,60	177,00	24,450	90,00	21,00	24,141	4,06405	0,080303
640	77,40	176,00	24,987	92,00	21,00	26,503	4,24714	0,081137
641	70,00	169,00	24,509	89,00	21,00	24,363	4,52221	0,081139
642	75,80	176,00	24,471	91,00	21,00	23,613	4,18638	0,081381
643	73,50	182,00	22,189	88,00	21,00	21,213	3,65785	0,082607
644	87,00	188,00	24,615	96,00	21,00	25,338	3,76963	0,082741

645	68,70	170,00	23,772	94,00	21,00	24,790	4,77932	0,087203
646	78,60	178,00	24,807	108,00	21,00	25,185	5,07360	0,095168
647	72,50	171,00	24,794	88,00	21,10	24,077	4,32136	0,079145
648	77,40	176,00	24,987	91,00	21,10	24,756	4,18638	0,080255
649	78,80	179,00	24,593	91,00	21,10	24,757	4,00268	0,080427
650	72,00	171,00	24,623	91,00	21,10	24,935	4,51454	0,082221
651	77,10	181,00	23,534	96,00	21,10	22,693	4,17237	0,086889
652	77,00	182,00	23,246	90,00	21,20	22,883	3,77132	0,081905
653	74,20	179,00	23,158	93,00	21,20	24,130	4,12010	0,085557
654	74,70	179,00	23,314	94,00	21,20	23,884	4,17882	0,086091
655	68,60	170,00	23,737	93,00	21,20	25,038	4,71411	0,086359
656	78,40	178,00	24,744	92,00	21,30	24,280	4,12225	0,081207
657	77,00	179,00	24,032	93,00	21,30	23,838	4,12010	0,083471
658	68,60	167,00	24,598	92,00	21,30	25,315	4,86640	0,084172
659	73,30	175,00	23,935	95,00	21,30	23,847	4,49566	0,086467
660	65,00	167,00	23,307	89,00	21,40	24,478	4,66366	0,084407
661	80,60	186,00	23,297	95,00	21,40	24,768	3,82568	0,085394
662	71,50	170,00	24,740	96,00	21,40	24,001	4,90978	0,086718
663	61,30	160,00	23,945	93,00	21,40	25,162	5,49670	0,088500
664	68,00	173,00	22,720	94,00	21,40	22,294	4,56860	0,089090
665	78,80	180,00	24,321	88,00	21,50	24,241	3,76942	0,078137
666	77,30	177,00	24,674	90,00	21,50	25,004	4,06405	0,079818
667	70,50	172,00	23,830	88,00	21,50	24,722	4,25568	0,081027
668	84,90	186,00	24,540	94,00	21,50	25,238	3,77135	0,081618
669	76,00	180,00	23,457	91,00	21,50	21,984	3,94350	0,082774
670	61,80	163,50	23,118	89,00	21,50	23,772	4,92395	0,085769
671	76,40	175,00	24,947	98,00	21,50	25,509	4,68020	0,086769
672	71,60	177,00	22,854	93,00	21,50	23,262	4,24423	0,086800
673	71,30	179,00	22,253	92,00	21,50	20,334	4,06138	0,086917
674	86,00	193,00	23,088	99,00	21,50	22,812	3,65993	0,087889
675	75,50	179,00	23,564	99,00	21,50	24,664	4,47259	0,090029
676	68,40	166,00	24,822	88,00	21,60	24,662	4,66794	0,080267
677	81,80	181,00	24,969	94,00	21,60	24,193	4,05752	0,081788
678	76,40	176,00	24,664	93,00	21,60	25,816	4,30790	0,082733
679	73,00	172,00	24,676	92,00	21,60	25,281	4,51025	0,082765
680	74,00	175,00	24,163	94,00	21,60	24,591	4,43416	0,085017
681	75,50	175,00	24,653	96,00	21,60	24,976	4,55716	0,085672
682	72,00	172,00	24,337	89,00	21,70	24,405	4,31930	0,080806
683	69,50	174,00	22,955	88,00	21,70	21,854	4,12774	0,082594
684	70,80	172,50	23,793	92,00	21,70	24,380	4,47635	0,084675
685	73,90	174,00	24,409	88,00	21,80	25,343	4,12774	0,079283
686	78,40	181,00	23,931	89,00	21,80	24,854	3,77054	0,079661
687	78,40	180,00	24,198	91,00	21,80	24,354	3,94350	0,081076
688	73,00	171,00	24,965	91,00	21,80	26,341	4,51454	0,081468
689	76,40	176,00	24,664	93,00	21,80	25,816	4,30790	0,082733
690	80,40	179,50	24,953	95,00	21,80	25,383	4,20651	0,083037
691	74,60	175,00	24,359	93,00	21,80	25,137	4,37268	0,083661
692	67,00	165,00	24,610	92,00	21,80	25,055	5,01812	0,084653
693	69,50	168,00	24,624	93,00	21,80	25,350	4,85938	0,084772
694	62,70	166,00	22,754	90,00	21,80	22,968	4,80473	0,086994

695	77,00	177,00	24,578	91,00	21,90	24,597	4,12410	0,080914
696	73,70	172,00	24,912	91,00	21,90	25,327	4,44659	0,081346
697	71,40	170,50	24,561	90,00	21,90	25,405	4,48418	0,081573
698	69,70	171,00	23,836	89,00	21,90	25,166	4,38574	0,082173
699	76,90	177,00	24,546	95,00	21,90	23,998	4,36440	0,084544
700	69,30	169,50	24,121	88,00	22,00	25,533	4,42208	0,080966
701	67,20	170,00	23,253	88,00	22,00	24,407	4,38821	0,082847
702	78,00	179,00	24,344	94,00	22,00	24,413	4,17882	0,083645
703	67,40	170,00	23,322	91,00	22,00	24,347	4,58371	0,085502
704	71,80	172,50	24,129	94,00	22,00	24,831	4,60295	0,085711
705	71,80	173,00	23,990	90,00	22,10	24,644	4,31692	0,082262
706	82,40	182,00	24,876	97,00	22,10	25,956	4,16875	0,084375
707	70,00	173,00	23,389	92,00	22,10	23,684	4,44274	0,085525
708	76,00	176,00	24,535	97,00	22,10	24,399	4,55106	0,086594
709	70,30	168,00	24,908	97,00	22,10	25,186	5,12667	0,087746
710	78,00	178,00	24,618	90,00	22,20	23,687	4,00350	0,079713
711	64,50	161,00	24,883	88,00	22,20	24,341	5,04770	0,081370
712	77,09	176,00	24,887	96,00	22,20	25,293	4,49026	0,084892
713	85,00	185,00	24,836	92,00	22,30	26,446	3,71688	0,079461
714	77,00	176,00	24,858	90,00	22,30	25,254	4,12564	0,079648
715	69,60	167,50	24,807	89,00	22,30	24,369	4,62781	0,080847
716	72,70	174,00	24,012	89,00	22,30	24,243	4,18989	0,081063
717	67,80	169,00	23,739	88,00	22,30	24,747	4,45626	0,081954
718	63,00	159,50	24,764	89,00	22,30	25,945	5,24292	0,082946
719	70,00	170,00	24,221	92,00	22,30	25,095	4,64890	0,084288
720	77,00	179,00	24,032	94,00	22,30	24,844	4,17882	0,084368
721	72,50	180,00	22,377	91,00	22,30	20,200	3,94350	0,085417
722	75,00	174,00	24,772	92,00	22,40	25,820	4,37640	0,082074
723	65,20	163,00	24,540	89,00	22,40	25,238	4,96253	0,082550
724	63,00	162,00	24,005	90,00	22,40	24,953	5,11264	0,084972
725	67,20	176,00	21,694	88,00	22,40	21,331	4,00418	0,085277
726	72,70	172,50	24,432	88,00	22,50	23,836	4,22327	0,079576
727	75,80	177,00	24,195	93,00	22,50	24,918	4,24423	0,083563
728	76,30	175,00	24,914	88,00	22,60	25,195	4,06542	0,077983
729	74,10	174,00	24,475	88,00	22,60	25,708	4,12774	0,079140
730	71,10	169,50	24,747	90,00	22,60	26,197	4,55319	0,081402
731	72,70	175,50	23,604	92,00	22,60	24,421	4,27903	0,084397
732	80,10	183,00	23,918	95,00	22,60	24,114	3,99636	0,084595
733	76,40	178,50	23,978	90,00	22,70	24,773	3,97362	0,081011
734	79,00	181,00	24,114	93,00	22,70	24,667	4,00010	0,082819
735	73,00	174,00	24,112	94,00	22,70	25,235	4,50079	0,085383
736	63,20	166,00	22,935	90,00	22,70	23,681	4,80473	0,086534
737	72,00	170,00	24,913	88,00	22,80	24,248	4,38821	0,079123
738	73,50	172,00	24,845	91,00	22,80	26,457	4,44659	0,081494
739	74,50	173,00	24,892	92,00	22,80	26,518	4,44274	0,082046
740	73,30	175,00	23,935	92,00	22,80	25,004	4,31121	0,083737
741	70,30	168,00	24,908	94,00	22,80	24,240	4,92619	0,085032
742	63,80	166,00	23,153	90,00	22,80	23,514	4,80473	0,085991
743	84,00	185,00	24,543	95,00	22,90	23,579	3,88165	0,082702
744	77,80	176,50	24,974	94,00	22,90	24,873	4,33635	0,082812

745	68,70	168,00	24,341	92,00	22,90	25,394	4,79259	0,084510
746	67,50	172,00	22,816	96,00	22,90	22,118	4,76500	0,090993
747	66,00	163,00	24,841	89,00	23,00	24,416	4,96253	0,081881
748	76,00	179,00	23,720	91,00	23,00	24,281	4,00268	0,082390
749	67,60	170,00	23,391	89,00	23,00	22,487	4,45336	0,083458
750	75,00	176,00	24,212	93,00	23,00	25,083	4,30790	0,083760
751	70,20	173,00	23,456	89,00	23,10	22,879	4,25403	0,082579
752	76,50	176,00	24,697	94,00	23,10	25,721	4,36867	0,083550
753	74,90	175,00	24,457	98,00	23,10	25,406	4,68020	0,087923
754	71,90	170,00	24,879	105,00	23,10	24,063	5,49746	0,094495
755	65,50	164,00	24,353	88,00	23,20	25,410	4,81564	0,081788
756	72,80	176,00	23,502	90,00	23,20	23,094	4,12564	0,082683
757	74,30	178,00	23,450	92,00	23,20	23,619	4,12225	0,084168
758	64,40	162,00	24,539	95,00	23,20	25,514	5,47226	0,088388
759	80,00	179,00	24,968	90,00	23,30	24,999	3,94398	0,078746
760	70,30	169,00	24,614	89,00	23,40	26,164	4,52221	0,080908
761	73,90	174,00	24,409	91,00	23,40	24,363	4,31422	0,081985
762	74,40	177,50	23,614	90,00	23,40	22,661	4,03365	0,082072
763	74,40	174,00	24,574	92,00	23,40	23,623	4,37640	0,082514
764	75,00	174,00	24,772	93,00	23,40	26,092	4,43859	0,082966
765	75,20	173,50	24,982	88,00	23,50	26,362	4,15931	0,078178
766	73,50	171,50	24,990	89,00	23,50	25,029	4,35238	0,079509
767	74,90	174,00	24,739	92,00	23,50	25,503	4,37640	0,082147
768	64,40	166,50	23,230	90,00	23,50	23,923	4,76781	0,085670
769	77,00	176,00	24,858	91,00	23,60	24,576	4,18638	0,080533
770	69,30	171,00	23,700	96,00	23,60	24,401	4,83675	0,088977
771	82,00	182,50	24,620	102,00	23,60	26,034	4,42117	0,089216
772	80,90	184,00	23,895	88,00	23,70	24,227	3,54994	0,078199
773	67,90	168,00	24,058	89,00	23,70	24,448	4,59229	0,082395
774	89,30	189,00	24,999	97,00	23,70	25,042	3,76836	0,082526
775	70,60	170,00	24,429	92,00	23,80	25,230	4,64890	0,083809
776	76,30	175,00	24,914	88,00	23,90	24,925	4,06542	0,077983
777	66,60	165,00	24,463	91,00	24,00	24,856	4,94885	0,084068
778	73,90	174,00	24,409	94,00	24,00	24,783	4,50079	0,084688
779	72,10	175,00	23,543	93,00	24,00	23,152	4,37268	0,085584
780	69,10	168,00	24,483	95,00	24,00	25,440	4,99300	0,086929
781	76,20	175,00	24,882	99,00	24,00	25,286	4,74173	0,087807
782	69,20	175,50	22,467	89,00	24,20	23,372	4,09574	0,084376
783	71,70	176,00	23,147	92,00	24,20	24,116	4,24714	0,085383
784	69,20	168,00	24,518	94,00	24,20	25,764	4,92619	0,085931
785	65,80	163,00	24,766	90,00	24,40	26,221	5,03351	0,082969
786	75,50	175,00	24,653	89,00	24,50	25,389	4,12685	0,079425
787	77,10	178,00	24,334	91,00	24,50	23,978	4,06287	0,081225
788	83,10	183,00	24,814	98,00	24,60	25,602	4,16488	0,085153
789	75,20	173,50	24,982	97,00	24,60	25,824	4,72232	0,086174
790	58,90	177,00	18,800	99,00	24,60	16,725	4,60485	0,105245
791	71,70	169,50	24,956	94,00	24,70	26,060	4,81554	0,084545
792	74,50	174,00	24,607	97,00	24,70	26,017	4,68747	0,086921
793	76,80	177,00	24,514	102,00	24,70	25,898	4,78530	0,090853
794	79,10	182,50	23,749	105,00	24,70	23,149	4,59082	0,094071

795	71,90	171,00	24,589	88,00	24,80	24,612	4,32136	0,079584
796	79,70	179,50	24,736	93,00	24,80	25,225	4,08972	0,081764
797	83,00	183,00	24,784	92,00	24,90	25,427	3,82792	0,080004
798	76,40	180,00	23,580	90,00	25,00	22,908	3,88546	0,081578
799	68,00	168,00	24,093	89,00	25,00	24,353	4,59229	0,082314
800	66,90	166,00	24,278	92,00	25,00	25,593	4,94157	0,085165
801	71,30	169,00	24,964	89,00	25,10	25,666	4,52221	0,080150
802	71,70	169,50	24,956	94,00	25,20	24,713	4,81554	0,084545
803	77,10	176,00	24,890	92,00	25,30	25,433	4,24714	0,081348
804	73,00	172,00	24,676	95,00	25,30	25,556	4,70129	0,085464
805	73,00	173,00	24,391	98,00	25,40	25,459	4,82045	0,088590
806	79,20	180,00	24,444	89,00	25,50	24,831	3,82743	0,078759
807	69,30	166,50	24,998	93,00	25,50	26,786	4,97184	0,084302
808	63,50	162,00	24,196	93,00	25,50	25,488	5,32837	0,087343
809	69,30	167,00	24,849	90,00	25,70	24,834	4,73123	0,081787
810	67,50	165,00	24,793	98,00	26,10	25,711	5,43404	0,089728
811	74,70	173,00	24,959	92,00	26,40	26,333	4,44274	0,081899
812	72,00	170,00	24,913	88,00	27,00	26,274	4,38821	0,079123
813	70,30	168,00	24,908	90,00	27,10	25,727	4,65905	0,081414
814	76,80	180,00	23,704	91,00	29,90	22,644	3,94350	0,082198
815	79,20	180,00	24,444	98,00	30,00	25,110	4,35001	0,086723
816	74,30	173,00	24,825	90,00	33,00	26,297	4,31692	0,080406
817	68,00	165,00	24,977	98,00	33,00	25,280	5,43404	0,089287
818	75,00	176,00	24,212	88,00	33,90	23,523	4,00418	0,079257
819	72,00	171,00	24,623	90,00	34,00	24,659	4,45014	0,081318
820	72,00	174,00	23,781	92,00	34,30	24,656	4,37640	0,084338
821	71,00	179,00	22,159	90,00	34,90	22,310	3,94398	0,085267
822	84,80	185,50	24,644	94,00	35,90	23,724	3,79892	0,081499
823	74,30	173,00	24,825	98,00	36,20	25,617	4,82045	0,087553
824	79,90	185,00	23,346	92,00	36,80	22,421	3,71688	0,082807
825	73,00	171,00	24,965	91,00	38,90	25,398	4,51454	0,081468
826	65,90	176,00	21,275	114,00	39,40	21,598	5,58634	0,111920
827	84,80	175,00	27,690	95,00	8,60	29,192	4,49566	0,078462
828	83,30	174,00	27,514	105,00	10,20	29,082	5,18574	0,087341
829	71,40	166,00	25,911	98,00	10,40	26,537	5,35240	0,086866
830	91,40	180,00	28,210	104,00	12,50	28,675	4,69881	0,083649
831	86,90	179,00	27,122	100,00	12,60	28,244	4,53137	0,082800
832	76,00	170,00	26,298	91,00	12,70	27,173	4,58371	0,078924
833	75,30	164,00	27,997	97,00	12,70	30,006	5,44698	0,082151
834	80,10	165,00	29,421	90,00	12,90	31,708	4,87959	0,073518
835	92,40	177,00	29,493	99,00	13,80	30,260	4,60485	0,077953
836	82,50	178,00	26,038	99,00	14,10	27,953	4,53816	0,084466
837	88,30	173,00	29,503	107,00	14,20	30,943	5,38777	0,085202
838	87,60	182,00	26,446	95,00	14,70	26,641	4,05516	0,079332
839	78,00	170,00	26,990	91,00	15,60	27,028	4,58371	0,077569
840	75,00	171,00	25,649	95,00	15,60	25,803	4,77229	0,083531
841	80,50	170,00	27,855	102,00	15,70	29,616	5,30146	0,085135
842	76,80	170,00	26,574	94,00	15,90	27,175	4,77932	0,080958
843	91,50	178,00	28,879	100,00	16,00	31,469	4,59761	0,079629
844	88,10	178,00	27,806	95,00	16,50	29,009	4,30044	0,077581

845	78,00	174,00	25,763	92,00	16,60	26,340	4,37640	0,079956
846	88,00	174,00	29,066	103,00	16,80	30,991	5,06110	0,082599
847	76,80	172,00	25,960	94,00	17,00	25,725	4,63760	0,081751
848	85,30	175,00	27,853	97,00	17,20	29,940	4,61867	0,079800
849	74,20	170,00	25,675	91,00	17,20	26,734	4,58371	0,080195
850	90,30	176,00	29,152	103,00	17,60	30,898	4,91611	0,081967
851	88,30	180,50	27,102	95,00	17,70	27,989	4,14521	0,078370
852	85,70	179,00	26,747	97,00	17,90	27,996	4,35505	0,081064
853	71,50	169,00	25,034	96,00	17,90	25,894	4,98414	0,086292
854	92,00	184,00	27,174	96,00	18,00	27,509	3,99408	0,078300
855	88,00	173,00	29,403	99,00	18,00	31,300	4,88344	0,079011
856	92,00	178,00	29,037	110,00	18,00	31,353	5,19270	0,087274
857	81,10	173,00	27,097	94,00	18,10	29,135	4,56860	0,079217
858	75,50	165,00	27,732	94,00	18,10	29,354	5,15671	0,079873
859	79,50	170,00	27,509	98,00	18,50	27,623	5,04030	0,082481
860	75,00	168,00	26,573	88,00	18,60	28,252	4,52555	0,076243
861	78,70	177,00	25,120	88,00	18,70	24,809	3,94398	0,077116
862	76,90	175,00	25,110	92,00	18,70	24,662	4,31121	0,081103
863	64,70	160,00	25,273	89,00	18,80	26,077	5,20172	0,081700
864	69,00	166,00	25,040	91,00	18,80	25,500	4,87314	0,082521
865	74,20	169,00	25,979	95,00	18,80	26,378	4,91811	0,083309
866	83,50	170,00	28,893	104,00	18,90	31,384	5,43212	0,084713
867	68,70	164,00	25,543	99,00	18,90	27,078	5,58742	0,089132
868	89,70	184,00	26,495	97,00	19,00	27,430	4,04963	0,080462
869	78,50	168,00	27,813	96,00	19,00	30,110	5,05983	0,080683
870	81,70	180,00	25,216	96,00	19,00	24,811	4,23381	0,083212
871	91,00	177,00	29,047	108,00	19,00	31,265	5,14647	0,085909
872	76,30	173,00	25,494	90,00	19,10	24,812	4,31692	0,078995
873	79,90	172,50	26,852	89,00	19,20	27,425	4,28652	0,075570
874	86,50	181,00	26,403	100,00	19,20	28,160	4,40219	0,083828
875	77,20	173,00	25,794	92,00	19,30	27,143	4,44274	0,080121
876	86,00	185,00	25,128	95,00	19,30	24,288	3,88165	0,081414
877	75,50	170,00	26,125	105,00	19,30	27,317	5,49746	0,091467
878	81,30	178,00	25,660	91,00	19,40	26,073	4,06287	0,078403
879	90,00	186,50	25,875	99,00	19,40	26,111	4,01428	0,082865
880	79,10	176,00	25,536	88,00	19,50	25,519	4,00418	0,076494
881	83,00	180,50	25,476	100,00	19,50	26,474	4,43408	0,085970
882	72,70	166,00	26,383	89,00	19,60	28,256	4,73633	0,077945
883	81,50	174,00	26,919	93,00	19,60	28,683	4,43859	0,078494
884	82,10	178,50	25,767	88,00	19,70	25,456	3,85559	0,075501
885	88,20	182,00	26,627	97,00	19,80	27,603	4,16875	0,080634
886	73,20	170,00	25,329	92,00	19,80	25,757	4,64890	0,081813
887	85,00	178,50	26,677	94,00	19,90	28,739	4,20979	0,078804
888	75,30	170,00	26,055	91,00	19,90	25,734	4,58371	0,079412
889	81,80	175,00	26,710	94,00	19,90	26,881	4,43416	0,079523
890	80,00	172,00	27,042	94,00	19,90	27,331	4,63760	0,079556
891	95,80	190,00	26,537	90,00	20,00	26,525	3,35056	0,073388
892	88,50	181,00	27,014	97,00	20,10	28,105	4,22981	0,080083
893	98,00	183,00	29,263	105,00	20,10	30,351	4,55841	0,081736
894	74,60	167,00	26,749	96,00	20,10	27,525	5,13691	0,083057

895	95,00	189,00	26,595	91,00	20,20	26,724	3,45279	0,074292
896	82,70	176,50	26,547	92,00	20,20	28,219	4,21551	0,077816
897	83,50	182,00	25,208	91,00	20,20	25,197	3,82807	0,078459
898	91,00	188,00	25,747	94,00	20,20	26,191	3,66328	0,078626
899	80,80	177,00	25,791	92,00	20,20	26,504	4,18416	0,079218
900	86,20	185,00	25,186	95,00	20,20	24,372	3,88165	0,081288
901	81,90	179,00	25,561	88,00	20,30	26,198	3,82662	0,075800
902	67,80	162,00	25,834	89,00	20,30	27,068	5,04076	0,080014
903	88,00	184,00	25,992	88,00	20,40	26,021	3,54994	0,073934
904	77,60	171,00	26,538	92,00	20,40	27,127	4,57896	0,079076
905	79,60	177,00	25,408	91,00	20,40	25,994	4,12410	0,079143
906	73,70	168,00	26,113	93,00	20,40	25,814	4,85938	0,081520
907	77,50	176,00	25,019	94,00	20,40	26,142	4,36867	0,082830
908	77,50	176,00	25,019	89,00	20,50	25,204	4,06490	0,078424
909	89,10	180,00	27,500	96,00	20,50	27,723	4,23381	0,078538
910	86,30	185,00	25,215	92,00	20,50	25,075	3,71688	0,078661
911	81,90	178,00	25,849	92,00	20,50	25,570	4,12225	0,078877
912	71,00	164,00	26,398	90,00	20,50	26,576	4,95584	0,079270
913	80,00	174,00	26,424	93,00	20,50	26,126	4,43859	0,079472
914	77,10	173,00	25,761	96,00	20,50	26,591	4,69450	0,083677
915	80,20	173,00	26,797	103,00	20,50	28,531	5,13552	0,087450
916	89,10	183,00	26,606	93,00	20,60	26,380	3,88406	0,077139
917	80,90	170,00	27,993	94,00	20,60	29,140	4,77932	0,078199
918	85,60	175,00	27,951	96,00	20,60	29,302	4,55716	0,078793
919	76,90	173,00	25,694	94,00	20,60	26,248	4,56860	0,082076
920	75,90	173,00	25,360	94,00	20,60	25,799	4,56860	0,082795
921	75,80	174,00	25,036	95,00	20,60	24,825	4,56301	0,084153
922	76,70	166,00	27,834	104,00	20,60	29,809	5,76370	0,087887
923	83,80	177,00	26,748	88,00	20,70	27,170	3,94398	0,073954
924	85,40	176,00	27,570	91,00	20,70	28,040	4,18638	0,075162
925	79,90	178,00	25,218	90,00	20,70	26,400	4,00350	0,078444
926	69,40	164,00	25,803	90,00	20,70	25,633	4,95584	0,080483
927	72,00	165,50	26,287	92,00	20,70	26,057	4,97967	0,080891
928	76,10	170,50	26,178	95,00	20,70	26,892	4,80825	0,082522
929	96,60	184,00	28,533	105,00	20,70	30,136	4,49439	0,082900
930	80,50	173,00	26,897	90,00	20,80	27,720	4,31692	0,076223
931	76,40	174,00	25,235	88,00	20,80	25,497	4,12774	0,077543
932	76,70	175,00	25,045	89,00	20,80	26,577	4,12685	0,078594
933	76,50	170,00	26,471	92,00	20,80	28,003	4,64890	0,079443
934	73,60	170,00	25,467	90,00	20,80	26,852	4,51853	0,079744
935	84,20	181,00	25,701	94,00	20,80	26,257	4,05752	0,080227
936	80,00	173,00	26,730	95,00	20,80	28,566	4,63155	0,080792
937	82,50	177,00	26,333	88,00	20,90	27,463	3,94398	0,074729
938	78,20	173,00	26,129	90,00	20,90	27,941	4,31692	0,077710
939	75,50	171,00	25,820	90,00	20,90	26,669	4,45014	0,078785
940	78,40	175,00	25,600	91,00	20,90	27,022	4,24975	0,079195
941	78,50	171,00	26,846	93,00	20,90	27,300	4,64339	0,079323
942	95,90	188,00	27,133	99,00	20,90	27,914	3,92920	0,079963
943	80,10	176,00	25,859	94,00	20,90	26,341	4,36867	0,081028
944	77,50	176,00	25,019	92,00	20,90	24,265	4,24714	0,081068

945	83,00	171,00	28,385	90,00	21,00	30,684	4,45014	0,073964
946	98,00	186,00	28,327	97,00	21,00	28,831	3,93438	0,076539
947	87,60	180,00	27,037	94,00	21,00	27,787	4,11766	0,077777
948	74,30	169,00	26,014	90,00	21,00	26,051	4,58816	0,078854
949	73,00	170,00	25,260	89,00	21,00	25,136	4,45336	0,079289
950	91,00	177,00	29,047	101,00	21,00	30,769	4,72514	0,080341
951	82,00	178,00	25,881	100,00	21,00	26,371	4,59761	0,085666
952	76,10	170,00	26,332	89,00	21,10	26,120	4,45336	0,077121
953	79,40	172,00	26,839	91,00	21,10	28,936	4,44659	0,077405
954	79,00	169,50	27,497	92,00	21,10	29,508	4,68434	0,077567
955	84,00	181,00	25,640	92,00	21,10	26,047	3,94270	0,078644
956	81,00	178,00	25,565	93,00	21,10	26,461	4,18163	0,080324
957	79,60	174,00	26,291	94,00	21,10	28,143	4,50079	0,080595
958	84,90	173,50	28,204	99,00	21,10	29,833	4,84755	0,081117
959	70,40	165,00	25,859	92,00	21,10	27,730	5,01812	0,081905
960	86,30	185,00	25,215	96,00	21,10	25,736	3,93659	0,082081
961	76,90	167,00	27,574	98,00	21,10	29,268	5,27224	0,083088
962	86,20	180,00	26,605	100,00	21,10	26,738	4,46624	0,083635
963	92,70	185,00	27,085	92,00	21,20	26,928	3,71688	0,074997
964	83,20	178,00	26,259	89,50	21,20	26,019	3,97382	0,075932
965	79,80	177,00	25,472	89,00	21,20	25,819	4,00401	0,077274
966	85,70	175,00	27,984	98,00	21,20	29,559	4,68020	0,080372
967	82,00	179,00	25,592	94,00	21,20	26,883	4,17882	0,080903
968	88,00	175,00	28,735	101,00	21,20	29,369	4,86484	0,081383
969	86,90	177,00	27,738	102,00	21,20	28,813	4,78530	0,083669
970	96,00	184,00	28,355	106,00	21,20	29,078	4,55003	0,084038
971	83,30	175,00	27,200	104,00	21,20	28,002	5,04957	0,086923
972	79,50	174,00	26,258	90,00	21,30	26,508	4,25205	0,077230
973	77,90	165,00	28,613	95,00	21,30	30,133	5,22602	0,079056
974	81,90	178,00	25,849	93,00	21,30	27,592	4,18163	0,079734
975	88,70	184,00	26,199	96,00	21,30	26,427	3,99408	0,080230
976	72,90	169,00	25,524	93,00	21,30	27,184	4,78609	0,082522
977	79,60	175,00	25,992	96,00	21,30	27,895	4,55716	0,082704
978	80,00	176,00	25,826	96,00	21,30	27,310	4,49026	0,082821
979	81,80	174,00	27,018	99,00	21,30	27,994	4,81197	0,083354
980	94,20	186,00	27,229	90,00	21,40	27,354	3,55409	0,072913
981	80,00	170,00	27,682	92,00	21,40	29,071	4,64890	0,077108
982	77,00	173,00	25,728	90,00	21,40	26,165	4,31692	0,078515
983	81,80	179,00	25,530	94,00	21,40	26,286	4,17882	0,081034
984	76,00	171,50	25,840	94,00	21,40	25,557	4,67256	0,082124
985	75,80	172,00	25,622	95,00	21,40	25,894	4,70129	0,083346
986	89,10	182,00	26,899	105,00	21,40	26,904	4,62350	0,086696
987	89,60	173,00	29,938	91,00	21,50	31,572	4,37982	0,071759
988	89,20	179,00	27,839	92,00	21,50	28,289	4,06138	0,074861
989	83,90	180,00	25,895	88,00	21,50	26,768	3,76942	0,074938
990	75,50	169,50	26,279	88,00	21,50	26,168	4,42208	0,076470
991	74,70	170,00	25,848	89,00	21,50	26,453	4,45336	0,078082
992	77,00	173,00	25,728	90,00	21,50	25,400	4,31692	0,078515
993	87,90	176,50	28,216	97,00	21,50	28,790	4,51769	0,078777
994	76,00	170,00	26,298	91,00	21,50	28,273	4,58371	0,078924

995	78,00	165,00	28,650	95,00	21,50	29,155	5,22602	0,078988
996	78,30	168,00	27,742	94,00	21,50	28,269	4,92619	0,079137
997	85,60	177,00	27,323	97,00	21,50	29,409	4,48460	0,080371
998	89,40	185,00	26,121	99,00	21,50	27,065	4,10146	0,082677
999	73,80	169,00	25,839	94,00	21,50	27,327	4,85209	0,082730
1000	87,00	184,50	25,558	99,00	21,50	25,936	4,13099	0,084001
1001	72,30	169,00	25,314	99,00	21,50	25,475	5,18229	0,088331
1002	74,60	168,00	26,431	102,00	21,50	27,106	5,46107	0,088688
1003	77,50	168,00	27,459	90,00	21,60	29,237	4,65905	0,076290
1004	81,40	178,00	25,691	89,00	21,60	25,860	3,94415	0,076617
1005	78,90	170,00	27,301	91,00	21,60	27,793	4,58371	0,076978
1006	72,00	164,50	26,607	89,00	21,60	27,577	4,84786	0,077859
1007	72,60	164,50	26,829	90,00	21,60	27,513	4,91754	0,078299
1008	78,50	172,00	26,535	93,00	21,60	27,723	4,57392	0,079710
1009	95,80	191,00	26,260	98,00	21,60	26,265	3,71356	0,080262
1010	88,10	180,00	27,191	98,00	21,60	27,647	4,35001	0,080780
1011	79,60	177,00	25,408	93,00	21,60	25,081	4,24423	0,080882
1012	78,00	176,00	25,181	93,00	21,60	25,159	4,30790	0,081598
1013	89,40	189,00	25,027	97,00	21,60	26,153	3,76836	0,082464
1014	77,30	174,00	25,532	95,00	21,60	26,935	4,56301	0,083061
1015	76,90	174,00	25,400	96,00	21,60	25,722	4,62523	0,084226
1016	85,00	176,00	27,441	90,00	21,70	28,316	4,12564	0,074569
1017	84,20	173,00	28,133	94,00	21,70	29,639	4,56860	0,077261
1018	85,30	179,00	26,622	93,00	21,70	27,597	4,12010	0,077964
1019	78,00	167,00	27,968	93,00	21,70	29,108	4,93401	0,078106
1020	88,60	183,00	26,456	94,00	21,70	26,535	3,94020	0,078261
1021	70,80	168,00	25,085	88,00	21,70	25,294	4,52555	0,079229
1022	81,80	177,00	26,110	93,00	21,70	25,934	4,24423	0,079425
1023	86,00	180,00	26,543	96,00	21,70	27,374	4,23381	0,080414
1024	79,50	168,00	28,168	97,00	21,70	30,213	5,12667	0,080838
1025	70,40	167,00	25,243	90,00	21,70	26,037	4,73123	0,080933
1026	73,60	171,00	25,170	91,00	21,70	26,338	4,51454	0,081025
1027	76,50	172,50	25,709	96,00	21,70	26,268	4,72960	0,083911
1028	79,30	174,00	26,192	98,00	21,70	27,774	4,74971	0,084237
1029	85,50	181,00	26,098	91,00	21,80	26,538	3,88530	0,076877
1030	81,80	173,50	27,174	92,00	21,80	28,426	4,40942	0,077274
1031	84,00	175,00	27,429	94,00	21,80	27,514	4,43416	0,078128
1032	72,70	168,50	25,606	90,00	21,80	26,258	4,62344	0,079809
1033	75,50	169,50	26,279	92,00	21,80	27,026	4,68434	0,079946
1034	79,00	174,50	25,944	94,00	21,80	27,836	4,46733	0,081197
1035	78,50	172,00	26,535	95,00	21,80	28,084	4,70129	0,081424
1036	76,00	171,00	25,991	97,00	21,80	26,644	4,90123	0,084539
1037	80,00	172,00	27,042	90,00	21,90	28,025	4,38294	0,076171
1038	85,50	179,00	26,685	92,00	21,90	26,490	4,06138	0,077005
1039	79,50	174,00	26,258	90,00	21,90	26,140	4,25205	0,077230
1040	87,90	175,00	28,702	96,00	21,90	31,161	4,55716	0,077412
1041	90,10	184,00	26,613	94,00	21,90	26,987	3,88299	0,077743
1042	79,50	170,00	27,509	93,00	21,90	28,964	4,71411	0,078273
1043	76,90	173,00	25,694	90,00	21,90	27,142	4,31692	0,078583
1044	95,00	186,00	27,460	98,00	21,90	28,902	3,98873	0,078947

1045	86,80	182,00	26,205	94,00	21,90	26,435	3,99837	0,078978
1046	94,10	178,00	29,700	102,00	21,90	30,809	4,71655	0,079718
1047	89,30	181,00	27,258	99,00	21,90	28,533	4,34472	0,081246
1048	86,30	177,00	27,546	99,00	21,90	28,454	4,60485	0,081584
1049	88,10	174,00	29,099	102,00	21,90	29,746	4,99881	0,081735
1050	76,00	168,50	26,768	98,00	21,90	27,786	5,15470	0,084369
1051	83,00	176,00	26,795	90,00	22,00	26,996	4,12564	0,075762
1052	87,00	176,50	27,927	93,00	22,00	29,814	4,27593	0,076049
1053	89,40	177,00	28,536	96,00	22,00	29,624	4,42449	0,077272
1054	78,30	170,00	27,093	92,00	22,00	28,323	4,64890	0,078221
1055	78,00	174,50	25,616	90,00	22,00	25,372	4,22003	0,078405
1056	82,00	173,00	27,398	94,00	22,00	27,472	4,56860	0,078636
1057	71,50	165,00	26,263	90,00	22,00	27,004	4,87959	0,079300
1058	73,50	169,00	25,734	90,10	22,00	26,939	4,59476	0,079513
1059	77,50	162,00	29,531	99,00	22,00	32,027	5,76022	0,081414
1060	84,00	180,00	25,926	96,00	22,00	25,678	4,23381	0,081686
1061	80,20	170,00	27,751	100,00	22,00	28,720	5,17086	0,083674
1062	72,30	170,00	25,017	94,00	22,00	25,201	4,77932	0,084284
1063	78,10	170,00	27,024	99,00	22,00	29,161	5,10557	0,084316
1064	67,80	161,00	26,156	95,00	22,00	27,975	5,55746	0,084969
1065	91,00	183,00	27,173	104,00	22,00	27,164	4,50217	0,085058
1066	86,00	178,00	27,143	98,00	22,05	27,812	4,47871	0,081329
1067	75,30	168,00	26,679	90,00	22,10	28,147	4,65905	0,077768
1068	82,00	173,00	27,398	93,00	22,10	28,261	4,50566	0,077800
1069	92,30	184,00	27,263	96,00	22,10	27,401	3,99408	0,078130
1070	79,10	169,00	27,695	94,00	22,10	27,986	4,85209	0,078992
1071	82,00	180,00	25,309	92,00	22,10	26,386	4,00154	0,079550
1072	70,90	165,00	26,042	90,00	22,10	27,459	4,87959	0,079747
1073	75,20	170,50	25,868	92,00	22,10	25,976	4,61378	0,080553
1074	75,10	173,00	25,093	91,00	22,10	24,904	4,37982	0,080721
1075	89,60	183,50	26,609	98,00	22,10	28,058	4,13492	0,081168
1076	82,40	176,00	26,601	96,00	22,10	26,852	4,49026	0,081205
1077	77,50	170,50	26,660	96,00	22,10	27,526	4,87310	0,082384
1078	81,50	177,00	26,014	97,00	22,10	26,300	4,48460	0,083045
1079	82,00	180,00	25,309	102,00	22,10	26,386	4,58250	0,088197
1080	74,50	166,00	27,036	88,00	22,20	29,175	4,66794	0,075823
1081	81,00	176,00	26,149	89,00	22,20	27,225	4,06490	0,076148
1082	81,10	172,00	27,413	91,00	22,20	27,831	4,44659	0,076319
1083	75,80	171,00	25,923	88,00	22,20	26,427	4,32136	0,076830
1084	88,40	178,00	27,901	95,00	22,20	29,022	4,30044	0,077406
1085	87,00	183,00	25,979	92,00	22,20	26,502	3,82792	0,077533
1086	79,50	170,50	27,348	92,00	22,20	29,326	4,61378	0,077621
1087	91,30	179,00	28,495	100,00	22,20	30,918	4,53137	0,080118
1088	86,90	183,50	25,808	95,00	22,20	27,286	3,96733	0,080305
1089	90,00	187,00	25,737	97,00	22,20	25,668	3,87814	0,081373
1090	78,50	172,00	26,535	95,00	22,20	27,843	4,70129	0,081424
1091	76,00	174,00	25,102	93,00	22,20	25,850	4,43859	0,082237
1092	78,20	173,00	26,129	88,00	22,30	27,074	4,19115	0,075983
1093	82,40	181,00	25,152	89,00	22,30	24,322	3,77054	0,077061
1094	94,40	187,70	26,794	95,00	22,30	27,467	3,73261	0,077439

1095	81,60	179,00	25,467	90,00	22,30	26,463	3,94398	0,077713
1096	85,00	181,00	25,945	93,00	22,30	26,332	4,00010	0,078874
1097	87,90	177,00	28,057	97,00	22,30	29,972	4,48460	0,078963
1098	79,60	173,00	26,596	93,00	22,30	28,400	4,50566	0,079356
1099	72,00	166,00	26,129	91,00	22,30	27,693	4,87314	0,080213
1100	77,90	175,00	25,437	93,00	22,30	26,032	4,37268	0,081281
1101	76,90	170,00	26,609	96,00	22,30	27,221	4,90978	0,082609
1102	69,00	166,00	25,040	96,00	22,30	26,169	5,21540	0,087055
1103	82,80	174,00	27,348	88,00	22,40	28,083	4,12774	0,073494
1104	77,40	174,00	25,565	88,00	22,40	26,977	4,12774	0,076874
1105	78,90	169,50	27,462	92,00	22,40	28,905	4,68434	0,077633
1106	73,50	170,00	25,433	88,00	22,40	26,287	4,38821	0,078043
1107	80,10	176,00	25,859	91,00	22,40	26,341	4,18638	0,078442
1108	81,00	180,00	25,000	91,00	22,40	25,714	3,94350	0,079331
1109	76,30	173,00	25,494	92,00	22,40	25,201	4,44274	0,080750
1110	92,50	185,00	27,027	99,00	22,40	28,817	4,10146	0,080820
1111	71,00	168,00	25,156	90,00	22,40	26,851	4,65905	0,080878
1112	89,50	185,00	26,150	97,00	22,40	27,597	3,99153	0,080947
1113	101,50	189,00	28,415	104,00	22,40	29,573	4,13688	0,081241
1114	78,00	172,00	26,366	98,00	22,40	28,235	4,89244	0,084354
1115	78,00	172,50	26,213	99,00	22,40	27,677	4,91965	0,085421
1116	78,90	168,50	27,789	93,00	22,50	29,973	4,82257	0,078090
1117	83,00	181,00	25,335	91,00	22,50	25,896	3,88530	0,078413
1118	83,40	173,00	27,866	95,00	22,50	28,760	4,63155	0,078581
1119	75,50	173,00	25,226	89,00	22,50	25,090	4,25403	0,078668
1120	78,30	170,00	27,093	93,00	22,50	27,285	4,71411	0,079071
1121	72,00	168,00	25,510	89,00	22,50	26,907	4,59229	0,079237
1122	78,60	177,00	25,089	91,00	22,50	25,699	4,12410	0,079812
1123	82,70	181,00	25,243	94,00	22,50	25,378	4,05752	0,081194
1124	81,80	176,00	26,408	96,00	22,50	28,044	4,49026	0,081601
1125	70,60	167,50	25,164	91,00	22,50	25,401	4,76215	0,081881
1126	73,00	168,00	25,865	93,00	22,50	27,233	4,85938	0,082040
1127	75,00	171,00	25,649	94,00	22,50	26,059	4,70783	0,082651
1128	88,00	186,00	25,436	98,00	22,50	26,943	3,98873	0,083080
1129	67,20	161,00	25,925	95,00	22,50	26,305	5,55746	0,085474
1130	75,00	171,00	25,649	99,00	22,50	27,341	5,03021	0,087048
1131	79,50	167,00	28,506	88,00	22,60	29,896	4,59610	0,072974
1132	84,20	181,00	25,701	88,00	22,60	25,747	3,71317	0,075106
1133	98,80	193,00	26,524	94,00	22,60	26,988	3,40779	0,076077
1134	78,70	173,00	26,296	89,00	22,60	26,681	4,25403	0,076521
1135	79,80	171,00	27,290	91,00	22,60	27,325	4,51454	0,076772
1136	81,00	174,00	26,754	91,00	22,60	27,059	4,31422	0,077122
1137	74,20	169,00	25,979	90,00	22,60	26,128	4,58816	0,078925
1138	81,00	178,00	25,565	92,00	22,60	27,364	4,12225	0,079460
1139	89,30	178,00	28,185	99,00	22,60	29,809	4,53816	0,080122
1140	87,50	174,00	28,901	100,00	22,60	29,586	4,87424	0,080498
1141	72,50	166,00	26,310	93,00	22,60	28,288	5,01001	0,081598
1142	76,50	169,00	26,785	96,00	22,60	28,398	4,98414	0,082490
1143	74,50	170,00	25,779	94,00	22,60	25,599	4,77932	0,082616
1144	80,10	172,00	27,075	98,00	22,60	28,992	4,89244	0,082873

1145	84,60	183,00	25,262	97,00	22,60	24,744	4,10870	0,083285
1146	72,40	167,00	25,960	95,00	22,60	26,227	5,06927	0,083848
1147	79,50	172,00	26,873	89,00	22,70	26,867	4,31930	0,075640
1148	81,30	176,00	26,246	89,00	22,70	26,614	4,06490	0,075961
1149	78,80	169,50	27,428	91,00	22,70	28,861	4,61876	0,076854
1150	91,00	177,50	28,883	97,00	22,70	30,167	4,45180	0,077341
1151	82,10	175,00	26,808	92,00	22,70	27,132	4,31121	0,077641
1152	80,10	170,00	27,716	93,00	22,70	29,115	4,71411	0,077882
1153	81,00	171,00	27,701	94,00	22,70	29,976	4,70783	0,078518
1154	79,00	173,00	26,396	93,00	22,70	27,423	4,50566	0,079757
1155	83,50	181,50	25,347	93,00	22,70	25,258	3,97073	0,080000
1156	63,30	154,00	26,691	89,00	22,70	26,855	5,72327	0,080301
1157	69,50	166,00	25,221	89,00	22,70	26,272	4,73633	0,080320
1158	83,50	179,00	26,060	95,00	22,70	25,492	4,23756	0,080781
1159	73,90	170,00	25,571	92,00	22,70	26,856	4,64890	0,081295
1160	76,70	173,00	25,627	93,00	22,70	26,030	4,50566	0,081344
1161	79,00	171,00	27,017	96,00	22,70	29,152	4,83675	0,081536
1162	83,70	173,50	27,805	99,00	22,70	28,134	4,84755	0,081891
1163	81,20	180,00	25,062	95,00	22,70	25,930	4,17573	0,082682
1164	86,80	174,00	28,670	103,00	22,70	30,510	5,06110	0,083358
1165	79,60	170,00	27,543	100,00	22,70	27,781	5,17086	0,084094
1166	81,50	172,00	27,549	102,00	22,70	29,126	5,14746	0,085265
1167	87,70	178,00	27,680	93,00	22,80	29,289	4,18163	0,076179
1168	80,90	175,00	26,416	90,00	22,80	26,480	4,18830	0,076702
1169	73,80	169,00	25,839	88,00	22,80	27,706	4,45626	0,077449
1170	73,50	170,00	25,433	88,00	22,80	26,678	4,38821	0,078043
1171	97,60	181,00	29,792	102,00	22,80	31,393	4,51716	0,078892
1172	79,70	178,00	25,155	91,00	22,80	26,716	4,06287	0,079449
1173	81,60	176,00	26,343	94,00	22,80	27,354	4,36867	0,080032
1174	64,80	159,00	25,632	88,00	22,80	26,678	5,20986	0,080278
1175	95,50	189,50	26,594	99,00	22,80	27,680	3,84616	0,080719
1176	76,60	174,00	25,301	92,00	22,80	26,244	4,37640	0,080927
1177	76,50	168,00	27,105	95,00	22,80	27,992	4,99300	0,081228
1178	88,40	182,00	26,688	98,00	22,80	27,563	4,22556	0,081342
1179	74,00	169,50	25,757	93,00	22,80	27,603	4,74994	0,081904
1180	80,70	175,00	26,351	96,00	22,80	26,755	4,55716	0,081951
1181	85,70	184,00	25,313	96,00	22,80	25,342	3,99408	0,082092
1182	82,00	180,00	25,309	95,00	22,80	24,942	4,17573	0,082144
1183	78,00	171,00	26,675	96,00	22,80	26,595	4,83675	0,082232
1184	69,00	166,00	25,040	91,00	22,80	26,571	4,87314	0,082521
1185	79,80	173,00	26,663	101,00	22,80	27,769	5,00946	0,086038
1186	72,60	165,00	26,667	101,00	22,80	28,131	5,64218	0,088091
1187	74,30	169,00	26,014	88,00	22,90	27,923	4,45626	0,077101
1188	81,00	178,00	25,565	90,00	22,90	24,914	4,00350	0,077732
1189	88,00	182,00	26,567	94,00	22,90	27,525	3,99837	0,078259
1190	74,00	170,00	25,606	89,00	22,90	27,029	4,45336	0,078574
1191	87,00	178,00	27,459	97,00	22,90	29,237	4,41928	0,079881
1192	73,50	169,00	25,734	91,00	22,90	27,448	4,65413	0,080307
1193	77,90	171,00	26,641	94,00	22,90	27,144	4,70783	0,080587
1194	88,00	181,00	26,861	100,00	22,90	28,377	4,40219	0,082872

1195	86,90	175,50	28,214	102,00	22,90	30,587	4,89067	0,083078
1196	71,90	169,00	25,174	93,00	22,90	26,211	4,78609	0,083285
1197	88,20	185,00	25,771	99,00	22,90	26,223	4,10146	0,083426
1198	75,60	170,00	26,159	96,00	22,90	26,991	4,90978	0,083553
1199	94,40	180,00	29,136	107,00	22,90	30,189	4,87334	0,084229
1200	74,40	161,00	28,703	101,00	22,90	31,264	5,99497	0,084910
1201	76,00	170,00	26,298	98,00	22,90	27,050	5,04030	0,084995
1202	78,70	174,00	25,994	99,00	22,90	27,773	4,81197	0,085528
1203	82,40	174,00	27,216	104,00	22,90	28,480	5,12342	0,087138
1204	90,00	183,00	26,874	93,00	23,00	27,807	3,88406	0,076624
1205	73,00	164,50	26,977	89,00	23,00	28,871	4,84786	0,077146
1206	75,00	168,00	26,573	90,00	23,00	26,694	4,65905	0,077976
1207	82,10	177,00	26,206	92,00	23,00	26,806	4,18416	0,078380
1208	76,10	173,00	25,427	90,00	23,00	26,280	4,31692	0,079133
1209	84,60	176,50	27,157	95,00	23,00	27,601	4,39679	0,079146
1210	90,30	183,00	26,964	97,00	23,00	28,506	4,10870	0,079742
1211	76,00	173,00	25,393	92,00	23,00	26,236	4,44274	0,080962
1212	81,20	174,00	26,820	96,00	23,00	28,207	4,62523	0,081225
1213	96,50	193,00	25,907	99,00	23,00	27,538	3,65993	0,081392
1214	74,70	171,00	25,546	93,00	23,00	26,050	4,64339	0,081991
1215	82,00	172,00	27,718	99,00	23,00	29,117	4,95618	0,082420
1216	71,40	166,00	25,911	93,00	23,00	26,663	5,01001	0,082434
1217	76,30	167,00	27,358	97,00	23,00	28,548	5,20457	0,082671
1218	97,10	186,00	28,067	106,00	23,00	29,234	4,42390	0,084156
1219	86,50	178,00	27,301	102,00	23,00	27,453	4,71655	0,084322
1220	77,40	174,00	25,565	99,00	23,00	27,364	4,81197	0,086483
1221	82,50	181,00	25,182	101,00	23,00	26,354	4,45967	0,087381
1222	87,50	176,00	28,248	96,00	23,10	28,620	4,49026	0,078018
1223	86,50	182,00	26,114	93,00	23,10	26,559	3,94160	0,078319
1224	70,40	166,50	25,395	90,00	23,10	25,846	4,76781	0,080731
1225	78,30	167,00	28,076	97,00	23,10	29,139	5,20457	0,081257
1226	86,70	179,00	27,059	98,00	23,10	27,701	4,41381	0,081269
1227	80,50	176,00	25,988	95,00	23,10	26,139	4,42946	0,081618
1228	74,00	170,00	25,606	94,00	23,10	25,872	4,77932	0,082988
1229	86,00	178,00	27,143	101,00	23,10	29,305	4,65708	0,083818
1230	79,10	176,50	25,391	97,00	23,10	27,147	4,51769	0,084516
1231	87,00	180,00	26,852	103,00	23,10	28,013	4,64065	0,085615
1232	85,30	181,00	26,037	102,00	23,10	27,577	4,51716	0,086304
1233	83,00	169,00	29,061	92,00	23,20	30,588	4,72010	0,074870
1234	78,00	170,00	26,990	88,00	23,20	28,074	4,38821	0,075011
1235	86,00	180,00	26,543	93,00	23,20	28,575	4,05959	0,077901
1236	75,20	173,00	25,126	88,00	23,20	25,749	4,19115	0,077991
1237	78,40	175,00	25,600	92,00	23,20	27,151	4,31121	0,080065
1238	84,80	183,00	25,322	96,00	23,20	25,354	4,05253	0,082297
1239	70,00	166,00	25,403	92,00	23,20	25,074	4,94157	0,082632
1240	69,70	166,50	25,142	92,00	23,20	25,239	4,90382	0,083076
1241	74,50	168,00	26,396	99,00	23,20	27,180	5,26039	0,086157
1242	78,40	168,00	27,778	88,00	23,30	28,426	4,52555	0,074022
1243	91,00	182,00	27,473	93,00	23,30	28,694	3,94160	0,075715
1244	76,00	173,00	25,393	88,00	23,30	25,061	4,19115	0,077442

1245	74,80	171,00	25,581	88,00	23,30	25,838	4,32136	0,077514
1246	94,00	188,00	26,596	98,00	23,30	27,443	3,87600	0,080218
1247	73,50	170,00	25,433	92,00	23,30	26,157	4,64890	0,081590
1248	84,70	173,00	28,300	100,00	23,30	29,637	4,94644	0,081868
1249	89,80	184,00	26,524	99,00	23,30	27,108	4,16077	0,082060
1250	96,60	185,00	28,225	106,00	23,30	29,119	4,48645	0,084068
1251	80,00	177,00	25,535	103,00	23,30	25,906	4,84547	0,089280
1252	81,90	171,00	28,009	91,00	23,40	28,299	4,51454	0,075454
1253	83,60	178,00	26,386	90,00	23,40	28,260	4,00350	0,076112
1254	85,90	182,00	25,933	90,00	23,40	25,562	3,77132	0,076145
1255	91,50	175,50	29,708	97,00	23,40	30,631	4,58472	0,076335
1256	80,70	176,50	25,905	90,00	23,40	26,404	4,09471	0,077377
1257	85,90	179,00	26,809	93,00	23,40	26,898	4,12010	0,077601
1258	86,90	172,00	29,374	97,00	23,40	30,685	4,82871	0,077690
1259	84,00	177,00	26,812	94,00	23,40	27,138	4,30431	0,078871
1260	70,50	167,00	25,279	88,00	23,40	25,163	4,59610	0,079059
1261	82,40	181,00	25,152	92,00	23,40	26,314	3,94270	0,079659
1262	85,40	178,00	26,954	96,00	23,40	28,843	4,35985	0,080042
1263	71,80	163,50	26,859	92,00	23,40	28,843	5,13563	0,080224
1264	80,50	171,00	27,530	96,00	23,40	29,660	4,83675	0,080520
1265	79,90	170,00	27,647	96,00	23,40	29,027	4,90978	0,080528
1266	83,40	170,00	28,858	99,00	23,40	30,034	5,10557	0,080705
1267	70,10	167,00	25,135	91,00	23,40	26,692	4,79881	0,082065
1268	72,00	168,00	25,510	93,00	23,40	27,037	4,85938	0,082798
1269	71,80	164,00	26,695	95,00	23,40	28,405	5,30658	0,083051
1270	80,50	176,00	25,988	97,00	23,40	26,515	4,55106	0,083336
1271	77,60	175,00	25,339	98,00	23,40	25,770	4,68020	0,085872
1272	93,60	184,00	27,647	96,00	23,50	27,921	3,99408	0,077405
1273	101,50	184,00	29,980	102,00	23,50	31,255	4,32754	0,077918
1274	80,50	172,00	27,211	95,00	23,50	28,016	4,70129	0,080069
1275	91,10	185,00	26,618	100,00	23,50	27,950	4,15643	0,082470
1276	75,60	172,00	25,554	94,00	23,50	26,448	4,63760	0,082614
1277	85,00	174,00	28,075	101,00	23,50	28,710	4,93652	0,082890
1278	74,00	164,00	27,513	97,00	23,50	29,081	5,44698	0,083110
1279	71,50	162,00	27,244	96,00	23,50	28,630	5,54423	0,083303
1280	86,00	184,00	25,402	98,00	23,50	25,725	4,10520	0,083607
1281	80,90	176,00	26,117	104,00	23,50	26,440	4,97699	0,089056
1282	83,00	174,00	27,414	90,00	23,60	28,170	4,25205	0,075044
1283	93,50	182,00	28,227	96,00	23,60	29,016	4,11195	0,076758
1284	100,00	183,00	29,861	100,00	23,60	30,919	4,27728	0,076803
1285	87,20	184,00	25,756	91,00	23,60	26,458	3,71643	0,076921
1286	77,50	170,00	26,817	90,00	23,60	28,673	4,51853	0,077046
1287	84,50	179,00	26,372	92,00	23,60	27,392	4,06138	0,077612
1288	92,90	189,00	26,007	94,00	23,60	26,540	3,61054	0,077894
1289	78,20	173,00	26,129	91,00	23,60	28,064	4,37982	0,078573
1290	86,20	178,00	27,206	95,00	23,60	27,895	4,30044	0,078717
1291	74,20	165,00	27,254	93,00	23,60	27,617	5,08741	0,079944
1292	66,70	162,00	25,415	88,00	23,60	27,177	4,96890	0,079982
1293	89,60	186,00	25,899	96,00	23,60	26,773	3,88003	0,080413
1294	80,30	175,00	26,220	94,00	23,60	26,825	4,43416	0,080510

1295	83,40	178,00	26,322	96,00	23,60	27,937	4,35985	0,081316
1296	75,20	164,00	27,960	96,00	23,60	30,069	5,37677	0,081376
1297	80,00	174,00	26,424	96,00	23,60	26,853	4,62523	0,082036
1298	86,00	178,00	27,143	99,00	23,60	28,042	4,53816	0,082159
1299	84,60	175,00	27,624	100,00	23,60	29,663	4,80328	0,082721
1300	71,00	168,50	25,007	92,00	23,60	25,589	4,75619	0,082880
1301	71,80	160,00	28,047	97,00	23,60	30,174	5,79192	0,083072
1302	82,20	174,00	27,150	99,00	23,60	28,855	4,81197	0,083083
1303	78,80	177,00	25,152	95,00	23,60	26,448	4,36440	0,083180
1304	79,40	172,00	26,839	98,00	23,60	28,231	4,89244	0,083359
1305	78,00	174,00	25,763	96,00	23,60	27,484	4,62523	0,083432
1306	80,90	172,50	27,188	100,00	23,60	27,871	4,98303	0,084209
1307	87,40	181,00	26,678	102,00	23,60	27,075	4,51716	0,084916
1308	74,50	172,00	25,183	99,00	23,60	26,752	4,95618	0,087863
1309	93,00	178,00	29,352	94,00	23,70	31,046	4,24103	0,074044
1310	76,00	168,00	26,927	88,00	23,70	28,110	4,52555	0,075573
1311	75,80	167,00	27,179	90,00	23,70	29,120	4,73123	0,077042
1312	79,90	175,50	25,941	90,00	23,70	26,829	4,15683	0,077525
1313	89,00	175,50	28,896	97,00	23,70	30,685	4,58472	0,077758
1314	78,70	175,00	25,698	90,00	23,70	27,275	4,18830	0,078125
1315	81,50	179,00	25,436	92,00	23,70	25,511	4,06138	0,079505
1316	71,00	162,00	27,054	92,00	23,70	27,694	5,25645	0,080207
1317	89,00	175,00	29,061	101,00	23,70	31,382	4,86484	0,080772
1318	89,90	175,00	29,355	103,00	23,70	31,049	4,98798	0,081821
1319	77,40	168,00	27,423	97,00	23,70	28,856	5,12667	0,082294
1320	80,80	173,00	26,997	102,00	23,70	28,083	5,07248	0,086172
1321	88,10	182,00	26,597	105,00	23,70	27,205	4,62350	0,087350
1322	73,20	169,00	25,629	99,00	23,70	27,188	5,18229	0,087606
1323	93,00	183,00	27,770	93,00	23,80	28,526	3,88406	0,074967
1324	89,60	178,00	28,279	94,00	23,80	29,821	4,24103	0,075905
1325	82,70	174,50	27,159	91,00	23,80	28,751	4,28184	0,076243
1326	87,50	178,00	27,616	95,00	23,80	28,434	4,30044	0,077935
1327	88,00	176,00	28,409	97,00	23,80	29,565	4,55106	0,078531
1328	83,00	180,00	25,617	92,00	23,80	25,631	4,00154	0,078910
1329	83,10	174,00	27,447	95,00	23,80	28,887	4,56301	0,079149
1330	75,70	168,50	26,662	92,00	23,80	27,887	4,75619	0,079413
1331	79,80	171,50	27,132	94,00	23,80	27,222	4,67256	0,079496
1332	77,10	167,00	27,645	94,00	23,80	29,246	5,00163	0,079559
1333	79,40	169,00	27,800	96,00	23,80	29,986	4,98414	0,080469
1334	96,60	184,50	28,378	102,00	23,80	29,526	4,29685	0,080713
1335	77,70	174,50	25,517	93,00	23,80	26,787	4,40549	0,081227
1336	72,30	169,00	25,314	92,00	23,80	25,081	4,72010	0,082086
1337	77,40	170,00	26,782	96,00	23,80	28,159	4,90978	0,082253
1338	75,90	170,50	26,109	98,00	23,80	26,677	5,00284	0,085278
1339	75,80	163,00	28,529	106,00	23,80	29,512	6,17120	0,088923
1340	85,40	173,00	28,534	88,00	23,90	29,208	4,19115	0,071650
1341	91,50	179,00	28,557	94,00	23,90	30,166	4,17882	0,075201
1342	88,10	181,00	26,892	91,00	23,90	27,479	3,88530	0,075357
1343	84,20	172,00	28,461	93,00	23,90	29,840	4,57392	0,076070
1344	75,50	168,00	26,750	89,00	23,90	27,763	4,59229	0,076769

1345	81,00	177,00	25,855	90,00	23,90	26,462	4,06405	0,077368
1346	83,20	172,00	28,123	94,00	23,90	28,773	4,63760	0,077503
1347	83,40	177,50	26,471	92,00	23,90	28,365	4,15307	0,077746
1348	74,50	167,00	26,713	90,00	23,90	27,597	4,73123	0,077936
1349	80,00	173,00	26,730	92,00	23,90	28,211	4,44274	0,078241
1350	86,80	176,00	28,022	96,00	23,90	29,392	4,49026	0,078437
1351	90,50	180,50	27,778	97,00	23,90	29,630	4,26073	0,078717
1352	94,80	178,00	29,920	102,00	23,90	31,088	4,71655	0,079325
1353	69,60	166,00	25,258	89,00	23,90	26,452	4,73633	0,080243
1354	81,00	175,00	26,449	96,00	23,90	27,855	4,55716	0,081748
1355	77,60	166,00	28,161	98,00	23,90	30,311	5,35240	0,082175
1356	89,30	186,00	25,812	98,00	23,90	26,406	3,98873	0,082272
1357	78,30	173,00	26,162	96,00	23,90	26,871	4,69450	0,082820
1358	84,00	181,50	25,499	97,00	23,90	25,986	4,19916	0,083109
1359	74,50	169,00	26,085	96,00	23,90	28,010	4,98414	0,083960
1360	76,30	169,00	26,715	98,00	23,90	28,073	5,11622	0,084356
1361	81,20	170,00	28,097	102,00	23,90	29,486	5,30146	0,084645
1362	84,10	178,00	26,543	102,00	23,90	28,455	4,71655	0,085918
1363	93,60	185,00	27,348	107,00	23,90	28,988	4,54148	0,086665
1364	75,90	172,00	25,656	99,00	23,90	26,581	4,95618	0,086779
1365	90,60	179,00	28,276	93,00	24,00	28,974	4,12010	0,074893
1366	87,00	176,00	28,086	92,00	24,00	30,115	4,24714	0,075053
1367	82,30	173,00	27,498	90,00	24,00	28,056	4,31692	0,075107
1368	95,00	187,00	27,167	95,00	24,00	27,500	3,77062	0,076873
1369	84,00	170,00	29,066	96,00	24,00	30,991	4,90978	0,077886
1370	69,70	164,00	25,915	89,00	24,00	26,668	4,88573	0,079361
1371	78,20	173,00	26,129	92,00	24,00	27,693	4,44274	0,079437
1372	76,40	169,00	26,750	93,00	24,00	27,881	4,78609	0,079982
1373	90,40	182,00	27,291	98,00	24,00	28,121	4,22556	0,080138
1374	71,90	167,50	25,627	91,00	24,00	27,185	4,76215	0,080891
1375	78,00	170,00	26,990	95,00	24,00	27,841	4,84455	0,080978
1376	84,00	176,00	27,118	97,00	24,00	28,124	4,55106	0,081005
1377	95,00	186,00	27,460	101,00	24,00	28,902	4,15186	0,081364
1378	82,80	168,00	29,337	101,00	24,00	31,899	5,39416	0,081920
1379	81,30	172,00	27,481	99,00	24,00	28,257	4,95618	0,082892
1380	75,70	173,00	25,293	94,00	24,00	26,498	4,56860	0,082941
1381	73,00	163,00	27,476	98,00	24,00	29,258	5,60190	0,084301
1382	86,80	181,50	26,349	101,00	24,00	27,484	4,42773	0,084665
1383	63,80	159,00	25,236	92,00	24,00	26,160	5,50857	0,084802
1384	99,40	185,00	29,043	111,00	24,00	30,666	4,76169	0,086372
1385	82,10	170,50	28,242	90,00	24,10	29,458	4,48418	0,074322
1386	83,10	171,00	28,419	93,00	24,10	29,891	4,64339	0,076368
1387	76,70	168,50	27,014	90,00	24,10	27,990	4,62344	0,077010
1388	70,90	164,00	26,361	88,00	24,10	27,986	4,81564	0,077581
1389	80,00	172,00	27,042	92,00	24,10	28,604	4,51025	0,077864
1390	80,80	176,00	26,085	91,00	24,10	27,886	4,18638	0,077988
1391	89,80	176,00	28,990	98,00	24,10	30,700	4,61188	0,078277
1392	82,80	178,00	26,133	93,00	24,10	27,451	4,18163	0,079155
1393	70,10	167,00	25,135	88,00	24,10	25,362	4,59610	0,079360
1394	79,30	171,00	27,119	94,00	24,10	27,321	4,70783	0,079636

1395	88,00	179,00	27,465	97,00	24,10	28,236	4,35505	0,079645
1396	76,20	168,00	26,998	93,00	24,10	28,781	4,85938	0,079727
1397	83,20	178,00	26,259	94,00	24,10	27,000	4,24103	0,079750
1398	85,00	175,00	27,755	97,00	24,10	29,822	4,61867	0,079988
1399	88,00	178,00	27,774	98,00	24,10	30,064	4,47871	0,080092
1400	83,20	172,50	27,961	97,00	24,10	30,178	4,79294	0,080170
1401	78,80	172,00	26,636	94,00	24,10	27,973	4,63760	0,080362
1402	99,00	184,00	29,241	104,00	24,10	30,714	4,43877	0,080778
1403	87,90	181,00	26,831	98,00	24,10	27,398	4,28726	0,081277
1404	90,00	177,00	28,727	102,00	24,10	30,275	4,78530	0,081737
1405	88,00	176,00	28,409	102,00	24,10	29,774	4,85524	0,082580
1406	81,40	176,00	26,278	97,00	24,10	27,025	4,55106	0,082721
1407	74,60	162,50	28,251	98,00	24,10	30,630	5,64485	0,082879
1408	87,60	184,00	25,874	100,00	24,10	25,606	4,21635	0,084271
1409	82,60	178,00	26,070	99,00	24,10	27,868	4,53816	0,084398
1410	73,90	168,00	26,183	97,00	24,10	26,159	5,12667	0,084872
1411	80,10	178,00	25,281	99,00	24,10	26,350	4,53816	0,086145
1412	97,70	188,00	27,643	96,00	24,20	27,805	3,76963	0,076584
1413	85,40	179,00	26,653	92,00	24,20	27,995	4,06138	0,077066
1414	92,50	178,50	29,031	98,00	24,20	29,757	4,44612	0,077654
1415	75,00	168,00	26,573	90,00	24,20	28,611	4,65905	0,077976
1416	88,40	178,00	27,901	97,00	24,20	30,106	4,41928	0,079035
1417	82,60	173,00	27,599	96,00	24,20	29,299	4,69450	0,079920
1418	85,00	182,00	25,661	94,00	24,20	27,484	3,99837	0,080089
1419	97,80	188,00	27,671	101,00	24,20	28,174	4,03563	0,080518
1420	76,70	171,00	26,230	93,00	24,20	26,593	4,64339	0,080559
1421	72,90	165,00	26,777	93,00	24,20	27,444	5,08741	0,080891
1422	82,40	177,00	26,302	96,00	24,20	27,055	4,42449	0,081589
1423	81,00	179,50	25,139	94,00	24,20	26,298	4,14811	0,081757
1424	88,00	179,00	27,465	100,00	24,20	29,693	4,53137	0,082109
1425	85,80	180,00	26,481	98,00	24,20	27,413	4,35001	0,082217
1426	85,00	171,00	29,069	102,00	24,20	30,995	5,22378	0,082506
1427	81,20	168,00	28,770	101,00	24,20	31,241	5,39416	0,082993
1428	77,10	173,00	25,761	96,00	24,20	26,846	4,69450	0,083677
1429	86,00	176,00	27,763	102,00	24,20	28,407	4,85524	0,083855
1430	80,80	176,00	26,085	98,00	24,20	27,389	4,61188	0,083987
1431	76,00	166,00	27,580	99,00	24,20	29,832	5,42092	0,084175
1432	78,00	175,00	25,469	97,00	24,20	27,245	4,61867	0,084705
1433	72,70	168,00	25,758	96,00	24,20	27,605	5,05983	0,084919
1434	85,90	177,00	27,419	103,00	24,20	27,500	4,84547	0,085144
1435	91,10	185,00	26,618	104,00	24,20	28,069	4,37641	0,085769
1436	86,00	184,00	25,402	101,00	24,20	26,116	4,27195	0,086166
1437	82,70	177,50	26,249	88,00	24,30	26,863	3,91426	0,074784
1438	76,90	168,00	27,246	88,00	24,30	28,632	4,52555	0,074982
1439	86,00	176,00	27,763	92,00	24,30	28,736	4,24714	0,075634
1440	78,70	177,00	25,120	89,00	24,30	25,342	4,00401	0,077992
1441	79,30	176,00	25,600	90,00	24,30	26,251	4,12564	0,078101
1442	75,00	173,00	25,059	88,00	24,30	25,526	4,19115	0,078129
1443	81,00	168,50	28,529	95,00	24,30	30,441	4,95539	0,078385
1444	84,00	174,00	27,745	95,00	24,30	27,943	4,56301	0,078583

1445	86,30	176,00	27,860	96,00	24,30	28,535	4,49026	0,078739
1446	88,70	176,00	28,635	98,00	24,30	30,673	4,61188	0,078923
1447	71,00	164,00	26,398	90,00	24,30	28,154	4,95584	0,079270
1448	79,00	174,00	26,093	92,00	24,30	27,276	4,37640	0,079279
1449	81,40	170,00	28,166	96,00	24,30	29,361	4,90978	0,079536
1450	96,90	183,00	28,935	102,00	24,30	31,033	4,38970	0,080001
1451	81,70	172,00	27,616	96,00	24,30	29,764	4,76500	0,080118
1452	83,80	173,50	27,838	97,00	24,30	29,596	4,72232	0,080172
1453	76,50	170,00	26,471	94,00	24,30	27,520	4,77932	0,081170
1454	88,70	178,00	27,995	100,00	24,30	29,466	4,59761	0,081296
1455	78,00	173,00	26,062	95,00	24,30	27,982	4,63155	0,082167
1456	100,00	195,00	26,298	102,00	24,30	26,807	3,70626	0,082597
1457	85,00	182,00	25,661	97,00	24,30	25,819	4,16875	0,082645
1458	81,50	176,00	26,311	97,00	24,30	27,556	4,55106	0,082653
1459	99,50	191,00	27,274	104,00	24,30	28,782	4,02278	0,083051
1460	82,80	173,00	27,665	100,00	24,30	29,051	4,94644	0,083116
1461	73,70	170,00	25,502	94,00	24,30	26,378	4,77932	0,083213
1462	93,30	178,00	29,447	106,00	24,30	31,546	4,95455	0,083317
1463	81,70	176,00	26,375	98,00	24,30	27,882	4,61188	0,083369
1464	72,90	169,00	25,524	94,00	24,30	26,796	4,85209	0,083409
1465	82,00	177,00	26,174	98,00	24,30	27,874	4,54472	0,083559
1466	85,50	171,00	29,240	104,00	24,30	31,103	5,35288	0,083795
1467	98,80	190,00	27,368	106,00	24,30	28,448	4,18361	0,084676
1468	85,90	175,50	27,889	104,00	24,30	30,093	5,01312	0,085363
1469	73,00	167,00	26,175	98,00	24,30	27,629	5,27224	0,086022
1470	80,00	172,00	27,042	102,00	24,30	28,604	5,14746	0,086327
1471	77,90	173,00	26,028	100,00	24,30	28,065	4,94644	0,086566
1472	74,20	170,00	25,675	100,00	24,30	27,373	5,17086	0,088126
1473	77,70	163,00	29,245	109,00	24,30	31,500	6,38492	0,089943
1474	73,00	170,00	25,260	101,00	24,30	26,059	5,23615	0,089980
1475	82,30	174,00	27,183	91,00	24,40	27,751	4,31422	0,076307
1476	78,00	170,00	26,990	90,00	24,40	28,771	4,51853	0,076716
1477	90,40	175,50	29,350	98,00	24,40	30,171	4,64589	0,077746
1478	85,80	177,00	27,387	95,00	24,40	28,810	4,36440	0,078592
1479	82,80	172,00	27,988	96,00	24,40	29,565	4,76500	0,079407
1480	88,00	172,00	29,746	100,00	24,40	30,680	5,01993	0,079424
1481	80,00	177,00	25,535	92,00	24,40	27,327	4,18416	0,079745
1482	80,00	167,00	28,685	97,00	24,40	30,733	5,20457	0,080102
1483	81,60	174,00	26,952	95,00	24,40	27,442	4,56301	0,080116
1484	82,30	171,00	28,145	97,00	24,40	29,335	4,90123	0,080168
1485	76,00	171,00	25,991	92,00	24,40	27,394	4,57896	0,080182
1486	75,30	163,50	28,168	95,00	24,40	30,426	5,34743	0,080252
1487	75,80	172,00	25,622	92,00	24,40	27,435	4,51025	0,080714
1488	77,60	170,00	26,851	95,00	24,40	27,308	4,84455	0,081256
1489	89,90	177,00	28,695	102,00	24,40	31,154	4,78530	0,081797
1490	86,40	181,00	26,373	98,00	24,40	26,785	4,28726	0,082215
1491	84,70	174,00	27,976	100,00	24,40	29,334	4,87424	0,082263
1492	73,00	170,00	25,260	93,00	24,40	26,190	4,71411	0,082853
1493	80,80	170,00	27,958	102,00	24,40	29,096	5,30146	0,084925
1494	87,10	180,00	26,883	103,00	24,40	27,701	4,64065	0,085550

1495	87,30	181,50	26,501	90,00	24,50	26,355	3,79950	0,075156
1496	85,70	180,50	26,304	90,00	24,50	26,081	3,85657	0,075739
1497	75,80	167,00	27,179	90,00	24,50	27,860	4,73123	0,077042
1498	77,20	170,00	26,713	90,00	24,50	27,240	4,51853	0,077245
1499	74,70	172,00	25,250	88,00	24,50	26,310	4,25568	0,077961
1500	87,80	175,00	28,669	97,00	24,50	30,510	4,61867	0,078278
1501	76,50	164,50	28,270	94,00	24,50	29,599	5,19637	0,078975
1502	69,60	163,00	26,196	89,00	24,50	28,148	4,96253	0,079033
1503	75,80	170,00	26,228	91,00	24,50	27,327	4,58371	0,079062
1504	74,00	170,00	25,606	90,00	24,50	26,643	4,51853	0,079456
1505	85,40	176,50	27,414	96,00	24,50	28,281	4,45723	0,079479
1506	84,90	178,00	26,796	95,00	24,50	28,058	4,30044	0,079519
1507	89,00	174,00	29,396	100,00	24,50	31,678	4,87424	0,079591
1508	85,60	174,00	28,273	98,00	24,50	30,341	4,74971	0,080051
1509	92,00	182,00	27,774	100,00	24,50	28,422	4,33921	0,080823
1510	79,00	171,50	26,860	96,00	24,50	28,492	4,80072	0,081735
1511	79,20	176,50	25,424	94,00	24,50	27,057	4,33635	0,081834
1512	100,00	186,00	28,905	108,00	24,50	30,094	4,53277	0,084078
1513	72,00	169,00	25,209	94,00	24,50	25,595	4,85209	0,084103
1514	78,10	172,50	26,247	98,00	24,50	27,596	4,85629	0,084486
1515	90,70	181,00	27,685	105,00	24,50	28,635	4,68968	0,085281
1516	92,70	181,00	28,296	90,00	24,60	30,157	3,82791	0,072042
1517	78,80	171,00	26,948	90,00	24,60	28,836	4,45014	0,076569
1518	86,20	172,00	29,137	96,00	24,60	29,993	4,76500	0,077305
1519	80,40	170,00	27,820	93,00	24,60	29,464	4,71411	0,077688
1520	76,00	166,00	27,580	93,00	24,60	28,720	5,01001	0,079073
1521	89,30	177,50	28,344	98,00	24,60	30,216	4,51158	0,079127
1522	94,00	182,00	28,378	100,00	24,60	29,003	4,33921	0,079672
1523	84,20	180,50	25,844	94,00	24,60	25,563	4,08746	0,080042
1524	92,00	182,50	27,622	99,00	24,60	28,331	4,25160	0,080198
1525	86,80	184,00	25,638	95,00	24,60	26,686	3,93853	0,080549
1526	71,10	167,50	25,342	90,00	24,60	24,595	4,69497	0,080601
1527	92,60	189,50	25,787	97,00	24,60	25,991	3,74147	0,080731
1528	88,00	174,00	29,066	101,00	24,60	30,991	4,93652	0,080995
1529	75,40	166,00	27,362	95,00	24,60	28,779	5,14693	0,081202
1530	89,10	174,00	29,429	103,00	24,60	31,043	5,06110	0,081917
1531	87,00	177,00	27,770	100,00	24,60	28,744	4,66499	0,081966
1532	91,80	185,50	26,678	101,00	24,60	27,194	4,18151	0,083058
1533	77,30	173,00	25,828	96,00	24,60	26,679	4,69450	0,083533
1534	90,30	178,50	28,341	104,00	24,60	29,793	4,80091	0,083741
1535	69,70	166,00	25,294	93,00	24,60	25,184	5,01001	0,083769
1536	87,30	178,00	27,553	102,00	24,60	28,463	4,71655	0,083806
1537	80,70	174,00	26,655	99,00	24,60	27,758	4,81197	0,084109
1538	70,80	166,00	25,693	95,00	24,60	26,757	5,14693	0,084682
1539	76,20	173,00	25,460	99,00	24,60	26,324	4,88344	0,086970
1540	85,00	176,00	27,441	105,00	24,60	29,103	5,03788	0,086997
1541	82,00	181,00	25,030	101,00	24,60	25,754	4,45967	0,087736
1542	100,00	183,00	29,861	115,00	24,60	32,036	5,12134	0,088323
1543	91,00	184,00	26,879	90,00	24,70	26,993	3,66092	0,073943
1544	84,70	171,00	28,966	93,00	24,70	29,972	4,64339	0,075403

1545	94,60	178,00	29,857	100,00	24,70	30,729	4,59761	0,077879
1546	85,00	181,00	25,945	92,00	24,70	27,211	3,94270	0,078026
1547	71,20	167,00	25,530	90,00	24,70	26,157	4,73123	0,080325
1548	86,30	176,00	27,860	98,00	24,70	29,514	4,61188	0,080380
1549	93,30	183,00	27,860	100,00	24,70	29,622	4,27728	0,080437
1550	70,20	165,50	25,630	90,00	24,70	26,290	4,84199	0,080479
1551	81,50	170,50	28,036	98,00	24,70	30,375	5,00284	0,081325
1552	83,20	168,00	29,478	101,00	24,70	31,775	5,39416	0,081658
1553	74,00	168,00	26,219	94,00	24,70	27,807	4,92619	0,082173
1554	73,70	170,00	25,502	94,00	24,70	26,378	4,77932	0,083213
1555	79,20	174,00	26,159	98,00	24,70	27,855	4,74971	0,084308
1556	87,50	179,00	27,309	108,00	24,70	28,144	5,00197	0,089015
1557	77,60	171,00	26,538	88,00	24,80	28,088	4,32136	0,075638
1558	82,50	173,00	27,565	91,00	24,80	28,256	4,37982	0,075819
1559	99,40	184,50	29,201	98,00	24,80	30,271	4,07572	0,076085
1560	84,70	176,00	27,344	93,00	24,80	27,850	4,30790	0,077236
1561	92,20	178,00	29,100	98,00	24,80	31,131	4,47871	0,077641
1562	84,00	179,00	26,216	92,00	24,80	26,820	4,06138	0,077919
1563	82,80	171,00	28,316	95,00	24,80	28,922	4,77229	0,078199
1564	68,00	164,00	25,283	88,00	24,80	26,879	4,81564	0,079771
1565	85,50	170,00	29,585	101,00	24,80	32,185	5,23615	0,080981
1566	78,60	169,00	27,520	96,00	24,80	29,201	4,98414	0,081014
1567	93,70	181,00	28,601	102,00	24,80	30,221	4,51716	0,081066
1568	78,80	170,00	27,266	96,00	24,80	29,227	4,90978	0,081276
1569	83,50	176,00	26,956	97,00	24,80	28,147	4,55106	0,081328
1570	78,00	171,00	26,675	95,00	24,80	27,665	4,77229	0,081375
1571	67,40	160,50	26,164	91,00	24,80	28,109	5,30743	0,081501
1572	83,20	176,50	26,708	97,00	24,80	28,776	4,51769	0,081717
1573	85,80	175,00	28,016	100,00	24,80	29,815	4,80328	0,081948
1574	85,60	174,00	28,273	101,00	24,80	30,235	4,93652	0,082502
1575	81,00	169,00	28,360	100,00	24,80	30,341	5,24836	0,082715
1576	73,20	167,00	26,247	95,00	24,80	26,615	5,06927	0,083236
1577	78,90	173,00	26,362	97,00	24,80	27,623	4,75747	0,083258
1578	73,00	166,00	26,492	96,00	24,80	28,150	5,21540	0,083845
1579	77,00	168,00	27,282	99,00	24,80	27,881	5,26039	0,084282
1580	79,30	174,00	26,192	100,00	24,80	28,267	4,87424	0,085956
1581	71,80	162,00	27,359	101,00	24,80	29,000	5,90428	0,087398
1582	96,80	185,00	28,283	95,00	24,90	29,510	3,88165	0,075240
1583	86,40	176,00	27,893	93,00	24,90	28,360	4,30790	0,076220
1584	86,00	175,50	27,922	93,00	24,90	30,240	4,34015	0,076275
1585	90,90	179,00	28,370	96,00	24,90	29,620	4,29630	0,077139
1586	101,00	190,00	27,978	98,00	24,90	28,258	3,76685	0,077145
1587	90,00	174,00	29,727	98,00	24,90	32,254	4,74971	0,077420
1588	87,70	174,00	28,967	98,00	24,90	30,671	4,74971	0,078768
1589	85,00	174,00	28,075	98,00	24,90	30,422	4,74971	0,080427
1590	87,30	180,00	26,944	97,00	24,90	29,064	4,29191	0,080443
1591	81,00	172,00	27,380	96,00	24,90	29,252	4,76500	0,080579
1592	76,10	168,00	26,963	94,00	24,90	28,388	4,92619	0,080655
1593	101,90	185,00	29,774	107,00	24,90	31,465	4,54148	0,081892
1594	81,00	172,00	27,380	99,00	24,90	29,365	4,95618	0,083097

1595	80,70	170,00	27,924	100,00	24,90	29,809	5,17086	0,083328
1596	88,00	178,00	27,774	103,00	24,90	29,516	4,77603	0,084178
1597	92,00	182,00	27,774	105,00	24,90	30,064	4,62350	0,084864
1598	77,30	170,00	26,747	99,00	24,90	28,351	5,10557	0,084896
1599	73,20	167,00	26,247	98,00	24,90	27,720	5,27224	0,085865
1600	80,30	176,00	25,923	103,00	24,90	26,177	4,91611	0,088638
1601	72,30	169,00	25,314	102,00	24,90	25,343	5,38055	0,091008
1602	90,60	180,00	27,963	92,00	25,00	28,778	4,00154	0,074433
1603	82,40	172,00	27,853	92,00	25,00	28,525	4,51025	0,076344
1604	77,10	162,00	29,378	93,00	25,00	30,980	5,32837	0,076744
1605	88,70	174,50	29,129	97,00	25,00	30,575	4,65292	0,077563
1606	88,20	177,50	27,994	97,00	25,00	28,280	4,45180	0,078969
1607	77,10	173,00	25,761	91,00	25,00	27,354	4,37982	0,079319
1608	84,00	170,00	29,066	98,00	25,00	30,396	5,04030	0,079509
1609	80,20	168,00	28,416	96,00	25,00	30,408	5,05983	0,079539
1610	82,90	177,00	26,461	94,00	25,00	28,112	4,30431	0,079568
1611	86,00	169,50	29,934	100,00	25,00	32,584	5,20944	0,079673
1612	95,60	187,50	27,193	99,00	25,00	28,793	3,95733	0,079952
1613	80,00	169,00	28,010	96,00	25,00	29,915	4,98414	0,080066
1614	86,80	172,00	29,340	100,00	25,00	30,643	5,01993	0,080154
1615	89,00	178,00	28,090	99,00	25,00	29,157	4,53816	0,080302
1616	86,10	180,00	26,574	96,00	25,00	26,216	4,23381	0,080352
1617	80,50	175,00	26,286	94,00	25,00	26,545	4,43416	0,080376
1618	77,50	168,00	27,459	95,00	25,00	28,452	4,99300	0,080528
1619	93,00	185,00	27,173	99,00	25,00	28,310	4,10146	0,080530
1620	74,40	167,00	26,677	93,00	25,00	27,906	4,93401	0,080606
1621	87,60	176,00	28,280	100,00	25,00	30,559	4,73354	0,081207
1622	73,00	162,00	27,816	95,00	25,00	28,695	5,47226	0,081303
1623	86,00	178,00	27,143	98,00	25,00	28,961	4,47871	0,081329
1624	81,00	172,00	27,380	97,00	25,00	28,688	4,82871	0,081418
1625	81,70	174,00	26,985	98,00	25,00	28,881	4,74971	0,082579
1626	80,50	170,00	27,855	99,00	25,00	28,636	5,10557	0,082631
1627	74,00	167,00	26,534	95,00	25,00	27,482	5,06927	0,082635
1628	73,10	166,00	26,528	95,00	25,00	27,834	5,14693	0,082896
1629	90,00	184,00	26,583	101,00	25,00	27,187	4,27195	0,083594
1630	82,50	172,00	27,887	101,00	25,00	28,895	5,08369	0,083745
1631	77,60	170,00	26,851	98,00	25,00	28,599	5,04030	0,083822
1632	74,90	170,00	25,917	97,00	25,00	27,551	4,97503	0,084949
1633	79,00	170,00	27,336	102,00	25,00	28,858	5,30146	0,086210
1634	85,20	175,00	27,820	107,00	25,00	29,246	5,23439	0,088096
1635	80,00	176,00	25,826	104,00	25,00	27,057	4,97699	0,089722
1636	78,70	170,00	27,232	106,00	25,00	29,298	5,56282	0,089818
1637	80,80	172,50	27,154	90,00	25,10	28,859	4,34979	0,075851
1638	84,30	172,00	28,495	94,00	25,10	29,675	4,63760	0,076827
1639	90,00	182,00	27,171	94,00	25,10	27,505	3,99837	0,077095
1640	76,20	173,00	25,460	89,00	25,10	25,284	4,25403	0,078185
1641	103,30	189,00	28,919	102,00	25,10	29,810	4,03155	0,078750
1642	86,70	175,00	28,310	97,00	25,10	30,700	4,61867	0,078939
1643	87,90	177,00	28,057	98,00	25,10	28,579	4,54472	0,079777
1644	90,00	174,00	29,727	101,00	25,10	31,125	4,93652	0,079790

1645	93,10	178,00	29,384	102,00	25,10	31,374	4,71655	0,080288
1646	87,00	180,00	26,852	97,00	25,10	27,543	4,29191	0,080628
1647	82,50	171,00	28,214	98,00	25,10	30,057	4,96571	0,080864
1648	81,50	170,00	28,201	98,00	25,10	30,359	5,04030	0,081126
1649	81,30	172,50	27,322	97,00	25,10	29,521	4,79294	0,081415
1650	84,30	170,00	29,170	101,00	25,10	31,116	5,23615	0,081748
1651	83,00	172,00	28,056	99,00	25,10	28,470	4,95618	0,081757
1652	72,60	167,00	26,032	93,00	25,10	27,820	4,93401	0,081932
1653	85,40	180,00	26,358	98,00	25,10	27,373	4,35001	0,082474
1654	81,00	173,00	27,064	98,00	25,10	27,361	4,82045	0,082656
1655	84,00	176,00	27,118	99,00	25,10	27,548	4,67270	0,082675
1656	72,00	166,50	25,972	95,00	25,10	27,495	5,10792	0,083949
1657	61,00	156,00	25,066	92,00	25,10	26,203	5,77556	0,086001
1658	95,10	182,00	28,710	95,00	25,20	29,337	4,05516	0,075104
1659	88,50	176,00	28,571	94,00	25,20	29,359	4,36867	0,075816
1660	71,10	161,00	27,429	88,00	25,20	29,426	5,04770	0,076253
1661	74,60	162,00	28,426	92,00	25,20	30,524	5,25645	0,077605
1662	83,50	167,00	29,940	97,00	25,20	32,314	5,20457	0,077847
1663	86,00	180,00	26,543	94,00	25,20	26,773	4,11766	0,078739
1664	67,50	156,50	27,560	90,00	25,20	28,917	5,57569	0,078850
1665	83,40	174,00	27,547	95,00	25,20	29,011	4,56301	0,078959
1666	91,80	176,00	29,636	101,00	25,20	31,581	4,79439	0,079498
1667	83,70	176,00	27,021	95,00	25,20	27,534	4,42946	0,079524
1668	78,90	166,00	28,633	96,00	25,20	30,977	5,21540	0,079612
1669	85,10	175,00	27,788	97,00	25,20	28,658	4,61867	0,079925
1670	77,30	167,00	27,717	95,00	25,20	29,116	5,06927	0,080267
1671	83,30	170,50	28,655	99,00	25,20	31,106	5,06772	0,080967
1672	74,20	161,00	28,625	97,00	25,20	30,969	5,70324	0,081694
1673	80,00	171,00	27,359	97,00	25,20	29,453	4,90123	0,081697
1674	79,20	168,50	27,895	98,00	25,20	30,208	5,15470	0,082081
1675	76,40	170,50	26,281	95,00	25,20	27,886	4,80825	0,082306
1676	82,00	178,00	25,881	97,00	25,20	27,757	4,41928	0,083096
1677	86,40	179,00	26,965	100,00	25,20	28,159	4,53137	0,083119
1678	86,40	179,00	26,965	100,00	25,20	27,810	4,53137	0,083119
1679	66,80	161,50	25,611	92,00	25,20	25,880	5,29748	0,083320
1680	93,70	184,00	27,676	104,00	25,20	28,181	4,43877	0,083796
1681	79,00	173,00	26,396	98,00	25,20	26,694	4,82045	0,084045
1682	71,00	165,00	26,079	95,00	25,20	27,009	5,22602	0,084098
1683	83,60	182,50	25,100	98,00	25,20	26,114	4,19510	0,084620
1684	76,60	172,00	25,892	98,00	25,20	27,268	4,89244	0,085378
1685	73,80	170,00	25,536	98,00	25,20	26,553	5,04030	0,086675
1686	98,00	186,00	28,327	112,00	25,20	29,041	4,75062	0,088375
1687	82,10	173,00	27,432	89,00	25,30	27,630	4,25403	0,074393
1688	84,00	173,00	28,066	92,00	25,30	29,769	4,44274	0,075737
1689	80,90	171,00	27,667	93,00	25,30	29,273	4,64339	0,077746
1690	72,70	165,00	26,703	90,00	25,30	28,177	4,87959	0,078425
1691	91,50	180,00	28,241	98,00	25,30	29,139	4,35001	0,078766
1692	88,20	176,00	28,474	98,00	25,30	30,582	4,61188	0,079221
1693	87,40	176,00	28,215	98,00	25,30	29,318	4,61188	0,079704
1694	87,20	179,50	27,064	97,00	25,30	27,592	4,32334	0,080318

1695	80,40	173,00	26,864	95,00	25,30	28,145	4,63155	0,080524
1696	96,60	185,50	28,073	102,00	25,30	29,028	4,23620	0,081078
1697	88,20	173,00	29,470	102,00	25,30	32,053	5,07248	0,081282
1698	82,30	170,00	28,478	100,00	25,30	30,171	5,17086	0,082245
1699	82,70	171,00	28,282	100,00	25,30	30,035	5,09472	0,082381
1700	70,50	161,00	27,198	95,00	25,30	27,999	5,55746	0,082785
1701	87,50	174,00	28,901	103,00	25,30	30,088	5,06110	0,082913
1702	72,20	165,00	26,520	96,00	25,30	28,305	5,29535	0,084039
1703	92,00	180,00	28,395	106,00	25,30	29,548	4,81515	0,084887
1704	79,40	166,00	28,814	104,00	25,30	31,292	5,76370	0,085884
1705	86,20	180,00	26,605	104,00	25,30	28,172	4,69881	0,086981
1706	75,00	171,00	25,649	99,00	25,30	25,931	5,03021	0,087048
1707	78,60	176,00	25,374	105,00	25,30	26,472	5,03788	0,091658
1708	77,00	164,00	28,629	89,00	25,40	30,870	4,88573	0,074262
1709	75,70	163,00	28,492	89,00	25,40	30,500	4,96253	0,074728
1710	96,70	189,00	27,071	93,00	25,40	27,717	3,55795	0,075033
1711	89,00	175,00	29,061	95,00	25,40	30,490	4,49566	0,075973
1712	92,80	176,00	29,959	98,00	25,40	31,874	4,61188	0,076581
1713	74,40	166,00	27,000	90,00	25,40	28,435	4,80473	0,077616
1714	77,10	166,00	27,979	93,00	25,40	28,799	5,01001	0,078320
1715	87,60	175,00	28,604	97,00	25,40	29,711	4,61867	0,078397
1716	71,80	166,00	26,056	89,00	25,40	27,726	4,73633	0,078595
1717	85,40	176,00	27,570	96,00	25,40	29,597	4,49026	0,079291
1718	77,60	170,00	26,851	94,00	25,40	28,247	4,77932	0,080401
1719	84,60	178,50	26,552	97,00	25,40	27,865	4,38703	0,081575
1720	81,40	176,00	26,278	96,00	25,40	28,249	4,49026	0,081868
1721	84,00	172,00	28,394	100,00	25,40	29,964	5,01993	0,081926
1722	89,00	175,00	29,061	103,00	25,40	31,085	4,98798	0,082371
1723	78,00	175,00	25,469	95,00	25,40	24,907	4,49566	0,082958
1724	86,00	179,00	26,841	100,00	25,40	28,821	4,53137	0,083377
1725	70,50	164,50	26,053	94,00	25,40	27,971	5,19637	0,083395
1726	71,70	166,00	26,020	95,00	25,40	26,682	5,14693	0,083972
1727	85,40	176,00	27,570	102,00	25,40	28,040	4,85524	0,084247
1728	73,50	170,00	25,433	95,00	25,40	25,506	4,84455	0,084251
1729	89,10	174,50	29,261	107,00	25,40	31,421	5,27224	0,085302
1730	75,10	162,00	28,616	103,00	25,40	29,316	6,04841	0,086498
1731	87,00	180,00	26,852	107,00	25,40	28,247	4,87334	0,088940
1732	77,80	170,00	26,920	89,00	25,50	28,101	4,45336	0,075994
1733	86,70	175,50	28,149	95,00	25,50	29,765	4,46241	0,077495
1734	73,50	158,00	29,442	93,00	25,50	31,348	5,67154	0,077596
1735	80,80	165,00	29,679	96,00	25,50	30,877	5,29535	0,077965
1736	83,40	174,00	27,547	94,00	25,50	28,566	4,50079	0,078128
1737	76,70	168,00	27,175	93,00	25,50	27,740	4,85938	0,079380
1738	83,00	171,50	28,220	97,00	25,50	30,593	4,86481	0,079911
1739	92,90	183,50	27,589	99,00	25,50	29,177	4,19080	0,080043
1740	87,00	177,00	27,770	98,00	25,50	29,182	4,54472	0,080326
1741	75,80	172,00	25,622	92,00	25,50	26,151	4,51025	0,080714
1742	89,00	177,00	28,408	100,00	25,50	30,294	4,66499	0,080733
1743	91,20	177,00	29,110	102,00	25,50	31,144	4,78530	0,081018
1744	93,90	186,00	27,142	100,00	25,50	28,040	4,09748	0,081186

1745	89,40	173,00	29,871	103,00	25,50	31,769	5,13552	0,081342
1746	80,40	175,50	26,104	95,00	25,50	28,158	4,46241	0,081492
1747	72,00	159,00	28,480	96,00	25,50	30,278	5,80752	0,081636
1748	87,40	180,00	26,975	99,00	25,50	27,822	4,40812	0,082039
1749	68,00	163,00	25,594	91,00	25,50	25,727	5,10451	0,082071
1750	81,10	172,00	27,413	98,00	25,50	27,606	4,89244	0,082190
1751	88,50	180,00	27,315	101,00	25,50	29,172	4,52437	0,083002
1752	92,50	181,50	28,079	104,00	25,50	29,892	4,59926	0,083561
1753	85,00	175,00	27,755	102,00	25,50	29,712	4,92640	0,084111
1754	82,40	177,00	26,302	99,00	25,50	27,422	4,60485	0,084139
1755	79,00	168,00	27,990	102,00	25,50	30,537	5,46107	0,085364
1756	78,90	170,00	27,301	101,00	25,50	28,361	5,23615	0,085437
1757	77,00	174,00	25,433	100,00	25,50	26,938	4,87424	0,087659
1758	83,40	179,00	26,029	105,00	25,50	26,071	4,82542	0,089356
1759	79,00	175,00	25,796	179,00	25,50	27,652	9,69916	0,154989
1760	83,00	169,00	29,061	89,00	25,60	30,092	4,52221	0,072429
1761	88,00	178,00	27,774	90,00	25,60	29,297	4,00350	0,073554
1762	87,30	175,00	28,506	93,00	25,60	30,310	4,37268	0,075336
1763	92,10	183,00	27,502	94,00	25,60	28,619	3,94020	0,076266
1764	98,40	185,00	28,751	98,00	25,60	30,304	4,04649	0,076773
1765	87,40	177,00	27,897	94,00	25,60	28,692	4,30431	0,076812
1766	88,00	176,00	28,409	95,00	25,60	29,253	4,42946	0,076912
1767	82,70	175,00	27,004	92,00	25,60	27,628	4,31121	0,077265
1768	85,90	180,50	26,366	92,00	25,60	28,113	3,97200	0,077302
1769	92,70	179,00	28,932	98,00	25,60	30,829	4,41381	0,077723
1770	81,40	172,00	27,515	93,00	25,60	28,078	4,57392	0,077805
1771	83,00	172,00	28,056	95,00	25,60	30,399	4,70129	0,078453
1772	73,30	164,30	27,154	92,00	25,60	28,170	5,07255	0,079448
1773	74,00	165,50	27,017	93,00	25,60	28,225	5,04853	0,080290
1774	68,20	164,00	25,357	89,00	25,60	26,580	4,88573	0,080520
1775	94,50	185,50	27,463	100,00	25,60	28,009	4,12683	0,080661
1776	91,00	187,00	26,023	97,00	25,60	25,938	3,87814	0,080775
1777	84,50	181,00	25,793	95,00	25,60	27,395	4,11494	0,080888
1778	92,50	177,00	29,525	103,00	25,60	31,352	4,84547	0,081044
1779	83,70	172,00	28,292	99,00	25,60	29,311	4,95618	0,081300
1780	78,00	173,00	26,062	94,00	25,60	27,360	4,56860	0,081302
1781	89,20	175,00	29,127	102,00	25,60	30,670	4,92640	0,081450
1782	77,50	171,00	26,504	95,00	25,60	26,840	4,77229	0,081725
1783	86,00	174,00	28,405	101,00	25,60	29,039	4,93652	0,082246
1784	78,10	166,00	28,342	100,00	25,60	30,633	5,48945	0,083494
1785	90,70	175,00	29,616	107,00	25,60	32,127	5,23439	0,084498
1786	77,60	170,00	26,851	100,00	25,60	28,716	5,17086	0,085533
1787	70,10	158,00	28,080	100,00	25,60	30,428	6,20177	0,086113
1788	89,70	176,00	28,958	108,00	25,60	30,860	5,22060	0,086329
1789	74,90	172,00	25,318	98,00	25,60	25,611	4,89244	0,086665
1790	81,90	175,00	26,743	103,00	25,60	26,926	4,98798	0,087066
1791	86,90	175,00	28,376	90,00	25,70	30,150	4,18830	0,073130
1792	89,10	172,50	29,943	96,00	25,70	32,133	4,72960	0,075801
1793	79,50	171,00	27,188	91,00	25,70	29,245	4,51454	0,076965
1794	86,80	180,50	26,642	93,00	25,70	27,861	4,02972	0,077601

1795	80,00	169,00	28,010	94,00	25,70	30,238	4,85209	0,078398
1796	75,60	166,00	27,435	92,00	25,70	28,421	4,94157	0,078499
1797	80,00	173,50	26,576	94,00	25,70	27,776	4,53455	0,080134
1798	84,00	176,50	26,964	96,00	25,70	27,924	4,45723	0,080360
1799	82,40	174,00	27,216	96,00	25,70	27,452	4,62523	0,080435
1800	98,00	189,50	27,290	101,00	25,70	27,779	3,95088	0,080943
1801	92,50	182,00	27,925	101,00	25,70	29,053	4,39605	0,081337
1802	81,90	177,00	26,142	96,00	25,70	27,710	4,42449	0,081921
1803	94,80	179,00	29,587	108,00	25,70	30,952	5,00197	0,084384
1804	79,30	170,00	27,439	101,00	25,70	28,315	5,23615	0,085149
1805	96,00	180,00	29,630	110,00	25,70	31,289	5,04795	0,085626
1806	77,00	172,00	26,028	99,00	25,70	25,944	4,95618	0,085951
1807	82,60	173,50	27,440	103,00	25,70	28,315	5,09815	0,085954
1808	76,00	173,00	25,393	101,00	25,70	26,105	5,00946	0,088883
1809	80,90	179,00	25,249	104,00	25,70	26,968	4,76659	0,090319
1810	86,80	172,50	29,170	95,00	25,80	31,019	4,66627	0,076331
1811	86,00	169,50	29,934	97,00	25,80	32,029	5,01244	0,077282
1812	78,90	165,00	28,981	94,00	25,80	29,691	5,15671	0,077562
1813	79,70	167,00	28,578	94,00	25,80	30,706	5,00163	0,077819
1814	84,20	174,00	27,811	95,00	25,80	29,562	4,56301	0,078458
1815	77,20	169,00	27,030	93,00	25,80	29,168	4,78609	0,079429
1816	81,00	170,50	27,864	96,00	25,80	30,171	4,87310	0,079993
1817	91,40	178,00	28,847	101,00	25,80	29,819	4,65708	0,080484
1818	82,00	178,00	25,881	94,00	25,80	25,488	4,24103	0,080526
1819	86,00	175,00	28,082	99,00	25,80	29,360	4,74173	0,081003
1820	83,40	176,00	26,924	97,00	25,80	27,872	4,55106	0,081393
1821	88,50	175,00	28,898	102,00	25,80	30,587	4,92640	0,081878
1822	83,80	172,00	28,326	101,00	25,80	30,404	5,08369	0,082877
1823	86,50	183,00	25,829	98,00	25,80	27,694	4,16488	0,082907
1824	87,60	173,00	29,269	104,00	25,80	31,139	5,19856	0,083253
1825	95,80	183,50	28,451	106,00	25,80	30,034	4,58221	0,083964
1826	85,60	173,00	28,601	104,00	25,80	31,043	5,19856	0,084545
1827	88,00	185,00	25,712	106,00	25,80	27,037	4,48645	0,089460
1828	80,00	167,00	28,685	93,00	25,90	30,325	4,93401	0,076799
1829	78,30	163,00	29,470	95,00	25,90	31,478	5,38865	0,077990
1830	72,90	165,00	26,777	90,00	25,90	27,208	4,87959	0,078282
1831	80,70	174,00	26,655	93,00	25,90	28,711	4,43859	0,079012
1832	95,90	179,00	29,930	102,00	25,90	32,026	4,64896	0,079086
1833	74,20	161,00	28,625	95,00	25,90	30,661	5,55746	0,080010
1834	95,60	182,00	28,861	102,00	25,90	29,938	4,45290	0,080357
1835	92,70	179,00	28,932	102,00	25,90	30,929	4,64896	0,080896
1836	64,10	156,00	26,340	90,00	25,90	26,984	5,62026	0,081397
1837	88,00	173,00	29,403	102,00	25,90	30,721	5,07248	0,081405
1838	78,20	170,00	27,059	96,00	25,90	28,047	4,90978	0,081691
1839	78,00	172,00	26,366	95,00	25,90	27,748	4,70129	0,081771
1840	78,60	170,00	27,197	97,00	25,90	28,227	4,97503	0,082262
1841	75,60	172,00	25,554	94,00	25,90	27,351	4,63760	0,082614
1842	80,30	170,50	27,623	99,00	25,90	29,550	5,06772	0,082971
1843	84,00	176,00	27,118	100,00	25,90	28,699	4,73354	0,083510
1844	79,00	171,00	27,017	99,00	25,90	28,109	5,03021	0,084084

1845	72,60	162,00	27,663	98,00	25,90	29,711	5,68821	0,084178
1846	69,70	165,50	25,447	96,00	25,90	26,566	5,25519	0,086254
1847	81,00	165,00	29,752	89,00	26,00	32,189	4,81034	0,072161
1848	90,20	175,00	29,453	96,00	26,00	30,592	4,55716	0,076091
1849	89,80	180,00	27,716	95,00	26,00	29,334	4,17573	0,077315
1850	85,50	172,50	28,733	96,00	26,00	29,266	4,72960	0,077914
1851	83,30	170,00	28,824	96,00	26,00	29,889	4,90978	0,078322
1852	90,00	181,50	27,321	96,00	26,00	28,613	4,14203	0,078555
1853	75,30	168,00	26,679	91,00	26,00	28,266	4,72581	0,078633
1854	80,40	172,50	27,020	93,00	26,00	28,344	4,53964	0,078639
1855	78,00	170,50	26,832	93,00	26,00	27,987	4,67859	0,079468
1856	93,60	179,00	29,213	104,00	26,00	30,972	4,76659	0,081952
1857	74,70	169,00	26,155	94,00	26,00	27,355	4,85209	0,082064
1858	78,20	174,00	25,829	95,00	26,00	27,567	4,56301	0,082422
1859	84,30	175,00	27,527	100,00	26,00	27,759	4,80328	0,082918
1860	98,70	190,00	27,341	104,00	26,00	28,639	4,07938	0,083135
1861	88,20	178,00	27,837	102,00	26,00	28,287	4,71655	0,083235
1862	87,50	177,00	27,929	102,00	26,00	28,950	4,78530	0,083286
1863	93,70	183,00	27,979	104,00	26,00	28,583	4,50217	0,083416
1864	82,20	173,00	27,465	100,00	26,00	29,693	4,94644	0,083520
1865	90,70	174,00	29,958	107,00	26,00	32,520	5,31041	0,084095
1866	90,40	182,50	27,142	103,00	26,00	28,500	4,47771	0,084420
1867	77,30	165,00	28,393	101,00	26,00	30,694	5,64218	0,084483
1868	80,00	173,00	26,730	100,00	26,00	27,026	4,94644	0,085044
1869	85,70	173,00	28,634	105,00	26,00	30,775	5,26162	0,085292
1870	71,90	165,00	26,410	98,00	26,00	28,168	5,43404	0,086029
1871	71,80	166,00	26,056	100,00	26,00	27,850	5,48945	0,088309
1872	80,30	168,00	28,451	91,00	26,10	29,203	4,72581	0,075334
1873	89,60	176,00	28,926	95,00	26,10	30,420	4,42946	0,075994
1874	94,00	186,50	27,025	94,00	26,10	29,047	3,74400	0,076432
1875	88,80	175,00	28,996	97,00	26,10	30,807	4,61867	0,077689
1876	90,30	174,00	29,826	99,00	26,10	31,901	4,81197	0,078037
1877	89,60	179,50	27,809	96,00	26,10	29,122	4,26492	0,078064
1878	93,40	186,00	26,997	97,00	26,10	28,084	3,93438	0,079032
1879	84,00	170,00	29,066	98,00	26,10	31,585	5,04030	0,079509
1880	78,60	168,00	27,849	95,00	26,10	29,499	4,99300	0,079775
1881	72,40	160,00	28,281	94,00	26,10	29,929	5,57048	0,080058
1882	86,70	173,00	28,969	100,00	26,10	31,173	4,94644	0,080604
1883	84,60	176,00	27,311	97,00	26,10	29,395	4,55106	0,080622
1884	88,10	180,00	27,191	98,00	26,10	28,105	4,35001	0,080780
1885	88,00	177,00	28,089	100,00	26,10	28,514	4,66499	0,081343
1886	78,50	172,00	26,535	96,00	26,10	28,444	4,76500	0,082281
1887	85,00	177,00	27,131	99,00	26,10	28,601	4,60485	0,082414
1888	86,30	173,00	28,835	102,00	26,10	30,207	5,07248	0,082470
1889	83,80	167,50	29,869	103,00	26,10	31,580	5,56920	0,082671
1890	90,50	177,00	28,887	106,00	26,10	31,277	5,02604	0,084629
1891	98,00	189,00	27,435	106,00	26,10	27,972	4,24224	0,084763
1892	78,30	166,00	28,415	102,00	26,10	30,824	5,62655	0,085019
1893	73,00	168,00	25,865	98,00	26,10	27,233	5,19353	0,086451
1894	87,30	176,00	28,183	107,00	26,10	29,595	5,15969	0,087090

1895	83,60	174,00	27,613	88,00	26,20	29,871	4,12774	0,073025
1896	86,50	174,50	28,407	92,00	26,20	29,980	4,34366	0,074807
1897	86,80	178,00	27,396	94,00	26,20	28,708	4,24103	0,077529
1898	75,70	168,00	26,821	91,00	26,20	28,326	4,72581	0,078355
1899	85,00	169,50	29,586	99,00	26,20	32,186	5,14376	0,079493
1900	73,40	160,00	28,672	95,00	26,20	31,024	5,64428	0,080173
1901	89,00	178,00	28,090	99,00	26,20	30,547	4,53816	0,080302
1902	94,00	178,00	29,668	103,00	26,20	30,674	4,77603	0,080557
1903	75,30	164,50	27,827	95,00	26,20	29,581	5,26612	0,080661
1904	84,10	169,00	29,446	100,00	26,20	31,929	5,24836	0,080669
1905	81,00	166,50	29,218	99,00	26,20	31,371	5,38024	0,080877
1906	86,70	173,00	28,969	101,00	26,20	31,472	5,00946	0,081410
1907	87,00	176,00	28,086	100,00	26,20	29,794	4,73354	0,081580
1908	80,00	171,50	27,200	97,00	26,20	29,373	4,86481	0,081896
1909	77,50	171,50	26,350	96,00	26,20	28,215	4,80072	0,082786
1910	92,80	177,00	29,621	107,00	26,20	32,132	5,08625	0,084010
1911	91,00	178,50	28,560	105,00	26,20	30,170	4,86008	0,084112
1912	84,10	178,00	26,543	100,00	26,20	28,215	4,59761	0,084234
1913	76,60	172,00	25,892	97,00	26,20	27,520	4,82871	0,084507
1914	80,40	168,00	28,486	105,00	26,20	30,286	5,66185	0,086851
1915	96,50	180,00	29,784	113,00	26,20	32,133	5,22265	0,087657
1916	78,00	165,00	28,650	106,00	26,20	31,100	5,98934	0,088135
1917	78,80	177,00	25,152	101,00	26,20	25,917	4,72514	0,088433
1918	74,70	170,50	25,696	88,00	26,30	27,017	4,35463	0,077394
1919	82,10	171,00	28,077	94,00	26,30	29,782	4,70783	0,077815
1920	77,10	167,00	27,645	92,00	26,30	28,693	4,86640	0,077866
1921	76,40	166,00	27,725	92,00	26,30	30,006	4,94157	0,077950
1922	86,50	179,00	26,997	94,00	26,30	27,618	4,17882	0,078072
1923	97,80	185,00	28,576	100,00	26,30	29,571	4,15643	0,078659
1924	82,60	175,50	26,818	94,00	26,30	28,910	4,40128	0,079196
1925	78,10	169,50	27,184	94,00	26,30	27,980	4,81554	0,079861
1926	96,60	180,00	29,815	104,00	26,30	31,328	4,69881	0,080620
1927	84,90	175,00	27,722	98,00	26,30	28,243	4,68020	0,080876
1928	94,50	180,00	29,167	105,00	26,30	29,933	4,75698	0,082597
1929	76,60	174,00	25,301	96,00	26,30	26,376	4,62523	0,084445
1930	87,20	174,00	28,802	105,00	26,30	29,861	5,18574	0,084717
1931	85,80	176,00	27,699	103,00	26,30	29,533	4,91611	0,084809
1932	88,00	175,00	28,735	106,00	26,30	29,369	5,17278	0,085411
1933	91,90	178,00	29,005	97,00	26,40	29,822	4,41928	0,077015
1934	80,80	170,00	27,958	93,00	26,40	29,636	4,71411	0,077431
1935	84,40	172,00	28,529	95,00	26,40	30,855	4,70129	0,077583
1936	80,00	167,50	28,514	94,00	26,40	30,113	4,96374	0,077818
1937	74,90	168,00	26,538	90,00	26,40	28,328	4,65905	0,078045
1938	78,20	169,50	27,219	92,00	26,40	29,397	4,68434	0,078095
1939	96,40	188,00	27,275	98,00	26,40	27,872	3,87600	0,078881
1940	74,30	167,00	26,641	91,00	26,40	28,695	4,79881	0,078943
1941	86,20	173,00	28,801	99,00	26,40	30,772	4,88344	0,080107
1942	88,80	175,00	28,996	101,00	26,40	31,006	4,86484	0,080893
1943	82,00	177,00	26,174	95,00	26,40	26,023	4,36440	0,081001
1944	77,50	163,50	28,991	98,00	26,40	30,701	5,55935	0,081213

1945	73,50	165,00	26,997	94,00	26,40	29,128	5,15671	0,081315
1946	81,50	171,00	27,872	98,00	26,40	29,202	4,96571	0,081524
1947	92,60	177,00	29,557	104,00	26,40	31,963	4,90565	0,081772
1948	88,60	181,00	27,044	100,00	26,40	28,260	4,40219	0,082498
1949	81,70	169,00	28,605	101,00	26,40	29,815	5,31445	0,083064
1950	84,40	180,00	26,049	99,00	26,40	25,974	4,40812	0,083972
1951	82,00	169,00	28,710	103,00	26,40	30,967	5,44666	0,084502
1952	80,30	174,60	26,341	99,00	26,40	27,107	4,76968	0,084631
1953	81,00	176,00	26,149	99,00	26,40	27,719	4,67270	0,084704
1954	82,40	175,00	26,906	101,00	26,40	27,615	4,86484	0,085029
1955	81,60	173,00	27,265	102,00	26,40	28,655	5,07248	0,085608
1956	76,40	164,40	28,268	105,00	26,40	30,018	5,97322	0,088249
1957	80,20	175,00	26,188	104,00	26,40	27,521	5,04957	0,089149
1958	83,30	169,00	29,166	112,00	26,40	31,603	6,04221	0,090927
1959	78,50	164,00	29,186	90,00	26,50	30,450	4,95584	0,074137
1960	91,50	178,00	28,879	94,00	26,50	30,161	4,24103	0,074851
1961	82,60	169,00	28,921	93,00	26,50	30,114	4,78609	0,075928
1962	82,00	175,00	26,776	90,00	26,50	26,970	4,18830	0,076015
1963	78,70	168,50	27,719	95,00	26,50	29,118	4,95539	0,079905
1964	102,80	187,50	29,241	104,00	26,50	30,029	4,22489	0,080021
1965	86,70	174,00	28,637	99,00	26,50	29,445	4,81197	0,080183
1966	86,10	170,00	29,792	101,00	26,50	31,488	5,23615	0,080605
1967	82,90	169,00	29,026	100,00	26,50	31,439	5,24836	0,081446
1968	88,20	178,00	27,837	100,00	26,50	29,921	4,59761	0,081603
1969	84,70	175,00	27,657	99,00	26,50	28,598	4,74173	0,081830
1970	92,30	178,00	29,131	104,00	26,50	30,577	4,83553	0,082335
1971	72,00	163,00	27,099	95,00	26,50	28,906	5,38865	0,082475
1972	79,60	174,00	26,291	97,00	26,50	27,042	4,68747	0,083168
1973	78,00	176,00	25,181	95,00	26,50	26,882	4,42946	0,083353
1974	78,70	171,00	26,914	98,00	26,50	28,794	4,96571	0,083446
1975	78,00	170,00	26,990	98,00	26,50	28,306	5,04030	0,083535
1976	78,80	176,00	25,439	96,00	26,50	27,337	4,49026	0,083659
1977	85,00	181,00	25,945	99,00	26,50	26,960	4,34472	0,083963
1978	75,00	170,50	25,800	96,00	26,50	27,403	4,87310	0,084204
1979	84,30	175,50	27,370	103,00	26,50	27,659	4,95189	0,085608
1980	77,60	172,00	26,230	100,00	26,50	27,699	5,01993	0,086371
1981	86,40	179,00	26,965	104,00	26,50	27,111	4,76659	0,086444
1982	78,80	165,00	28,944	94,00	26,60	31,344	5,15671	0,077627
1983	84,00	172,00	28,394	95,00	26,60	29,128	4,70129	0,077830
1984	89,10	182,00	26,899	96,00	26,60	28,775	4,11195	0,079265
1985	86,80	173,00	29,002	99,00	26,60	30,814	4,88344	0,079737
1986	86,10	177,00	27,483	97,00	26,60	29,043	4,48460	0,080060
1987	79,00	170,00	27,336	95,00	26,60	28,066	4,84455	0,080293
1988	77,30	165,00	28,393	96,00	26,60	30,485	5,29535	0,080301
1989	79,80	172,00	26,974	96,00	26,60	28,170	4,76500	0,081385
1990	86,80	175,50	28,182	100,00	26,60	30,442	4,76826	0,081511
1991	84,80	171,00	29,000	102,00	26,60	30,912	5,22378	0,082635
1992	74,40	170,00	25,744	94,00	26,60	25,805	4,77932	0,082690
1993	87,00	176,00	28,086	103,00	26,60	30,435	4,91611	0,084027
1994	91,80	182,00	27,714	104,00	26,60	28,012	4,56662	0,084178

1995	90,80	181,00	27,716	104,00	26,60	29,554	4,63216	0,084406
1996	69,00	164,00	25,654	95,00	26,60	26,323	5,30658	0,085283
1997	70,00	156,50	28,580	100,00	26,60	30,298	6,34781	0,085513
1998	88,00	180,00	27,160	93,00	26,70	28,982	4,05959	0,076716
1999	80,70	167,00	28,936	94,00	26,70	30,734	5,00163	0,077175
2000	78,00	174,00	25,763	89,00	26,70	26,848	4,18989	0,077348
2001	94,60	180,00	29,198	100,00	26,70	30,856	4,46624	0,078608
2002	77,80	163,00	29,282	96,00	26,70	31,739	5,45972	0,079148
2003	87,00	174,00	28,736	98,00	26,70	30,387	4,74971	0,079190
2004	77,00	171,00	26,333	94,00	26,70	27,097	4,70783	0,081214
2005	75,00	168,00	26,573	94,00	26,70	28,132	4,92619	0,081441
2006	84,30	172,00	28,495	100,00	26,70	30,918	5,01993	0,081731
2007	88,50	180,00	27,315	100,00	26,70	28,265	4,46624	0,082180
2008	92,00	176,00	29,700	105,00	26,70	31,281	5,03788	0,082526
2009	74,80	171,00	25,581	94,00	26,70	25,709	4,70783	0,082799
2010	91,00	186,00	26,304	100,00	26,70	27,669	4,09748	0,082902
2011	70,80	165,00	26,006	94,00	26,70	27,538	5,15671	0,083370
2012	91,30	180,00	28,179	104,00	26,70	29,377	4,69881	0,083710
2013	90,00	177,00	28,727	106,00	26,70	30,479	5,02604	0,084942
2014	76,60	169,00	26,820	99,00	26,70	28,324	5,18229	0,084994
2015	81,20	175,00	26,514	100,00	26,70	28,299	4,80328	0,085015
2016	78,20	167,00	28,040	102,00	26,70	30,487	5,54305	0,085518
2017	75,20	165,00	27,622	101,00	26,70	28,884	5,64218	0,086049
2018	82,80	179,00	25,842	101,00	26,70	27,204	4,59016	0,086367
2019	85,00	179,00	26,529	104,00	26,70	28,196	4,76659	0,087391
2020	82,00	172,00	27,718	92,00	26,80	30,106	4,51025	0,076592
2021	90,10	180,00	27,809	95,00	26,80	29,013	4,17573	0,077144
2022	80,50	166,00	29,213	95,00	26,80	31,071	5,14693	0,077735
2023	86,30	174,00	28,504	96,00	26,80	29,998	4,62523	0,077993
2024	80,00	171,00	27,359	93,00	26,80	27,984	4,64339	0,078328
2025	92,90	180,00	28,673	100,00	26,80	30,105	4,46624	0,079564
2026	76,90	163,00	28,944	96,00	26,80	30,943	5,45972	0,079765
2027	82,30	168,00	29,160	98,00	26,80	31,498	5,19353	0,079809
2028	86,00	172,00	29,070	100,00	26,80	29,906	5,01993	0,080651
2029	94,50	179,00	29,493	104,00	26,80	31,122	4,76659	0,081431
2030	90,00	179,00	28,089	101,00	26,80	30,439	4,59016	0,081697
2031	76,20	169,50	26,523	95,00	26,80	28,549	4,88116	0,082047
2032	84,70	178,00	26,733	99,00	26,80	27,267	4,53816	0,082997
2033	85,00	175,00	27,755	101,00	26,80	29,931	4,86484	0,083286
2034	96,60	182,00	29,163	107,00	26,80	30,420	4,73727	0,083713
2035	71,20	166,00	25,838	95,00	26,80	27,325	5,14693	0,084365
2036	79,00	173,00	26,396	100,00	26,80	27,423	4,94644	0,085760
2037	78,60	177,00	25,089	98,00	26,80	25,832	4,54472	0,085952
2038	81,00	166,00	29,395	106,00	26,80	31,001	5,90091	0,086379
2039	87,00	182,00	26,265	103,00	26,80	26,517	4,50976	0,086407
2040	96,00	184,00	28,355	97,00	26,90	29,392	4,04963	0,076902
2041	88,80	178,00	28,027	95,00	26,90	29,076	4,30044	0,077173
2042	90,30	174,00	29,826	98,00	26,90	31,528	4,74971	0,077249
2043	81,00	168,00	28,699	95,00	26,90	30,444	4,99300	0,078191
2044	74,50	164,00	27,699	92,00	26,90	28,653	5,09610	0,078473

2045	77,10	166,50	27,812	93,00	26,90	29,999	4,97184	0,078516
2046	75,90	168,00	26,892	93,00	26,90	27,713	4,85938	0,079937
2047	81,30	165,00	29,862	99,00	26,90	31,480	5,50340	0,080072
2048	73,30	167,00	26,283	92,00	26,90	28,132	4,86640	0,080535
2049	95,80	180,00	29,568	104,00	26,90	31,309	4,69881	0,081068
2050	84,90	170,00	29,377	101,00	26,90	31,076	5,23615	0,081362
2051	83,20	170,00	28,789	100,00	26,90	31,162	5,17086	0,081650
2052	93,10	180,00	28,735	103,00	26,90	30,793	4,64065	0,081834
2053	90,30	177,00	28,823	103,00	26,90	29,485	4,84547	0,082355
2054	85,40	175,00	27,886	101,00	26,90	29,111	4,86484	0,083026
2055	78,50	174,00	25,928	96,00	26,90	27,691	4,62523	0,083077
2056	83,00	175,00	27,102	100,00	26,90	28,564	4,80328	0,083781
2057	84,10	177,00	26,844	100,00	26,90	27,650	4,66499	0,083839
2058	83,70	175,00	27,331	102,00	26,90	27,720	4,92640	0,084980
2059	75,00	170,00	25,952	99,00	26,90	27,845	5,10557	0,086623
2060	88,80	174,00	29,330	93,00	27,00	30,145	4,43859	0,074131
2061	89,40	175,00	29,192	93,00	27,00	30,260	4,37268	0,074152
2062	94,70	178,00	29,889	97,00	27,00	31,327	4,41928	0,075490
2063	79,00	170,00	27,336	90,00	27,00	28,745	4,51853	0,076067
2064	92,80	177,00	29,621	99,00	27,00	30,710	4,60485	0,077729
2065	84,00	174,00	27,745	95,00	27,00	29,809	4,56301	0,078583
2066	83,60	176,00	26,989	94,00	27,00	27,956	4,36867	0,078750
2067	86,30	174,00	28,504	98,00	27,00	30,308	4,74971	0,079618
2068	79,30	167,00	28,434	96,00	27,00	30,743	5,13691	0,079742
2069	87,80	173,00	29,336	100,00	27,00	30,832	4,94644	0,079930
2070	105,00	189,00	29,394	105,00	27,00	30,131	4,18956	0,080189
2071	76,50	162,50	28,970	97,00	27,00	31,275	5,57331	0,080669
2072	81,50	172,50	27,389	97,00	27,00	28,700	4,79294	0,081281
2073	85,00	170,00	29,412	101,00	27,00	30,540	5,23615	0,081299
2074	84,00	175,50	27,273	98,00	27,00	29,234	4,64589	0,081646
2075	85,70	175,00	27,984	100,00	27,00	29,775	4,80328	0,082012
2076	87,20	174,00	28,802	102,00	27,00	30,064	4,99881	0,082296
2077	86,40	174,00	28,537	102,00	27,00	30,349	4,99881	0,082804
2078	76,10	167,00	27,287	97,00	27,00	28,570	5,20457	0,082816
2079	87,50	174,00	28,901	103,00	27,00	30,691	5,06110	0,082913
2080	79,00	173,00	26,396	97,00	27,00	27,787	4,75747	0,083188
2081	90,50	180,00	27,932	103,00	27,00	29,387	4,64065	0,083393
2082	80,00	172,00	27,042	101,00	27,00	28,951	5,08369	0,085481
2083	77,10	174,00	25,466	99,00	27,00	27,110	4,81197	0,086707
2084	88,70	174,00	29,297	92,00	27,10	30,783	4,37640	0,073389
2085	88,00	174,00	29,066	95,00	27,10	31,388	4,56301	0,076183
2086	88,00	177,00	28,089	94,00	27,10	29,904	4,30431	0,076463
2087	79,00	166,00	28,669	93,00	27,10	30,918	5,01001	0,077059
2088	85,90	173,50	28,536	95,00	27,10	30,553	4,59713	0,077234
2089	89,10	174,50	29,261	98,00	27,10	31,421	4,71480	0,078127
2090	84,10	178,00	26,543	94,00	27,10	27,494	4,24103	0,079180
2091	83,50	175,00	27,265	95,00	27,10	28,884	4,49566	0,079274
2092	86,00	171,00	29,411	99,00	27,10	31,213	5,03021	0,079457
2093	83,20	171,00	28,453	97,00	27,10	29,622	4,90123	0,079589
2094	79,00	167,00	28,327	96,00	27,10	30,720	5,13691	0,079944

2095	91,00	176,00	29,378	102,00	27,10	31,173	4,85524	0,080754
2096	89,80	175,00	29,322	103,00	27,10	31,009	4,98798	0,081881
2097	82,10	171,00	28,077	99,00	27,10	28,926	5,03021	0,081954
2098	86,20	180,00	26,605	99,00	27,10	27,455	4,40812	0,082799
2099	80,80	169,00	28,290	101,00	27,10	29,309	5,31445	0,083680
2100	91,80	175,00	29,976	107,00	27,10	32,540	5,23439	0,083821
2101	78,10	171,00	26,709	98,00	27,10	28,778	4,96571	0,083873
2102	84,60	175,00	27,624	102,00	27,10	29,109	4,92640	0,084376
2103	84,40	177,00	26,940	101,00	27,10	28,009	4,72514	0,084477
2104	80,00	173,50	26,576	100,00	27,10	27,297	4,91018	0,085249
2105	78,00	168,50	27,472	101,00	27,10	29,590	5,35413	0,085459
2106	74,70	160,00	29,180	103,00	27,10	31,619	6,23521	0,085913
2107	77,80	172,00	26,298	100,00	27,10	28,274	5,01993	0,086223
2108	80,70	179,00	25,186	104,00	27,10	26,094	4,76659	0,090468
2109	82,60	175,00	26,971	90,00	27,20	28,632	4,18830	0,075646
2110	94,50	185,00	27,611	95,00	27,20	28,871	3,88165	0,076456
2111	84,00	176,00	27,118	92,00	27,20	28,699	4,24714	0,076830
2112	99,20	186,00	28,674	100,00	27,20	29,493	4,09748	0,078268
2113	70,80	157,50	28,541	92,00	27,20	29,837	5,64013	0,078493
2114	89,80	175,00	29,322	99,00	27,20	31,786	4,74173	0,078701
2115	99,40	185,00	29,043	102,00	27,20	30,566	4,26640	0,079369
2116	89,30	174,00	29,495	101,00	27,20	30,645	4,93652	0,080207
2117	83,60	172,00	28,259	98,00	27,20	30,323	4,89244	0,080543
2118	85,00	178,00	26,827	97,00	27,20	28,334	4,41928	0,081129
2119	82,50	170,00	28,547	99,00	27,20	30,669	5,10557	0,081291
2120	76,10	162,00	28,997	98,00	27,20	30,709	5,68821	0,081577
2121	97,00	187,00	27,739	103,00	27,20	28,484	4,20089	0,082197
2122	79,60	172,00	26,906	97,00	27,20	26,797	4,82871	0,082370
2123	79,50	168,00	28,168	99,00	27,20	29,894	5,26039	0,082505
2124	76,80	172,00	25,960	95,00	27,20	26,853	4,70129	0,082621
2125	99,20	182,00	29,948	109,00	27,20	31,400	4,85108	0,083781
2126	82,20	167,00	29,474	104,00	27,20	31,482	5,67854	0,084343
2127	67,60	161,00	26,079	97,00	27,20	28,004	5,70324	0,086928
2128	84,40	176,50	27,093	106,00	27,20	28,322	5,06225	0,088450
2129	91,80	176,00	29,636	114,00	27,20	32,149	5,58634	0,089730
2130	80,70	169,00	28,255	95,00	27,30	30,213	4,91811	0,078774
2131	80,10	169,00	28,045	95,00	27,30	29,958	4,91811	0,079166
2132	84,10	176,00	27,150	96,00	27,30	28,166	4,49026	0,080107
2133	87,00	171,00	29,753	102,00	27,30	32,096	5,22378	0,081236
2134	88,80	174,00	29,330	102,00	27,30	31,115	4,99881	0,081305
2135	90,60	188,00	25,634	97,00	27,30	26,680	3,82281	0,081374
2136	74,80	165,00	27,475	97,00	27,30	28,809	5,36469	0,082936
2137	81,10	174,00	26,787	99,00	27,30	27,457	4,81197	0,083832
2138	85,20	176,00	27,505	102,00	27,30	29,071	4,85524	0,084379
2139	93,50	182,00	28,227	106,00	27,30	30,602	4,68038	0,084753
2140	82,00	168,00	29,053	104,00	27,30	30,678	5,59491	0,084901
2141	98,80	184,50	29,024	110,00	27,30	31,438	4,73950	0,085747
2142	96,80	181,00	29,547	95,00	27,40	30,902	4,11494	0,073882
2143	85,50	170,00	29,585	97,00	27,40	32,090	4,97503	0,077774
2144	88,90	180,00	27,438	95,00	27,40	27,976	4,17573	0,077836

2145	93,10	180,00	28,735	98,00	27,40	31,098	4,35001	0,077861
2146	81,70	171,00	27,940	94,00	27,40	29,721	4,70783	0,078069
2147	86,00	170,00	29,758	98,00	27,40	32,008	5,04030	0,078271
2148	81,20	165,00	29,826	97,00	27,40	30,875	5,36469	0,078518
2149	94,00	186,00	27,171	98,00	27,40	27,161	3,98873	0,079506
2150	83,00	173,00	27,732	96,00	27,40	28,915	4,69450	0,079663
2151	96,00	181,00	29,303	102,00	27,40	30,693	4,51716	0,079766
2152	76,00	166,00	27,580	94,00	27,40	29,498	5,07846	0,079924
2153	80,50	178,00	25,407	93,00	27,40	26,254	4,18163	0,080656
2154	102,00	197,50	26,150	101,00	27,40	27,473	3,53131	0,081576
2155	87,40	181,00	26,678	98,00	27,40	28,264	4,28726	0,081586
2156	96,20	183,00	28,726	107,00	27,40	29,968	4,67093	0,084329
2157	84,30	168,50	29,691	105,00	27,40	31,647	5,62020	0,084360
2158	74,00	160,00	28,906	101,00	27,40	30,998	6,08739	0,084775
2159	84,00	175,00	27,429	103,00	27,40	29,312	4,98798	0,085608
2160	95,40	183,00	28,487	109,00	27,40	29,354	4,78348	0,086385
2161	88,10	179,00	27,496	106,00	27,40	28,053	4,88426	0,086969
2162	90,10	176,00	29,087	110,00	27,40	30,819	5,34247	0,087667
2163	83,00	172,00	28,056	94,00	27,50	29,542	4,63760	0,077628
2164	83,30	170,50	28,655	95,00	27,50	29,878	4,80825	0,077696
2165	87,50	175,50	28,409	97,00	27,50	30,295	4,58472	0,078644
2166	87,60	180,00	27,037	96,00	27,50	27,556	4,23381	0,079432
2167	81,80	169,00	28,640	97,00	27,50	29,757	5,05017	0,079709
2168	74,40	169,50	25,896	91,00	27,50	26,895	4,61876	0,079855
2169	80,30	169,00	28,115	96,00	27,50	29,403	4,98414	0,079867
2170	95,00	182,00	28,680	101,00	27,50	29,501	4,39605	0,079903
2171	82,50	172,00	27,887	97,00	27,50	29,872	4,82871	0,080428
2172	75,00	168,00	26,573	94,00	27,50	27,653	4,92619	0,081441
2173	70,80	166,00	25,693	92,00	27,50	27,524	4,94157	0,082008
2174	76,50	164,00	28,443	99,00	27,50	30,753	5,58742	0,082965
2175	86,20	174,00	28,471	103,00	27,50	29,852	5,06110	0,083745
2176	88,00	176,00	28,409	104,00	27,50	30,504	4,97699	0,084199
2177	77,00	164,00	28,629	102,00	27,50	30,665	5,79820	0,085109
2178	82,60	173,00	27,599	104,00	27,50	29,410	5,19856	0,086580
2179	67,50	160,00	26,367	98,00	27,50	27,507	5,86576	0,087456
2180	80,50	168,00	28,522	111,00	27,50	30,640	6,06376	0,091738
2181	84,70	170,00	29,308	90,00	27,60	30,700	4,51853	0,072615
2182	90,40	177,00	28,855	95,00	27,60	31,340	4,36440	0,075902
2183	74,50	167,00	26,713	88,00	27,60	28,427	4,59610	0,076204
2184	76,00	163,00	28,605	91,00	27,60	30,431	5,10451	0,076206
2185	81,60	176,00	26,343	90,00	27,60	26,501	4,12564	0,076626
2186	93,50	179,00	29,181	99,00	27,60	30,247	4,47259	0,078068
2187	93,10	178,00	29,384	100,00	27,60	30,794	4,59761	0,078714
2188	84,40	169,00	29,551	98,00	27,60	31,288	5,11622	0,078868
2189	76,00	165,00	27,916	94,00	27,60	30,016	5,15671	0,079522
2190	86,00	173,00	28,735	99,00	27,60	31,199	4,88344	0,080231
2191	76,00	170,00	26,298	93,00	27,60	28,273	4,71411	0,080658
2192	73,60	164,00	27,365	94,00	27,60	28,443	5,23641	0,080831
2193	92,30	177,00	29,462	103,00	27,60	31,947	4,84547	0,081161
2194	90,40	185,00	26,413	98,00	27,60	26,354	4,04649	0,081238

2195	83,60	171,00	28,590	100,00	27,60	30,618	5,09472	0,081789
2196	87,30	173,00	29,169	102,00	27,60	31,509	5,07248	0,081839
2197	87,30	172,00	29,509	103,00	27,60	31,811	5,21125	0,082243
2198	93,60	182,00	28,257	104,00	27,60	28,950	4,56662	0,083095
2199	91,20	175,00	29,780	106,00	27,60	31,753	5,17278	0,083402
2200	96,00	185,00	28,050	105,00	27,60	29,320	4,43142	0,083622
2201	94,00	178,00	29,668	109,00	27,60	30,580	5,13315	0,085249
2202	96,70	182,00	29,193	112,00	27,60	30,262	5,02187	0,087564
2203	84,40	172,50	28,364	93,00	27,70	30,554	4,53964	0,076134
2204	92,10	180,00	28,426	97,00	27,70	30,524	4,29191	0,077623
2205	82,20	171,00	28,111	94,00	27,70	30,465	4,70783	0,077752
2206	84,00	170,00	29,066	97,00	27,70	30,495	4,97503	0,078697
2207	77,70	164,00	28,889	95,00	27,70	30,877	5,30658	0,078792
2208	86,30	172,00	29,171	99,00	27,70	30,627	4,95618	0,079659
2209	82,70	173,00	27,632	96,00	27,70	29,340	4,69450	0,079856
2210	87,80	175,00	28,669	99,00	27,70	29,896	4,74173	0,079892
2211	93,00	179,00	29,025	101,00	27,70	31,439	4,59016	0,079930
2212	77,00	170,00	26,644	93,00	27,70	28,221	4,71411	0,079958
2213	99,40	183,00	29,681	104,00	27,70	31,352	4,50217	0,080196
2214	80,00	170,00	27,682	96,00	27,70	28,740	4,90978	0,080461
2215	88,50	174,00	29,231	101,00	27,70	30,408	4,93652	0,080689
2216	80,80	170,00	27,958	97,00	27,70	29,312	4,97503	0,080762
2217	79,70	170,00	27,578	97,00	27,70	29,718	4,97503	0,081503
2218	81,00	177,00	25,855	95,00	27,70	26,083	4,36440	0,081667
2219	88,90	180,50	27,286	100,00	27,70	28,910	4,43408	0,082123
2220	91,60	178,00	28,910	104,00	27,70	30,803	4,83553	0,082753
2221	86,00	170,50	29,584	105,00	27,70	30,947	5,45728	0,084067
2222	82,40	168,00	29,195	105,00	27,70	31,735	5,66185	0,085440
2223	78,20	172,00	26,433	100,00	27,70	28,319	5,01993	0,085928
2224	73,10	165,00	26,850	102,00	27,70	28,950	5,71158	0,088557
2225	81,80	168,00	28,982	93,00	27,80	31,489	4,85938	0,076045
2226	85,40	173,00	28,534	96,00	27,80	30,758	4,69450	0,078164
2227	73,40	166,00	26,637	90,00	27,80	26,662	4,80473	0,078319
2228	91,50	175,00	29,878	100,00	27,80	32,149	4,80328	0,078509
2229	73,70	170,00	25,502	91,00	27,80	26,119	4,58371	0,080557
2230	69,70	160,50	27,057	92,00	27,80	29,201	5,38072	0,080574
2231	80,20	173,00	26,797	99,00	27,80	28,413	4,88344	0,084054
2232	85,00	172,00	28,732	105,00	27,80	30,586	5,33885	0,085346
2233	86,50	176,50	27,767	106,00	27,80	28,631	5,06225	0,087013
2234	88,00	175,00	28,735	111,00	27,80	31,301	5,48098	0,089440
2235	78,40	176,00	25,310	88,00	27,90	25,731	4,00418	0,076948
2236	80,80	168,00	28,628	95,00	27,90	30,562	4,99300	0,078320
2237	75,20	168,00	26,644	93,00	27,90	26,791	4,85938	0,080432
2238	74,50	163,50	27,869	95,00	27,90	29,090	5,34743	0,080826
2239	84,00	177,00	26,812	97,00	27,90	27,726	4,48460	0,081389
2240	82,00	172,00	27,718	98,00	27,90	28,017	4,89244	0,081588
2241	84,90	171,00	29,035	102,00	27,90	30,655	5,22378	0,082571
2242	84,10	168,00	29,797	103,00	27,90	31,213	5,52798	0,082679
2243	77,70	169,00	27,205	98,00	27,90	28,237	5,11622	0,083340
2244	82,50	168,50	29,057	103,00	27,90	30,584	5,48714	0,083953

2245	77,70	165,00	28,540	101,00	27,90	30,661	5,64218	0,084193
2246	90,80	181,00	27,716	104,00	27,90	28,234	4,63216	0,084406
2247	83,00	170,00	28,720	104,00	27,90	30,673	5,43212	0,085053
2248	82,50	175,50	26,785	101,00	27,90	28,281	4,82946	0,085163
2249	83,90	171,00	28,693	105,00	27,90	29,926	5,41745	0,085673
2250	76,00	169,00	26,610	101,00	27,90	28,656	5,31445	0,087167
2251	81,90	174,00	27,051	104,00	27,90	28,153	5,12342	0,087492
2252	96,40	185,00	28,167	111,00	27,90	30,105	4,76169	0,088155
2253	91,50	182,00	27,623	109,00	27,90	28,776	4,85108	0,088418
2254	72,20	165,00	26,520	105,00	27,90	28,065	5,91988	0,091918
2255	85,20	170,00	29,481	95,00	28,00	31,586	4,84455	0,076349
2256	83,20	172,50	27,961	93,00	28,00	28,882	4,53964	0,076864
2257	80,80	168,50	28,458	94,00	28,00	30,044	4,88897	0,077688
2258	74,20	159,00	29,350	94,00	28,00	31,140	5,65801	0,078347
2259	73,80	161,50	28,295	93,00	28,00	30,367	5,36986	0,078811
2260	93,20	177,00	29,749	101,00	28,00	31,435	4,72514	0,079072
2261	72,20	160,00	28,203	93,00	28,00	30,256	5,49670	0,079352
2262	92,60	180,00	28,580	101,00	28,00	30,092	4,52437	0,080533
2263	83,60	168,00	29,620	100,00	28,00	30,708	5,32727	0,080591
2264	93,00	184,00	27,469	100,00	28,00	29,474	4,21635	0,080977
2265	80,00	174,00	26,424	95,00	28,00	27,580	4,56301	0,081181
2266	91,10	182,00	27,503	102,00	28,00	29,180	4,45290	0,082981
2267	92,30	177,40	29,329	106,00	28,00	30,628	4,99730	0,083682
2268	81,80	175,00	26,710	104,00	28,00	27,711	5,04957	0,087982
2269	77,90	165,00	28,613	106,00	28,00	30,133	5,98934	0,088210
2270	96,80	181,00	29,547	96,00	28,10	32,047	4,17237	0,074660
2271	88,80	177,00	28,344	95,00	28,10	29,064	4,36440	0,076811
2272	86,30	174,00	28,504	95,00	28,10	29,790	4,56301	0,077180
2273	97,80	181,00	29,853	100,00	28,10	32,120	4,40219	0,077240
2274	90,80	178,00	28,658	98,00	28,10	29,984	4,47871	0,078437
2275	80,60	167,00	28,900	96,00	28,10	30,489	5,13691	0,078882
2276	92,30	178,00	29,131	100,00	28,10	29,887	4,59761	0,079168
2277	81,90	168,00	29,018	97,00	28,10	31,530	5,12667	0,079251
2278	90,10	177,00	28,759	99,00	28,10	29,807	4,60485	0,079274
2279	83,40	169,00	29,201	98,00	28,10	31,448	5,11622	0,079498
2280	99,00	185,00	28,926	102,00	28,10	30,421	4,26640	0,079583
2281	80,00	164,00	29,744	98,00	28,10	32,180	5,51719	0,079714
2282	79,80	166,00	28,959	97,00	28,10	30,562	5,28390	0,079835
2283	84,20	171,00	28,795	100,00	28,10	30,157	5,09472	0,081400
2284	89,10	173,00	29,770	103,00	28,10	31,742	5,13552	0,081525
2285	75,00	162,00	28,578	97,00	28,10	30,604	5,61621	0,081532
2286	82,20	176,00	26,537	97,00	28,10	27,365	4,55106	0,082183
2287	80,00	168,00	28,345	100,00	28,10	30,532	5,32727	0,082991
2288	72,10	169,00	25,244	94,00	28,10	26,962	4,85209	0,084025
2289	82,90	170,00	28,685	103,00	28,10	29,712	5,36678	0,084303
2290	86,00	174,00	28,405	104,00	28,10	30,500	5,12342	0,084689
2291	93,80	181,00	28,632	107,00	28,10	29,541	4,80474	0,084979
2292	74,90	164,00	27,848	100,00	28,10	29,716	5,65767	0,084993
2293	90,30	176,00	29,152	107,00	28,10	31,685	5,15969	0,085150
2294	81,70	166,00	29,649	107,00	28,10	32,164	5,96953	0,086695

2295	86,80	177,00	27,706	107,00	28,10	28,882	5,08625	0,087838
2296	79,10	169,00	27,695	92,00	28,20	28,537	4,72010	0,077311
2297	92,00	176,00	29,700	101,00	28,20	31,658	4,79439	0,079382
2298	87,40	176,00	28,215	98,00	28,20	29,741	4,61188	0,079704
2299	79,90	173,00	26,697	94,00	28,20	27,456	4,56860	0,080008
2300	88,00	177,00	28,089	100,00	28,20	30,332	4,66499	0,081343
2301	72,70	164,00	27,030	94,00	28,20	28,705	5,23641	0,081497
2302	88,60	179,00	27,652	100,00	28,20	29,918	4,53137	0,081738
2303	85,50	170,00	29,585	102,00	28,20	31,519	5,30146	0,081783
2304	84,00	176,00	27,118	98,00	28,20	29,275	4,61188	0,081840
2305	91,00	175,00	29,714	104,00	28,20	30,828	5,04957	0,081948
2306	78,20	169,00	27,380	97,00	28,20	29,366	5,05017	0,082137
2307	84,90	170,00	29,377	103,00	28,20	30,882	5,36678	0,082974
2308	82,30	170,00	28,478	101,00	28,20	29,964	5,23615	0,083067
2309	80,00	168,00	28,345	102,00	28,20	30,217	5,46107	0,084651
2310	93,50	181,00	28,540	108,00	28,20	29,629	4,86228	0,085957
2311	66,30	159,00	26,225	98,00	28,20	27,938	5,95710	0,088047
2312	87,70	178,00	27,680	92,00	28,30	28,296	4,12225	0,075360
2313	102,10	185,50	29,671	106,00	28,30	31,718	4,45505	0,081204
2314	92,10	185,00	26,910	103,00	28,30	26,685	4,32140	0,084328
2315	88,20	172,00	29,813	107,00	28,30	32,447	5,46649	0,084855
2316	72,30	166,00	26,237	97,00	28,30	27,708	5,28390	0,085265
2317	81,40	170,00	28,166	105,00	28,30	30,105	5,49746	0,086992
2318	92,50	177,50	29,359	98,00	28,40	31,054	4,51158	0,077291
2319	85,50	172,00	28,901	96,00	28,40	31,092	4,76500	0,077726
2320	87,60	172,00	29,611	98,00	28,40	31,171	4,89244	0,078072
2321	104,00	188,50	29,269	106,00	28,40	31,724	4,27191	0,081291
2322	78,70	169,00	27,555	97,00	28,40	29,468	5,05017	0,081789
2323	83,10	171,00	28,419	100,00	28,40	29,265	5,09472	0,082116
2324	94,00	178,00	29,668	105,00	28,40	30,580	4,89503	0,082121
2325	81,50	168,50	28,705	100,00	28,40	30,757	5,28764	0,082173
2326	91,00	178,00	28,721	104,00	28,40	30,166	4,83553	0,083117
2327	91,50	178,00	28,879	105,00	28,40	30,262	4,89503	0,083610
2328	91,10	178,00	28,753	107,00	28,40	30,814	5,01407	0,085452
2329	85,00	175,00	27,755	104,00	28,40	29,493	5,04957	0,085760
2330	85,00	175,00	27,755	105,00	28,40	29,602	5,11117	0,086585
2331	90,00	174,00	29,727	92,00	28,50	31,219	4,37640	0,072680
2332	83,10	170,00	28,754	91,00	28,50	30,816	4,58371	0,074362
2333	80,90	168,00	28,664	95,00	28,50	30,094	4,99300	0,078256
2334	89,30	175,00	29,159	99,00	28,50	29,727	4,74173	0,078995
2335	85,80	172,00	29,002	98,00	28,50	29,818	4,89244	0,079160
2336	91,00	176,00	29,378	100,00	28,50	31,850	4,73354	0,079171
2337	81,10	168,00	28,734	98,00	28,50	31,199	5,19353	0,080594
2338	89,30	176,00	28,829	101,00	28,50	30,098	4,79439	0,080974
2339	91,20	175,00	29,780	103,00	28,50	30,817	4,98798	0,081041
2340	88,00	175,00	28,735	101,00	28,50	30,996	4,86484	0,081383
2341	92,00	186,00	26,593	101,00	28,50	26,840	4,15186	0,083123
2342	88,30	176,00	28,506	103,00	28,50	30,828	4,91611	0,083200
2343	88,10	175,00	28,767	104,00	28,50	31,136	5,04957	0,083736
2344	86,80	176,00	28,022	103,00	28,50	30,144	4,91611	0,084156

2345	94,80	181,00	28,937	107,00	28,50	30,434	4,80474	0,084381
2346	76,40	168,00	27,069	99,00	28,50	28,523	5,26039	0,084722
2347	83,40	174,00	27,547	103,00	28,50	29,791	5,06110	0,085609
2348	79,70	166,00	28,923	104,00	28,50	31,419	5,76370	0,085668
2349	88,50	177,00	28,249	107,00	28,50	29,994	5,08625	0,086709
2350	72,40	164,00	26,919	100,00	28,50	28,215	5,65767	0,086938
2351	85,20	169,00	29,831	96,00	28,60	31,068	4,98414	0,076775
2352	75,10	164,00	27,922	91,00	28,60	29,699	5,02596	0,077206
2353	83,10	172,00	28,090	95,00	28,60	29,798	4,70129	0,078390
2354	91,50	175,00	29,878	100,00	28,60	31,498	4,80328	0,078509
2355	93,50	179,00	29,181	102,00	28,60	30,934	4,64896	0,080433
2356	75,40	167,00	27,036	94,00	28,60	28,712	5,00163	0,080750
2357	85,50	181,00	26,098	97,00	28,60	25,918	4,22981	0,081946
2358	92,30	181,00	28,174	105,00	28,60	30,327	4,68968	0,084292
2359	68,80	159,00	27,214	97,00	28,60	29,163	5,88230	0,085024
2360	81,00	166,00	29,395	106,00	28,60	31,387	5,90091	0,086379
2361	88,30	174,00	29,165	109,00	28,60	31,406	5,43513	0,087212
2362	78,00	162,00	29,721	90,00	28,70	31,589	5,11264	0,073696
2363	90,10	178,50	28,278	96,00	28,70	30,030	4,32794	0,077414
2364	85,80	174,00	28,339	96,00	28,70	28,952	4,62523	0,078296
2365	72,30	164,00	26,881	91,00	28,70	27,465	5,02596	0,079187
2366	80,30	170,00	27,785	95,00	28,70	29,311	4,84455	0,079424
2367	84,50	171,50	28,730	98,00	28,70	30,074	4,92892	0,079777
2368	72,00	163,00	27,099	92,00	28,70	28,791	5,17553	0,079871
2369	75,00	172,00	25,352	93,00	28,70	27,097	4,57392	0,082170
2370	83,90	170,00	29,031	102,00	28,70	30,552	5,30146	0,082820
2371	87,40	177,00	27,897	102,00	28,70	29,235	4,78530	0,083350
2372	80,00	170,00	27,682	102,00	28,70	29,402	5,30146	0,085490
2373	82,30	172,00	27,819	107,00	28,70	28,590	5,46649	0,088864
2374	77,90	173,50	25,878	91,00	28,80	27,755	4,34688	0,078965
2375	91,00	178,00	28,721	99,00	28,80	29,962	4,53816	0,079121
2376	93,60	180,00	28,889	100,00	28,80	30,073	4,46624	0,079167
2377	84,30	175,00	27,527	96,00	28,80	28,651	4,55716	0,079601
2378	89,60	173,00	29,938	101,00	28,80	31,757	5,00946	0,079644
2379	94,20	181,00	28,754	101,00	28,80	29,597	4,45967	0,079987
2380	82,40	169,00	28,851	99,00	28,80	30,327	5,18229	0,080957
2381	93,80	177,00	29,940	104,00	28,80	31,390	4,90565	0,081073
2382	82,50	169,00	28,886	101,00	28,80	30,069	5,31445	0,082526
2383	91,70	176,00	29,604	106,00	28,80	30,687	5,09878	0,083494
2384	76,80	166,00	27,871	99,00	28,80	28,657	5,42092	0,083589
2385	84,00	173,00	28,066	104,00	28,80	30,091	5,19856	0,085615
2386	88,10	173,00	29,436	95,00	28,90	30,859	4,63155	0,075761
2387	88,20	177,00	28,153	94,00	28,90	29,344	4,30431	0,076347
2388	86,00	174,00	28,405	95,00	28,90	29,248	4,56301	0,077360
2389	84,90	170,00	29,377	99,00	28,90	31,753	5,10557	0,079751
2390	75,60	166,00	27,435	95,00	28,90	28,197	5,14693	0,081059
2391	78,60	165,50	28,696	98,00	28,90	30,339	5,39303	0,081272
2392	86,00	175,00	28,082	100,00	28,90	30,002	4,80328	0,081821
2393	85,80	177,00	27,387	99,00	28,90	28,697	4,60485	0,081901
2394	82,00	175,00	26,776	97,00	28,90	28,032	4,61867	0,081927

2395	77,90	170,00	26,955	97,00	28,90	27,912	4,97503	0,082754
2396	83,80	173,00	28,000	101,00	28,90	29,687	5,00946	0,083278
2397	82,60	173,00	27,599	101,00	28,90	29,077	5,00946	0,084083
2398	81,60	173,00	27,265	101,00	28,90	28,200	5,00946	0,084768
2399	83,40	172,00	28,191	109,00	28,90	30,029	5,59419	0,089727
2400	96,70	184,00	28,562	97,00	29,00	29,348	4,04963	0,076531
2401	79,10	163,00	29,772	94,00	29,00	31,743	5,31760	0,076648
2402	94,20	179,00	29,400	98,00	29,00	30,428	4,41381	0,076896
2403	65,00	158,00	26,037	88,00	29,00	27,329	5,29328	0,079693
2404	77,90	165,50	28,441	96,00	29,00	29,710	5,25519	0,080090
2405	76,80	169,00	26,890	94,00	29,00	27,359	4,85209	0,080561
2406	73,80	161,00	28,471	96,00	29,00	29,748	5,63034	0,081144
2407	91,50	176,00	29,539	103,00	29,00	31,083	4,91611	0,081249
2408	84,10	176,00	27,150	98,00	29,00	27,706	4,61188	0,081775
2409	83,10	175,00	27,135	98,00	29,00	27,801	4,68020	0,082040
2410	92,00	177,00	29,366	104,00	29,00	31,739	4,90565	0,082127
2411	94,10	180,00	29,043	105,00	29,00	31,162	4,75698	0,082830
2412	91,20	178,00	28,784	104,00	29,00	31,156	4,83553	0,082995
2413	87,80	175,00	28,669	103,00	29,00	29,283	4,98798	0,083120
2414	88,30	180,00	27,253	101,00	29,00	27,616	4,52437	0,083127
2415	96,00	182,00	28,982	107,00	29,00	31,089	4,73727	0,084061
2416	81,90	169,00	28,675	103,00	29,00	30,926	5,44666	0,084571
2417	82,20	170,00	28,443	104,00	29,00	29,921	5,43212	0,085604
2418	77,30	166,00	28,052	102,00	29,00	29,751	5,62655	0,085751
2419	81,00	172,00	27,380	104,00	29,00	29,591	5,27504	0,087294
2420	90,00	177,00	28,727	109,00	29,00	30,886	5,20670	0,087346
2421	62,30	149,00	28,062	102,00	29,00	29,335	7,31739	0,090489
2422	91,40	178,00	28,847	97,00	29,10	30,726	4,41928	0,077296
2423	94,00	181,00	28,693	99,00	29,10	29,620	4,34472	0,078514
2424	85,90	170,00	29,723	99,00	29,10	32,062	5,10557	0,079131
2425	81,20	172,50	27,288	96,00	29,10	28,685	4,72960	0,080641
2426	77,30	165,00	28,393	97,00	29,10	30,067	5,36469	0,081138
2427	81,30	169,50	28,298	99,00	29,10	30,686	5,14376	0,081887
2428	87,00	174,50	28,571	102,00	29,10	29,875	4,96245	0,082620
2429	83,40	173,00	27,866	101,00	29,10	29,738	5,00946	0,083544
2430	77,50	169,00	27,135	99,00	29,10	28,836	5,18229	0,084335
2431	77,50	162,00	29,531	103,00	29,10	32,123	6,04841	0,084703
2432	89,70	175,00	29,290	107,00	29,10	31,066	5,23439	0,085124
2433	80,70	166,00	29,286	105,00	29,10	30,867	5,83230	0,085776
2434	75,60	164,00	28,108	92,00	29,20	29,074	5,09610	0,077710
2435	91,50	176,00	29,539	99,00	29,20	31,083	4,67270	0,078094
2436	95,10	180,00	29,352	100,00	29,20	31,917	4,46624	0,078332
2437	90,50	180,00	27,932	99,00	29,20	29,495	4,40812	0,080155
2438	81,10	172,00	27,413	97,00	29,20	29,294	4,82871	0,081351
2439	82,00	170,00	28,374	99,00	29,20	30,148	5,10557	0,081621
2440	98,30	186,00	28,414	104,00	29,20	29,676	4,31506	0,081895
2441	78,40	164,00	29,149	100,00	29,20	31,387	5,65767	0,082444
2442	89,10	176,00	28,764	103,00	29,20	30,727	4,91611	0,082701
2443	92,20	177,00	29,430	105,00	29,20	30,947	4,96584	0,082797
2444	79,00	169,00	27,660	99,00	29,20	29,265	5,18229	0,083264

2445	96,00	180,00	29,630	107,00	29,20	30,720	4,87334	0,083291
2446	90,00	174,00	29,727	106,00	29,20	31,689	5,24807	0,083740
2447	91,90	182,00	27,744	104,00	29,20	29,150	4,56662	0,084117
2448	86,50	172,00	29,239	105,00	29,20	31,200	5,33885	0,084357
2449	85,80	173,50	28,503	104,00	29,20	30,824	5,16082	0,084617
2450	85,90	174,00	28,372	106,00	29,20	30,355	5,24807	0,086384
2451	91,30	175,00	29,812	110,00	29,20	31,792	5,41932	0,086486
2452	81,80	173,00	27,331	94,00	29,30	29,419	4,56860	0,078765
2453	81,80	166,00	29,685	98,00	29,30	32,206	5,35240	0,079338
2454	80,40	169,00	28,150	96,00	29,30	29,979	4,98414	0,079801
2455	86,70	174,00	28,637	99,00	29,30	29,957	4,81197	0,080183
2456	90,00	174,00	29,727	102,00	29,30	31,125	4,99881	0,080580
2457	86,30	178,00	27,238	98,00	29,30	28,507	4,47871	0,081140
2458	76,60	162,00	29,188	98,00	29,30	31,531	5,68821	0,081221
2459	82,00	174,00	27,084	97,00	29,30	29,118	4,68747	0,081537
2460	83,50	176,00	26,956	98,00	29,30	28,962	4,61188	0,082167
2461	78,70	164,00	29,261	100,00	29,30	31,519	5,65767	0,082234
2462	78,80	172,00	26,636	97,00	29,30	27,973	4,82871	0,082927
2463	89,60	180,00	27,654	104,00	29,30	28,926	4,69881	0,084766
2464	87,50	172,00	29,577	107,00	29,30	31,605	5,46649	0,085307
2465	75,90	167,00	27,215	100,00	29,30	28,136	5,40762	0,085527
2466	84,00	172,00	28,394	109,00	29,30	28,919	5,59419	0,089299
2467	94,80	178,00	29,920	102,00	29,40	32,106	4,71655	0,079325
2468	92,70	176,00	29,926	103,00	29,40	32,576	4,91611	0,080546
2469	82,00	170,00	28,374	98,00	29,40	30,671	5,04030	0,080796
2470	83,20	169,00	29,131	100,00	29,40	30,773	5,24836	0,081250
2471	91,50	175,00	29,878	104,00	29,40	31,406	5,04957	0,081649
2472	93,50	179,00	29,181	104,00	29,40	30,443	4,76659	0,082011
2473	86,60	178,00	27,332	100,00	29,40	28,741	4,59761	0,082605
2474	82,00	170,00	28,374	101,00	29,40	29,416	5,23615	0,083270
2475	91,20	175,00	29,780	106,00	29,40	31,659	5,17278	0,083402
2476	90,00	174,00	29,727	106,00	29,40	31,501	5,24807	0,083740
2477	88,80	176,00	28,667	106,00	29,40	29,894	5,09878	0,085302
2478	90,70	176,00	29,281	110,00	29,40	30,178	5,34247	0,087280
2479	83,90	172,00	28,360	98,00	29,50	29,922	4,89244	0,080351
2480	87,70	175,00	28,637	101,00	29,50	30,367	4,86484	0,081568
2481	86,10	176,50	27,638	100,00	29,50	29,016	4,69912	0,082341
2482	91,50	184,00	27,026	101,00	29,50	28,584	4,27195	0,082678
2483	79,30	166,50	28,605	100,00	29,50	30,637	5,44835	0,082857
2484	79,00	170,50	27,176	99,00	29,50	28,542	5,06772	0,083879
2485	83,60	171,00	28,590	103,00	29,50	31,030	5,28833	0,084242
2486	77,50	170,50	26,660	99,00	29,50	27,765	5,06772	0,084958
2487	76,00	166,00	27,580	100,00	29,50	29,054	5,48945	0,085025
2488	77,20	166,00	28,016	102,00	29,50	29,814	5,62655	0,085825
2489	83,20	174,00	27,481	104,00	29,50	29,040	5,12342	0,086578
2490	95,00	182,00	28,680	94,00	29,60	30,829	3,99837	0,074366
2491	99,00	186,00	28,616	96,00	29,60	30,239	3,88003	0,075239
2492	87,00	173,00	29,069	95,00	29,60	30,697	4,63155	0,076398
2493	90,50	176,50	29,051	96,00	29,60	30,774	4,45723	0,076464
2494	77,00	166,50	27,776	94,00	29,60	29,956	5,03987	0,079429

2495	95,30	180,00	29,414	104,00	29,60	30,253	4,69881	0,081351
2496	83,60	170,00	28,927	102,00	29,60	30,022	5,30146	0,083018
2497	87,80	174,00	29,000	104,00	29,60	31,210	5,12342	0,083527
2498	81,00	171,00	27,701	100,00	29,60	28,434	5,09472	0,083530
2499	82,40	168,00	29,195	90,00	29,70	31,637	4,65905	0,073234
2500	81,10	167,50	28,906	96,00	29,70	30,798	5,09820	0,078753
2501	88,10	178,60	27,619	98,00	29,70	29,214	4,43964	0,080256
2502	75,00	162,50	28,402	96,00	29,70	30,809	5,50178	0,080899
2503	82,50	168,00	29,230	100,00	29,70	30,407	5,32727	0,081306
2504	79,40	169,40	27,669	97,00	29,70	29,938	5,01996	0,081468
2505	91,40	175,00	29,845	104,00	29,70	31,272	5,04957	0,081709
2506	87,20	176,00	28,151	102,00	29,70	28,916	4,85524	0,083084
2507	87,00	175,50	28,247	107,00	29,70	29,569	5,19688	0,087083
2508	93,90	178,00	29,636	93,00	29,80	31,487	4,18163	0,072787
2509	88,00	173,00	29,403	99,00	29,80	30,432	4,88344	0,079011
2510	80,30	165,00	29,495	99,00	29,80	31,220	5,50340	0,080735
2511	82,60	169,00	28,921	101,00	29,80	31,016	5,31445	0,082459
2512	97,20	182,00	29,344	106,00	29,80	30,260	4,68038	0,082589
2513	84,30	176,00	27,215	100,00	29,80	29,163	4,73354	0,083312
2514	97,70	181,00	29,822	110,00	29,80	30,871	4,97739	0,085021
2515	80,00	171,00	27,359	101,00	29,80	29,453	5,15925	0,085066
2516	88,80	177,00	28,344	94,00	29,90	30,426	4,30431	0,076003
2517	83,20	168,00	29,478	97,00	29,90	31,104	5,12667	0,078424
2518	76,00	167,00	27,251	94,00	29,90	27,727	5,00163	0,080325
2519	92,90	178,00	29,321	102,00	29,90	30,036	4,71655	0,080403
2520	91,30	176,50	29,308	102,00	29,90	31,477	4,82012	0,080768
2521	79,20	170,50	27,244	97,00	29,90	27,718	4,93797	0,082046
2522	90,60	179,00	28,276	102,00	29,90	30,133	4,64896	0,082141
2523	90,20	174,50	29,622	104,00	29,90	31,185	5,08633	0,082235
2524	90,50	177,50	28,724	103,00	29,90	29,966	4,81061	0,082427
2525	88,60	172,00	29,949	105,00	29,90	31,585	5,33885	0,083018
2526	83,40	174,00	27,547	100,00	29,90	29,457	4,87424	0,083115
2527	84,70	171,00	28,966	103,00	29,90	30,771	5,28833	0,083511
2528	80,40	170,00	27,820	101,00	29,90	29,355	5,23615	0,084371
2529	74,30	165,00	27,291	99,00	29,90	29,370	5,50340	0,085025
2530	87,40	171,00	29,890	99,00	30,00	31,884	5,03021	0,078606
2531	88,20	175,00	28,800	98,00	30,00	30,163	4,68020	0,078846
2532	84,70	171,00	28,966	98,00	30,00	30,271	4,96571	0,079457
2533	85,80	173,00	28,668	100,00	30,00	30,814	4,94644	0,081167
2534	88,30	175,00	28,833	101,00	30,00	30,305	4,86484	0,081198
2535	87,00	175,00	28,408	101,00	30,00	29,982	4,86484	0,082005
2536	89,00	180,00	27,469	102,00	30,00	28,354	4,58250	0,083509
2537	89,50	177,00	28,568	104,00	30,00	29,767	4,90565	0,083649
2538	87,80	179,00	27,402	104,00	30,00	29,168	4,76659	0,085523
2539	85,90	175,00	28,049	105,00	30,00	30,284	5,11117	0,085979
2540	76,90	170,00	26,609	106,00	30,00	28,177	5,56282	0,091214
2541	94,40	181,00	28,815	97,00	30,10	31,192	4,22981	0,076711
2542	76,70	161,00	29,590	95,00	30,10	31,621	5,55746	0,078262
2543	85,60	172,00	28,935	103,00	30,10	30,632	5,21125	0,083329
2544	87,80	174,00	29,000	104,00	30,10	31,509	5,12342	0,083527

2545	100,00	185,00	29,218	110,00	30,10	30,979	4,70663	0,085252
2546	81,00	171,00	27,701	106,00	30,10	29,646	5,48203	0,088541
2547	84,10	171,50	28,594	94,00	30,20	30,931	4,67256	0,076763
2548	79,10	166,00	28,705	93,00	30,20	30,859	5,01001	0,076994
2549	78,10	163,00	29,395	97,00	30,20	31,388	5,53081	0,079768
2550	95,00	178,00	29,984	104,00	30,20	32,549	4,83553	0,080767
2551	87,30	179,00	27,246	98,00	30,20	28,974	4,41381	0,080896
2552	84,00	168,00	29,762	101,00	30,20	31,638	5,39416	0,081138
2553	69,00	164,00	25,654	91,00	30,20	26,323	5,02596	0,081692
2554	79,10	166,00	28,705	99,00	30,20	30,757	5,42092	0,081961
2555	82,20	166,00	29,830	103,00	30,20	32,093	5,69512	0,083115
2556	77,50	163,00	29,169	101,00	30,20	31,214	5,81528	0,083485
2557	83,20	168,00	29,478	104,00	30,20	30,624	5,59491	0,084083
2558	81,00	170,00	28,028	103,00	30,20	29,184	5,36678	0,085616
2559	80,00	171,50	27,200	103,00	30,20	29,373	5,24962	0,086962
2560	88,00	173,00	29,403	109,00	30,20	31,879	5,51396	0,086991
2561	78,10	164,50	28,862	90,00	30,30	30,643	4,91754	0,074578
2562	78,70	175,00	25,698	88,00	30,30	26,636	4,06542	0,076389
2563	93,30	180,00	28,796	100,00	30,30	30,968	4,46624	0,079336
2564	93,40	177,50	29,645	103,00	30,30	30,645	4,81061	0,080712
2565	89,00	174,00	29,396	102,00	30,30	30,713	4,99881	0,081183
2566	77,00	164,00	28,629	100,00	30,30	30,563	5,65767	0,083440
2567	74,00	165,00	27,181	97,00	30,30	28,549	5,36469	0,083532
2568	87,10	173,00	29,102	104,00	30,30	31,331	5,19856	0,083572
2569	77,90	162,00	29,683	102,00	30,30	32,203	5,97634	0,083593
2570	103,00	186,00	29,772	110,00	30,30	30,807	4,64168	0,083964
2571	84,70	173,00	28,300	105,00	30,30	30,163	5,26162	0,085962
2572	79,00	168,00	27,990	103,00	30,30	30,322	5,52798	0,086201
2573	89,30	174,00	29,495	109,00	30,30	31,316	5,43513	0,086560
2574	87,60	178,00	27,648	106,00	30,30	29,360	4,95455	0,086893
2575	95,60	185,00	27,933	114,00	30,30	29,387	4,92694	0,091042
2576	80,80	172,00	27,312	93,00	30,40	28,262	4,57392	0,078190
2577	82,30	171,00	28,145	96,00	30,40	29,760	4,83675	0,079342
2578	75,50	165,00	27,732	96,00	30,40	30,013	5,29535	0,081572
2579	89,60	175,00	29,257	103,00	30,40	30,929	4,98798	0,082003
2580	87,00	176,00	28,086	101,00	30,40	28,725	4,79439	0,082395
2581	87,80	173,00	29,336	104,00	30,40	31,414	5,19856	0,083127
2582	80,90	171,00	27,667	100,00	30,40	29,273	5,09472	0,083598
2583	83,60	172,00	28,259	102,00	30,40	29,901	5,14746	0,083831
2584	98,70	186,00	28,529	107,00	30,40	30,752	4,47833	0,084030
2585	84,00	168,00	29,762	107,00	30,40	32,294	5,79577	0,085958
2586	83,40	170,50	28,689	98,00	30,50	30,432	5,00284	0,080085
2587	89,90	179,00	28,058	99,00	30,50	28,901	4,47259	0,080138
2588	87,80	175,00	28,669	105,00	30,50	30,407	5,11117	0,084734
2589	91,30	176,00	29,474	108,00	30,50	31,962	5,22060	0,085317
2590	78,10	163,00	29,395	104,00	30,50	31,774	6,02879	0,085524
2591	83,00	170,00	28,720	112,00	30,50	30,775	5,95521	0,091595
2592	83,20	169,00	29,131	94,00	30,60	30,872	4,85209	0,076375
2593	73,90	164,00	27,476	92,00	30,60	28,699	5,09610	0,078897
2594	83,10	168,00	29,443	100,00	30,60	31,541	5,32727	0,080914

2595	83,30	169,50	28,994	100,00	30,60	31,502	5,20944	0,081385
2596	83,00	172,00	28,056	104,00	30,60	28,899	5,27504	0,085886
2597	91,50	180,00	28,241	95,00	30,70	29,139	4,17573	0,076355
2598	76,10	163,00	28,642	93,00	30,70	30,579	5,24655	0,077813
2599	79,60	163,50	29,777	98,00	30,70	32,218	5,55935	0,079778
2600	93,00	177,00	29,685	105,00	30,70	31,545	4,96584	0,082321
2601	88,80	181,00	27,105	100,00	30,70	28,108	4,40219	0,082374
2602	84,90	171,00	29,035	114,00	30,70	31,549	5,99909	0,092285
2603	89,50	176,00	28,893	104,00	30,80	30,380	4,97699	0,083255
2604	76,70	163,00	28,868	101,00	30,80	31,054	5,81528	0,084065
2605	90,00	182,00	27,171	104,00	30,80	28,651	4,56662	0,085296
2606	96,60	181,00	29,486	114,00	30,80	31,593	5,20773	0,088781
2607	78,20	165,00	28,724	91,00	30,90	31,186	4,94885	0,075534
2608	93,20	178,00	29,415	107,00	30,90	30,641	5,01407	0,084163
2609	87,10	174,00	28,769	105,00	30,90	30,935	5,18574	0,084782
2610	90,00	175,00	29,388	107,00	30,90	30,606	5,23439	0,084935
2611	86,50	171,00	29,582	107,00	30,90	30,945	5,54662	0,085547
2612	79,00	173,00	26,396	100,00	30,90	28,272	4,94644	0,085760
2613	90,00	173,50	29,898	97,00	31,00	30,874	4,72232	0,076447
2614	93,00	177,00	29,685	106,00	31,00	31,357	5,02604	0,083105
2615	84,40	176,50	27,093	100,00	31,00	29,014	4,69912	0,083443
2616	87,60	171,00	29,958	107,00	31,00	31,504	5,54662	0,084829
2617	100,60	183,50	29,876	111,00	31,00	31,683	4,86205	0,085105
2618	86,00	172,00	29,070	106,00	31,00	30,897	5,40267	0,085490
2619	82,60	167,00	29,617	108,00	31,00	31,843	5,94966	0,087304
2620	86,90	180,00	26,821	105,00	31,00	27,738	4,75698	0,087345
2621	88,70	176,00	28,635	95,00	31,10	30,980	4,42946	0,076507
2622	90,00	180,00	27,778	97,00	31,10	29,302	4,29191	0,078826
2623	86,60	171,50	29,444	103,00	31,10	30,868	5,24962	0,082486
2624	75,30	165,00	27,658	97,00	31,10	29,815	5,36469	0,082568
2625	95,10	182,00	28,710	107,00	31,10	30,865	4,73727	0,084591
2626	80,60	167,00	28,900	103,00	31,10	30,791	5,61079	0,084634
2627	97,10	185,00	28,371	109,00	31,10	30,040	4,65157	0,086150
2628	91,50	176,00	29,539	110,00	31,10	30,987	5,34247	0,086771
2629	76,70	167,00	27,502	104,00	31,10	29,402	5,67854	0,088328
2630	85,80	173,00	28,668	91,00	31,20	29,894	4,37982	0,073862
2631	94,00	179,00	29,337	100,00	31,20	31,415	4,53137	0,078576
2632	94,30	179,00	29,431	102,00	31,20	31,816	4,64896	0,079978
2633	91,00	177,00	29,047	103,00	31,20	29,876	4,84547	0,081932
2634	83,30	168,00	29,514	105,00	31,20	31,625	5,66185	0,084824
2635	87,30	178,00	27,553	105,00	31,20	29,465	4,89503	0,086271
2636	86,10	173,00	28,768	107,00	31,20	30,731	5,38777	0,086647
2637	81,40	170,00	28,166	105,00	31,20	30,211	5,49746	0,086992
2638	71,00	162,00	27,054	100,00	31,20	28,272	5,83224	0,087181
2639	85,70	173,00	28,634	90,00	31,30	29,545	4,31692	0,073107
2640	80,40	166,00	29,177	94,00	31,30	31,223	5,07846	0,076980
2641	94,50	178,00	29,826	105,00	31,30	31,528	4,89503	0,081831
2642	110,00	194,00	29,227	110,00	31,30	31,186	4,15811	0,083234
2643	92,70	177,00	29,589	107,00	31,30	31,715	5,08625	0,084070
2644	99,00	185,00	28,926	110,00	31,30	30,121	4,70663	0,085825

2645	86,80	173,00	29,002	109,00	31,30	30,615	5,51396	0,087791
2646	115,10	196,00	29,961	105,00	31,40	31,786	3,80172	0,077747
2647	78,10	174,00	25,796	100,00	31,40	27,652	4,87424	0,086834
2648	84,40	170,00	29,204	109,00	31,40	31,746	5,75896	0,088153
2649	90,60	175,00	29,584	100,00	31,50	30,282	4,80328	0,079028
2650	89,90	178,00	28,374	100,00	31,50	29,311	4,59761	0,080571
2651	84,00	170,00	29,066	100,00	31,50	31,585	5,17086	0,081131
2652	80,00	169,00	28,010	100,00	31,50	30,453	5,24836	0,083402
2653	74,90	159,00	29,627	101,00	31,50	32,234	6,18157	0,083656
2654	92,90	178,00	29,321	110,00	31,50	31,687	5,19270	0,086709
2655	86,60	184,00	25,579	108,00	31,50	26,995	4,66132	0,091712
2656	80,60	166,00	29,250	96,00	31,60	31,506	5,21540	0,078488
2657	72,80	162,00	27,740	92,00	31,60	28,925	5,25645	0,078879
2658	85,30	171,00	29,171	99,00	31,60	29,841	5,03021	0,079891
2659	81,10	165,00	29,789	102,00	31,60	31,670	5,71158	0,082634
2660	85,30	176,00	27,537	100,00	31,60	29,000	4,73354	0,082660
2661	82,20	169,00	28,781	103,00	31,60	30,645	5,44666	0,084365
2662	88,90	173,00	29,704	107,00	31,60	32,227	5,38777	0,084818
2663	98,00	181,00	29,914	106,00	31,70	31,913	4,74720	0,081762
2664	81,70	172,00	27,616	101,00	31,70	29,543	5,08369	0,084291
2665	87,80	173,00	29,336	113,00	31,70	31,802	5,76648	0,090321
2666	85,80	172,00	29,002	96,00	31,80	31,412	4,76500	0,077545
2667	86,60	172,00	29,273	99,00	31,80	31,533	4,95618	0,079475
2668	88,50	172,50	29,742	103,00	31,80	32,083	5,17322	0,081696
2669	89,70	174,00	29,627	104,00	31,80	30,812	5,12342	0,082344
2670	91,00	177,00	29,047	106,00	31,80	30,174	5,02604	0,084318
2671	76,70	166,00	27,834	101,00	31,80	30,136	5,55799	0,085352
2672	105,60	188,00	29,878	113,00	31,80	32,149	4,67482	0,085592
2673	87,00	173,00	29,069	103,00	31,90	30,995	5,13552	0,082832
2674	74,70	165,00	27,438	95,00	32,00	29,549	5,22602	0,081298
2675	85,30	170,00	29,516	102,00	32,00	30,958	5,30146	0,081911
2676	76,00	164,00	28,257	98,00	32,00	30,110	5,51719	0,082487
2677	87,80	172,00	29,678	103,00	32,10	31,537	5,21125	0,081931
2678	87,00	171,00	29,753	107,00	32,10	31,533	5,54662	0,085219
2679	92,20	178,00	29,100	108,00	32,10	30,044	5,07360	0,085563
2680	89,60	173,00	29,938	109,00	32,10	32,034	5,51396	0,085953
2681	85,60	173,50	28,436	99,00	32,20	30,225	4,84755	0,080674
2682	88,70	174,00	29,297	104,00	32,20	31,173	5,12342	0,082961
2683	81,60	167,00	29,259	90,00	32,30	31,321	4,73123	0,073347
2684	88,60	181,00	27,044	94,00	32,30	29,186	4,05752	0,077548
2685	83,30	170,00	28,824	97,00	32,30	30,899	4,97503	0,079138
2686	90,00	176,00	29,055	105,00	32,30	31,573	5,03788	0,083744
2687	80,70	167,00	28,936	100,00	32,40	31,335	5,40762	0,082101
2688	86,70	172,00	29,306	109,00	32,40	31,864	5,59419	0,087435
2689	82,00	168,00	29,053	97,00	32,50	30,579	5,12667	0,079187
2690	94,70	179,00	29,556	102,00	32,50	31,294	4,64896	0,079753
2691	84,00	169,00	29,411	100,00	32,50	31,696	5,24836	0,080733
2692	79,70	167,00	28,578	102,00	32,50	30,501	5,54305	0,084442
2693	75,60	166,00	27,435	102,00	32,50	29,657	5,62655	0,087031
2694	88,90	174,00	29,363	104,00	32,60	30,865	5,12342	0,082837

2695	91,00	174,50	29,885	104,00	32,70	32,529	5,08633	0,081753
2696	83,90	169,00	29,376	105,00	32,70	31,558	5,57892	0,084837
2697	81,40	170,00	28,166	96,00	32,80	28,830	4,90978	0,079536
2698	97,00	180,00	29,938	107,00	32,80	31,758	4,87334	0,082717
2699	83,20	174,00	27,481	105,00	32,80	29,152	5,18574	0,087411
2700	83,90	170,00	29,031	102,00	32,90	31,247	5,30146	0,082820
2701	77,00	161,00	29,706	101,00	32,90	32,041	5,99497	0,082988
2702	84,10	172,00	28,428	106,00	32,90	30,735	5,40267	0,086773
2703	88,80	176,00	28,667	113,00	33,00	31,121	5,52536	0,090935
2704	68,60	156,00	28,189	95,00	33,10	30,239	6,00864	0,082119
2705	99,80	186,00	28,847	108,00	33,10	29,718	4,53277	0,084191
2706	81,70	175,00	26,678	100,00	33,10	28,501	4,80328	0,084668
2707	67,10	163,00	25,255	95,00	33,10	26,316	5,38865	0,086443
2708	76,40	167,00	27,394	96,00	33,20	29,496	5,13691	0,081747
2709	97,10	184,00	28,680	104,00	33,20	30,012	4,43877	0,081828
2710	104,00	187,00	29,741	108,00	33,20	31,894	4,47006	0,082275
2711	85,10	171,00	29,103	100,00	33,30	31,332	5,09472	0,080825
2712	91,70	178,00	28,942	103,00	33,30	31,442	4,77603	0,081898
2713	86,10	171,00	29,445	105,00	33,40	31,447	5,41745	0,084207
2714	92,90	178,00	29,321	107,00	33,50	31,007	5,01407	0,084344
2715	78,00	173,00	26,062	95,00	33,60	26,862	4,63155	0,082167
2716	86,50	172,00	29,239	106,00	33,60	31,004	5,40267	0,085160
2717	88,70	174,50	29,129	101,00	33,90	31,561	4,90052	0,080761
2718	81,50	172,00	27,549	97,00	33,90	29,014	4,82871	0,081085
2719	92,50	178,00	29,195	105,00	33,90	31,735	4,89503	0,083006
2720	88,70	177,00	28,312	106,00	33,90	30,808	5,02604	0,085770
2721	71,00	168,00	25,156	89,00	34,00	24,328	4,59229	0,079979
2722	88,40	173,00	29,537	109,00	34,00	31,843	5,51396	0,086729
2723	72,00	169,00	25,209	97,00	34,00	26,918	5,05017	0,086787
2724	76,30	160,00	29,805	97,00	34,10	31,783	5,79192	0,079773
2725	92,70	178,00	29,258	108,00	34,10	30,930	5,07360	0,085255
2726	80,20	170,00	27,751	108,00	34,10	29,707	5,69357	0,090368
2727	82,60	175,00	26,971	93,00	34,30	28,981	4,37268	0,078168
2728	92,00	180,00	28,395	105,00	34,30	30,592	4,75698	0,084086
2729	90,70	174,50	29,786	93,00	34,40	31,667	4,40549	0,073267
2730	81,20	171,00	27,769	109,00	34,40	29,291	5,67584	0,090898
2731	80,00	165,00	29,385	109,00	34,70	31,375	6,19781	0,089112
2732	74,00	171,00	25,307	90,00	35,00	26,909	4,45014	0,079846
2733	78,20	170,00	27,059	93,00	35,40	28,625	4,71411	0,079138
2734	70,00	165,00	25,712	98,00	35,40	27,420	5,43404	0,087578
2735	96,00	182,00	28,982	99,00	35,60	29,892	4,28239	0,077776
2736	86,10	172,00	29,104	99,00	35,60	31,531	4,95618	0,079782
2737	78,40	177,00	25,025	89,00	35,70	26,551	4,00401	0,078191
2738	71,00	168,00	25,156	91,00	35,70	24,594	4,72581	0,081776
2739	80,90	167,00	29,008	106,00	35,70	30,821	5,81407	0,086883
2740	80,70	173,00	26,964	95,00	35,80	27,691	4,63155	0,080324
2741	102,00	185,00	29,803	112,00	35,90	32,061	4,81677	0,085663
2742	85,70	173,00	28,634	93,00	36,00	29,647	4,50566	0,075544
2743	78,00	172,00	26,366	91,00	36,00	26,653	4,44659	0,078328
2744	75,00	168,00	26,573	91,00	36,00	28,611	4,72581	0,078842

2745	90,00	174,00	29,727	102,00	36,00	32,160	4,99881	0,080580
2746	79,00	175,00	25,796	97,00	36,00	25,623	4,61867	0,083988
2747	95,90	180,00	29,599	103,00	36,20	31,916	4,64065	0,080233
2748	92,80	176,00	29,959	110,00	36,20	32,613	5,34247	0,085958
2749	98,00	183,00	29,263	109,00	36,30	31,912	4,78348	0,084850
2750	84,00	174,50	27,586	97,00	36,40	28,617	4,65292	0,080429
2751	71,00	157,00	28,804	88,00	36,50	29,561	5,37832	0,074741
2752	76,60	172,00	25,892	90,00	36,60	26,387	4,38294	0,078409
2753	88,70	172,00	29,982	100,00	36,80	31,811	5,01993	0,079006
2754	85,00	170,00	29,412	103,00	36,80	31,504	5,36678	0,082908
2755	83,00	172,00	28,056	90,00	36,90	28,684	4,38294	0,074324
2756	82,60	168,00	29,266	108,00	36,90	31,622	5,86275	0,087739
2757	78,00	176,00	25,181	90,00	37,00	25,557	4,12564	0,078966
2758	79,00	170,00	27,336	97,00	37,00	27,953	4,97503	0,081984
2759	83,00	168,00	29,408	106,00	37,00	31,788	5,72880	0,085838
2760	83,90	175,00	27,396	103,00	37,10	29,385	4,98798	0,085676
2761	75,00	161,00	28,934	95,00	37,50	30,631	5,55746	0,079440
2762	87,20	179,00	27,215	98,00	37,50	29,392	4,41381	0,080958
2763	82,00	179,00	25,592	97,00	37,50	25,982	4,35505	0,083485
2764	73,30	160,00	28,633	100,00	37,50	30,670	6,01350	0,084469
2765	85,70	175,00	27,984	103,00	37,50	29,775	4,98798	0,084472
2766	91,80	177,00	29,302	98,00	37,60	31,081	4,54472	0,077501
2767	82,00	173,00	27,398	98,00	37,60	29,500	4,82045	0,081983
2768	72,30	170,00	25,017	96,00	37,60	25,201	4,90978	0,086077
2769	92,00	179,00	28,713	107,00	37,80	31,174	4,94311	0,085291
2770	73,00	159,00	28,875	110,00	37,90	31,263	6,85585	0,092685
2771	75,00	173,00	25,059	92,00	38,00	25,258	4,44274	0,081681
2772	77,00	168,00	27,282	95,00	38,10	29,473	4,99300	0,080876
2773	92,70	178,00	29,258	99,00	38,20	30,344	4,53816	0,078151
2774	82,30	180,00	25,401	91,00	38,30	24,811	3,94350	0,078494
2775	89,60	186,00	25,899	98,00	38,30	27,528	3,98873	0,082088
2776	90,10	178,00	28,437	98,00	38,40	28,977	4,47871	0,078842
2777	79,50	169,00	27,835	95,00	38,60	29,374	4,91811	0,079564
2778	97,00	195,00	25,510	94,00	38,70	25,223	3,31110	0,077680
2779	92,40	180,00	28,519	99,00	38,70	29,601	4,40812	0,079052
2780	81,10	166,00	29,431	100,00	38,70	32,008	5,48945	0,081422
2781	90,70	175,00	29,616	108,00	38,80	32,032	5,29603	0,085287
2782	87,60	175,00	28,604	101,00	38,90	29,814	4,86484	0,081630
2783	80,30	168,00	28,451	104,00	39,00	30,866	5,59491	0,086096
2784	91,50	181,00	27,930	101,00	39,10	28,301	4,45967	0,081553
2785	79,90	174,00	26,391	96,00	39,10	26,809	4,62523	0,082104
2786	83,50	169,00	29,236	104,00	39,10	31,685	5,51278	0,084297
2787	82,80	178,00	26,133	94,00	39,30	27,823	4,24103	0,080006
2788	78,80	169,00	27,590	100,00	39,30	29,288	5,24836	0,084247
2789	85,00	173,00	28,401	90,00	39,50	29,033	4,31692	0,073508
2790	80,50	165,00	29,568	104,00	39,60	31,690	5,85044	0,084672
2791	72,70	162,00	27,702	88,00	39,80	29,427	4,96890	0,075519
2792	92,80	180,00	28,642	100,00	40,00	30,579	4,46624	0,079621
2793	86,00	177,00	27,451	100,00	40,00	29,115	4,66499	0,082600
2794	87,90	174,00	29,033	104,00	40,00	29,958	5,12342	0,083464

2795	90,10	180,00	27,809	97,00	40,10	29,122	4,29191	0,078768
2796	82,70	173,00	27,632	97,00	40,40	29,340	4,75747	0,080688
2797	87,00	174,00	28,736	99,00	40,70	29,879	4,81197	0,079998
2798	92,00	178,00	29,037	105,00	41,00	29,764	4,89503	0,083307
2799	84,20	168,00	29,833	105,00	41,20	32,469	5,66185	0,084218
2800	89,00	178,00	28,090	104,00	41,20	29,050	4,83553	0,084357
2801	95,60	182,00	28,861	100,00	41,40	30,945	4,33921	0,078781
2802	82,00	167,00	29,402	96,00	41,50	31,879	5,13691	0,077982
2803	86,70	173,00	28,969	100,00	41,60	30,174	4,94644	0,080604
2804	85,00	169,00	29,761	99,00	42,00	31,543	5,18229	0,079298
2805	80,20	176,00	25,891	104,00	42,00	27,770	4,97699	0,089573
2806	87,80	179,00	27,402	100,00	42,10	28,604	4,53137	0,082233
2807	94,80	180,00	29,259	113,00	42,10	31,127	5,22265	0,088702
2808	75,20	164,00	27,960	97,00	42,40	29,961	5,44698	0,082224
2809	92,00	178,00	29,037	98,00	43,00	29,963	4,47871	0,077753
2810	82,90	170,00	28,685	104,00	44,10	30,937	5,43212	0,085121
2811	81,60	166,00	29,612	110,00	45,40	31,837	6,17547	0,089198
2812	88,70	173,00	29,637	104,00	46,00	31,677	5,19856	0,082564
2813	94,00	178,00	29,668	115,00	46,00	31,147	5,49062	0,089942
2814	83,70	173,00	27,966	96,00	46,90	30,293	4,69450	0,079218
2815	89,00	176,00	28,732	108,00	47,30	30,891	5,22060	0,086781
2816	87,10	171,50	29,614	101,00	47,90	31,459	5,12130	0,080574
2817	93,00	180,00	28,704	120,00	50,70	31,061	5,63061	0,095408
2818	71,60	162,50	27,115	88,00	51,30	29,271	4,93005	0,076487
2819	89,50	175,00	29,224	98,00	51,70	31,280	4,68020	0,078080
2820	84,00	169,00	29,411	89,00	52,00	31,696	4,52221	0,071853
2821	78,00	174,00	25,763	94,00	53,00	26,721	4,50079	0,081694
2822	94,40	173,00	31,541	102,00	5,60	33,168	5,07248	0,077683
2823	116,90	183,00	34,907	124,00	12,60	37,822	5,62873	0,085820
2824	90,20	167,00	32,343	106,00	13,00	35,030	5,81407	0,080804
2825	84,00	166,00	30,483	90,00	16,00	32,234	4,80473	0,071584
2826	94,60	169,00	33,122	110,00	19,00	35,240	5,90978	0,082042
2827	88,00	164,00	32,719	110,00	19,80	35,368	6,36088	0,083967
2828	90,60	170,00	31,349	103,00	19,90	33,349	5,36678	0,079456
2829	84,20	167,00	30,191	105,00	20,00	31,790	5,74630	0,083800
2830	94,70	169,00	33,157	115,00	20,00	36,058	6,24095	0,085711
2831	99,40	167,00	35,641	116,00	20,60	38,682	6,49251	0,082883
2832	86,70	167,00	31,088	90,00	20,80	32,620	4,73123	0,070441
2833	87,50	170,00	30,277	110,00	20,90	32,884	5,82437	0,086848
2834	89,00	170,00	30,796	100,00	21,00	31,919	5,17086	0,078063
2835	101,00	180,00	31,173	109,00	21,80	32,640	4,98974	0,082024
2836	100,10	170,00	34,637	106,00	22,10	37,357	5,56282	0,076511
2837	130,90	190,00	36,260	115,00	22,30	39,025	4,65306	0,076155
2838	100,60	181,50	30,538	93,00	22,70	31,596	3,97073	0,070656
2839	92,90	174,50	30,509	103,00	22,80	32,883	5,02438	0,079859
2840	92,00	175,00	30,041	96,00	23,10	31,056	4,55716	0,075095
2841	97,70	177,00	31,185	101,00	23,40	33,156	4,72514	0,076625
2842	117,00	189,50	32,581	119,00	23,40	34,995	4,89473	0,084742
2843	103,40	183,00	30,876	105,00	23,50	33,305	4,55841	0,078865
2844	91,20	174,00	30,123	104,00	23,70	31,889	5,12342	0,081438

2845	95,40	176,00	30,798	109,00	23,80	33,560	5,28153	0,083622
2846	97,40	176,00	31,444	112,00	23,80	33,705	5,46438	0,084743
2847	90,20	173,00	30,138	95,00	23,90	31,907	4,63155	0,074580
2848	89,90	173,00	30,038	99,00	24,00	31,235	4,88344	0,077893
2849	85,00	168,00	30,116	103,00	24,10	32,063	5,52798	0,082095
2850	80,40	161,00	31,017	94,00	24,30	33,128	5,48459	0,075043
2851	95,00	175,00	31,020	108,00	24,40	32,539	5,29603	0,082694
2852	90,00	172,00	30,422	98,00	24,50	33,049	4,89244	0,076678
2853	85,10	163,00	32,030	99,00	24,60	34,685	5,67302	0,076884
2854	90,00	170,00	31,142	100,00	24,60	33,357	5,17086	0,077484
2855	95,50	178,00	30,141	105,00	24,60	32,002	4,89503	0,081259
2856	109,50	184,00	32,343	117,00	24,60	34,652	5,16256	0,084969
2857	100,30	176,00	32,380	107,00	24,80	35,223	5,15969	0,079392
2858	81,60	163,00	30,712	102,00	24,80	33,204	5,88644	0,081464
2859	127,80	186,00	36,941	112,00	24,90	39,398	4,75062	0,074039
2860	95,40	177,50	30,280	98,00	24,90	31,898	4,51158	0,075717
2861	84,00	167,00	30,119	98,00	24,90	31,793	5,27224	0,078338
2862	85,90	169,00	30,076	99,00	24,90	32,655	5,18229	0,078743
2863	90,70	172,00	30,658	93,00	25,00	32,009	4,57392	0,072391
2864	92,00	175,00	30,041	101,00	25,00	32,065	4,86484	0,079006
2865	95,40	178,00	30,110	103,00	25,10	32,238	4,77603	0,079767
2866	95,30	176,00	30,766	109,00	25,10	32,573	5,28153	0,083681
2867	88,50	171,00	30,266	107,00	25,10	31,611	5,54662	0,084253
2868	90,50	171,50	30,769	96,00	25,20	33,442	4,80072	0,074655
2869	82,00	164,00	30,488	100,00	25,20	32,593	5,65767	0,080013
2870	99,10	177,00	31,632	110,00	25,20	33,599	5,26694	0,082665
2871	106,50	188,00	30,132	98,00	25,30	32,264	3,87600	0,073812
2872	93,60	176,50	30,046	101,00	25,30	31,979	4,75962	0,078661
2873	91,00	172,00	30,760	109,00	25,30	33,085	5,59419	0,084659
2874	98,00	172,00	33,126	102,00	25,40	34,890	5,14746	0,075403
2875	94,00	176,00	30,346	101,00	25,40	32,516	4,79439	0,078252
2876	86,00	165,00	31,589	106,00	25,40	33,144	5,98934	0,082580
2877	100,00	176,00	32,283	115,00	25,40	34,813	5,64733	0,085498
2878	107,00	180,00	33,025	119,00	25,40	35,845	5,57230	0,086169
2879	96,20	179,00	30,024	98,00	25,50	31,494	4,41381	0,075826
2880	83,10	162,00	31,664	98,00	25,50	33,637	5,68821	0,076929
2881	94,40	176,50	30,303	103,00	25,50	32,734	4,88064	0,079765
2882	103,60	183,00	30,936	109,00	25,50	32,265	4,78348	0,081764
2883	82,20	165,00	30,193	102,00	25,50	31,973	5,71158	0,081895
2884	92,40	172,00	31,233	107,00	25,50	34,045	5,46649	0,082264
2885	87,20	170,00	30,173	95,00	25,70	32,312	4,84455	0,075177
2886	93,70	175,00	30,596	98,00	25,70	32,983	4,68020	0,075729
2887	90,50	171,00	30,950	100,00	25,70	33,645	5,09472	0,077577
2888	93,00	176,00	30,023	105,00	25,70	31,676	5,03788	0,081933
2889	92,00	175,00	30,041	102,00	25,80	32,431	4,92640	0,079788
2890	96,20	177,00	30,706	100,00	25,90	32,763	4,66499	0,076653
2891	93,00	172,00	31,436	101,00	25,90	32,796	5,08369	0,077317
2892	95,00	174,00	31,378	102,00	25,90	33,465	4,99881	0,077728
2893	75,30	156,50	30,744	98,00	25,90	33,327	6,19325	0,079822
2894	86,00	165,00	31,589	103,00	25,90	34,033	5,78100	0,080243

2895	97,00	179,00	30,274	97,00	26,10	31,981	4,35505	0,074639
2896	83,00	165,00	30,487	94,00	26,10	31,619	5,15671	0,074986
2897	91,90	166,00	33,350	101,00	26,10	35,916	5,55799	0,075660
2898	104,00	185,00	30,387	105,00	26,10	32,653	4,43142	0,079276
2899	113,90	192,00	30,897	110,00	26,10	32,731	4,27330	0,080623
2900	103,50	180,00	31,944	109,00	26,10	33,964	4,98974	0,080697
2901	97,20	173,00	32,477	110,00	26,10	34,278	5,57707	0,082159
2902	96,00	178,00	30,299	98,00	26,20	31,652	4,47871	0,075578
2903	92,00	173,00	30,739	98,00	26,20	31,935	4,82045	0,075929
2904	88,30	169,00	30,916	102,00	26,20	32,326	5,38055	0,079652
2905	83,10	161,00	32,059	100,00	26,30	34,795	5,92201	0,078095
2906	109,00	183,00	32,548	109,00	26,30	34,212	4,78348	0,079041
2907	87,20	168,00	30,896	102,00	26,30	33,156	5,46107	0,079925
2908	86,60	168,00	30,683	103,00	26,30	32,997	5,52798	0,081080
2909	115,30	171,00	39,431	123,00	26,30	42,156	6,58168	0,081192
2910	98,40	180,00	30,370	101,00	26,40	31,830	4,52437	0,077337
2911	95,20	177,00	30,387	102,00	26,40	31,851	4,78530	0,078732
2912	90,00	173,00	30,071	104,00	26,40	31,552	5,19856	0,081767
2913	96,40	164,00	35,842	106,00	26,50	38,882	6,07943	0,076142
2914	91,30	172,50	30,683	100,00	26,50	33,431	4,98303	0,077686
2915	101,00	183,00	30,159	102,00	26,50	31,115	4,38970	0,077821
2916	93,00	174,00	30,717	101,00	26,50	32,949	4,93652	0,078065
2917	83,60	165,00	30,707	99,00	26,50	33,111	5,50340	0,078596
2918	91,00	170,00	31,488	103,00	26,50	33,674	5,36678	0,079223
2919	94,10	170,50	32,370	106,00	26,50	35,287	5,52225	0,079925
2920	84,50	167,00	30,299	102,00	26,50	32,819	5,54305	0,081213
2921	88,70	170,00	30,692	104,00	26,50	32,398	5,43212	0,081369
2922	92,00	171,50	31,279	108,00	26,50	33,599	5,57060	0,083071
2923	104,10	186,00	30,090	110,00	26,50	31,575	4,64168	0,083372
2924	90,10	166,00	32,697	114,00	26,50	35,492	6,45025	0,086532
2925	120,00	185,00	35,062	110,00	26,60	37,681	4,70663	0,075494
2926	79,20	160,00	30,938	98,00	26,60	32,267	5,86576	0,078616
2927	79,30	159,00	31,367	100,00	26,60	33,864	6,10673	0,079735
2928	90,40	173,00	30,205	102,00	26,60	31,354	5,07248	0,079958
2929	86,00	169,00	30,111	102,00	26,60	31,783	5,38055	0,081066
2930	94,80	173,00	31,675	107,00	26,60	33,730	5,38777	0,081261
2931	91,00	170,00	31,488	106,00	26,60	33,511	5,56282	0,081530
2932	86,80	169,00	30,391	95,00	26,70	33,014	4,91811	0,075038
2933	94,00	168,00	33,305	103,00	26,70	35,447	5,52798	0,076767
2934	84,70	165,00	31,111	101,00	26,70	32,733	5,64218	0,079488
2935	99,30	174,00	32,798	109,00	26,70	35,601	5,43513	0,080646
2936	96,60	178,50	30,318	106,00	26,70	31,765	4,91925	0,081599
2937	91,30	164,00	33,946	110,00	26,70	36,890	6,36088	0,081931
2938	96,50	173,00	32,243	114,00	26,70	35,074	5,82964	0,085558
2939	81,00	162,00	30,864	99,00	26,80	33,034	5,76022	0,079051
2940	78,80	161,50	30,212	98,00	26,80	32,810	5,73197	0,079497
2941	88,00	171,00	30,095	101,00	26,80	32,129	5,15925	0,079829
2942	84,90	167,00	30,442	103,00	26,80	32,895	5,61079	0,081752
2943	94,90	171,00	32,454	102,00	26,90	34,177	5,22378	0,076663
2944	98,40	181,00	30,036	101,00	26,90	32,150	4,45967	0,077694

2945	113,80	181,00	34,736	115,00	26,90	37,585	5,26534	0,080291
2946	97,50	177,00	31,121	106,00	26,90	33,585	5,02604	0,080528
2947	94,00	174,00	31,048	105,00	26,90	32,994	5,18574	0,080580
2948	86,90	169,00	30,426	103,00	26,90	32,165	5,44666	0,081295
2949	103,60	177,00	33,068	112,00	26,90	34,752	5,38746	0,081713
2950	99,60	178,00	31,435	109,00	26,90	33,532	5,13315	0,082023
2951	94,10	176,00	30,378	106,00	26,90	32,821	5,09878	0,082068
2952	88,60	168,00	31,392	107,00	26,90	33,153	5,79577	0,082957
2953	88,20	171,00	30,163	96,00	27,00	31,937	4,83675	0,075763
2954	88,00	166,50	31,743	99,00	27,00	34,607	5,38024	0,076529
2955	100,40	182,50	30,144	101,00	27,00	32,097	4,36464	0,077188
2956	84,40	167,00	30,263	97,00	27,00	31,787	5,20457	0,077293
2957	101,50	182,00	30,642	103,00	27,00	32,076	4,50976	0,077969
2958	91,90	171,00	31,428	102,00	27,00	33,360	5,22378	0,078323
2959	92,00	172,00	31,098	103,00	27,00	33,558	5,21125	0,079418
2960	87,10	170,00	30,138	102,00	27,00	32,271	5,30146	0,080778
2961	88,50	170,00	30,623	104,00	27,00	32,402	5,43212	0,081491
2962	86,80	166,00	31,499	108,00	27,00	34,013	6,03816	0,084043
2963	89,00	172,00	30,084	101,00	27,10	31,293	5,08369	0,079616
2964	77,60	160,00	30,313	98,00	27,10	31,759	5,86576	0,079693
2965	102,00	180,00	31,481	107,00	27,10	33,667	4,87334	0,079992
2966	88,20	170,00	30,519	104,00	27,10	31,660	5,43212	0,081676
2967	84,00	166,00	30,483	103,00	27,10	32,853	5,69512	0,081923
2968	86,90	169,00	30,426	108,00	27,10	33,143	5,77740	0,085241
2969	90,50	172,50	30,414	97,00	27,20	32,417	4,79294	0,075799
2970	97,90	179,50	30,385	100,00	27,20	32,383	4,49866	0,076653
2971	93,00	176,00	30,023	103,00	27,20	31,401	4,91611	0,080373
2972	90,00	173,00	30,071	104,00	27,20	31,460	5,19856	0,081767
2973	109,20	180,00	33,704	115,00	27,20	35,422	5,33916	0,082150
2974	90,30	171,00	30,881	106,00	27,20	33,054	5,48203	0,082353
2975	97,90	176,00	31,605	96,00	27,30	34,052	4,49026	0,072390
2976	88,00	169,00	30,811	97,00	27,30	33,661	5,05017	0,075920
2977	85,90	166,00	31,173	99,00	27,30	33,560	5,42092	0,077576
2978	100,00	176,00	32,283	107,00	27,30	34,355	5,15969	0,079551
2979	90,70	170,00	31,384	109,00	27,30	32,897	5,75896	0,084023
2980	96,00	176,00	30,992	110,00	27,30	32,928	5,34247	0,084037
2981	99,20	179,00	30,960	111,00	27,30	32,210	5,17860	0,084145
2982	84,90	167,50	30,261	109,00	27,30	32,866	5,97341	0,086730
2983	101,30	182,50	30,415	96,00	27,40	32,151	4,08212	0,072932
2984	94,00	175,50	30,519	98,00	27,40	32,718	4,64589	0,075748
2985	101,50	171,00	34,712	112,00	27,40	37,683	5,86976	0,080489
2986	94,20	175,00	30,759	106,00	27,40	32,825	5,17278	0,081621
2987	80,00	163,00	30,110	101,00	27,40	32,785	5,81528	0,081737
2988	96,40	176,50	30,945	108,00	27,40	33,298	5,18338	0,082476
2989	88,10	170,00	30,484	105,00	27,40	31,970	5,49746	0,082524
2990	89,00	162,00	33,913	114,00	27,40	36,723	6,84212	0,085488
2991	91,10	173,00	30,439	93,00	27,50	32,091	4,50566	0,072529
2992	99,60	182,00	30,069	99,00	27,50	31,732	4,28239	0,075891
2993	110,50	181,00	33,729	110,00	27,50	36,394	4,97739	0,078322
2994	102,00	182,00	30,793	106,00	27,50	32,089	4,68038	0,079977

2995	83,90	164,00	31,194	102,00	27,50	33,584	5,79820	0,080376
2996	94,20	175,00	30,759	105,00	27,50	32,911	5,11117	0,080851
2997	100,70	178,00	31,783	109,00	27,50	34,490	5,13315	0,081425
2998	110,80	189,00	31,018	114,00	27,50	32,790	4,66399	0,083997
2999	100,00	182,00	30,190	111,00	27,50	32,332	4,96493	0,084862
3000	100,40	175,00	32,784	117,00	27,50	35,293	5,85117	0,086343
3001	104,00	178,00	32,824	103,00	27,60	35,193	4,77603	0,075306
3002	99,30	178,00	31,341	100,00	27,60	33,999	4,59761	0,075402
3003	105,90	175,00	34,580	106,00	27,60	37,610	5,17278	0,075493
3004	84,50	167,00	30,299	96,00	27,60	31,742	5,13691	0,076436
3005	91,80	168,00	32,526	101,00	27,60	34,559	5,39416	0,076475
3006	86,60	168,00	30,683	100,00	27,60	32,387	5,32727	0,078719
3007	88,40	170,50	30,409	102,00	27,60	32,679	5,26245	0,080181
3008	98,90	178,50	31,040	106,00	27,60	33,408	4,91925	0,080329
3009	96,00	175,00	31,347	106,00	27,60	33,841	5,17278	0,080598
3010	91,00	173,00	30,405	104,00	27,60	32,852	5,19856	0,081167
3011	89,60	169,00	31,371	105,00	27,60	33,622	5,57892	0,081200
3012	110,00	188,00	31,123	114,00	27,60	33,083	4,72814	0,084031
3013	90,20	168,00	31,959	100,00	27,70	34,372	5,32727	0,076610
3014	104,50	185,00	30,533	105,00	27,70	32,559	4,43142	0,079023
3015	86,40	167,00	30,980	102,00	27,70	33,594	5,54305	0,080018
3016	100,50	180,00	31,019	108,00	27,70	32,536	4,93153	0,081541
3017	90,80	169,00	31,792	98,00	27,80	33,865	5,11622	0,075117
3018	90,60	171,00	30,984	100,00	27,80	32,919	5,09472	0,077520
3019	96,30	177,00	30,738	103,00	27,80	32,367	4,84547	0,078898
3020	93,40	176,00	30,152	102,00	27,80	31,652	4,85524	0,079365
3021	95,50	177,00	30,483	108,00	27,80	32,764	5,14647	0,083189
3022	99,80	170,50	34,331	116,00	27,80	37,035	6,17256	0,084102
3023	92,00	174,00	30,387	109,00	27,80	31,940	5,43513	0,084858
3024	93,70	172,00	31,673	114,00	27,80	34,449	5,91362	0,086833
3025	98,10	170,00	33,945	111,00	27,90	36,955	5,88978	0,081205
3026	87,60	170,00	30,311	103,00	27,90	31,668	5,36678	0,081260
3027	85,00	167,00	30,478	104,00	27,90	32,936	5,67854	0,082480
3028	101,60	181,00	31,012	110,00	27,90	32,614	4,97739	0,082832
3029	88,80	168,00	31,463	99,00	28,00	33,400	5,26039	0,076639
3030	95,30	172,00	32,213	103,00	28,00	34,735	5,21125	0,077574
3031	99,30	180,00	30,648	102,00	28,00	32,432	4,58250	0,077630
3032	90,30	167,00	32,378	103,00	28,00	34,541	5,61079	0,078459
3033	94,60	172,00	31,977	104,00	28,00	33,923	5,27504	0,078713
3034	93,10	176,00	30,056	103,00	28,00	32,265	4,91611	0,080315
3035	106,00	168,00	37,557	117,00	28,00	40,449	6,46612	0,080490
3036	106,80	185,00	31,205	114,00	28,00	32,680	4,92694	0,084560
3037	100,00	182,00	30,190	96,00	28,10	31,879	4,11195	0,073395
3038	110,00	188,00	31,123	100,00	28,10	33,502	3,98241	0,073712
3039	90,80	173,00	30,338	101,00	28,10	32,686	5,00946	0,078941
3040	95,90	174,00	31,675	105,00	28,10	34,211	5,18574	0,079512
3041	85,10	166,40	30,734	101,00	28,10	32,189	5,52475	0,079799
3042	94,00	169,00	32,912	108,00	28,10	35,363	5,77740	0,080893
3043	96,30	178,00	30,394	106,00	28,10	31,591	4,95455	0,081578
3044	96,90	176,00	31,282	100,00	28,20	33,851	4,73354	0,075924

3045	92,60	171,00	31,668	101,00	28,20	33,641	5,15925	0,077163
3046	87,90	170,00	30,415	99,00	28,20	32,953	5,10557	0,077926
3047	94,00	176,00	30,346	102,00	28,20	32,068	4,85524	0,079027
3048	118,70	178,50	37,254	118,00	28,20	39,714	5,63012	0,079180
3049	104,50	182,00	31,548	109,00	28,20	33,339	4,85108	0,080924
3050	98,20	176,00	31,702	108,00	28,20	33,121	5,22060	0,081272
3051	105,30	176,00	33,994	114,00	28,20	36,546	5,58634	0,081887
3052	105,80	184,00	31,250	111,00	28,20	32,817	4,82832	0,082480
3053	88,80	169,00	31,091	98,00	28,30	33,382	5,11622	0,076241
3054	99,60	172,00	33,667	105,00	28,30	36,733	5,33885	0,076787
3055	92,30	172,00	31,199	102,00	28,30	33,590	5,14746	0,078476
3056	95,80	176,00	30,927	104,00	28,30	33,278	4,97699	0,079564
3057	89,60	172,00	30,287	104,00	28,30	32,446	5,27504	0,081615
3058	91,90	175,00	30,008	106,00	28,30	32,026	5,17278	0,082978
3059	86,80	167,00	31,123	90,00	28,40	33,251	4,73123	0,070387
3060	88,50	170,00	30,623	96,00	28,40	32,927	4,90978	0,075223
3061	102,10	178,00	32,224	102,00	28,40	33,828	4,71655	0,075498
3062	102,60	182,00	30,975	102,00	28,40	32,568	4,45290	0,076659
3063	108,10	188,00	30,585	105,00	28,40	32,006	4,24857	0,078302
3064	99,70	182,00	30,099	105,00	28,40	31,860	4,62350	0,080436
3065	115,00	181,50	34,910	120,00	28,40	36,975	5,51544	0,083390
3066	99,80	180,00	30,802	116,00	28,40	32,703	5,39743	0,087990
3067	92,80	171,00	31,736	103,00	28,50	33,083	5,28833	0,078578
3068	103,30	180,50	31,706	107,00	28,50	33,926	4,83889	0,079503
3069	95,70	174,00	31,609	105,00	28,50	33,976	5,18574	0,079623
3070	98,20	178,00	30,994	105,00	28,50	32,760	4,89503	0,079762
3071	84,00	167,00	30,119	101,00	28,50	32,705	5,47533	0,080736
3072	84,80	167,50	30,225	102,00	28,50	32,464	5,50187	0,081224
3073	97,50	178,00	30,773	108,00	28,50	32,754	5,07360	0,082434
3074	83,00	164,00	30,860	105,00	28,50	32,257	6,00910	0,083337
3075	105,90	186,00	30,610	113,00	28,50	33,438	4,80510	0,084672
3076	106,40	176,00	34,349	120,00	28,50	37,182	5,95244	0,085601
3077	114,60	185,00	33,484	121,00	28,50	35,786	5,31282	0,085633
3078	87,00	169,00	30,461	97,00	28,60	32,650	5,05017	0,076501
3079	93,00	169,00	32,562	104,00	28,60	34,154	5,51278	0,078454
3080	111,90	186,50	32,172	109,00	28,60	33,842	4,55544	0,078905
3081	105,00	176,50	33,705	110,00	28,60	36,031	5,30455	0,079351
3082	88,90	169,00	31,126	103,00	28,60	32,751	5,44666	0,080071
3083	92,70	169,50	32,266	107,00	28,60	34,564	5,66952	0,081091
3084	84,90	168,00	30,081	103,00	28,60	32,752	5,52798	0,082159
3085	91,50	167,00	32,809	109,00	28,60	35,394	6,01747	0,082302
3086	98,00	175,00	32,000	110,00	28,60	34,184	5,41932	0,082497
3087	87,60	170,00	30,311	106,00	28,60	32,296	5,56282	0,083626
3088	88,00	171,00	30,095	108,00	28,60	32,403	5,61123	0,085362
3089	85,00	168,00	30,116	96,00	28,70	32,154	5,05983	0,076516
3090	93,00	175,00	30,367	102,00	28,70	32,273	4,92640	0,079215
3091	96,40	175,00	31,478	105,00	28,70	33,744	5,11117	0,079616
3092	89,10	167,00	31,948	104,00	28,70	33,419	5,67854	0,079930
3093	97,70	177,00	31,185	106,00	28,70	32,739	5,02604	0,080418
3094	90,90	174,00	30,024	103,00	28,70	32,412	5,06110	0,080832

3095	77,40	160,50	30,046	101,00	28,70	32,163	6,04096	0,082488
3096	81,30	164,00	30,228	96,00	28,80	31,654	5,37677	0,077253
3097	88,10	167,00	31,590	100,00	28,80	33,549	5,40762	0,077437
3098	97,40	174,00	32,171	108,00	28,80	34,841	5,37277	0,080942
3099	95,70	175,00	31,249	107,00	28,80	32,733	5,23439	0,081528
3100	83,90	167,00	30,084	108,00	28,80	32,755	5,94966	0,086400
3101	86,10	168,00	30,506	110,00	28,80	32,703	5,99674	0,086926
3102	90,00	173,00	30,071	114,00	28,80	32,101	5,82964	0,089629
3103	90,00	168,50	31,699	101,00	28,90	34,238	5,35413	0,077683
3104	96,30	174,00	31,807	104,00	28,90	34,121	5,12342	0,078537
3105	82,20	163,00	30,938	101,00	28,90	33,802	5,81528	0,080272
3106	107,50	179,00	33,551	112,00	28,90	35,518	5,23750	0,080474
3107	88,30	169,00	30,916	104,00	28,90	33,522	5,51278	0,081214
3108	91,00	173,00	30,405	105,00	28,90	32,407	5,26162	0,081947
3109	96,10	174,00	31,741	110,00	28,90	33,886	5,49751	0,083183
3110	103,80	181,00	31,684	114,00	28,90	34,061	5,20773	0,084626
3111	89,00	172,00	30,084	97,00	29,00	31,933	4,82871	0,076463
3112	89,70	169,00	31,406	101,00	29,00	33,416	5,31445	0,078049
3113	106,40	177,00	33,962	109,00	29,00	36,577	5,20670	0,078123
3114	101,50	170,00	35,121	110,00	29,00	37,624	5,82437	0,078666
3115	92,00	174,00	30,387	103,00	29,00	31,494	5,06110	0,080187
3116	102,00	181,00	31,135	107,00	29,00	33,432	4,80474	0,080362
3117	94,80	172,00	32,044	107,00	29,00	34,080	5,46649	0,080870
3118	85,80	167,00	30,765	103,00	29,00	33,437	5,61079	0,081179
3119	88,20	169,00	30,881	105,00	29,00	32,540	5,57892	0,082057
3120	90,00	170,00	31,142	107,00	29,00	33,441	5,62819	0,082908
3121	101,00	181,00	30,829	110,00	29,00	32,649	4,97739	0,083159
3122	85,00	167,50	30,296	105,00	29,00	32,547	5,70388	0,083481
3123	87,60	168,00	31,037	108,00	29,00	33,489	5,86275	0,084368
3124	106,90	182,00	32,273	117,00	29,00	34,954	5,30669	0,085558
3125	88,70	170,50	30,512	111,00	29,00	32,446	5,84726	0,087058
3126	93,00	172,00	31,436	114,00	29,00	33,942	5,91362	0,087268
3127	97,90	177,00	31,249	104,00	29,10	32,567	4,90565	0,078793
3128	101,80	182,00	30,733	105,00	29,10	32,794	4,62350	0,079326
3129	97,10	179,00	30,305	104,00	29,10	31,570	4,76659	0,079971
3130	88,00	171,00	30,095	104,00	29,10	31,946	5,35288	0,082201
3131	93,00	168,00	32,951	110,00	29,10	35,406	5,99674	0,082571
3132	96,90	174,00	32,006	101,00	29,20	34,035	4,93652	0,075956
3133	89,70	171,50	30,497	100,00	29,20	32,340	5,05716	0,078227
3134	84,00	167,00	30,119	98,00	29,20	31,793	5,27224	0,078338
3135	111,30	173,00	37,188	119,00	29,20	39,548	6,14559	0,081206
3136	83,70	167,00	30,012	102,00	29,20	31,662	5,54305	0,081730
3137	92,00	170,00	31,834	110,00	29,20	33,914	5,82437	0,083993
3138	81,30	164,00	30,228	105,00	29,20	32,918	6,00910	0,084495
3139	99,70	177,00	31,824	92,00	29,30	33,585	4,18416	0,068860
3140	126,00	187,00	36,032	106,00	29,30	38,850	4,36237	0,071055
3141	92,30	170,50	31,751	94,00	29,30	34,296	4,74342	0,071795
3142	91,30	173,00	30,506	95,00	29,30	32,437	4,63155	0,073980
3143	82,40	165,00	30,266	97,00	29,30	32,692	5,36469	0,077754
3144	100,40	172,00	33,937	110,00	29,30	36,815	5,65805	0,080016

3145	97,20	176,00	31,379	107,00	29,30	33,302	5,15969	0,081071
3146	104,80	181,00	31,989	111,00	29,30	34,641	5,03496	0,081874
3147	116,80	192,00	31,684	114,00	29,30	34,381	4,47759	0,082166
3148	96,70	176,00	31,218	109,00	29,30	33,611	5,28153	0,082871
3149	103,20	168,00	36,565	120,00	29,30	39,225	6,66747	0,084040
3150	98,00	171,00	33,515	117,00	29,30	35,889	6,19319	0,086073
3151	89,70	163,00	33,761	102,00	29,40	36,093	5,88644	0,076482
3152	93,00	172,00	31,436	101,00	29,40	33,860	5,08369	0,077317
3153	92,00	175,00	30,041	100,00	29,40	32,523	4,80328	0,078224
3154	102,70	175,00	33,535	108,00	29,40	35,295	5,29603	0,078507
3155	86,70	170,00	30,000	100,00	29,40	30,820	5,17086	0,079438
3156	100,80	175,00	32,914	108,00	29,40	35,510	5,29603	0,079490
3157	93,90	174,00	31,015	104,00	29,40	33,548	5,12342	0,079869
3158	99,60	181,00	30,402	105,00	29,40	32,225	4,68968	0,080121
3159	104,20	171,00	35,635	114,00	29,40	37,935	5,99909	0,080505
3160	98,10	179,00	30,617	106,00	29,40	32,570	4,88426	0,080954
3161	89,80	171,00	30,710	106,00	29,40	32,767	5,48203	0,082658
3162	91,80	173,00	30,673	107,00	29,40	33,333	5,38777	0,083022
3163	88,80	166,00	32,225	109,00	29,40	34,825	6,10681	0,083543
3164	97,60	172,00	32,991	113,00	29,40	35,450	5,84971	0,083763
3165	97,20	175,00	31,739	114,00	29,40	34,203	5,66603	0,085966
3166	90,90	170,00	31,453	96,00	29,50	33,389	4,90978	0,073893
3167	109,40	173,00	36,553	110,00	29,50	38,635	5,57707	0,075931
3168	102,60	178,00	32,382	104,00	29,50	34,696	4,83553	0,076728
3169	79,50	161,00	30,670	98,00	29,50	32,459	5,77615	0,078826
3170	82,80	166,00	30,048	99,00	29,50	32,623	5,42092	0,079501
3171	92,50	174,00	30,552	103,00	29,50	33,285	5,06110	0,079898
3172	80,40	162,00	30,636	100,00	29,50	33,116	5,83224	0,080246
3173	97,10	175,00	31,706	107,00	29,50	33,926	5,23439	0,080743
3174	100,00	174,00	33,029	110,00	29,50	35,564	5,49751	0,081006
3175	100,80	176,00	32,541	111,00	29,50	34,726	5,40342	0,082087
3176	95,00	176,00	30,669	107,00	29,50	33,329	5,15969	0,082318
3177	85,30	168,00	30,223	104,00	29,50	31,919	5,59491	0,082697
3178	98,80	172,00	33,396	114,00	29,50	35,689	5,91362	0,083819
3179	102,10	184,00	30,157	111,00	29,50	32,475	4,82832	0,084461
3180	112,00	185,00	32,725	118,00	29,50	35,668	5,14739	0,084797
3181	94,00	175,00	30,694	102,00	29,60	31,965	4,92640	0,078653
3182	91,20	165,00	33,499	106,00	29,60	35,734	5,98934	0,079411
3183	92,00	172,00	31,098	103,00	29,60	33,474	5,21125	0,079418
3184	119,00	179,00	37,140	120,00	29,60	39,898	5,70902	0,080574
3185	90,60	173,00	30,272	105,00	29,60	32,158	5,26162	0,082188
3186	81,00	162,00	30,864	95,00	29,70	33,291	5,47226	0,075857
3187	85,00	162,00	32,388	100,00	29,70	34,779	5,83224	0,077324
3188	91,50	168,00	32,419	102,00	29,70	35,039	5,46107	0,077401
3189	100,40	181,00	30,646	104,00	29,70	31,819	4,63216	0,078936
3190	98,30	180,00	30,340	104,00	29,70	31,792	4,69881	0,079688
3191	90,50	172,50	30,414	102,00	29,70	32,684	5,10981	0,079706
3192	99,50	176,00	32,122	107,00	29,70	34,478	5,15969	0,079817
3193	91,00	165,00	33,425	107,00	29,70	35,652	6,05882	0,080277
3194	97,00	173,50	32,224	108,00	29,70	35,129	5,41164	0,080970

3195	112,00	181,00	34,187	119,00	29,70	36,689	5,49586	0,083972
3196	126,70	198,00	32,318	122,00	29,70	34,016	4,51428	0,085453
3197	95,70	176,50	30,720	93,00	29,80	32,953	4,27593	0,071367
3198	98,70	180,00	30,463	101,00	29,80	31,944	4,52437	0,077180
3199	95,10	173,00	31,775	103,00	29,80	33,448	5,13552	0,078059
3200	103,00	177,00	32,877	107,00	29,80	35,541	5,08625	0,078368
3201	95,20	174,50	31,264	103,00	29,80	32,834	5,02438	0,078567
3202	104,50	177,00	33,356	111,00	29,80	35,991	5,32720	0,080517
3203	93,50	175,00	30,531	105,00	29,80	32,644	5,11117	0,081254
3204	95,00	176,00	30,669	107,00	29,80	33,154	5,15969	0,082318
3205	86,20	169,00	30,181	104,00	29,80	32,231	5,51278	0,082528
3206	91,60	172,00	30,963	109,00	29,80	33,319	5,59419	0,084289
3207	96,30	166,00	34,947	117,00	29,80	37,742	6,65648	0,084956
3208	95,80	171,00	32,762	103,00	29,90	34,977	5,28833	0,076929
3209	85,10	163,00	32,030	102,00	29,90	33,518	5,88644	0,079214
3210	95,50	175,00	31,184	105,00	29,90	33,155	5,11117	0,080116
3211	80,30	163,50	30,039	100,00	29,90	31,237	5,70071	0,080932
3212	90,40	170,00	31,280	105,00	29,90	33,020	5,49746	0,081118
3213	98,60	174,00	32,567	111,00	29,90	35,276	5,55989	0,082514
3214	91,10	167,00	32,665	111,00	29,90	35,457	6,15313	0,084057
3215	90,60	172,00	30,625	112,00	29,90	32,841	5,78581	0,087245
3216	101,20	179,00	31,585	118,00	29,90	34,190	5,59108	0,088269
3217	98,40	175,00	32,131	97,00	30,00	34,179	4,61867	0,072550
3218	95,40	172,00	32,247	102,00	30,00	34,314	5,14746	0,076767
3219	107,20	180,00	33,086	110,00	30,00	35,627	5,04795	0,079553
3220	105,00	182,00	31,699	109,00	30,00	33,197	4,85108	0,080666
3221	111,00	169,00	38,864	122,00	30,00	41,807	6,70510	0,081793
3222	85,00	167,00	30,478	105,00	30,00	32,847	5,74630	0,083274
3223	88,00	165,50	32,128	111,00	30,00	34,563	6,29029	0,085375
3224	102,50	173,50	34,051	100,00	30,10	35,887	4,91018	0,072266
3225	92,30	168,50	32,509	100,00	30,10	34,764	5,28764	0,075631
3226	102,00	166,00	37,016	111,00	30,10	39,726	6,24415	0,077567
3227	88,80	165,00	32,617	102,00	30,10	34,813	5,71158	0,077785
3228	106,80	182,50	32,066	107,00	30,10	33,639	4,70396	0,078473
3229	106,30	180,00	32,809	109,00	30,10	34,957	4,98974	0,079274
3230	89,00	170,00	30,796	103,00	30,10	32,696	5,36678	0,080405
3231	78,50	160,00	30,664	101,00	30,10	32,190	6,08739	0,081503
3232	94,00	177,00	30,004	105,00	30,10	32,113	4,96584	0,081736
3233	102,70	180,00	31,698	110,00	30,10	34,316	5,04795	0,081860
3234	87,30	164,00	32,458	108,00	30,10	35,308	6,22013	0,082880
3235	96,00	169,00	33,612	113,00	30,10	36,608	6,10844	0,083458
3236	97,00	176,00	31,315	118,00	30,10	34,052	5,83037	0,089529
3237	101,00	173,50	33,552	106,00	30,20	35,793	5,28621	0,077358
3238	86,50	169,00	30,286	98,00	30,20	32,895	5,11622	0,077587
3239	90,20	171,00	30,847	100,00	30,20	33,100	5,09472	0,077749
3240	99,00	175,00	32,327	110,00	30,20	34,937	5,41932	0,081941
3241	90,60	172,00	30,625	108,00	30,20	32,929	5,53033	0,084129
3242	93,20	170,00	32,249	100,00	30,30	34,469	5,17086	0,075700
3243	102,90	183,00	30,727	101,00	30,30	32,093	4,33349	0,076106
3244	87,30	167,00	31,303	98,00	30,30	33,873	5,27224	0,076351

3245	98,40	176,00	31,767	102,00	30,30	33,916	4,85524	0,076653
3246	103,40	181,00	31,562	105,00	30,30	33,355	4,68968	0,078146
3247	92,10	164,00	34,243	107,00	30,30	37,071	6,14977	0,079234
3248	111,30	168,00	39,435	120,00	30,30	42,078	6,66747	0,079911
3249	88,20	168,50	31,065	103,00	30,30	33,605	5,48714	0,080296
3250	111,00	173,00	37,088	118,00	30,30	39,647	6,08238	0,080669
3251	103,00	184,00	30,423	107,00	30,30	31,628	4,60567	0,080942
3252	87,20	168,00	30,896	104,00	30,30	33,670	5,59491	0,081492
3253	85,00	166,00	30,846	106,00	30,30	32,670	5,90091	0,083647
3254	103,40	176,00	33,381	97,00	30,40	35,671	4,55106	0,070526
3255	100,20	178,00	31,625	100,00	30,40	33,671	4,59761	0,074950
3256	95,40	178,00	30,110	100,00	30,40	32,511	4,59761	0,077443
3257	97,60	178,00	30,804	103,00	30,40	32,878	4,77603	0,078563
3258	93,00	172,50	31,254	103,00	30,40	33,736	5,17322	0,079039
3259	93,30	176,00	30,120	104,00	30,40	31,248	4,97699	0,080979
3260	92,60	174,50	30,410	108,00	30,40	31,968	5,33423	0,083916
3261	91,00	167,00	32,629	99,00	30,50	34,604	5,33993	0,075025
3262	92,30	171,00	31,565	100,00	30,50	33,683	5,09472	0,076565
3263	93,50	176,00	30,185	100,00	30,50	32,779	4,73354	0,077753
3264	91,40	169,00	32,002	102,00	30,50	34,498	5,38055	0,077841
3265	106,30	177,00	33,930	109,00	30,50	35,948	5,20670	0,078172
3266	98,90	176,00	31,928	106,00	30,50	34,573	5,09878	0,079391
3267	111,00	179,00	34,643	113,00	30,50	37,426	5,29640	0,079477
3268	102,00	174,00	33,690	110,00	30,50	36,149	5,49751	0,079944
3269	79,90	160,00	31,211	101,00	30,50	33,103	6,08739	0,080548
3270	100,00	182,00	30,190	106,00	30,50	31,335	4,68038	0,081040
3271	91,00	173,00	30,405	107,00	30,50	33,030	5,38777	0,083508
3272	92,50	174,00	30,552	109,00	30,50	32,493	5,43513	0,084552
3273	98,80	178,00	31,183	112,00	30,50	33,822	5,31184	0,084735
3274	95,50	174,00	31,543	113,00	30,50	33,981	5,68470	0,085809
3275	99,80	170,00	34,533	120,00	30,50	36,495	6,47907	0,086790
3276	94,00	175,00	30,694	113,00	30,50	33,096	5,60434	0,087135
3277	111,00	175,00	36,245	110,00	30,60	38,524	5,41932	0,075923
3278	110,90	185,00	32,403	105,00	30,60	34,343	4,43142	0,075953
3279	100,30	168,00	35,537	107,00	30,60	38,291	5,79577	0,076373
3280	94,80	170,00	32,803	104,00	30,60	35,606	5,43212	0,077840
3281	105,40	172,00	35,627	111,00	30,60	38,155	5,72192	0,078169
3282	111,30	182,50	33,417	110,00	30,60	35,643	4,87374	0,078484
3283	92,00	175,00	30,041	101,00	30,60	31,331	4,86484	0,079006
3284	98,90	180,00	30,525	105,00	30,60	33,166	4,75698	0,080128
3285	104,00	183,00	31,055	108,00	30,60	32,496	4,72720	0,080806
3286	98,20	176,00	31,702	108,00	30,60	33,201	5,22060	0,081272
3287	75,50	156,00	31,024	101,00	30,60	33,728	6,47525	0,081902
3288	85,30	163,00	32,105	110,00	30,60	35,001	6,45619	0,085294
3289	96,60	173,00	32,276	116,00	30,60	34,653	5,95598	0,086998
3290	91,50	168,00	32,419	117,00	30,60	35,039	6,46612	0,088783
3291	105,80	176,00	34,155	101,00	30,70	36,330	4,79439	0,072320
3292	101,50	164,00	37,738	113,00	30,70	40,622	6,57211	0,078428
3293	90,20	167,00	32,343	104,00	30,70	34,575	5,67854	0,079279
3294	96,90	179,00	30,243	106,00	30,70	32,394	4,88426	0,081621

3295	96,70	175,00	31,576	108,00	30,70	33,775	5,29603	0,081722
3296	95,30	174,50	31,297	108,00	30,70	33,288	5,33423	0,082324
3297	109,00	180,00	33,642	110,00	30,80	36,504	5,04795	0,078675
3298	105,50	178,00	33,298	109,00	30,80	35,439	5,13315	0,078936
3299	94,50	174,00	31,213	107,00	30,80	32,522	5,31041	0,081825
3300	92,40	174,00	30,519	106,00	30,80	33,159	5,24807	0,082284
3301	89,00	170,00	30,796	107,00	30,80	32,954	5,62819	0,083528
3302	92,40	164,00	34,355	114,00	30,80	37,060	6,64255	0,084235
3303	86,00	168,00	30,471	107,00	30,80	32,927	5,79577	0,084621
3304	99,60	179,50	30,912	112,00	30,80	32,578	5,20080	0,084872
3305	88,80	165,00	32,617	94,00	30,90	34,887	5,15671	0,071684
3306	105,40	176,00	34,026	110,00	30,90	36,056	5,34247	0,078963
3307	88,90	163,00	33,460	105,00	30,90	35,828	6,09999	0,079204
3308	92,00	170,50	31,647	104,00	30,90	33,537	5,39232	0,079606
3309	88,80	167,00	31,841	105,00	30,90	34,634	5,74630	0,080881
3310	117,50	174,00	38,810	123,00	30,90	41,455	6,30938	0,081346
3311	111,00	185,00	32,432	113,00	30,90	34,452	4,87185	0,081691
3312	95,00	173,00	31,742	108,00	30,90	33,728	5,45086	0,081906
3313	80,00	160,00	31,250	103,00	30,90	33,150	6,23521	0,082075
3314	119,00	185,00	34,770	119,00	30,90	37,128	5,20253	0,082128
3315	95,20	176,00	30,733	108,00	30,90	31,841	5,22060	0,082971
3316	96,00	172,00	32,450	112,00	30,90	34,697	5,78581	0,083942
3317	86,10	168,00	30,506	107,00	30,90	32,968	5,79577	0,084555
3318	103,80	175,00	33,894	119,00	30,90	36,703	5,97466	0,085891
3319	93,10	175,00	30,400	98,00	31,00	31,777	4,68020	0,076054
3320	100,70	173,00	33,646	105,00	31,00	35,356	5,26162	0,076596
3321	97,40	162,00	37,113	109,00	31,00	39,472	6,48112	0,076969
3322	94,10	170,00	32,561	104,00	31,00	35,344	5,43212	0,078225
3323	90,70	171,00	31,018	102,00	31,00	32,874	5,22378	0,079012
3324	88,00	166,00	31,935	105,00	31,00	34,580	5,83230	0,080964
3325	94,00	168,00	33,305	109,00	31,00	35,936	5,92974	0,081239
3326	105,50	176,50	33,866	113,00	31,00	36,075	5,48637	0,081258
3327	101,20	176,00	32,670	111,00	31,00	34,873	5,40342	0,081871
3328	92,30	173,00	30,840	106,00	31,00	32,318	5,32469	0,081949
3329	103,00	181,00	31,440	110,00	31,00	34,109	4,97739	0,082079
3330	119,00	182,00	35,926	121,00	31,00	37,910	5,53471	0,082378
3331	92,40	175,00	30,171	108,00	31,00	32,220	5,29603	0,084238
3332	100,00	176,00	32,283	115,00	31,00	34,813	5,64733	0,085498
3333	111,80	185,50	32,490	119,00	31,00	35,118	5,16720	0,085810
3334	87,50	166,00	31,754	114,00	31,00	34,299	6,45025	0,088238
3335	95,10	176,00	30,701	116,00	31,00	33,278	5,70833	0,089179
3336	78,70	161,50	30,174	92,00	31,10	32,676	5,29748	0,074693
3337	96,00	167,00	34,422	105,00	31,10	37,258	5,74630	0,076784
3338	95,00	173,00	31,742	102,00	31,10	33,807	5,07248	0,077355
3339	89,40	167,00	32,056	101,00	31,10	34,559	5,47533	0,077451
3340	93,50	176,00	30,185	101,00	31,10	31,420	4,79439	0,078531
3341	92,50	170,00	32,007	104,00	31,10	33,412	5,43212	0,079125
3342	90,40	170,00	31,280	104,00	31,10	32,605	5,43212	0,080346
3343	101,70	166,00	36,907	115,00	31,10	39,363	6,51898	0,080521
3344	106,20	176,00	34,285	113,00	31,10	36,858	5,52536	0,080709

3345	109,90	186,00	31,767	111,00	31,10	33,279	4,69615	0,081143
3346	95,00	170,00	32,872	110,00	31,10	34,521	5,82437	0,082215
3347	99,20	174,00	32,765	112,00	31,10	35,346	5,62229	0,082922
3348	92,80	173,00	31,007	108,00	31,10	32,946	5,45086	0,083195
3349	90,20	172,00	30,489	108,00	31,10	32,684	5,53033	0,084377
3350	109,90	180,00	33,920	120,00	31,10	36,532	5,63061	0,085358
3351	92,00	175,00	30,041	96,00	31,20	31,973	4,55716	0,075095
3352	106,50	178,00	33,613	110,00	31,20	36,133	5,19270	0,079161
3353	113,00	177,00	36,069	115,00	31,20	38,613	5,56830	0,079181
3354	111,40	180,00	34,383	113,00	31,20	36,390	5,22265	0,079655
3355	97,00	179,00	30,274	104,00	31,20	31,981	4,76659	0,080026
3356	90,00	168,00	31,888	106,00	31,20	34,843	5,72880	0,081327
3357	83,30	166,00	30,229	103,00	31,20	32,379	5,69512	0,082382
3358	84,00	167,00	30,119	106,00	31,20	32,431	5,81407	0,084733
3359	109,00	185,00	31,848	118,00	31,20	33,298	5,14739	0,086346
3360	89,20	171,00	30,505	111,00	31,20	32,967	5,80511	0,086945
3361	102,90	177,50	32,660	103,00	31,30	34,861	4,81061	0,075665
3362	94,20	175,00	30,759	100,00	31,30	33,171	4,80328	0,077001
3363	93,20	165,00	34,233	105,00	31,30	37,061	5,91988	0,077532
3364	97,40	168,00	34,510	107,00	31,30	37,287	5,79577	0,077882
3365	112,50	179,00	35,111	113,00	31,30	37,196	5,29640	0,078769
3366	100,90	180,00	31,142	106,00	31,30	33,944	4,81515	0,079819
3367	89,70	171,00	30,676	104,00	31,30	32,727	5,35288	0,081159
3368	88,00	165,00	32,323	106,00	31,30	34,250	5,98934	0,081324
3369	88,40	171,00	30,232	104,00	31,30	32,110	5,35288	0,081952
3370	92,60	173,00	30,940	108,00	31,30	33,463	5,45086	0,083315
3371	91,70	171,00	31,360	110,00	31,30	33,115	5,74047	0,084588
3372	103,80	178,00	32,761	120,00	31,30	35,561	5,78878	0,087848
3373	90,50	173,00	30,238	99,00	31,40	32,389	4,88344	0,077549
3374	86,70	168,00	30,719	100,00	31,40	33,038	5,32727	0,078658
3375	101,00	171,00	34,541	110,00	31,40	37,256	5,74047	0,079313
3376	94,70	172,00	32,011	106,00	31,40	34,508	5,40267	0,080170
3377	99,80	171,00	34,130	111,00	31,40	36,692	5,80511	0,080674
3378	87,60	164,00	32,570	106,00	31,40	35,428	6,07943	0,081160
3379	111,00	178,00	35,033	116,00	31,40	37,952	5,55023	0,081207
3380	91,50	172,50	30,750	105,00	31,40	33,420	5,30006	0,081452
3381	90,90	166,00	32,987	108,00	31,40	35,733	6,03816	0,081496
3382	87,20	165,00	32,029	107,00	31,40	34,607	6,05882	0,082593
3383	109,20	177,00	34,856	119,00	31,40	37,526	5,80957	0,083826
3384	103,00	178,00	32,509	103,00	31,50	34,764	4,77603	0,075793
3385	88,80	170,00	30,727	100,00	31,50	32,787	5,17086	0,078181
3386	95,00	176,00	30,669	103,00	31,50	32,719	4,91611	0,079241
3387	104,30	183,00	31,145	107,00	31,50	33,108	4,67093	0,079904
3388	89,00	167,00	31,912	104,00	31,50	34,555	5,67854	0,079990
3389	95,70	167,00	34,315	110,00	31,50	37,338	6,08529	0,080609
3390	95,10	176,00	30,701	105,00	31,50	33,191	5,03788	0,080723
3391	96,00	170,00	33,218	111,00	31,50	36,053	5,88978	0,082385
3392	101,00	178,00	31,877	111,00	31,50	34,201	5,25226	0,082755
3393	86,90	169,00	30,426	105,00	31,50	33,143	5,57892	0,082873
3394	88,80	168,00	31,463	109,00	31,50	33,890	5,92974	0,084381

3395	98,00	171,00	33,515	115,00	31,50	35,614	6,06378	0,084601
3396	99,20	178,00	31,309	115,00	31,50	33,715	5,49062	0,086771
3397	99,40	179,00	31,023	101,00	31,60	33,303	4,59016	0,076461
3398	110,00	177,00	35,111	110,00	31,60	37,017	5,26694	0,077110
3399	99,70	167,00	35,749	109,00	31,60	38,677	6,01747	0,077725
3400	87,00	169,00	30,461	100,00	31,60	32,828	5,24836	0,078867
3401	105,00	180,00	32,407	108,00	31,60	34,498	4,93153	0,079194
3402	102,70	184,00	30,334	105,00	31,60	32,771	4,49439	0,079584
3403	95,00	172,00	32,112	106,00	31,60	34,622	5,40267	0,080001
3404	90,00	167,00	32,271	108,00	31,60	35,104	5,94966	0,082450
3405	100,00	172,00	33,802	117,00	31,60	36,874	6,10541	0,085335
3406	106,00	175,00	34,612	120,00	31,60	36,958	6,03642	0,085410
3407	93,80	175,00	30,629	102,00	31,70	33,196	4,92640	0,078764
3408	105,30	177,00	33,611	110,00	31,70	36,131	5,26694	0,079387
3409	91,10	173,00	30,439	103,00	31,70	32,713	5,13552	0,080327
3410	84,00	166,50	30,301	101,00	31,70	33,091	5,51647	0,080534
3411	91,70	165,00	33,682	108,00	31,70	36,614	6,12831	0,080615
3412	92,00	168,00	32,596	107,00	31,70	34,789	5,79577	0,080900
3413	84,50	167,00	30,299	107,00	31,70	31,921	5,88186	0,085194
3414	124,60	191,00	34,155	127,00	31,70	36,654	5,21059	0,087294
3415	106,90	178,50	33,551	109,00	31,80	35,381	5,09684	0,078428
3416	89,70	167,00	32,163	103,00	31,80	34,910	5,61079	0,078808
3417	74,90	158,00	30,003	97,00	31,80	32,296	5,97443	0,079922
3418	91,40	173,00	30,539	103,00	31,80	33,006	5,13552	0,080151
3419	88,80	164,00	33,016	106,00	31,80	35,263	6,07943	0,080427
3420	93,00	170,50	31,991	106,00	31,80	33,862	5,52225	0,080554
3421	90,00	170,00	31,142	104,00	31,80	33,692	5,43212	0,080583
3422	92,00	174,00	30,387	104,00	31,80	31,940	5,12342	0,080965
3423	98,00	173,50	32,556	109,00	31,80	34,743	5,47437	0,081163
3424	88,00	169,00	30,811	104,00	31,80	33,575	5,51278	0,081399
3425	99,20	173,00	33,145	112,00	31,80	35,762	5,70333	0,082524
3426	95,80	174,00	31,642	110,00	31,80	34,335	5,49751	0,083357
3427	94,00	168,50	33,108	115,00	31,80	34,727	6,28624	0,085924
3428	99,60	170,00	34,464	108,00	31,90	36,544	5,69357	0,078215
3429	96,30	178,00	30,394	104,00	31,90	31,681	4,83553	0,080039
3430	90,10	173,00	30,105	102,00	31,90	31,867	5,07248	0,080135
3431	100,00	181,50	30,356	107,00	31,90	32,170	4,77086	0,081617
3432	96,20	162,00	36,656	116,00	31,90	39,525	6,98662	0,082592
3433	95,00	174,50	31,198	115,00	31,90	33,756	5,76846	0,087844
3434	90,10	168,00	31,923	101,00	32,00	34,332	5,39416	0,077434
3435	101,60	174,00	33,558	109,00	32,00	36,210	5,43513	0,079425
3436	106,80	175,50	34,675	112,00	32,00	36,778	5,50335	0,079506
3437	100,00	176,00	32,283	107,00	32,00	35,270	5,15969	0,079551
3438	93,70	165,50	34,209	109,00	32,00	36,648	6,15210	0,080402
3439	88,00	165,00	32,323	105,00	32,00	34,478	5,91988	0,080557
3440	108,10	181,00	32,997	113,00	32,00	35,385	5,15013	0,081644
3441	79,80	161,00	30,786	106,00	32,00	33,547	6,35997	0,085047
3442	90,00	165,00	33,058	113,00	32,00	36,024	6,47594	0,085406
3443	88,00	169,00	30,811	112,00	32,00	33,575	6,04221	0,087660
3444	87,00	164,00	32,347	101,00	32,10	35,035	5,72793	0,077686

3445	91,80	170,00	31,765	102,00	32,10	33,993	5,30146	0,077997
3446	92,60	173,00	30,940	103,00	32,10	33,634	5,13552	0,079458
3447	96,30	166,00	34,947	111,00	32,10	37,682	6,24415	0,080599
3448	92,30	173,00	30,840	105,00	32,10	33,091	5,26162	0,081176
3449	91,10	170,00	31,522	106,00	32,10	33,308	5,56282	0,081471
3450	82,50	164,00	30,674	104,00	32,10	33,160	5,93879	0,082877
3451	93,60	174,50	30,739	108,00	32,10	32,974	5,33423	0,083317
3452	113,50	174,00	37,488	124,00	32,10	39,900	6,37191	0,083923
3453	80,40	161,50	30,826	106,00	32,10	32,989	6,31211	0,084842
3454	100,70	181,00	30,738	113,00	32,10	33,060	5,15013	0,085597
3455	95,00	171,00	32,489	101,00	32,20	35,041	5,15925	0,075858
3456	94,50	173,00	31,575	102,00	32,20	33,775	5,07248	0,077628
3457	95,30	169,00	33,367	107,00	32,20	35,309	5,71123	0,079413
3458	97,70	169,00	34,207	109,00	32,20	37,227	5,84358	0,079567
3459	85,00	161,50	32,589	104,00	32,20	35,523	6,16699	0,080210
3460	115,00	170,00	39,792	122,00	32,20	42,088	6,61016	0,080279
3461	107,10	186,00	30,957	110,00	32,20	32,887	4,64168	0,081808
3462	86,50	168,00	30,648	104,00	32,20	32,083	5,59491	0,081931
3463	108,90	183,00	32,518	114,00	32,20	34,999	5,06501	0,082717
3464	94,00	176,50	30,174	107,00	32,20	32,132	5,12281	0,083097
3465	89,60	170,00	31,003	107,00	32,20	33,535	5,62819	0,083155
3466	115,90	173,00	38,725	115,00	32,30	41,202	5,89280	0,076386
3467	102,30	180,00	31,574	103,00	32,30	33,531	4,64065	0,076851
3468	96,40	177,00	30,770	101,00	32,30	32,838	4,72514	0,077312
3469	84,60	167,50	30,154	97,00	32,30	32,744	5,16545	0,077364
3470	92,00	170,00	31,834	103,00	32,30	34,626	5,36678	0,078648
3471	98,00	174,00	32,369	106,00	32,30	34,454	5,24807	0,079119
3472	98,00	168,50	34,516	110,00	32,30	37,105	5,95307	0,079936
3473	80,30	156,50	32,786	103,00	32,30	35,734	6,57977	0,080375
3474	97,00	172,00	32,788	108,00	32,30	35,736	5,53033	0,080386
3475	85,90	168,00	30,435	102,00	32,30	31,643	5,46107	0,080729
3476	97,30	175,00	31,771	108,00	32,30	34,239	5,29603	0,081385
3477	102,00	170,00	35,294	115,00	32,30	38,100	6,15157	0,081973
3478	104,80	180,00	32,346	112,00	32,30	34,124	5,16441	0,082231
3479	87,80	168,00	31,108	107,00	32,30	33,066	5,79577	0,083460
3480	88,70	171,00	30,334	110,00	32,30	31,696	5,74047	0,086485
3481	97,00	176,00	31,315	115,00	32,30	33,143	5,64733	0,087252
3482	116,10	190,00	32,161	123,00	32,30	34,214	5,07085	0,088236
3483	99,30	175,00	32,424	105,00	32,40	33,916	5,11117	0,078059
3484	93,50	165,00	34,343	106,00	32,40	36,985	5,98934	0,078103
3485	94,20	174,00	31,114	104,00	32,40	33,072	5,12342	0,079700
3486	97,70	178,00	30,836	108,00	32,40	32,657	5,07360	0,082321
3487	100,00	180,00	30,864	109,00	32,40	32,948	4,98974	0,082570
3488	112,20	175,00	36,637	123,00	32,40	39,401	6,22175	0,084290
3489	88,00	168,00	31,179	112,00	32,40	33,483	6,13079	0,087228
3490	115,70	181,50	35,122	135,00	32,40	38,102	6,37650	0,093435
3491	99,70	176,00	32,186	102,00	32,50	34,090	4,85524	0,075985
3492	98,70	177,00	31,504	101,00	32,50	33,124	4,72514	0,076106
3493	128,70	181,00	39,285	120,00	32,50	41,484	5,55351	0,077184
3494	86,80	168,50	30,572	98,00	32,50	32,955	5,15470	0,077217

3495	98,50	178,50	30,914	102,00	32,50	32,751	4,68261	0,077507
3496	108,00	174,00	35,672	111,00	32,50	37,747	5,55989	0,077654
3497	112,00	172,00	37,858	116,00	32,50	40,175	6,04147	0,078449
3498	106,50	172,00	35,999	116,00	32,50	38,320	6,04147	0,081127
3499	91,90	170,00	31,799	107,00	32,50	34,430	5,62819	0,081761
3500	102,00	175,00	33,306	116,00	32,50	36,286	5,78945	0,084708
3501	104,00	186,00	30,061	113,00	32,50	31,906	4,80510	0,085701
3502	97,30	179,50	30,198	102,00	32,60	31,618	4,61559	0,078508
3503	89,60	172,00	30,287	102,00	32,60	32,985	5,14746	0,080045
3504	96,30	174,50	31,625	106,00	32,60	33,269	5,21026	0,080239
3505	99,00	181,00	30,219	105,00	32,60	31,191	4,68968	0,080445
3506	101,00	177,50	32,057	110,00	32,60	34,172	5,22966	0,081817
3507	93,70	173,50	31,127	107,00	32,60	33,676	5,34892	0,082093
3508	104,00	183,00	31,055	111,00	32,60	33,256	4,89606	0,083051
3509	91,10	172,50	30,615	107,00	32,60	33,268	5,42696	0,083246
3510	110,60	171,00	37,824	110,00	32,70	40,187	5,74047	0,074654
3511	93,90	162,50	35,560	107,00	32,70	38,028	6,28940	0,077622
3512	112,80	185,50	32,781	109,00	32,70	35,290	4,61926	0,078134
3513	97,70	174,00	32,270	108,00	32,70	34,035	5,37277	0,080777
3514	85,20	163,00	32,067	105,00	32,70	33,951	6,09999	0,081480
3515	90,70	162,50	34,348	110,00	32,70	37,181	6,50451	0,081664
3516	92,30	173,00	30,840	106,00	32,70	33,177	5,32469	0,081949
3517	94,70	164,00	35,210	113,00	32,70	37,777	6,57211	0,082139
3518	107,00	178,00	33,771	116,00	32,70	36,439	5,55023	0,083218
3519	80,00	160,00	31,250	107,00	32,70	33,980	6,53104	0,085262
3520	98,80	169,00	34,593	102,00	32,80	37,186	5,38055	0,073904
3521	94,10	169,00	32,947	103,00	32,80	35,402	5,44666	0,077093
3522	97,70	172,00	33,025	106,00	32,80	35,845	5,40267	0,078521
3523	89,30	170,00	30,900	101,00	32,80	33,588	5,23615	0,078667
3524	126,40	187,00	36,146	119,00	32,80	38,311	5,06295	0,079601
3525	100,00	175,00	32,653	109,00	32,80	34,263	5,35767	0,080654
3526	89,10	170,00	30,830	104,00	32,80	31,705	5,43212	0,081125
3527	99,40	176,00	32,089	109,00	32,80	34,132	5,28153	0,081363
3528	89,10	169,00	31,196	107,00	32,80	32,418	5,71123	0,083056
3529	91,50	171,00	31,292	109,00	32,80	33,033	5,67584	0,083941
3530	114,90	183,00	34,310	121,00	32,80	36,565	5,45952	0,084713
3531	90,60	170,50	31,166	97,00	32,90	33,552	4,93797	0,075011
3532	107,80	178,00	34,023	110,00	32,90	36,446	5,19270	0,078523
3533	103,00	172,00	34,816	110,00	32,90	37,117	5,65805	0,078664
3534	101,00	179,00	31,522	106,00	32,90	34,120	4,88426	0,079397
3535	95,00	171,00	32,489	107,00	32,90	34,517	5,54662	0,080365
3536	87,90	161,00	33,911	107,00	32,90	36,522	6,43301	0,080491
3537	87,40	168,00	30,967	103,00	32,90	32,643	5,52798	0,080585
3538	96,70	169,00	33,857	110,00	32,90	36,665	5,90978	0,080850
3539	84,50	159,00	33,424	108,00	32,90	36,134	6,70590	0,082544
3540	93,50	170,00	32,353	114,00	32,90	34,739	6,08611	0,086113
3541	93,00	173,00	31,074	95,00	33,00	33,530	4,63155	0,073076
3542	86,90	164,00	32,310	104,00	33,00	35,222	5,93879	0,080055
3543	94,60	176,00	30,540	104,00	33,00	33,095	4,97699	0,080235
3544	94,00	171,00	32,147	107,00	33,00	33,581	5,54662	0,080934

3545	89,00	162,00	33,913	109,00	33,00	36,723	6,48112	0,081739
3546	92,70	164,00	34,466	112,00	33,00	37,241	6,50168	0,082579
3547	99,00	177,00	31,600	111,00	33,00	33,320	5,32720	0,083473
3548	106,40	175,00	34,743	119,00	33,00	37,284	5,97466	0,084486
3549	108,60	176,00	35,059	121,00	33,00	38,098	6,01350	0,085145
3550	100,80	170,50	34,675	108,00	33,10	36,839	5,65222	0,077783
3551	108,10	177,00	34,505	110,00	33,10	36,715	5,26694	0,078011
3552	97,80	173,00	32,677	107,00	33,10	34,439	5,38777	0,079591
3553	92,60	169,00	32,422	107,00	33,10	35,193	5,71123	0,080950
3554	99,70	180,00	30,772	110,00	33,10	32,667	5,04795	0,083494
3555	96,00	170,50	33,023	118,00	33,10	35,200	6,30277	0,087795
3556	101,30	180,00	31,265	118,00	33,10	33,666	5,51400	0,088621
3557	90,10	173,00	30,105	100,00	33,20	32,596	4,94644	0,078564
3558	128,70	182,00	38,854	123,00	33,20	41,282	5,64878	0,079478
3559	89,70	168,00	31,781	105,00	33,20	34,489	5,66185	0,080739
3560	108,90	174,50	35,763	119,00	33,20	38,578	6,01683	0,082990
3561	84,80	168,00	30,045	104,00	33,20	31,520	5,59491	0,083022
3562	83,90	166,00	30,447	107,00	33,20	31,658	5,96953	0,085172
3563	84,40	165,00	31,001	109,00	33,20	33,617	6,19781	0,085987
3564	96,40	172,00	32,585	104,00	33,30	34,925	5,27504	0,077730
3565	108,40	178,00	34,213	110,00	33,30	36,458	5,19270	0,078233
3566	86,50	169,00	30,286	99,00	33,30	32,535	5,18229	0,078378
3567	81,50	160,00	31,836	100,00	33,30	34,312	6,01350	0,078704
3568	102,70	171,00	35,122	112,00	33,30	37,863	5,86976	0,079861
3569	105,50	180,50	32,382	110,00	33,30	34,469	5,01252	0,080591
3570	81,30	159,00	32,159	103,00	33,30	34,674	6,33130	0,080775
3571	88,80	166,00	32,225	106,00	33,30	35,131	5,90091	0,081243
3572	82,60	163,00	31,089	104,00	33,30	33,380	6,02879	0,082389
3573	77,00	160,00	30,078	102,00	33,30	32,657	6,16129	0,083376
3574	96,70	172,00	32,687	115,00	33,30	34,597	5,97754	0,085774
3575	105,70	183,50	31,391	119,00	33,30	34,137	5,31025	0,088280
3576	104,00	171,00	35,566	102,00	33,40	37,748	5,22378	0,072123
3577	127,40	179,00	39,762	118,00	33,40	42,258	5,59108	0,075709
3578	110,40	174,00	36,465	116,00	33,40	39,071	5,87199	0,079972
3579	107,90	181,00	32,936	112,00	33,40	35,749	5,09254	0,081022
3580	82,90	165,00	30,450	102,00	33,40	31,928	5,71158	0,081433
3581	106,50	173,00	35,584	116,00	33,40	38,625	5,95598	0,081520
3582	106,10	180,00	32,747	112,00	33,40	34,520	5,16441	0,081558
3583	105,70	176,00	34,123	114,00	33,40	36,555	5,58634	0,081680
3584	82,40	164,00	30,637	103,00	33,40	33,380	5,86849	0,082146
3585	101,50	175,00	33,143	114,00	33,40	35,972	5,66603	0,083520
3586	78,80	160,00	30,781	104,00	33,40	33,455	6,30914	0,083711
3587	92,50	168,00	32,774	113,00	33,40	35,575	6,19783	0,085129
3588	88,10	168,00	31,215	100,00	33,50	33,774	5,32727	0,077823
3589	96,00	177,00	30,643	103,00	33,50	31,727	4,84547	0,079062
3590	114,80	181,00	35,042	114,00	33,50	37,060	5,20773	0,079130
3591	93,80	174,00	30,982	103,00	33,50	33,171	5,06110	0,079158
3592	93,50	165,00	34,343	108,00	33,50	37,049	6,12831	0,079577
3593	75,00	157,00	30,427	98,00	33,50	32,255	6,14510	0,080248
3594	89,40	172,00	30,219	108,00	33,50	32,547	5,53033	0,084880

3595	85,70	166,00	31,100	110,00	33,50	33,561	6,17547	0,086330
3596	104,70	170,00	36,228	111,00	33,60	39,155	5,88978	0,077756
3597	99,00	176,00	31,960	104,00	33,60	33,669	4,97699	0,077840
3598	99,70	166,00	36,181	110,00	33,60	38,891	6,17547	0,078046
3599	100,80	168,00	35,714	111,00	33,60	38,416	6,06376	0,078966
3600	88,50	169,00	30,986	102,00	33,60	33,431	5,38055	0,079532
3601	93,90	172,00	31,740	105,00	33,60	33,965	5,33885	0,079864
3602	99,40	174,00	32,831	109,00	33,60	35,492	5,43513	0,080592
3603	95,00	168,00	33,659	109,00	33,60	36,048	5,92974	0,080668
3604	106,70	170,00	36,920	118,00	33,60	39,632	6,34804	0,081623
3605	118,00	176,00	38,094	123,00	33,60	40,776	6,13564	0,081893
3606	95,60	172,00	32,315	111,00	33,60	33,784	5,72192	0,083424
3607	96,80	171,00	33,104	114,00	33,60	35,789	5,99909	0,084557
3608	94,30	175,00	30,792	101,00	33,70	33,295	4,86484	0,077716
3609	93,70	170,00	32,422	107,00	33,70	34,440	5,62819	0,080711
3610	101,00	168,00	35,785	115,00	33,70	38,544	6,33195	0,081704
3611	92,70	168,00	32,844	110,00	33,70	35,288	5,99674	0,082749
3612	111,30	176,00	35,931	121,00	33,70	38,693	6,01350	0,083762
3613	87,70	162,00	33,417	101,00	33,80	36,403	5,90428	0,076486
3614	117,00	172,00	39,548	122,00	33,80	42,304	6,42530	0,080139
3615	97,60	173,00	32,611	108,00	33,80	34,657	5,45086	0,080444
3616	106,00	179,00	33,083	112,00	33,80	36,050	5,23750	0,081232
3617	97,20	173,00	32,477	109,00	33,80	34,953	5,51396	0,081412
3618	95,30	174,50	31,297	107,00	33,80	33,784	5,27224	0,081561
3619	105,00	175,00	34,286	114,00	33,80	37,116	5,66603	0,081654
3620	118,00	182,00	35,624	121,00	33,80	38,038	5,53471	0,082843
3621	95,70	170,00	33,114	114,00	33,80	35,870	6,08611	0,084789
3622	98,60	176,00	31,831	116,00	33,80	34,623	5,70833	0,087056
3623	85,00	164,00	31,603	103,00	33,90	33,485	5,86849	0,080463
3624	97,00	175,00	31,673	108,00	33,90	34,049	5,29603	0,081553
3625	140,00	189,00	39,193	130,00	33,90	41,646	5,50898	0,081955
3626	89,20	162,00	33,989	110,00	33,90	36,869	6,55329	0,082365
3627	92,00	167,50	32,791	110,00	33,90	35,448	6,04082	0,082962
3628	117,20	180,00	36,173	122,00	33,90	38,502	5,74726	0,083138
3629	121,00	183,00	36,131	123,00	33,90	38,404	5,57232	0,083194
3630	96,30	176,00	31,089	110,00	33,90	33,716	5,34247	0,083863
3631	85,10	168,00	30,152	109,00	33,90	31,924	5,92974	0,086809
3632	86,00	164,00	31,975	113,00	33,90	34,859	6,57211	0,087589
3633	95,20	177,00	30,387	114,00	33,90	32,475	5,50801	0,087995
3634	96,90	160,00	37,852	109,00	34,00	40,683	6,67905	0,076438
3635	86,10	167,00	30,872	103,00	34,00	33,644	5,61079	0,080990
3636	103,00	175,00	33,633	112,00	34,00	35,951	5,54265	0,081257
3637	110,20	183,00	32,906	113,00	34,00	35,573	5,00868	0,081345
3638	102,00	174,00	33,690	112,00	34,00	35,812	5,62229	0,081397
3639	91,50	167,00	32,809	108,00	34,00	35,613	5,94966	0,081547
3640	86,80	170,00	30,035	103,00	34,00	32,241	5,36678	0,081758
3641	93,20	172,50	31,321	107,00	34,00	33,068	5,42696	0,081991
3642	95,20	165,00	34,968	115,00	34,00	37,401	6,61509	0,083723
3643	91,80	172,00	31,030	110,00	34,00	33,650	5,65805	0,084939
3644	94,80	167,00	33,992	117,00	34,00	36,609	6,56043	0,086280

3645	99,00	179,00	30,898	101,00	34,10	33,159	4,59016	0,076667
3646	100,00	178,00	31,562	104,00	34,10	33,679	4,83553	0,078052
3647	112,80	183,00	33,683	114,00	34,10	35,668	5,06501	0,080799
3648	98,40	179,50	30,540	107,00	34,10	32,038	4,90808	0,081741
3649	81,10	159,50	31,879	104,00	34,10	34,518	6,35744	0,081907
3650	95,60	167,00	34,279	112,00	34,10	36,916	6,22098	0,082132
3651	108,30	174,00	35,771	107,00	34,20	38,530	5,31041	0,074718
3652	97,80	169,00	34,242	107,00	34,20	36,298	5,71123	0,078054
3653	112,80	188,00	31,915	108,00	34,20	34,558	4,40835	0,078286
3654	105,90	170,60	36,386	113,00	34,20	39,205	5,96873	0,078789
3655	96,00	167,00	34,422	108,00	34,20	36,815	5,94966	0,078978
3656	111,10	175,00	36,278	116,00	34,20	38,612	5,78945	0,080017
3657	101,10	177,00	32,270	110,00	34,20	34,341	5,26694	0,081571
3658	112,20	180,00	34,630	119,00	34,20	37,163	5,57230	0,083486
3659	102,00	177,50	32,375	113,00	34,20	34,914	5,40941	0,083498
3660	99,40	169,00	34,803	116,00	34,20	37,041	6,30722	0,083709
3661	98,00	169,00	34,313	117,00	34,20	37,272	6,37350	0,085233
3662	82,50	163,00	31,051	110,00	34,20	32,576	6,45619	0,087213
3663	103,00	175,00	33,633	101,00	34,30	35,679	4,86484	0,073276
3664	92,10	164,50	34,035	110,00	34,30	36,984	6,31388	0,081663
3665	109,00	180,00	33,642	119,00	34,30	36,097	5,57230	0,085112
3666	90,90	164,00	33,797	116,00	34,30	36,801	6,78347	0,086653
3667	95,00	167,00	34,064	102,00	34,40	37,079	5,54305	0,075113
3668	97,20	171,00	33,241	108,00	34,40	35,796	5,61123	0,079887
3669	100,00	175,00	32,653	110,00	34,40	34,484	5,41932	0,081394
3670	108,00	175,50	35,065	117,00	34,40	37,145	5,81008	0,082439
3671	94,50	173,50	31,393	111,00	34,40	33,729	5,59987	0,084681
3672	89,20	171,00	30,505	110,00	34,40	31,819	5,74047	0,086161
3673	103,00	177,00	32,877	119,00	34,40	35,686	5,80957	0,087157
3674	97,90	176,00	31,605	116,00	34,40	34,132	5,70833	0,087471
3675	82,90	164,00	30,822	98,00	34,50	33,157	5,51719	0,077844
3676	102,50	173,00	34,248	108,00	34,50	36,754	5,45086	0,077860
3677	95,00	173,00	31,742	103,00	34,50	34,206	5,13552	0,078114
3678	90,20	167,00	32,343	104,00	34,50	34,424	5,67854	0,079279
3679	92,30	172,00	31,199	105,00	34,50	33,423	5,33885	0,080785
3680	86,10	165,00	31,625	104,00	34,50	33,752	5,85044	0,080960
3681	98,40	177,00	31,409	108,00	34,50	33,337	5,14647	0,081546
3682	82,00	164,00	30,488	102,00	34,50	33,035	5,79820	0,081613
3683	95,00	168,00	33,659	111,00	34,50	36,251	6,06376	0,082148
3684	92,10	166,00	33,423	110,00	34,50	36,340	6,17547	0,082283
3685	98,40	178,00	31,057	111,00	34,50	33,511	5,25226	0,084206
3686	107,80	168,00	38,194	126,00	34,50	40,645	7,07051	0,085713
3687	93,40	169,00	32,702	114,00	34,50	35,644	6,17469	0,085752
3688	86,40	166,00	31,354	111,00	34,50	34,096	6,24415	0,086644
3689	98,10	170,00	33,945	124,00	34,50	36,757	6,74129	0,090716
3690	131,00	190,00	36,288	113,00	34,60	38,300	4,54868	0,074793
3691	117,90	180,00	36,389	118,00	34,60	39,261	5,51400	0,080094
3692	97,00	167,00	34,781	111,00	34,60	37,631	6,15313	0,080613
3693	121,40	179,00	37,889	123,00	34,60	40,346	5,88601	0,081496
3694	105,30	181,00	32,142	111,00	34,60	34,115	5,03496	0,081615

3695	88,00	169,00	30,811	105,00	34,60	33,317	5,57892	0,082181
3696	88,00	161,00	33,949	112,00	34,60	36,233	6,79846	0,084188
3697	100,00	179,00	31,210	112,00	34,60	33,936	5,23750	0,084449
3698	100,20	172,00	33,870	117,00	34,60	35,880	6,10541	0,085221
3699	92,80	170,50	31,923	112,00	34,60	34,802	5,91230	0,085236
3700	87,70	167,00	31,446	117,00	34,60	34,280	6,56043	0,090876
3701	94,90	168,00	33,624	102,00	34,70	36,620	5,46107	0,075541
3702	99,20	177,50	31,486	105,00	34,70	34,161	4,93029	0,079040
3703	110,10	176,00	35,544	115,00	34,70	38,125	5,64733	0,080186
3704	96,50	169,00	33,787	111,00	34,70	36,724	5,97599	0,081698
3705	91,70	174,00	30,288	105,00	34,70	32,448	5,18574	0,081922
3706	96,50	170,00	33,391	111,00	34,70	36,099	5,88978	0,082100
3707	92,40	169,00	32,352	109,00	34,70	34,889	5,84358	0,082582
3708	108,90	180,00	33,611	108,00	34,80	36,267	4,93153	0,077292
3709	92,80	171,00	31,736	104,00	34,80	33,402	5,35288	0,079341
3710	128,10	190,00	35,485	120,00	34,80	38,468	4,91412	0,080620
3711	89,60	167,00	32,127	107,00	34,80	35,025	5,88186	0,081930
3712	89,00	161,00	34,335	110,00	34,80	37,295	6,65224	0,082064
3713	99,40	172,00	33,599	113,00	34,80	36,527	5,84971	0,082749
3714	96,70	175,00	31,576	110,00	34,80	32,967	5,41932	0,083235
3715	92,10	172,00	31,132	110,00	34,80	33,513	5,65805	0,084754
3716	118,20	174,00	39,041	133,00	34,80	41,925	6,93515	0,087612
3717	106,20	180,00	32,778	103,00	34,90	35,068	4,64065	0,074957
3718	103,60	170,50	35,638	107,00	34,90	38,337	5,58722	0,075668
3719	113,00	170,00	39,100	114,00	34,90	41,265	6,08611	0,075897
3720	108,00	166,00	39,193	113,00	34,90	41,813	6,38154	0,076013
3721	97,10	174,00	32,072	108,00	34,90	34,266	5,37277	0,081109
3722	100,60	176,00	32,477	110,00	34,90	34,278	5,34247	0,081456
3723	100,40	174,00	33,162	111,00	34,90	34,861	5,55989	0,081525
3724	89,70	172,00	30,320	104,00	34,90	32,755	5,27504	0,081554
3725	99,50	168,00	35,254	115,00	35,00	37,823	6,33195	0,082523
3726	102,50	181,00	31,287	113,00	35,00	33,690	5,15013	0,084592
3727	112,00	176,00	36,157	127,00	35,00	38,703	6,38004	0,087549
3728	94,80	168,50	33,389	102,00	35,10	36,374	5,42063	0,075781
3729	106,00	171,00	36,250	115,00	35,10	39,123	6,06378	0,080289
3730	89,10	167,50	31,758	105,00	35,10	33,746	5,70388	0,080900
3731	107,30	184,50	31,522	111,00	35,10	33,713	4,79487	0,081894
3732	96,20	169,00	33,682	115,00	35,10	36,141	6,24095	0,084818
3733	92,40	173,00	30,873	110,00	35,10	33,644	5,57707	0,084980
3734	82,00	159,00	32,435	109,00	35,10	35,283	6,78087	0,084993
3735	92,10	167,00	33,024	116,00	35,10	35,844	6,49251	0,087206
3736	110,00	172,00	37,182	130,00	35,10	39,990	6,93771	0,088979
3737	113,60	177,00	36,260	110,00	35,20	38,863	5,26694	0,075472
3738	111,50	180,00	34,414	112,00	35,20	36,996	5,16441	0,078903
3739	106,00	179,50	32,899	110,00	35,20	35,348	5,08368	0,079967
3740	107,10	172,00	36,202	118,00	35,20	38,750	6,16936	0,082217
3741	110,60	178,00	34,907	118,00	35,20	37,579	5,66948	0,082806
3742	86,30	167,00	30,944	107,00	35,20	32,531	5,88186	0,084005
3743	106,90	172,00	36,134	122,00	35,20	39,007	6,42530	0,085110
3744	90,80	171,50	30,871	113,00	35,20	33,643	5,89188	0,087682

3745	96,90	175,00	31,641	103,00	35,30	33,931	4,98798	0,077831
3746	88,60	163,50	33,143	105,00	35,30	35,548	6,05433	0,079585
3747	89,40	170,00	30,934	106,00	35,30	32,945	5,56282	0,082500
3748	82,80	163,00	31,164	107,00	35,30	33,048	6,24242	0,084629
3749	94,00	172,00	31,774	112,00	35,30	34,004	5,78581	0,085128
3750	83,70	163,00	31,503	111,00	35,30	33,692	6,52747	0,087162
3751	104,40	170,00	36,125	115,00	35,40	39,107	6,15157	0,080712
3752	107,90	171,00	36,900	117,00	35,40	39,459	6,19319	0,080724
3753	93,60	170,00	32,388	108,00	35,40	35,004	5,69357	0,081523
3754	105,40	176,50	33,834	117,00	35,40	36,239	5,72895	0,084187
3755	106,80	179,00	33,332	121,00	35,40	36,314	5,76801	0,087320
3756	96,30	170,00	33,322	120,00	35,40	36,094	6,47907	0,088880
3757	111,30	174,00	36,762	114,00	35,50	39,474	5,74712	0,078169
3758	107,90	172,50	36,261	117,00	35,50	38,703	6,06210	0,081313
3759	103,10	172,00	34,850	114,00	35,50	37,580	5,91362	0,081472
3760	88,20	162,00	33,608	109,00	35,50	35,719	6,48112	0,082232
3761	92,40	171,00	31,599	110,00	35,50	33,884	5,74047	0,084160
3762	101,80	178,00	32,130	114,00	35,50	35,028	5,43101	0,084545
3763	95,00	168,00	33,659	116,00	35,50	36,658	6,39903	0,085849
3764	102,00	179,00	31,834	105,00	35,60	33,914	4,82542	0,078133
3765	92,90	166,50	33,511	107,00	35,60	36,433	5,92549	0,079778
3766	99,50	176,00	32,122	113,00	35,60	34,324	5,52536	0,084293
3767	103,90	178,00	32,793	116,00	35,60	35,668	5,55023	0,084865
3768	107,30	172,50	36,060	122,00	35,60	38,768	6,38010	0,085104
3769	94,60	167,00	33,920	116,00	35,60	36,532	6,49251	0,085663
3770	94,90	167,00	34,028	117,00	35,60	35,992	6,56043	0,086219
3771	93,60	171,00	32,010	114,00	35,60	34,351	5,99909	0,086474
3772	111,60	174,00	36,861	118,00	35,70	39,624	5,99690	0,080766
3773	98,00	173,00	32,744	112,00	35,70	35,176	5,70333	0,083197
3774	89,00	166,00	32,298	110,00	35,70	34,753	6,17547	0,084183
3775	110,30	171,00	37,721	124,00	35,70	40,227	6,64647	0,084308
3776	116,60	178,00	36,801	125,00	35,70	39,718	6,08719	0,084683
3777	92,70	173,50	30,795	110,00	35,70	33,471	5,53712	0,085001
3778	92,10	169,00	32,247	112,00	35,70	34,084	6,04221	0,085039
3779	104,90	176,50	33,673	118,00	35,70	36,402	5,78962	0,085176
3780	98,60	167,00	35,354	124,00	35,70	37,519	7,03620	0,089077
3781	113,20	185,00	33,075	135,00	35,70	34,974	6,08582	0,096327
3782	104,30	175,50	33,863	107,00	35,80	36,604	5,19688	0,077166
3783	86,40	164,00	32,124	100,00	35,80	34,326	5,65767	0,077273
3784	116,30	171,00	39,773	118,00	35,80	42,149	6,25791	0,077445
3785	114,70	179,00	35,798	118,00	35,80	38,445	5,59108	0,081199
3786	101,30	177,00	32,334	115,00	35,80	34,566	5,56830	0,085167
3787	110,00	179,00	34,331	122,00	35,80	36,652	5,82700	0,086326
3788	88,60	166,00	32,153	113,00	35,80	34,744	6,38154	0,086739
3789	131,10	190,00	36,316	121,00	35,90	38,759	4,96636	0,080047
3790	109,00	175,00	35,592	123,00	35,90	38,176	6,22175	0,085932
3791	115,00	181,00	35,103	127,00	35,90	38,082	5,95735	0,088052
3792	103,80	173,00	34,682	126,00	35,90	37,715	6,58839	0,090076
3793	102,50	171,50	34,849	111,00	36,00	36,847	5,76333	0,079444
3794	113,00	184,00	33,377	119,00	36,00	35,181	5,27405	0,084627

3795	112,00	178,00	35,349	123,00	36,00	37,455	5,96780	0,085594
3796	92,20	173,50	30,629	113,00	36,00	33,284	5,72541	0,087634
3797	105,60	172,00	35,695	109,00	36,10	38,736	5,59419	0,076664
3798	94,60	172,00	31,977	103,00	36,10	34,470	5,21125	0,077956
3799	94,40	161,00	36,418	109,00	36,10	38,971	6,57915	0,078187
3800	89,10	169,00	31,196	101,00	36,10	33,921	5,31445	0,078399
3801	94,00	175,00	30,694	102,00	36,10	33,183	4,92640	0,078653
3802	103,00	182,00	31,095	110,00	36,10	33,724	4,90800	0,082457
3803	118,70	174,00	39,206	128,00	36,10	41,492	6,62213	0,084082
3804	88,60	169,00	31,021	109,00	36,10	33,556	5,84358	0,084926
3805	122,70	178,00	38,726	132,00	36,10	41,637	6,50538	0,086436
3806	98,00	164,00	36,437	112,00	36,20	39,202	6,50168	0,079574
3807	90,00	168,00	31,888	106,00	36,20	34,292	5,72880	0,081327
3808	110,30	183,00	32,936	114,00	36,20	35,029	5,06501	0,082016
3809	93,50	174,00	30,883	107,00	36,20	33,227	5,31041	0,082408
3810	93,00	169,00	32,562	110,00	36,20	35,196	5,90978	0,082981
3811	108,20	174,00	35,738	119,00	36,20	38,722	6,05938	0,083148
3812	118,90	176,00	38,385	133,00	36,20	41,233	6,74696	0,088103
3813	89,90	164,00	33,425	121,00	36,20	36,135	7,13600	0,091057
3814	91,90	174,00	30,354	95,00	36,30	31,810	4,56301	0,074012
3815	114,90	170,00	39,758	117,00	36,30	42,493	6,28254	0,077034
3816	88,40	170,00	30,588	100,00	36,30	32,273	5,17086	0,078416
3817	104,80	175,50	34,026	110,00	36,30	36,646	5,38073	0,079077
3818	97,30	177,00	31,057	105,00	36,30	32,837	4,96584	0,079878
3819	100,00	173,00	33,412	110,00	36,30	36,329	5,57707	0,080618
3820	95,40	173,00	31,875	107,00	36,30	34,514	5,38777	0,080920
3821	113,70	171,00	38,884	123,00	36,30	41,097	6,58168	0,081952
3822	102,00	175,00	33,306	113,00	36,30	35,728	5,60434	0,082517
3823	100,00	172,00	33,802	114,00	36,30	36,271	5,91362	0,083147
3824	97,60	171,00	33,378	114,00	36,30	36,154	5,99909	0,084095
3825	101,00	174,00	33,360	115,00	36,30	35,926	5,80955	0,084128
3826	98,80	176,00	31,896	113,00	36,30	34,773	5,52536	0,084690
3827	97,90	172,00	33,092	125,00	36,30	36,060	6,61736	0,092469
3828	98,40	172,00	33,261	108,00	36,40	35,888	5,53033	0,079622
3829	93,00	168,00	32,951	108,00	36,40	35,478	5,86275	0,081070
3830	94,00	172,00	31,774	107,00	36,40	33,924	5,46649	0,081328
3831	98,10	172,00	33,160	116,00	36,40	35,990	6,04147	0,085694
3832	119,20	179,00	37,202	117,00	36,50	39,662	5,53213	0,078472
3833	109,00	176,00	35,189	112,00	36,50	37,103	5,46438	0,078619
3834	109,00	169,00	38,164	117,00	36,50	40,889	6,37350	0,079398
3835	138,00	188,00	39,045	126,00	36,50	41,591	5,36857	0,079845
3836	96,10	170,50	33,058	111,00	36,50	35,596	5,84726	0,082530
3837	95,20	168,00	33,730	105,00	36,60	36,126	5,66185	0,077599
3838	99,00	173,00	33,078	110,00	36,60	35,689	5,57707	0,081160
3839	96,00	168,00	34,014	113,00	36,60	36,764	6,19783	0,083047
3840	91,30	171,00	31,223	109,00	36,60	33,534	5,67584	0,084064
3841	97,80	168,00	34,651	102,00	36,70	37,186	5,46107	0,074040
3842	99,40	169,00	34,803	106,00	36,70	37,225	5,64507	0,076493
3843	108,30	173,50	35,977	111,00	36,70	38,629	5,59987	0,077325
3844	102,00	173,00	34,081	107,00	36,70	36,443	5,38777	0,077391

3845	109,60	171,00	37,482	114,00	36,70	39,942	5,99909	0,077839
3846	103,50	172,00	34,985	110,00	36,70	37,299	5,65805	0,078410
3847	102,50	178,00	32,351	108,00	36,70	35,039	5,07360	0,079731
3848	100,00	172,00	33,802	117,00	36,70	36,271	6,10541	0,085335
3849	101,00	180,00	31,173	116,00	36,70	33,560	5,39743	0,087291
3850	118,90	180,00	36,698	118,00	36,80	38,995	5,51400	0,079644
3851	104,70	176,00	33,800	112,00	36,80	35,534	5,46438	0,080757
3852	94,80	172,00	32,044	107,00	36,80	34,935	5,46649	0,080870
3853	122,80	179,00	38,326	124,00	36,80	40,773	5,94502	0,081533
3854	101,00	175,00	32,980	112,00	36,80	35,510	5,54265	0,082326
3855	106,30	176,00	34,317	110,00	36,90	36,573	5,34247	0,078517
3856	103,10	170,00	35,675	116,00	36,90	38,262	6,21705	0,082097
3857	101,90	172,00	34,444	114,00	36,90	37,471	5,91362	0,082110
3858	104,00	171,00	35,566	117,00	36,90	38,378	6,19319	0,082729
3859	92,00	171,00	31,463	102,00	37,00	33,727	5,22378	0,078266
3860	106,20	174,00	35,077	115,00	37,00	37,757	5,80955	0,081359
3861	105,50	169,00	36,938	120,00	37,00	39,396	6,57242	0,083225
3862	112,70	175,00	36,800	123,00	37,00	39,152	6,22175	0,084040
3863	90,00	166,00	32,661	113,00	37,00	34,862	6,38154	0,085837
3864	111,70	183,00	33,354	123,00	37,00	36,337	5,57232	0,087750
3865	111,50	172,50	37,471	109,00	37,10	39,641	5,55390	0,074114
3866	91,20	173,00	30,472	101,00	37,10	31,601	5,00946	0,078710
3867	105,00	170,00	36,332	114,00	37,10	38,401	6,08611	0,079705
3868	111,50	179,00	34,799	115,00	37,10	37,711	5,41425	0,080642
3869	97,30	169,00	34,067	116,00	37,10	36,167	6,30722	0,084909
3870	94,50	167,00	33,884	118,00	37,10	35,830	6,62836	0,087202
3871	99,60	175,00	32,522	108,00	37,20	34,854	5,29603	0,080128
3872	94,60	174,00	31,246	109,00	37,20	32,978	5,43513	0,083296
3873	112,00	176,50	35,952	123,00	37,20	38,548	6,09313	0,084992
3874	88,70	168,00	31,427	111,00	37,20	34,013	6,06376	0,085993
3875	97,20	167,00	34,852	120,00	37,20	37,766	6,76425	0,087030
3876	94,10	166,00	34,149	105,00	37,30	36,712	5,83230	0,077426
3877	84,00	164,00	31,231	104,00	37,40	33,793	5,93879	0,081887
3878	110,50	170,00	38,235	122,00	37,40	40,368	6,61016	0,082444
3879	93,30	170,00	32,284	110,00	37,40	34,813	5,82437	0,083211
3880	105,20	171,00	35,977	122,00	37,40	38,905	6,51690	0,085608
3881	94,20	172,00	31,842	114,00	37,40	34,477	5,91362	0,086526
3882	108,50	178,00	34,244	108,00	37,50	37,008	5,07360	0,076763
3883	110,50	180,00	34,105	111,00	37,50	36,274	5,10618	0,078670
3884	90,30	172,00	30,523	103,00	37,50	31,753	5,21125	0,080412
3885	121,50	175,00	39,673	126,00	37,50	42,016	6,40718	0,081881
3886	102,60	176,00	33,122	113,00	37,50	35,666	5,52536	0,082586
3887	100,90	170,00	34,913	117,00	37,50	37,282	6,28254	0,084004
3888	97,70	169,00	34,207	117,00	37,50	36,840	6,37350	0,085407
3889	107,60	171,50	36,583	124,00	37,50	39,453	6,59970	0,085921
3890	100,00	168,00	35,431	111,00	37,60	37,892	6,06376	0,079387
3891	103,90	174,00	34,318	114,00	37,60	36,829	5,74712	0,081837
3892	96,40	169,00	33,752	106,00	37,70	36,016	5,64507	0,078072
3893	84,30	164,00	31,343	101,00	37,70	34,166	5,72793	0,079336
3894	89,50	170,00	30,969	107,00	37,70	33,666	5,62819	0,083216

3895	104,70	168,00	37,096	112,00	37,80	39,255	6,13079	0,077686
3896	117,40	179,00	36,641	118,00	37,80	39,562	5,59108	0,079949
3897	89,00	169,00	31,161	107,00	37,80	33,965	5,71123	0,083118
3898	116,10	180,00	35,833	110,00	37,90	38,425	5,04795	0,075434
3899	107,30	178,00	33,866	107,00	37,90	36,474	5,01407	0,076619
3900	110,60	175,00	36,114	113,00	37,90	38,496	5,60434	0,078182
3901	115,10	179,00	35,923	110,00	38,00	38,629	5,11971	0,075518
3902	106,00	172,00	35,830	117,00	38,00	38,366	6,10541	0,082083
3903	107,00	175,00	34,939	118,00	38,00	38,097	5,91291	0,083462
3904	106,00	178,00	33,455	116,00	38,00	36,306	5,55023	0,083741
3905	90,60	168,00	32,100	105,00	38,10	34,841	5,66185	0,080204
3906	100,80	176,00	32,541	109,00	38,10	35,248	5,28153	0,080608
3907	107,00	171,00	36,592	117,00	38,10	38,886	6,19319	0,081176
3908	95,20	174,00	31,444	109,00	38,10	32,970	5,43513	0,082946
3909	114,30	174,00	37,753	117,00	38,20	40,731	5,93444	0,078816
3910	115,40	174,00	38,116	124,00	38,20	40,340	6,37191	0,083000
3911	96,80	166,00	35,128	115,00	38,20	38,108	6,51898	0,083216
3912	88,80	165,00	32,617	117,00	38,20	35,553	6,75430	0,089224
3913	90,10	168,00	31,923	102,00	38,30	34,724	5,46107	0,078200
3914	101,40	179,00	31,647	118,00	38,30	34,099	5,59108	0,088152
3915	96,30	162,00	36,694	111,00	38,40	39,666	6,62548	0,078977
3916	105,50	175,00	34,449	113,00	38,40	36,338	5,60434	0,080682
3917	115,00	176,00	37,126	134,00	38,40	39,235	6,80815	0,090761
3918	114,60	181,00	34,981	106,00	38,60	36,932	4,74720	0,073663
3919	107,30	178,00	33,866	117,00	38,60	36,540	5,60985	0,083779
3920	96,80	170,00	33,495	116,00	38,60	36,279	6,21705	0,085621
3921	100,80	171,00	34,472	113,00	38,70	36,869	5,93442	0,081583
3922	80,20	161,00	30,940	105,00	38,70	33,123	6,28694	0,083964
3923	96,50	165,00	35,445	120,00	38,70	37,849	6,96321	0,086576
3924	106,00	169,50	36,895	115,00	38,80	39,097	6,19606	0,079702
3925	90,50	171,00	30,950	105,00	38,80	33,645	5,41745	0,081455
3926	87,00	157,00	35,296	111,00	38,80	38,101	7,14438	0,082330
3927	113,00	178,00	35,665	113,00	38,90	37,853	5,37142	0,078170
3928	91,90	175,00	30,008	100,00	38,90	31,842	4,80328	0,078281
3929	83,00	165,00	30,487	97,00	39,00	32,680	5,36469	0,077379
3930	103,70	176,00	33,478	111,00	39,00	35,710	5,40342	0,080550
3931	92,00	166,00	33,387	110,00	39,00	36,094	6,17547	0,082343
3932	102,00	168,00	36,139	117,00	39,00	39,176	6,46612	0,082580
3933	101,30	172,00	34,241	117,00	39,00	36,941	6,10541	0,084603
3934	100,00	167,00	35,856	119,00	39,00	38,729	6,69630	0,084686
3935	107,30	176,00	34,640	120,00	39,00	36,801	5,95244	0,085122
3936	125,30	179,00	39,106	120,00	39,10	41,775	5,70902	0,077850
3937	98,00	170,00	33,910	108,00	39,10	36,389	5,69357	0,079064
3938	95,80	164,00	35,619	119,00	39,10	38,432	6,99495	0,085837
3939	103,70	170,00	35,882	120,00	39,20	38,866	6,47907	0,084600
3940	74,10	150,00	32,933	94,00	39,30	35,603	6,52742	0,074701
3941	99,40	175,00	32,457	109,00	39,30	35,006	5,35767	0,080978
3942	92,00	171,50	31,279	109,00	39,30	33,765	5,63483	0,083841
3943	122,00	182,00	36,831	132,00	39,30	39,851	6,16252	0,088388
3944	97,30	170,00	33,668	108,00	39,40	36,599	5,69357	0,079443

3945	113,00	172,00	38,196	123,00	39,40	40,374	6,48931	0,082691
3946	117,00	176,00	37,771	125,00	39,40	40,654	6,25782	0,083698
3947	84,30	167,00	30,227	97,00	39,50	32,376	5,20457	0,077354
3948	115,90	174,00	38,281	128,00	39,70	41,136	6,62213	0,085430
3949	110,00	168,00	38,974	124,00	39,80	41,567	6,93611	0,083224
3950	83,00	164,00	30,860	99,00	39,90	32,771	5,58742	0,078575
3951	78,30	160,50	30,396	98,00	39,90	32,396	5,82074	0,079423
3952	103,00	168,00	36,494	115,00	39,90	39,153	6,33195	0,080643
3953	108,00	175,00	35,265	121,00	39,90	38,247	6,09818	0,085055
3954	87,80	170,00	30,381	94,00	40,00	32,735	4,77932	0,074047
3955	117,10	172,00	39,582	123,00	40,00	42,213	6,48931	0,080750
3956	119,30	181,00	36,415	123,00	40,00	38,861	5,72653	0,083217
3957	108,90	177,00	34,760	120,00	40,00	36,871	5,86991	0,084685
3958	88,10	167,50	31,401	94,00	40,10	33,902	4,96374	0,072972
3959	116,00	175,00	37,878	124,00	40,10	40,007	6,28355	0,083109
3960	89,70	163,00	33,761	111,00	40,10	36,294	6,52747	0,083231
3961	109,00	171,50	37,059	125,00	40,10	39,669	6,66411	0,085871
3962	94,70	170,50	32,576	103,00	40,20	35,212	5,32738	0,077335
3963	106,90	179,00	33,364	120,00	40,20	36,277	5,70902	0,086545
3964	122,00	175,50	39,610	133,00	40,30	42,400	6,79339	0,086399
3965	102,00	174,00	33,690	121,00	40,30	36,014	6,18436	0,087938
3966	120,00	175,00	39,184	122,00	40,40	41,387	6,15996	0,079941
3967	107,00	175,00	34,939	118,00	40,40	37,188	5,91291	0,083462
3968	85,00	168,00	30,116	97,00	40,50	31,698	5,12667	0,077313
3969	116,60	183,00	34,817	112,00	40,50	37,485	4,95237	0,077648
3970	109,00	170,00	37,716	116,00	40,50	40,554	6,21705	0,079107
3971	121,70	176,00	39,288	126,00	40,50	41,654	6,31892	0,082181
3972	112,30	177,00	35,845	120,00	40,50	38,662	5,86991	0,082967
3973	96,30	178,00	30,394	102,00	40,60	32,483	4,71655	0,078499
3974	101,30	174,50	33,267	110,00	40,60	36,105	5,45824	0,080504
3975	118,00	179,00	36,828	118,00	40,70	38,873	5,59108	0,079678
3976	115,00	176,00	37,126	114,00	40,80	39,335	5,58634	0,077215
3977	120,50	177,00	38,463	118,00	40,80	40,950	5,74923	0,077840
3978	101,00	173,00	33,747	112,00	40,80	36,749	5,70333	0,081541
3979	112,00	176,00	36,157	123,00	40,80	38,649	6,13564	0,084792
3980	116,80	189,00	32,698	115,00	40,90	34,389	4,71675	0,081806
3981	104,40	171,00	35,703	117,00	40,90	38,121	6,19319	0,082518
3982	102,60	168,00	36,352	121,00	40,90	39,064	6,73461	0,085070
3983	108,00	180,00	33,333	102,00	41,00	35,131	4,58250	0,073403
3984	94,00	175,00	30,694	98,00	41,00	32,574	4,68020	0,075568
3985	96,00	172,00	32,450	102,00	41,00	35,299	5,14746	0,076447
3986	87,00	169,50	30,282	104,00	41,00	32,890	5,47227	0,082223
3987	107,70	171,00	36,832	114,00	41,10	38,929	5,99909	0,078751
3988	116,40	184,00	34,381	114,00	41,10	37,024	4,99540	0,079485
3989	129,00	183,00	38,520	125,00	41,30	40,829	5,68515	0,081014
3990	89,00	170,00	30,796	108,00	41,30	33,299	5,69357	0,084308
3991	106,00	172,00	35,830	110,00	41,40	38,478	5,65805	0,077172
3992	118,90	176,50	38,167	121,00	41,40	40,528	5,97170	0,080344
3993	107,20	172,00	36,236	110,00	41,50	39,000	5,65805	0,076595
3994	119,40	180,00	36,852	119,00	41,50	38,950	5,57230	0,080095

3995	107,30	175,50	34,837	119,00	41,60	37,567	5,93285	0,084213
3996	111,70	172,00	37,757	112,00	41,70	39,884	5,78581	0,075879
3997	90,70	170,00	31,384	104,00	41,80	34,212	5,43212	0,080168
3998	90,00	168,00	31,888	108,00	41,80	34,449	5,86275	0,082862
3999	88,10	160,00	34,414	109,00	41,90	37,440	6,67905	0,081447
4000	110,30	175,00	36,016	127,00	41,90	38,393	6,46902	0,088028
4001	101,00	177,00	32,239	106,00	42,00	34,457	5,02604	0,078657
4002	113,40	174,00	37,455	117,00	42,00	40,061	5,93444	0,079232
4003	96,00	170,00	33,218	107,00	42,00	36,053	5,62819	0,079416
4004	102,00	170,00	35,294	119,00	42,00	38,041	6,41355	0,084824
4005	82,60	163,00	31,089	104,00	42,10	32,790	6,02879	0,082389
4006	102,60	167,00	36,789	120,00	42,40	39,346	6,76425	0,083949
4007	101,50	181,00	30,982	103,00	42,50	32,747	4,57466	0,077611
4008	89,00	169,00	31,161	101,00	42,60	33,881	5,31445	0,078457
4009	96,00	163,00	36,132	114,00	42,60	38,515	6,74140	0,081698
4010	122,50	180,00	37,809	126,00	42,60	40,266	5,98066	0,083369
4011	108,90	178,00	34,371	129,00	42,60	37,141	6,32610	0,091465
4012	100,00	165,50	36,509	112,00	42,80	39,485	6,35940	0,079107
4013	97,60	168,00	34,580	114,00	42,80	36,986	6,26488	0,082863
4014	103,00	162,00	39,247	116,00	42,90	41,988	6,98662	0,078916
4015	110,90	174,00	36,630	128,00	42,99	39,655	6,62213	0,087979
4016	105,10	172,00	35,526	114,00	43,20	38,050	5,91362	0,080435
4017	90,00	170,00	31,142	106,00	43,20	32,938	5,56282	0,082133
4018	97,00	176,00	31,315	102,00	43,40	32,978	4,85524	0,077389
4019	115,70	179,00	36,110	117,00	43,60	39,092	5,53213	0,080046
4020	108,70	173,00	36,319	115,00	43,70	38,977	5,89280	0,079723
4021	93,00	167,00	33,346	106,00	43,80	35,842	5,81407	0,079174
4022	108,10	174,00	35,705	119,00	43,80	38,576	6,05938	0,083200
4023	98,00	179,00	30,586	110,00	43,80	31,743	5,11971	0,084066
4024	93,30	167,00	33,454	119,00	43,80	36,029	6,69630	0,088693
4025	96,00	172,00	32,450	102,00	44,00	35,073	5,14746	0,076447
4026	114,00	177,00	36,388	122,00	44,00	38,993	5,99062	0,083509
4027	99,40	162,00	37,875	124,00	44,00	40,706	7,56521	0,086383
4028	103,80	170,00	35,917	112,00	44,10	38,289	5,95521	0,078909
4029	116,80	178,00	36,864	119,00	44,20	39,422	5,72913	0,080526
4030	99,30	178,00	31,341	111,00	44,20	33,751	5,25226	0,083696
4031	113,20	187,00	32,372	108,00	44,30	33,928	4,47006	0,077755
4032	111,00	167,00	39,801	131,00	44,40	42,214	7,51260	0,086960
4033	102,10	180,00	31,512	103,00	44,60	33,703	4,64065	0,076951
4034	113,00	177,00	36,069	120,00	44,60	38,393	5,86991	0,082624
4035	105,50	167,50	37,603	120,00	44,70	40,541	6,71564	0,082609
4036	101,10	175,00	33,012	107,00	44,90	35,760	5,23439	0,078599
4037	100,30	164,00	37,292	124,00	44,90	39,949	7,34770	0,086748
4038	100,00	167,00	35,856	110,00	45,00	38,449	6,08529	0,078281
4039	93,80	161,00	36,187	111,00	45,00	39,114	6,72534	0,079961
4040	97,00	162,00	36,961	118,00	45,00	39,722	7,13118	0,083553
4041	108,00	178,00	34,087	118,00	45,00	36,515	5,66948	0,084130
4042	110,00	174,00	36,332	116,00	45,10	38,562	5,87199	0,080165
4043	77,00	158,00	30,844	103,00	45,10	33,612	6,42925	0,083315
4044	98,40	168,00	34,864	107,00	45,40	37,656	5,79577	0,077353

4045	122,00	180,00	37,654	121,00	45,40	39,971	5,68893	0,080280
4046	90,90	167,00	32,593	111,00	45,40	35,528	6,15313	0,084180
4047	102,30	174,00	33,789	109,00	45,50	36,458	5,43513	0,079062
4048	136,50	193,50	36,456	125,00	45,60	38,639	4,94123	0,081731
4049	104,50	173,50	34,715	112,00	46,00	36,636	5,66263	0,079902
4050	93,00	163,00	35,003	111,00	46,00	37,921	6,52747	0,081250
4051	94,00	170,00	32,526	109,00	46,00	34,933	5,75896	0,082044
4052	99,00	169,00	34,663	120,00	46,10	37,447	6,57242	0,086829
4053	133,00	184,50	39,071	138,00	46,40	41,869	6,29310	0,088235
4054	102,00	173,00	34,081	114,00	46,60	36,966	5,82964	0,082454
4055	101,30	165,00	37,208	118,00	47,00	40,213	6,82392	0,082422
4056	117,70	173,00	39,326	122,00	47,20	42,061	6,33529	0,080207
4057	124,20	181,00	37,911	123,00	47,40	40,414	5,72653	0,081013
4058	100,10	170,50	34,434	93,00	47,90	37,144	4,67859	0,067292
4059	88,00	165,00	32,323	105,00	50,50	35,237	5,91988	0,080557
4060	98,00	165,00	35,996	125,00	50,70	38,924	7,31166	0,089261
4061	109,00	171,00	37,276	129,00	51,00	39,983	6,97060	0,088403
4062	96,70	172,00	32,687	100,00	54,60	35,554	5,01993	0,074586
4063	93,30	164,50	34,479	119,00	58,00	37,002	6,94402	0,087585
4064	117,00	168,00	41,454	119,00	30,40	43,878	6,60034	0,076650
4065	132,80	172,00	44,889	142,00	32,70	46,712	7,70771	0,085723
4066	131,20	176,00	42,355	137,00	33,60	44,384	6,99178	0,084988
4067	129,80	178,00	40,967	134,00	35,90	43,020	6,62496	0,084516
4068	121,90	169,00	42,681	125,00	36,40	44,725	6,90420	0,078731
4069	123,80	163,00	46,596	140,00	36,70	48,039	8,60087	0,084685
4070	126,50	172,00	42,760	120,00	37,00	44,759	6,29731	0,074828
4071	117,20	171,00	40,081	127,00	37,20	42,705	6,84092	0,082924
4072	119,90	171,00	41,004	124,00	38,30	43,450	6,64647	0,079745
4073	118,90	170,00	41,142	120,00	38,40	43,250	6,47907	0,077227
4074	125,50	174,00	41,452	121,00	40,00	43,876	6,18436	0,076586
4075	120,20	171,00	41,107	124,00	40,10	43,719	6,64647	0,079613
4076	125,70	174,00	41,518	130,00	40,40	43,933	6,74730	0,082195
4077	121,40	173,00	40,563	126,00	41,00	43,022	6,58839	0,081145
4078	138,00	175,00	45,061	130,00	41,60	46,421	6,65458	0,077605
4079	123,00	170,00	42,561	125,00	42,20	44,430	6,80688	0,078647
4080	124,00	176,00	40,031	125,00	42,30	42,465	6,25782	0,080517
4081	116,30	170,00	40,242	119,00	43,10	42,582	6,41355	0,077720
4082	134,50	167,00	48,227	128,00	43,60	48,995	7,30835	0,074758
4083	117,00	168,50	41,208	135,00	44,00	43,629	7,62206	0,087172
4084	135,40	173,00	45,240	142,00	44,10	47,008	7,60258	0,085032
4085	120,90	171,00	41,346	128,00	44,40	43,959	6,90575	0,081863
4086	129,00	172,00	43,605	137,00	44,70	45,762	7,38667	0,084321
4087	138,00	177,00	44,049	141,00	44,80	45,938	7,13944	0,084972
4088	125,00	175,00	40,816	130,00	45,00	42,883	6,65458	0,082896
4089	132,00	176,00	42,614	131,00	46,00	44,829	6,62461	0,080937
4090	124,90	168,50	43,991	116,00	46,40	45,862	6,35292	0,071711
4091	128,50	172,00	43,436	132,00	46,50	45,383	7,06593	0,081454
4092	120,00	169,50	41,768	128,00	46,80	43,977	7,05334	0,081670
4093	106,30	159,00	42,047	132,00	48,00	43,952	8,50946	0,086573
4094	124,80	166,00	45,290	136,00	49,60	46,990	7,96532	0,083078

WHtR	TG(0_normal)	HDL(0_normal)	GLUCOSE(0_normal)	BLOOD PRESURE (0_normal)	ONE AND MORE(0_without mets components)
0,4464286	0	0	0	0	0
0,4000000	0	0	1	0	1
0,4127907	0	0	0	0	0
0,4195402	0	0	0	1	1
0,4340176	0	0	0	1	1
0,4540230	1	1	1	1	1
0,4393064	0	0	0	1	1
0,4069767	0	0	0	1	1
0,4529412	0	0	0	1	1
0,4047619	1	1	0	0	1
0,4523810	1	0	0	1	1
0,4475921	0	0	0	1	1
0,4198895	0	0	0	0	0
0,4355828	0	0	0	1	1
0,4093264	1	0	1	1	1
0,4464286	0	0	1	0	1
0,4450704	0	0	1	0	1
0,4318182	0	0	1	1	1
0,4593023	0	0	0	1	1
0,4540230	0	0	0	1	1
0,4261364	0	0	0	0	0
0,4252874	0	0	0	1	1
0,4619883	0	0	0	1	1
0,4588235	0	0	0	0	0
0,4488636	0	0	0	1	1
0,4382022	0	0	0	1	1
0,4478528	0	0	0	1	1
0,4109589	0	0	1	1	1
0,4444444	0	0	0	0	0
0,4574780	0	0	0	1	1
0,4294118	0	0	0	0	0
0,4302326	0	0	0	1	1
0,4269006	0	0	0	0	0
0,4306785	1	1	0	0	1
0,4588235	1	0	1	1	1
0,4268293	0	0	0	1	1
0,4831804	0	0	0	0	0
0,4318182	0	0	0	0	0
0,4277778	0	0	0	1	1
0,4534884	0	0	0	0	0
0,4475921	0	0	1	0	1
0,4514286	0	0	0	1	1
0,4186047	0	0	0	1	1
0,4364641	1	0	1	0	1

0,4756098	0	0	0	0	0
0,4261364	0	0	1	1	1
0,4385965	0	0	0	0	0
0,4450867	0	0	0	1	1
0,4634146	0	0	1	1	1
0,4391691	0	0	1	1	1
0,3966006	0	0	0	0	0
0,4451220	0	0	0	0	0
0,4411765	0	0	0	0	0
0,4573171	0	0	0	1	1
0,4660767	0	0	0	1	1
0,4593023	0	0	1	1	1
0,4727273	0	0	0	1	1
0,4444444	0	0	0	0	0
0,4527221	0	0	0	1	1
0,4268293	0	0	0	1	1
0,4840764	1	0	1	1	1
0,4670659	0	0	1	1	1
0,4615385	0	0	0	1	1
0,4400000	0	0	0	1	1
0,4364641	0	0	0	1	1
0,4787879	0	0	0	0	0
0,5251799	0	0	0	0	0
0,4842767	0	0	0	0	0
0,4482759	0	0	1	1	1
0,4759036	0	0	0	0	0
0,4567901	0	0	0	0	0
0,4730539	0	0	0	1	1
0,4670659	1	1	0	0	1
0,4046243	0	0	0	1	1
0,4393064	0	0	1	1	1
0,4404762	0	0	0	1	1
0,4216216	0	0	0	1	1
0,4488636	0	0	0	0	0
0,3941176	0	0	0	1	1
0,4342581	0	0	0	0	0
0,4277457	1	0	0	1	1
0,4146341	1	0	0	1	1
0,4712644	0	0	0	1	1
0,4597701	0	0	1	0	1
0,4354839	0	0	1	1	1
0,4745763	0	0	0	1	1
0,4913295	0	0	0	1	1
0,4775281	0	0	0	0	0
0,4886364	0	0	0	1	1
0,4651163	0	0	0	1	1
0,4829545	0	0	0	1	1
0,4432432	1	0	0	1	1
0,4712644	0	0	0	1	1
0,4516129	0	0	0	1	1

0,4419890	0	0	0	0	0
0,4636872	0	0	0	0	0
0,4819277	0	0	0	0	0
0,4910180	0	0	0	1	1
0,4597701	0	0	0	1	1
0,4624277	0	0	0	0	0
0,4500000	0	0	0	1	1
0,4655172	0	0	1	0	1
0,4825581	0	0	1	1	1
0,4800000	1	1	0	0	1
0,4748603	0	0	0	0	0
0,4733728	0	0	0	1	1
0,4719101	0	0	1	1	1
0,4764706	0	0	1	1	1
0,4645892	0	0	1	1	1
0,4722222	0	0	1	1	1
0,4827586	0	0	0	1	1
0,5000000	0	0	1	1	1
0,4806630	0	0	0	0	0
0,4770115	0	0	1	0	1
0,5015480	0	0	0	0	0
0,4391534	0	0	0	0	0
0,4438503	1	0	0	0	1
0,4883721	1	1	1	1	1
0,4943182	0	0	0	1	1
0,4565217	0	0	1	0	1
0,4754098	0	0	0	1	1
0,4644809	0	0	0	0	0
0,4705882	0	0	0	0	0
0,4659091	0	0	1	1	1
0,4827586	0	0	0	1	1
0,4611111	0	0	0	0	0
0,4597701	0	0	0	1	1
0,4525140	0	0	1	0	1
0,4790419	0	0	0	1	1
0,4925816	0	0	0	0	0
0,4712644	0	0	0	0	0
0,4802260	1	0	0	1	1
0,5212121	0	0	0	1	1
0,4678363	0	0	0	1	1
0,4702550	0	0	0	0	0
0,5000000	0	0	0	0	0
0,4255319	0	0	0	1	1
0,4494382	0	0	0	1	1
0,4719101	0	0	0	0	0
0,4651163	0	0	0	0	0
0,4632153	0	0	0	1	1
0,4790419	0	0	1	1	1
0,4545455	0	0	0	0	0
0,4825581	0	0	1	1	1

0,4857143	0	0	0	1	1
0,4882353	0	0	0	1	1
0,4857143	0	0	0	1	1
0,4678363	0	0	1	1	1
0,4761905	0	0	1	1	1
0,4475138	0	0	1	0	1
0,4659091	0	0	0	1	1
0,4772727	0	0	1	1	1
0,5125000	0	0	1	1	1
0,4855491	0	0	0	0	0
0,5153374	1	0	0	1	1
0,4636872	1	0	0	1	1
0,4672365	0	0	0	1	1
0,4729345	0	0	1	0	1
0,4528302	0	0	0	1	1
0,4821429	0	0	1	1	1
0,4571429	0	0	1	1	1
0,4780220	1	1	0	1	1
0,4886364	0	0	0	1	1
0,4659091	1	1	0	1	1
0,4777778	0	0	0	0	0
0,4942529	0	0	0	1	1
0,4942529	0	0	0	1	1
0,5000000	0	0	0	0	0
0,4640884	1	0	0	1	1
0,4725275	0	0	0	0	0
0,5087719	0	0	0	0	0
0,5029586	0	0	0	1	1
0,4786325	1	1	1	1	1
0,4795322	1	1	0	1	1
0,4885057	0	0	1	1	1
0,4971429	0	0	0	1	1
0,4924012	0	0	0	1	1
0,4940476	0	0	1	0	1
0,4512535	0	0	0	0	0
0,4486486	1	1	0	1	1
0,5030675	0	0	0	1	1
0,5029586	0	0	0	0	0
0,4883721	0	0	0	1	1
0,5092025	0	0	0	0	0
0,4615385	0	0	1	1	1
0,4739884	0	0	0	0	0
0,4662921	0	0	1	1	1
0,4606742	0	0	0	0	0
0,4590164	0	0	0	1	1
0,4722222	0	0	0	0	0
0,4941176	0	0	0	0	0
0,4686649	0	0	0	1	1
0,4833333	0	0	0	1	1
0,4651163	0	0	0	1	1

0,4726225	0	0	0	1	1
0,4829545	0	0	0	1	1
0,5030303	0	0	0	1	1
0,4913295	0	0	1	1	1
0,4781341	0	0	0	0	0
0,4713805	1	0	0	0	1
0,5153374	0	0	0	1	1
0,5000000	1	1	0	0	1
0,4715909	0	0	0	1	1
0,4795322	0	0	0	0	0
0,4597701	0	0	0	1	1
0,4852071	0	0	0	1	1
0,4858757	0	0	0	0	0
0,4519774	0	0	0	1	1
0,4651163	1	1	1	1	1
0,4754098	0	0	1	0	1
0,4606742	0	0	0	0	0
0,4795322	0	0	1	0	1
0,4912281	0	0	1	1	1
0,4689266	0	0	1	1	1
0,4627660	0	0	0	0	0
0,4910180	0	0	0	1	1
0,4696133	0	0	0	1	1
0,4545455	0	0	1	1	1
0,5058824	0	0	1	1	1
0,5000000	0	0	0	1	1
0,4624277	0	0	0	0	0
0,4956772	0	0	0	1	1
0,4505495	0	0	0	1	1
0,5180723	0	0	1	1	1
0,5088757	0	0	1	1	1
0,4659091	0	0	0	1	1
0,4910180	0	0	0	0	0
0,4913295	0	0	0	1	1
0,4526316	0	0	0	1	1
0,4651163	0	0	0	1	1
0,5059524	1	0	0	1	1
0,4699454	0	0	0	0	0
0,5341615	0	0	1	1	1
0,4819277	0	0	0	1	1
0,4941860	0	0	0	1	1
0,4624277	0	0	1	1	1
0,4797688	0	0	0	0	0
0,4910180	0	0	0	1	1
0,4772727	1	1	0	0	1
0,5185185	0	0	1	0	1
0,4860335	1	0	1	1	1
0,5121951	0	0	0	0	0
0,5089820	0	0	0	0	0
0,5370370	1	1	1	1	1

0,5029940	0	0	0	1	1
0,4797688	0	0	0	1	1
0,4648649	0	0	0	0	0
0,4772727	0	0	0	1	1
0,4494382	0	0	0	1	1
0,4823529	0	0	1	1	1
0,4912281	0	0	0	0	0
0,4901408	0	0	0	0	0
0,4745763	0	0	0	1	1
0,4827586	0	0	1	1	1
0,5075529	0	0	0	0	0
0,4886364	1	0	1	1	1
0,4767442	1	1	0	1	1
0,4855491	0	0	0	0	0
0,5060241	0	0	0	1	1
0,5029586	0	0	0	1	1
0,4770115	1	0	0	1	1
0,4955224	0	0	0	1	1
0,4928367	0	0	0	1	1
0,4712329	0	0	0	0	0
0,5030303	0	0	0	1	1
0,4815864	0	0	0	0	0
0,4795322	0	0	1	0	1
0,4880952	0	0	0	1	1
0,4754098	1	1	1	1	1
0,4970414	0	0	0	1	1
0,5384615	0	0	0	1	1
0,4807122	0	0	1	1	1
0,4955224	0	0	1	0	1
0,5072886	0	0	0	0	0
0,5000000	0	0	0	1	1
0,5212121	0	0	1	1	1
0,5120482	0	0	0	1	1
0,4640884	0	0	0	1	1
0,4751381	0	0	0	1	1
0,4860335	1	0	1	1	1
0,4831461	0	0	1	1	1
0,4462810	0	0	0	1	1
0,4793388	0	0	0	1	1
0,4910180	0	0	1	0	1
0,5212121	0	0	1	0	1
0,4829545	0	0	0	1	1
0,5178571	0	0	1	1	1
0,5000000	0	0	0	1	1
0,4956772	0	0	1	1	1
0,4970760	1	1	0	1	1
0,4970760	0	0	0	0	0
0,4530387	0	0	1	1	1
0,4912281	0	0	0	1	1
0,5149701	0	0	0	1	1

0,4857143	1	0	0	0	1
0,5119048	1	0	1	1	1
0,5304878	0	0	0	1	1
0,4912281	1	1	1	1	1
0,5058824	0	0	0	1	1
0,4709302	0	0	0	0	0
0,4909091	0	0	0	1	1
0,5000000	0	0	1	1	1
0,5014577	0	0	0	0	0
0,4797688	0	0	0	1	1
0,4550265	0	0	1	1	1
0,4970414	0	0	0	1	1
0,5119048	0	0	1	1	1
0,5000000	0	0	1	1	1
0,4911243	0	0	0	1	1
0,4941860	0	0	1	1	1
0,4786325	0	0	1	1	1
0,5058824	0	0	0	1	1
0,5370370	1	1	0	1	1
0,4857143	0	0	1	0	1
0,4819277	1	0	1	1	1
0,5454545	0	0	0	1	1
0,4761905	1	0	1	1	1
0,4676056	1	0	1	0	1
0,5058140	0	0	0	0	0
0,4880952	0	0	1	1	1
0,4555556	0	0	0	0	0
0,5014749	1	0	0	0	1
0,5263158	0	0	0	1	1
0,5304878	0	0	1	1	1
0,5157233	0	0	1	1	1
0,4581006	1	0	0	1	1
0,5000000	0	0	0	1	1
0,5163205	1	0	0	1	1
0,4585635	0	0	0	1	1
0,5151515	0	0	0	0	0
0,4924925	0	0	1	1	1
0,4821429	0	0	0	1	1
0,4941176	0	0	0	0	0
0,5120482	0	0	0	1	1
0,4715909	0	0	0	1	1
0,5030303	0	0	0	1	1
0,4912281	1	0	0	1	1
0,5149701	1	1	0	1	1
0,4914286	1	1	0	1	1
0,5029240	1	0	0	1	1
0,4901408	1	1	0	1	1
0,5029240	0	0	0	1	1
0,4857143	0	0	0	0	0
0,5087719	0	0	0	1	1

0,5014409	0	0	1	1	1
0,5000000	0	0	0	1	1
0,5209581	0	0	1	1	1
0,4623656	0	0	1	1	1
0,5058140	0	0	0	0	0
0,5000000	0	0	0	1	1
0,4806630	0	0	0	1	1
0,4887640	0	0	0	1	1
0,4914286	0	0	1	1	1
0,4733728	0	0	0	0	0
0,4829545	1	0	0	1	1
0,4882353	0	0	0	1	1
0,5178571	0	0	1	0	1
0,5192308	0	0	0	0	0
0,5000000	0	0	0	0	0
0,4985507	0	0	0	1	1
0,5060976	0	0	0	1	1
0,5120482	1	1	0	1	1
0,5060976	0	0	0	1	1
0,5058140	0	0	0	0	0
0,4636872	1	0	1	0	1
0,5058140	1	1	1	0	1
0,5180723	0	0	0	1	1
0,4985673	0	0	1	1	1
0,4887640	1	0	1	0	1
0,5149701	0	0	0	1	1
0,5028902	1	1	0	1	1
0,5000000	1	0	0	1	1
0,5087719	0	0	0	1	1
0,5246914	0	0	1	1	1
0,4748603	0	0	0	0	0
0,4911243	1	1	0	1	1
0,5120482	0	0	0	0	0
0,4971429	1	0	0	0	1
0,4909091	1	0	0	1	1
0,5121951	0	0	0	1	1
0,5045593	1	0	1	1	1
0,4971098	0	0	1	0	1
0,5087719	0	0	0	0	0
0,4957265	0	0	1	1	1
0,5014577	1	1	0	1	1
0,5147929	0	0	0	1	1
0,4939759	0	0	0	0	0
0,4885057	1	1	0	0	1
0,4705882	1	0	0	1	1
0,5093168	0	0	0	0	0
0,4882353	1	1	0	1	1
0,4692737	0	0	0	0	0
0,4640884	0	0	0	1	1
0,4971098	0	0	1	0	1

0,4941860	0	0	0	1	1
0,4640884	0	0	0	1	1
0,5120482	0	0	0	0	0
0,4942529	0	0	1	1	1
0,5000000	0	0	1	1	1
0,5117647	1	1	0	1	1
0,5149701	0	0	0	1	1
0,5000000	0	0	1	1	1
0,5240964	0	0	0	0	0
0,4610951	0	0	0	1	1
0,5209581	0	0	0	0	0
0,4742857	0	0	0	0	0
0,4858757	0	0	1	1	1
0,5000000	0	0	0	0	0
0,5043478	0	0	0	1	1
0,5240964	0	0	0	1	1
0,5090909	0	0	1	1	1
0,5029240	0	0	0	1	1
0,5194030	0	0	0	1	1
0,4565217	0	0	0	1	1
0,4873950	0	0	0	1	1
0,5119048	0	0	0	1	1
0,4910180	1	0	0	1	1
0,5028902	0	0	0	1	1
0,5299685	0	0	0	0	0
0,4955752	0	0	1	1	1
0,4942529	1	1	0	1	1
0,4929178	0	0	1	1	1
0,5132743	0	0	0	0	0
0,5151515	0	0	0	1	1
0,5212121	0	0	0	0	0
0,5276074	0	0	0	0	0
0,4802260	0	0	0	0	0
0,5058140	1	0	0	1	1
0,5000000	0	0	0	1	1
0,4941176	0	0	0	1	1
0,5173502	0	0	1	1	1
0,4777778	0	0	0	1	1
0,5443038	0	0	1	1	1
0,5178571	0	0	1	1	1
0,4887640	0	0	0	1	1
0,5058140	0	0	1	1	1
0,4913295	1	0	0	0	1
0,5103858	1	1	1	1	1
0,5212121	0	0	0	1	1
0,4911243	0	0	0	1	1
0,4971098	0	0	0	1	1
0,5092025	0	0	0	1	1
0,5443038	0	0	0	0	0
0,5119048	1	0	0	1	1

0,4823529	0	0	0	1	1
0,5058140	0	0	0	1	1
0,5073746	1	1	0	1	1
0,4829545	0	0	1	1	1
0,5089820	0	0	0	0	0
0,4971429	1	1	0	0	1
0,5214724	0	0	1	1	1
0,5276074	1	0	1	0	1
0,5375000	0	0	0	1	1
0,4623656	0	0	0	1	1
0,4910180	1	1	0	1	1
0,4858757	0	0	0	1	1
0,5194805	0	0	0	1	1
0,5149701	0	0	0	1	1
0,4886364	0	0	1	1	1
0,5325077	0	0	1	1	1
0,5087719	0	0	0	1	1
0,4970760	0	0	0	1	1
0,5364238	0	0	1	1	1
0,4823529	1	0	0	1	1
0,5243902	1	0	0	0	1
0,4941176	1	0	1	1	1
0,5043478	0	0	0	1	1
0,5029240	1	0	0	0	1
0,5433526	0	0	0	1	1
0,5000000	0	0	0	1	1
0,5197740	0	0	0	1	1
0,4888889	0	0	1	1	1
0,5056180	0	0	0	0	0
0,5172414	1	0	0	1	1
0,5294118	0	0	0	1	1
0,5325444	0	0	0	0	0
0,5257143	0	0	1	0	1
0,4888889	0	0	0	1	1
0,5146199	0	0	0	0	0
0,4838710	0	0	0	0	0
0,5263158	1	1	1	1	1
0,5141243	0	0	0	1	1
0,5444444	0	0	0	1	1
0,5071225	1	1	0	1	1
0,5000000	0	0	0	1	1
0,4861878	1	0	1	0	1
0,4756757	0	0	1	1	1
0,5055556	0	0	0	1	1
0,5112360	1	0	0	0	1
0,5139665	0	0	0	1	1
0,5056818	0	0	0	1	1
0,4812834	1	0	0	0	1
0,5028571	0	0	0	1	1
0,5000000	0	0	1	0	1

0,5432099	0	0	0	1	1
0,5359116	0	0	0	1	1
0,5207101	0	0	0	0	0
0,5217391	0	0	0	1	1
0,4787234	0	0	0	1	1
0,5112360	0	0	0	1	1
0,5294118	1	0	0	0	1
0,5204678	0	0	1	1	1
0,5644172	0	0	0	1	1
0,5146199	0	0	0	1	1
0,5112360	0	0	1	0	1
0,5116279	0	0	0	0	0
0,5438596	0	0	1	0	1
0,4971751	0	0	0	1	1
0,5000000	0	0	0	1	1
0,4972376	0	0	0	1	1
0,5195531	0	0	0	0	0
0,5195531	0	0	0	0	0
0,4986150	1	0	1	1	1
0,5582822	1	1	0	1	1
0,5000000	0	0	0	1	1
0,5114943	0	0	0	0	0
0,5161290	0	0	0	0	0
0,5222222	1	1	0	1	1
0,4875346	0	0	0	0	0
0,4835165	0	0	0	1	1
0,5086705	0	0	0	1	1
0,4986450	0	0	1	1	1
0,5142857	1	0	0	1	1
0,5027933	0	0	1	1	1
0,5449438	0	0	1	0	1
0,5207101	0	0	1	1	1
0,5000000	0	0	0	1	1
0,4890110	0	0	0	0	0
0,5172414	1	1	0	1	1
0,5254237	0	0	0	1	1
0,5307263	0	0	0	0	0
0,5731707	1	0	1	1	1
0,5142857	0	0	1	1	1
0,5082873	0	0	0	1	1
0,5028249	1	0	0	1	1
0,5423729	0	0	1	1	1
0,5083799	0	0	0	0	0
0,5000000	0	0	0	0	0
0,5465116	0	0	1	1	1
0,4684211	0	0	1	1	1
0,5082873	0	0	0	0	0
0,5112360	1	1	1	1	1
0,5028571	0	0	0	0	0
0,5116279	0	0	0	1	1

0,5361446	0	0	0	1	1
0,5132275	0	0	1	0	1
0,5100287	0	0	0	1	1
0,5321637	0	0	0	0	0
0,5397727	1	0	0	1	1
0,5248619	1	0	1	0	1
0,5625000	0	0	0	0	0
0,5085714	0	0	1	0	1
0,5325444	1	0	1	1	1
0,5476190	0	0	1	0	1
0,5575758	0	0	1	1	1
0,5111111	1	0	0	0	1
0,5000000	0	0	0	1	1
0,5215054	1	0	0	1	1
0,4851752	0	0	0	0	0
0,4943820	0	0	1	1	1
0,5071225	0	0	0	1	1
0,5056818	0	0	0	0	0
0,5170455	0	0	0	1	1
0,5146199	0	0	0	0	0
0,5141243	0	0	0	0	0
0,5219780	1	0	0	1	1
0,4972067	1	1	0	1	1
0,5000000	0	0	0	0	0
0,5172414	0	0	1	1	1
0,5176471	1	1	0	1	1
0,5432099	0	0	0	1	1
0,5229885	0	0	0	1	1
0,5112360	0	0	0	1	1
0,5257143	0	0	0	1	1
0,5238095	1	0	1	1	1
0,5317919	0	0	0	0	0
0,5732484	1	0	1	1	1
0,5337243	1	0	0	1	1
0,5248619	1	0	0	1	1
0,5055556	1	0	1	0	1
0,5200000	0	0	0	1	1
0,5028571	0	0	1	0	1
0,5232558	0	0	0	0	0
0,5257143	0	0	0	0	0
0,5107527	1	0	0	1	1
0,5347594	0	0	0	1	1
0,5679012	0	0	0	1	1
0,5681159	0	0	1	1	1
0,5454545	1	1	0	1	1
0,5027933	0	0	1	1	1
0,6079545	0	0	0	1	1
0,5197740	0	0	1	1	1
0,5166667	1	1	0	0	1
0,5562130	1	1	0	1	1

0,5731707	0	0	1	0	1
0,4971751	0	0	0	0	0
0,4917127	0	0	0	0	0
0,5329341	1	0	0	0	1
0,5099150	0	0	0	1	1
0,4918033	1	0	0	1	1
0,5529412	0	0	1	0	1
0,5284091	0	0	1	1	1
0,5397727	0	0	1	0	1
0,4835165	0	0	0	1	1
0,5131195	0	0	0	1	1
0,4835165	0	0	0	1	1
0,5000000	0	0	0	1	1
0,5340909	0	0	0	1	1
0,5113636	0	0	0	1	1
0,5333333	0	0	1	1	1
0,5254237	0	0	0	1	1
0,5000000	0	0	0	1	1
0,5122235	0	0	0	1	1
0,5527950	0	0	1	1	1
0,5361446	0	0	0	1	1
0,5384615	0	0	1	1	1
0,4734043	0	0	0	1	1
0,5260116	0	0	0	1	1
0,5114943	0	0	1	1	1
0,5432937	0	0	0	0	0
0,5614035	0	0	0	1	1
0,5164835	0	0	1	1	1
0,5371429	0	0	0	1	1
0,5294118	0	0	0	1	1
0,4943820	0	0	0	0	0
0,4930748	0	0	0	1	1
0,5266272	1	0	0	0	1
0,5069638	0	0	0	1	1
0,5227273	1	0	0	0	1
0,5085714	0	0	0	1	1
0,5079365	1	1	0	1	1
0,5227273	0	0	0	1	1
0,5348189	0	0	0	1	1
0,5535714	0	0	0	1	1
0,5307263	0	0	1	0	1
0,5402299	0	0	0	1	1
0,5444444	1	0	0	0	1
0,5529412	0	0	1	0	1
0,5084746	0	0	0	1	1
0,5227273	0	0	0	1	1
0,5266272	0	0	0	1	1
0,5170455	0	0	1	1	1
0,4835165	0	0	0	1	1
0,5106383	1	0	0	1	1

0,5529412	1	1	0	0	1
0,6067416	0	0	1	1	1
0,5146199	0	0	1	1	1
0,5170455	0	0	0	0	0
0,5083799	0	0	1	1	1
0,5321637	1	0	0	0	1
0,5303867	0	0	0	1	1
0,4945055	0	0	0	1	1
0,5195531	0	0	0	1	1
0,5251397	1	0	0	1	1
0,5470588	1	1	0	1	1
0,5168539	0	0	0	1	1
0,5195531	0	0	0	1	1
0,5508982	1	0	0	0	1
0,5428571	0	0	0	1	1
0,5329341	1	0	1	1	1
0,5107527	1	0	0	1	1
0,5647059	1	1	0	0	1
0,5812500	1	0	0	1	1
0,5433526	0	0	1	0	1
0,4888889	0	0	0	1	1
0,5084746	0	0	0	1	1
0,5116279	0	0	0	1	1
0,5053763	0	0	0	1	1
0,5055556	1	0	0	0	1
0,5443425	0	0	0	1	1
0,5600000	0	0	0	1	1
0,5254237	0	0	0	1	1
0,5139665	0	0	0	0	0
0,5129534	0	0	1	1	1
0,5530726	0	0	1	1	1
0,5301205	0	0	1	1	1
0,5193370	0	0	0	1	1
0,5284091	0	0	1	1	1
0,5348837	1	0	0	1	1
0,5371429	1	0	0	0	1
0,5485714	1	1	1	1	1
0,5174419	1	0	0	1	1
0,5057471	1	0	0	0	1
0,5333333	0	0	1	0	1
0,5057471	0	0	0	1	1
0,4917127	0	0	0	1	1
0,5055556	1	0	0	1	1
0,5321637	0	0	0	1	1
0,5284091	0	0	0	1	1
0,5292479	0	0	1	1	1
0,5314286	1	1	1	1	1
0,5575758	1	0	1	1	1
0,5535714	0	0	0	1	1
0,5421687	1	0	0	1	1

0,5141243	1	0	0	1	1
0,5290698	0	0	0	1	1
0,5278592	0	0	0	1	1
0,5204678	0	0	1	1	1
0,5367232	0	0	0	0	0
0,5191740	0	0	1	0	1
0,5176471	1	1	0	0	1
0,5251397	1	1	1	1	1
0,5352941	1	1	0	1	1
0,5449275	0	0	0	1	1
0,5202312	1	1	1	1	1
0,5329670	0	0	1	1	1
0,5317919	0	0	1	1	1
0,5511364	1	0	1	1	1
0,5773810	0	0	0	1	1
0,5056180	0	0	0	1	1
0,5465839	1	1	1	1	1
0,5454545	0	0	0	1	1
0,4972973	0	0	0	1	1
0,5113636	1	0	0	0	1
0,5313433	0	0	1	0	1
0,5114943	0	0	0	0	0
0,5207101	0	0	0	1	1
0,5579937	0	0	0	1	1
0,5411765	0	0	0	0	0
0,5251397	0	0	1	1	1
0,5055556	0	0	0	1	1
0,5287356	0	0	1	1	1
0,5460123	0	0	1	1	1
0,5555556	0	0	0	1	1
0,5000000	0	0	0	1	1
0,5101449	1	1	0	1	1
0,5254237	0	0	0	0	0
0,5028571	0	0	0	1	1
0,5057471	0	0	1	0	1
0,5309735	0	0	1	1	1
0,5242165	0	0	0	1	1
0,5191257	1	1	1	1	1
0,5042017	1	0	0	1	1
0,5138122	0	0	0	1	1
0,5402299	0	0	1	1	1
0,5421687	0	0	0	1	1
0,5176471	0	0	0	1	1
0,5290698	0	0	1	1	1
0,5317919	1	0	0	0	1
0,5257143	0	0	1	1	1
0,5595238	0	0	0	1	1
0,5421687	0	0	0	1	1
0,5135135	0	0	0	1	1
0,5325779	1	1	0	1	1

0,5476190	1	1	1	1	1
0,5581395	1	0	0	1	1
0,5460123	0	0	0	0	0
0,5083799	0	0	1	0	1
0,5235294	0	0	0	1	1
0,5284091	0	0	0	1	1
0,5144509	0	0	1	1	1
0,5340909	0	0	1	1	1
0,5600000	0	0	0	0	0
0,6176471	1	0	0	0	1
0,5365854	1	1	1	1	1
0,5113636	0	0	1	1	1
0,5168539	0	0	1	0	1
0,5864198	1	1	0	0	1
0,5027933	0	0	1	1	1
0,5266272	0	0	0	1	1
0,5229885	1	0	0	1	1
0,5070423	0	0	0	0	0
0,5287356	0	0	0	1	1
0,5344828	0	0	0	1	1
0,5072046	1	0	1	1	1
0,5189504	0	0	0	1	1
0,5287356	0	0	1	1	1
0,5405405	1	0	1	1	1
0,5170455	0	0	1	1	1
0,5614035	0	0	1	0	1
0,5589041	0	0	1	1	1
0,4782609	1	1	1	1	1
0,5297619	1	0	0	1	1
0,5132275	0	0	1	1	1
0,5411765	0	0	0	1	1
0,5028571	1	0	0	1	1
0,5515152	1	0	1	1	1
0,5402299	1	0	0	0	1
0,5314286	0	0	1	1	1
0,5654762	0	0	0	0	0
0,5657143	1	1	1	1	1
0,5071225	1	0	0	1	1
0,5227273	0	0	0	0	0
0,5595238	0	0	0	1	1
0,5521472	0	0	1	1	1
0,5085714	0	0	0	0	0
0,5112360	1	1	0	1	1
0,5355191	0	0	1	1	1
0,5590778	1	0	1	0	1
0,5593220	1	0	1	1	1
0,5545723	0	0	1	0	1
0,5574713	0	0	0	1	1
0,5762712	0	0	1	0	1
0,5753425	1	0	0	1	1

0,5146199	0	0	1	0	1
0,5181058	0	0	0	0	0
0,5027322	0	0	0	1	1
0,5000000	1	0	0	1	1
0,5297619	0	0	0	0	0
0,5542169	0	0	0	1	1
0,5266272	0	0	0	1	1
0,5545723	1	0	0	0	1
0,5227273	1	0	0	0	1
0,5523256	0	0	0	1	1
0,5664740	0	0	1	1	1
0,4944444	0	0	0	0	0
0,5585586	0	0	1	1	1
0,5740741	0	0	1	1	1
0,5389222	0	0	0	0	0
0,5939394	1	1	0	1	1
0,5317919	0	0	0	0	0
0,5176471	0	0	0	0	0
0,5357143	1	1	1	1	1
0,5055556	0	0	0	1	1
0,5444444	0	0	1	1	1
0,5202312	0	0	1	1	1
0,5939394	1	0	0	0	1
0,5000000	1	1	0	1	1
0,5263158	0	0	0	0	0
0,5287356	0	0	0	1	1
0,5027933	1	0	1	1	1
0,5067385	0	0	0	1	1
0,5664740	0	0	0	0	0
0,4972973	1	0	0	0	1
0,5321637	1	1	1	1	1
0,6477273	1	1	1	1	1
0,5428571	0	0	0	1	1
0,6034483	1	1	0	1	1
0,5903614	0	0	1	1	1
0,5777778	1	1	0	1	1
0,5586592	1	0	0	0	1
0,5352941	0	0	0	1	1
0,5914634	1	1	1	0	1
0,5454545	1	1	0	1	1
0,5593220	0	0	1	1	1
0,5561798	0	0	0	1	1
0,6184971	0	0	1	1	1
0,5219780	0	0	0	1	1
0,5352941	1	0	0	1	1
0,5555556	0	0	1	1	1
0,6000000	0	0	0	1	1
0,5529412	0	0	0	1	1
0,5617978	1	1	1	1	1
0,5337079	0	0	1	1	1

0,5287356	0	0	0	1	1
0,5919540	0	0	1	0	1
0,5465116	0	0	0	1	1
0,5542857	0	0	1	1	1
0,5352941	0	0	0	1	1
0,5852273	1	1	0	1	1
0,5263158	0	0	0	1	1
0,5418994	0	0	1	1	1
0,5680473	0	0	1	1	1
0,5217391	0	0	0	1	1
0,5722543	0	0	1	1	1
0,6179775	1	1	1	1	1
0,5433526	1	1	0	1	1
0,5696970	1	1	1	1	1
0,5764706	0	0	0	1	1
0,5238095	0	0	1	1	1
0,4971751	1	0	0	1	1
0,5257143	0	0	0	0	0
0,5562500	0	0	0	1	1
0,5481928	1	1	1	0	1
0,5621302	0	0	1	1	1
0,6117647	0	0	0	1	1
0,6036585	0	0	1	1	1
0,5271739	0	0	0	1	1
0,5714286	0	0	0	1	1
0,5333333	0	0	0	0	0
0,6101695	1	1	1	1	1
0,5202312	1	0	0	1	1
0,5159420	1	0	0	1	1
0,5524862	0	0	0	1	1
0,5317919	0	0	0	1	1
0,5135135	0	0	0	1	1
0,6176471	1	0	0	0	1
0,5112360	1	0	0	0	1
0,5308311	0	0	0	1	1
0,5000000	1	0	0	0	1
0,5540166	0	0	1	1	1
0,5361446	0	0	0	1	1
0,5344828	0	0	0	1	1
0,4929972	0	0	0	1	1
0,5329670	1	0	0	1	1
0,5411765	1	0	1	1	1
0,5266106	1	1	1	0	1
0,5352941	1	0	0	1	1
0,5371429	0	0	1	1	1
0,5465116	0	0	0	1	1
0,4736842	0	0	0	0	0
0,5359116	1	0	1	1	1
0,5737705	0	0	1	1	1
0,5748503	0	0	0	1	1

0,4814815	0	0	1	1	1
0,5212465	0	0	1	1	1
0,5000000	1	0	1	1	1
0,5000000	0	0	0	1	1
0,5197740	0	0	0	1	1
0,5135135	0	0	1	1	1
0,4916201	0	0	0	0	0
0,5493827	0	0	1	1	1
0,4782609	0	0	0	1	1
0,5380117	0	0	0	0	0
0,5141243	1	0	0	0	1
0,5535714	0	0	0	1	1
0,5340909	0	0	0	1	1
0,5056818	1	0	0	1	1
0,5333333	0	0	0	1	1
0,4972973	0	0	1	1	1
0,5168539	0	0	1	0	1
0,5487805	0	0	0	0	0
0,5344828	0	0	0	0	0
0,5549133	0	0	1	1	1
0,5953757	0	0	1	1	1
0,5081967	0	0	0	1	1
0,5529412	1	0	1	1	1
0,5485714	1	0	0	1	1
0,5433526	1	1	1	1	1
0,5433526	0	0	0	0	0
0,5459770	0	0	0	0	0
0,6265060	1	1	0	1	1
0,4971751	0	0	0	0	0
0,5170455	0	0	0	1	1
0,5056180	0	0	0	1	1
0,5487805	0	0	0	0	0
0,5558912	0	0	0	0	0
0,5571848	0	0	1	0	1
0,5706522	1	1	0	1	1
0,5202312	0	0	0	0	0
0,5057471	0	0	0	1	1
0,5085714	0	0	0	0	0
0,5411765	0	0	0	1	1
0,5294118	1	0	1	1	1
0,5193370	0	0	1	1	1
0,5491329	1	1	1	1	1
0,4971751	0	0	0	1	1
0,5202312	0	0	1	1	1
0,5263158	1	0	0	1	1
0,5200000	0	0	1	1	1
0,5438596	0	0	1	0	1
0,5265957	0	0	0	1	1
0,5340909	1	1	1	1	1
0,5227273	0	0	1	1	1

0,5263158	0	0	1	1	1
0,5215054	0	0	1	0	1
0,5222222	0	0	1	1	1
0,5325444	1	0	0	0	1
0,5235294	0	0	0	0	0
0,5706215	0	0	0	0	0
0,5617978	0	0	0	0	0
0,5235294	1	1	0	1	1
0,5290698	0	0	0	1	1
0,5427729	0	0	1	1	1
0,5082873	0	0	0	1	1
0,5224719	0	0	0	0	0
0,5402299	0	0	1	1	1
0,5706052	0	0	0	1	1
0,5575758	0	0	0	1	1
0,5189189	0	0	0	1	1
0,5868263	0	0	0	1	1
0,5555556	0	0	0	1	1
0,4972973	0	0	1	0	1
0,5028090	0	0	0	1	1
0,5028249	0	0	0	1	1
0,5600000	1	0	0	1	1
0,5251397	1	0	1	1	1
0,5771429	0	0	0	1	1
0,5762712	1	0	1	1	1
0,5760870	0	0	0	1	1
0,5942857	0	0	1	1	1
0,5172414	1	0	0	1	1
0,5757576	1	0	0	0	1
0,5224719	1	1	0	1	1
0,5217391	1	0	0	0	1
0,5502959	1	1	1	1	1
0,5485714	0	0	0	1	1
0,5454545	0	0	1	0	1
0,5689655	1	0	0	1	1
0,4838710	0	0	1	0	1
0,5411765	0	0	0	0	0
0,5202312	1	1	0	1	1
0,5251397	0	0	0	1	1
0,5481050	0	0	0	1	1
0,5523256	0	0	0	1	1
0,5769231	0	0	1	1	1
0,5260116	1	0	1	1	1
0,5139665	0	0	0	1	1
0,4888889	0	0	0	1	1
0,5191740	1	0	0	1	1
0,5235294	0	0	0	1	1
0,5202312	0	0	0	0	0
0,5495751	0	0	0	1	1
0,5352941	0	0	0	1	1

0,5757576	1	0	0	0	1
0,5595238	1	0	1	0	1
0,5480226	1	1	0	1	1
0,5351351	1	1	1	1	1
0,5562130	0	0	1	0	1
0,5365854	0	0	1	0	1
0,5857988	1	0	0	1	1
0,6071429	0	0	0	1	1
0,5357143	1	1	0	1	1
0,5000000	1	1	0	1	1
0,5352941	1	0	0	1	1
0,5410334	0	0	0	0	0
0,5471125	0	0	0	1	1
0,5406977	0	0	0	1	1
0,5130890	0	0	0	1	1
0,5444444	0	0	0	1	1
0,5254237	0	0	0	0	0
0,5284091	0	0	0	1	1
0,5132275	0	0	1	0	1
0,5459770	0	0	1	1	1
0,5517241	1	1	0	1	1
0,5113636	0	0	0	0	0
0,5433526	1	0	0	1	1
0,5195531	0	0	0	1	1
0,5568862	0	0	1	1	1
0,5136612	1	1	1	1	1
0,5238095	1	0	0	0	1
0,5254237	0	0	1	0	1
0,5333333	0	0	0	1	1
0,5773810	1	0	1	0	1
0,5389222	0	0	0	0	0
0,5321637	0	0	0	1	1
0,5565217	0	0	1	1	1
0,5632184	0	0	0	1	1
0,5027624	0	0	0	1	1
0,5302594	0	0	0	1	1
0,5371429	0	0	0	1	1
0,5341246	0	0	0	0	0
0,5427729	0	0	1	1	1
0,5386819	0	0	0	0	0
0,5523256	0	0	1	0	1
0,5672515	0	0	0	1	1
0,5232558	0	0	0	1	1
0,5139665	1	0	0	1	1
0,5172414	0	0	0	1	1
0,5485714	0	0	0	1	1
0,5108696	0	0	0	1	1
0,5470588	0	0	0	1	1
0,5202312	0	0	1	1	1
0,5268817	1	0	0	1	1

0,5164835	0	0	0	1	1
0,5730337	1	1	1	1	1
0,5469613	0	0	0	1	1
0,5593220	0	0	0	0	0
0,5862069	0	0	0	1	1
0,5816024	0	0	0	1	1
0,5113636	0	0	0	1	1
0,5269122	0	0	0	1	1
0,5423729	0	0	1	0	1
0,5411765	0	0	1	1	1
0,5157593	1	0	1	1	1
0,5433526	1	0	1	1	1
0,5454545	1	0	0	1	1
0,5331361	1	1	0	1	1
0,6111111	1	0	0	1	1
0,5333333	0	0	0	1	1
0,5882353	1	0	0	1	1
0,5529412	0	0	0	1	1
0,5823529	0	0	1	0	1
0,5900621	0	0	0	0	0
0,5683060	1	0	0	0	1
0,5505618	1	0	0	1	1
0,5357143	1	0	0	1	1
0,5375723	0	0	0	0	0
0,5217391	0	0	0	1	1
0,5562130	1	0	1	1	1
0,5111111	0	0	0	1	1
0,5454545	0	0	1	1	1
0,5395894	1	0	0	1	1
0,5260116	1	0	0	0	1
0,5340599	0	0	1	1	1
0,5454545	0	0	0	0	0
0,5630499	1	0	0	0	1
0,5480226	0	0	0	0	0
0,5666667	1	0	0	1	1
0,5301205	1	1	0	1	1
0,5056818	0	0	0	1	1
0,5290698	0	0	0	0	0
0,5146199	0	0	1	0	1
0,5337079	0	0	0	1	1
0,5027322	0	0	0	1	1
0,5395894	0	0	0	1	1
0,5586592	0	0	0	1	1
0,5177112	0	0	0	1	1
0,5187166	0	0	0	1	1
0,5523256	1	0	1	0	1
0,5344828	0	0	0	1	1
0,5086705	1	1	1	1	1
0,4917127	0	0	0	0	0
0,5061268	0	0	0	0	0

0,5027933	0	0	0	1	1
0,5138122	0	0	0	1	1
0,5480226	0	0	1	0	1
0,5375723	1	0	0	1	1
0,5481928	1	1	1	1	1
0,5314286	0	0	1	1	1
0,5647059	0	0	1	1	1
0,5783133	0	0	0	1	1
0,5057471	0	0	0	1	1
0,5057471	0	0	0	1	1
0,5427729	1	1	0	1	1
0,5176471	0	0	1	0	1
0,5170455	0	0	1	1	1
0,5055556	0	0	0	1	1
0,5317919	1	1	0	1	1
0,5351351	0	0	1	1	1
0,5357143	1	1	0	1	1
0,5243243	1	0	1	0	1
0,5502646	1	0	1	1	1
0,5697674	1	1	1	1	1
0,5739130	0	0	0	1	1
0,5519288	1	0	1	1	1
0,5027624	0	0	1	0	1
0,5491329	0	0	0	1	1
0,5144509	0	0	0	0	0
0,5470588	0	0	0	0	0
0,5297619	0	0	0	1	1
0,5141243	0	0	0	1	1
0,5193370	0	0	1	1	1
0,5454545	1	1	1	0	1
0,5432836	0	0	0	0	0
0,5535714	1	1	0	1	1
0,5497076	1	1	0	1	1
0,5268817	0	0	1	1	1
0,5900621	0	0	0	1	1
0,5789474	0	0	1	1	1
0,5269461	0	0	0	1	1
0,4861878	0	0	1	0	1
0,4870466	0	0	0	1	1
0,5144509	0	0	0	1	1
0,5321637	1	0	0	1	1
0,5229885	0	0	0	1	1
0,5325444	0	0	0	1	1
0,5168539	0	0	0	1	1
0,5561798	0	0	1	1	1
0,5747126	0	0	0	1	1
0,5602410	1	1	0	1	1
0,5680473	0	0	0	0	0
0,5529412	1	0	0	0	1
0,5697674	0	0	0	1	1

0,5300546	0	0	1	1	1
0,5688623	0	0	0	1	1
0,5174419	1	0	0	0	1
0,5056818	0	0	0	0	0
0,5368732	0	0	0	1	1
0,5464789	1	0	1	1	1
0,5257143	1	1	1	0	1
0,5470588	0	0	1	1	1
0,5497076	0	0	0	1	1
0,5375723	0	0	0	0	0
0,5123967	0	0	1	0	1
0,5779221	0	0	0	0	0
0,5361446	0	0	1	1	1
0,5307263	0	0	1	1	1
0,5411765	0	0	0	0	0
0,5375723	0	0	0	1	1
0,5614035	0	0	1	1	1
0,5706052	1	0	0	1	1
0,5277778	0	0	1	0	1
0,5919540	0	0	0	0	0
0,5882353	0	0	0	1	1
0,5930233	1	0	1	1	1
0,5224719	0	0	0	1	1
0,5142857	0	0	0	1	1
0,5207101	1	1	0	1	1
0,5176471	1	0	0	1	1
0,5635359	0	0	0	0	0
0,5112360	1	0	0	0	1
0,5340909	0	0	0	0	0
0,5534591	0	0	0	1	1
0,5224274	1	1	1	1	1
0,5287356	1	0	1	1	1
0,5654762	0	0	0	1	1
0,5384615	0	0	0	1	1
0,5486726	1	1	1	1	1
0,5485714	0	0	0	1	1
0,5217391	0	0	0	1	1
0,5277778	0	0	1	1	1
0,5614035	0	0	1	1	1
0,5481928	0	0	1	1	1
0,5838150	0	0	0	1	1
0,6121212	0	0	0	1	1
0,5207101	1	0	0	1	1
0,5056180	1	0	0	1	1
0,5164835	0	0	0	1	1
0,5235294	0	0	0	1	1
0,5449438	1	0	1	1	1
0,5384615	1	1	0	1	1
0,5497076	0	0	0	0	0
0,5524862	0	0	0	1	1

0,5811966	1	1	1	1	1
0,5502959	0	0	1	1	1
0,5351351	1	0	0	0	1
0,5647059	1	0	1	1	1
0,5944444	0	0	0	1	1
0,6273292	0	0	0	1	1
0,5764706	0	0	1	0	1
0,5689655	0	0	0	1	1
0,5977011	0	0	0	1	1
0,5081967	0	0	0	1	1
0,5410334	0	0	0	1	1
0,5357143	0	0	1	0	1
0,5197740	0	0	0	1	1
0,5202312	1	1	0	1	1
0,5382436	0	0	0	1	1
0,5300546	1	1	1	1	1
0,5317919	0	0	0	1	1
0,5517241	0	0	0	1	1
0,5129534	1	0	1	1	1
0,5438596	0	0	0	0	0
0,5755814	1	1	0	1	1
0,5602410	0	0	0	0	0
0,5808383	0	0	0	1	1
0,5698925	0	0	0	1	1
0,5730337	0	0	0	1	1
0,5689655	0	0	0	0	0
0,5580110	0	0	0	1	1
0,5454545	0	0	0	1	1
0,5109890	0	0	1	1	1
0,5405405	0	0	0	1	1
0,5808383	1	0	0	1	1
0,5474860	0	0	0	1	1
0,5397727	1	0	1	1	1
0,5529412	1	1	1	1	1
0,5674157	0	0	0	1	1
0,5495751	0	0	1	0	1
0,5722222	0	0	0	1	1
0,5635359	0	0	1	1	1
0,5443787	0	0	0	1	1
0,5176471	0	0	1	0	1
0,5166667	0	0	1	1	1
0,5086705	0	0	1	1	1
0,5257143	0	0	0	1	1
0,5245902	0	0	0	1	1
0,5542169	0	0	0	1	1
0,5525526	0	0	0	1	1
0,5892857	1	0	0	1	1
0,5238095	0	0	1	1	1
0,5109890	0	0	1	0	1
0,5086705	1	1	0	1	1

0,5146199	1	0	0	1	1
0,5212766	0	0	0	1	1
0,5411765	0	0	1	1	1
0,5780347	0	0	0	1	1
0,5380435	1	0	0	1	1
0,5729730	1	0	1	1	1
0,5819209	0	0	0	1	1
0,5321637	0	0	0	1	1
0,5056180	0	0	1	1	1
0,4945055	0	0	0	1	1
0,5527066	0	0	1	0	1
0,5099150	0	0	0	1	1
0,5195531	0	0	1	1	1
0,5639535	0	0	1	1	1
0,5310734	0	0	0	1	1
0,5269461	1	0	0	1	1
0,5082873	0	0	0	1	1
0,5393258	0	0	0	1	1
0,5626911	0	0	0	0	0
0,5614035	1	1	1	1	1
0,5647059	0	0	0	0	0
0,5823529	1	1	1	1	1
0,5449102	0	0	0	1	1
0,5535714	0	0	1	1	1
0,5792683	0	0	0	1	1
0,5511364	0	0	0	0	0
0,5600000	0	0	0	0	0
0,5217391	0	0	0	0	0
0,5543478	1	0	0	1	1
0,5523256	1	0	1	1	1
0,5405405	1	1	0	1	1
0,5465116	0	0	1	1	1
0,5804598	1	1	0	0	1
0,5914634	0	0	1	0	1
0,5925926	0	0	0	0	0
0,5326087	0	0	0	1	1
0,5909091	0	0	1	1	1
0,5172414	1	0	0	1	1
0,5274725	1	0	1	1	1
0,5464481	1	0	0	1	1
0,4945652	0	0	0	0	0
0,5294118	0	0	0	0	0
0,5139665	0	0	0	0	0
0,4973545	1	1	0	1	1
0,5260116	0	0	0	0	0
0,5337079	1	1	1	1	1
0,5636364	1	0	0	1	1
0,5432099	1	0	1	1	1
0,5161290	0	0	0	1	1
0,5371429	0	0	1	1	1

0,5393258	0	0	0	1	1
0,5853659	0	0	0	1	1
0,5517241	0	0	1	1	1
0,5561798	0	0	0	1	1
0,5714286	0	0	0	0	0
0,5459941	0	0	0	0	0
0,6062500	0	0	1	1	1
0,5689655	0	0	0	1	1
0,5367232	0	0	1	0	1
0,5697674	0	0	0	1	1
0,5517241	0	0	1	1	1
0,5797101	0	0	0	1	1
0,5635359	1	0	0	0	1
0,5755814	1	1	0	1	1
0,5280899	1	0	1	1	1
0,5238095	0	0	0	1	1
0,5389222	0	0	0	1	1
0,5128205	0	0	0	0	0
0,5527066	0	0	0	1	1
0,5142857	0	0	1	1	1
0,5139665	0	0	1	1	1
0,5679012	0	0	0	1	1
0,5771429	1	0	1	1	1
0,5885714	1	0	1	1	1
0,5773810	1	0	0	1	1
0,5895954	1	1	1	1	1
0,5769231	1	0	1	1	1
0,5857988	0	0	0	1	1
0,5081967	0	0	1	1	1
0,5280899	0	0	0	1	1
0,5214900	0	0	0	0	0
0,5337079	0	0	1	1	1
0,5511364	0	0	0	1	1
0,5111111	1	0	0	1	1
0,5459770	1	0	0	1	1
0,5459941	1	1	1	1	1
0,5481050	0	0	0	0	0
0,5628743	1	1	1	1	1
0,5680473	0	0	0	1	1
0,5528455	0	0	0	1	1
0,5329513	0	0	0	1	1
0,5443787	0	0	0	1	1
0,5647059	0	0	0	1	1
0,5747801	0	0	0	0	0
0,6503067	1	0	0	1	1
0,5086705	0	0	0	0	0
0,5251397	1	1	0	1	1
0,5027624	0	0	0	1	1
0,5406977	0	0	0	1	1
0,5297619	1	1	0	1	1

0,5084746	0	0	1	1	1
0,5465116	1	1	0	1	1
0,5183099	1	0	0	1	1
0,5389222	1	1	1	0	1
0,5317919	0	0	0	1	1
0,5454545	1	0	1	0	1
0,5373961	0	0	1	1	1
0,5730337	0	0	0	0	0
0,5361446	0	0	0	1	1
0,5485714	0	0	0	1	1
0,5903614	0	0	1	1	1
0,5268817	1	0	1	0	1
0,5549133	1	1	1	1	1
0,5344353	0	0	1	1	1
0,5680473	1	1	1	1	1
0,5798817	0	0	0	1	1
0,6000000	0	0	0	1	1
0,5730337	1	1	1	1	1
0,5783784	0	0	0	0	0
0,5755814	0	0	1	1	1
0,5195531	0	0	0	1	1
0,5227273	1	1	0	1	1
0,5202312	1	0	0	1	1
0,5080214	0	0	0	0	0
0,5647059	1	1	0	1	1
0,5426829	0	0	0	0	0
0,5317919	0	0	0	1	1
0,5502959	0	0	0	1	1
0,5384615	0	0	0	1	1
0,5432836	0	0	0	1	1
0,5588235	0	0	0	0	0
0,5511364	0	0	0	0	0
0,5430108	1	0	1	1	1
0,6011905	0	0	0	1	1
0,5755814	0	0	0	1	1
0,5433526	0	0	0	1	1
0,6012270	0	0	1	1	1
0,5564738	0	0	1	1	1
0,5786164	0	0	0	0	0
0,6000000	1	0	1	1	1
0,5278592	0	0	1	1	1
0,5438596	0	0	0	1	1
0,5341246	1	1	0	0	1
0,5365854	0	0	0	1	1
0,5348837	0	0	1	1	1
0,5170455	0	0	0	1	1
0,5568182	0	0	0	1	1
0,5224719	0	0	0	0	0
0,5269461	0	0	1	1	1
0,5497076	0	0	0	0	0

0,5418994	0	0	1	1	1
0,5535714	1	1	0	1	1
0,5280899	0	0	1	1	1
0,5542857	0	0	0	1	1
0,5505618	1	0	0	1	1
0,5623188	0	0	0	1	1
0,5465116	0	0	1	1	1
0,5652174	0	0	1	1	1
0,5414365	1	0	0	1	1
0,5762712	1	0	0	1	1
0,5795455	0	0	0	1	1
0,5511364	0	0	0	1	1
0,6030769	1	1	1	1	1
0,5434783	1	0	0	1	1
0,5561798	1	0	0	1	1
0,5773810	1	0	0	0	1
0,5561798	0	0	1	1	1
0,5106383	1	0	1	1	1
0,5139665	1	0	1	1	1
0,5490196	0	0	0	1	1
0,5357143	0	0	0	1	1
0,5449438	1	1	0	1	1
0,5549133	0	0	0	1	1
0,5164835	0	0	1	1	1
0,5372340	1	0	0	1	1
0,5438596	1	1	0	1	1
0,5636364	1	0	1	1	1
0,5423729	0	0	0	1	1
0,5236769	1	0	0	1	1
0,5586592	1	1	1	1	1
0,5444444	1	0	0	0	1
0,5964912	1	0	1	1	1
0,6011905	1	1	1	1	1
0,5549133	1	0	0	1	1
0,5795455	0	0	1	1	1
0,5568182	0	0	1	0	1
0,5963855	0	0	0	0	0
0,5542857	1	0	0	1	1
0,5714286	0	0	1	1	1
0,5819209	0	0	0	1	1
0,5621622	1	1	0	1	1
0,5489130	1	0	1	1	1
0,4957746	1	0	1	0	1
0,5238095	0	0	0	1	1
0,5227273	1	0	0	0	1
0,5028249	1	0	1	1	1
0,5113636	0	0	1	1	1
0,5086705	1	0	0	1	1
0,5637982	1	0	1	1	1
0,5459770	1	0	0	1	1

0,5454545	0	0	0	0	0
0,5568182	0	0	0	1	1
0,5487805	0	0	0	0	0
0,5287356	0	0	0	0	0
0,5647059	0	0	1	1	1
0,5573770	1	0	0	1	1
0,5581395	0	0	1	1	1
0,5590778	0	0	1	1	1
0,5529412	0	0	0	0	0
0,5617978	1	0	1	1	1
0,5491329	0	0	1	1	1
0,5230769	0	0	1	1	1
0,5329670	1	0	1	1	1
0,5511364	0	0	1	1	1
0,5445026	1	0	1	1	1
0,5780347	1	1	1	1	1
0,5529412	0	0	0	1	1
0,5955056	0	0	1	1	1
0,5568182	1	0	0	1	1
0,5562130	0	0	1	1	1
0,5536723	0	0	1	1	1
0,6081871	0	0	0	1	1
0,5578947	0	0	0	1	1
0,5925926	0	0	0	1	1
0,5868263	0	0	1	0	1
0,5930233	1	1	1	1	1
0,5780347	0	0	0	1	1
0,5882353	0	0	0	1	1
0,6687117	1	0	1	1	1
0,5941176	1	1	1	1	1
0,5229885	0	0	0	0	0
0,5294118	1	0	0	1	1
0,5584046	0	0	0	1	1
0,5367232	0	0	1	1	1
0,5581395	0	0	0	1	1
0,5813953	1	1	0	1	1
0,5197740	0	0	0	1	1
0,5808383	0	0	0	0	0
0,5459770	0	0	0	1	1
0,5672515	0	0	0	1	1
0,5380117	0	0	0	1	1
0,5810398	0	0	1	1	1
0,5348837	1	1	1	1	1
0,5588235	0	0	1	1	1
0,5762712	0	0	1	1	1
0,5414365	0	0	0	1	1
0,5747126	0	0	1	1	1
0,5470588	0	0	0	1	1
0,6000000	1	0	1	1	1
0,5722222	0	0	1	0	1

0,4958678	0	0	1	1	1
0,4986150	1	0	1	1	1
0,5389222	0	0	1	1	1
0,5294118	0	0	0	1	1
0,5116279	0	0	0	1	1
0,5542857	0	0	1	1	1
0,5714286	1	0	1	1	1
0,5460123	0	0	0	1	1
0,5352941	0	0	0	0	0
0,5294118	0	0	1	1	1
0,5439093	1	0	1	0	1
0,5337079	0	0	1	1	1
0,5747126	1	0	0	1	1
0,5632184	1	1	1	1	1
0,5494505	1	0	0	1	1
0,5597668	1	0	0	1	1
0,5325779	1	1	0	0	1
0,5806452	1	0	0	1	1
0,5562130	0	0	0	1	1
0,5681159	0	0	0	1	1
0,5801105	0	0	1	1	1
0,4972376	1	1	0	1	1
0,5263158	0	0	0	1	1
0,5581395	0	0	1	1	1
0,5470588	0	0	0	0	0
0,5602410	0	0	0	1	1
0,5521127	1	0	1	1	1
0,5494505	0	0	1	0	1
0,5207756	1	0	0	1	1
0,5424658	0	0	0	1	1
0,5163043	0	0	1	1	1
0,5373134	0	0	0	1	1
0,5118734	0	0	1	1	1
0,5804598	1	1	0	0	1
0,5722892	0	0	1	1	1
0,5919540	1	0	0	1	1
0,5649718	0	0	0	1	1
0,5444744	0	0	0	1	1
0,5549133	1	0	0	1	1
0,5826331	0	0	1	0	1
0,5602410	0	0	0	1	1
0,5730337	1	1	1	1	1
0,5689655	0	0	1	0	1
0,5722892	0	0	0	1	1
0,5722543	1	1	0	1	1
0,5965909	0	0	1	1	1
0,5580110	1	0	1	0	1
0,6284153	0	0	0	1	1
0,4891304	0	0	0	1	1
0,5438596	0	0	0	1	1

0,5617978	1	0	0	0	1
0,5082873	0	0	0	1	1
0,5389222	0	0	1	1	1
0,5568182	1	0	0	1	1
0,5464481	1	1	1	1	1
0,5438066	0	0	0	0	0
0,5747801	0	0	0	1	1
0,6011905	0	0	1	1	1
0,5595238	0	0	0	1	1
0,5529412	0	0	0	1	1
0,5632184	0	0	0	1	1
0,6033520	0	0	1	1	1
0,5146199	0	0	1	1	1
0,5260116	1	1	1	0	1
0,5311653	1	0	0	1	1
0,5284091	0	0	0	0	0
0,5505618	0	0	0	1	1
0,5139665	0	0	1	1	1
0,5555556	0	0	0	1	1
0,5365854	0	0	0	1	1
0,5941176	1	1	0	1	1
0,5680473	0	0	0	1	1
0,5635359	1	1	1	0	1
0,5647059	0	0	0	1	1
0,5511364	0	0	0	1	1
0,5555556	1	1	0	1	1
0,5669782	1	1	1	1	1
0,5495751	0	0	0	1	1
0,5714286	1	0	1	1	1
0,5804598	0	0	1	0	1
0,5917160	1	0	0	1	1
0,5688623	0	0	0	0	0
0,5606936	0	0	0	1	1
0,5783133	0	0	0	0	0
0,5892857	0	0	0	1	1
0,5747126	0	0	0	1	1
0,6234568	1	1	0	1	1
0,5135135	1	1	1	1	1
0,5284091	0	0	0	0	0
0,5299145	0	0	1	0	1
0,5363128	0	0	0	1	1
0,5157895	0	0	1	0	1
0,5632184	0	0	0	1	1
0,5632184	1	0	0	1	1
0,5632184	0	0	1	1	1
0,5388889	1	1	0	0	1
0,5581395	0	0	1	1	1
0,5595238	1	0	1	1	1
0,5783784	1	0	1	1	1
0,5755814	0	0	0	1	1

0,5882353	1	0	1	1	1
0,5786517	0	0	0	1	1
0,5769231	0	0	0	1	1
0,5823529	0	0	0	0	0
0,5868263	1	0	0	1	1
0,5852273	1	1	1	1	1
0,6035503	0	0	0	1	1
0,5111111	1	0	0	1	1
0,5348837	0	0	0	1	1
0,5740741	1	1	0	0	1
0,5558739	0	0	0	1	1
0,5464789	1	0	0	1	1
0,5260116	1	0	0	1	1
0,5764706	1	0	1	1	1
0,5714286	0	0	1	1	1
0,5310734	1	0	0	1	1
0,5899705	0	0	0	1	1
0,5280000	0	0	1	1	1
0,5680473	0	0	0	1	1
0,5813953	1	1	0	1	1
0,5561798	1	0	1	1	1
0,5333333	0	0	1	1	1
0,5371429	0	0	0	1	1
0,5654762	0	0	1	1	1
0,5351351	1	0	1	1	1
0,5568862	0	0	0	1	1
0,5681818	0	0	0	1	1
0,5864198	0	0	0	1	1
0,5505618	0	0	0	0	0
0,5639535	0	0	1	1	1
0,5632184	0	0	1	1	1
0,5823529	0	0	1	0	1
0,5688623	0	0	0	1	1
0,5722892	1	1	0	1	1
0,5489130	1	0	0	1	1
0,5872093	1	0	0	1	1
0,5764706	1	1	0	0	1
0,5705882	0	0	1	1	1
0,6000000	0	0	1	1	1
0,6114286	0	0	0	1	1
0,5909091	0	0	0	1	1
0,6235294	0	0	1	1	1
0,5217391	1	0	0	1	1
0,5465116	0	0	0	1	1
0,5164835	0	0	0	1	1
0,5144509	1	0	1	1	1
0,5396825	0	0	1	0	1
0,5542857	1	1	1	1	1
0,5536723	0	0	0	1	1
0,5804598	0	0	1	1	1

0,5730337	0	0	1	1	1
0,5388889	1	0	0	0	1
0,5730994	0	0	0	1	1
0,5764706	1	1	1	1	1
0,5623188	0	0	1	1	1
0,5941176	0	0	1	0	1
0,5755814	0	0	0	1	1
0,5568862	0	0	1	1	1
0,5444444	1	1	1	1	1
0,5664740	1	0	0	1	1
0,5625000	1	0	0	0	1
0,5705706	0	0	0	1	1
0,5897436	0	0	0	1	1
0,5219780	0	0	0	1	1
0,5340909	0	0	0	1	1
0,5465839	0	0	0	1	1
0,5679012	0	0	0	1	1
0,5808383	0	0	0	1	1
0,5222222	0	0	0	1	1
0,5750799	0	0	0	1	1
0,5459770	0	0	1	1	1
0,5738636	0	0	1	1	1
0,5397727	0	0	1	1	1
0,5783133	1	1	1	0	1
0,5542857	0	0	0	0	0
0,5688623	0	0	0	1	1
0,5806452	0	0	0	0	0
0,6024845	0	0	1	1	1
0,5672515	1	0	0	0	1
0,5816024	0	0	0	1	1
0,5571848	1	0	1	1	1
0,5449438	1	0	0	1	1
0,5586592	0	0	0	0	0
0,5586592	1	1	0	1	1
0,5696594	0	0	1	1	1
0,5652174	1	1	1	1	1
0,5664740	0	0	0	0	0
0,5757576	0	0	0	1	1
0,5369863	1	1	1	1	1
0,5697674	0	0	0	1	1
0,5764706	0	0	0	1	1
0,6021505	1	0	1	1	1
0,5144509	0	0	0	1	1
0,5317919	1	0	0	1	1
0,5438596	0	0	0	1	1
0,5454545	0	0	1	1	1
0,5444444	0	0	0	1	1
0,5568182	0	0	1	1	1
0,5568182	0	0	1	1	1
0,5403900	0	0	0	1	1

0,5491329	0	0	0	1	1
0,5498652	0	0	1	1	1
0,5895954	0	0	1	1	1
0,5882353	0	0	1	0	1
0,5847953	1	0	1	1	1
0,5900621	0	0	0	1	1
0,5919540	1	0	0	1	1
0,5818182	0	0	0	1	1
0,5888889	1	1	1	1	1
0,6265060	1	1	1	1	1
0,5777778	0	0	1	1	1
0,5789474	1	0	1	1	1
0,5965909	1	1	0	1	1
0,5426829	0	0	0	1	1
0,5460123	0	0	1	1	1
0,4920635	0	0	0	1	1
0,5428571	0	0	0	1	1
0,5568182	1	1	0	1	1
0,5421687	0	0	1	1	1
0,5602410	1	0	0	0	1
0,5542857	1	0	1	1	1
0,5361446	1	0	0	1	1
0,5454545	0	0	0	0	0
0,5529412	0	0	0	1	1
0,5434174	1	0	0	1	1
0,5454545	0	0	1	1	1
0,5813953	1	0	0	1	1
0,5885714	0	0	0	1	1
0,5428571	1	0	0	1	1
0,5586592	0	0	0	1	1
0,5714286	0	0	1	1	1
0,5722892	0	0	0	0	0
0,5795455	0	0	0	1	1
0,5588235	0	0	0	0	0
0,6131805	0	0	1	1	1
0,6358025	0	0	1	1	1
0,5944444	1	1	1	1	1
0,5235294	0	0	0	1	1
0,5413105	0	0	1	1	1
0,5886076	1	0	0	1	1
0,5818182	0	0	0	1	1
0,5402299	0	0	0	0	0
0,5535714	0	0	0	0	0
0,5655977	1	1	1	1	1
0,5395095	0	0	0	1	1
0,5536723	0	0	1	1	1
0,5348837	0	0	1	0	1
0,5649718	0	0	1	1	1
0,5762712	0	0	0	0	0
0,5376344	1	1	0	1	1

0,5953757	0	0	0	0	0
0,5413105	1	0	0	1	1
0,6037736	1	1	1	0	1
0,5500000	0	0	0	1	1
0,5582822	0	0	0	1	1
0,5697674	1	0	0	1	1
0,5611111	1	1	1	1	1
0,5730028	1	0	1	1	1
0,5828571	0	0	0	1	1
0,5593220	0	0	0	1	1
0,6071429	0	0	0	0	0
0,5941176	1	1	1	1	1
0,5747126	0	0	0	1	1
0,5865922	1	1	0	1	1
1,0228571	1	1	1	1	1
0,5266272	0	0	1	0	1
0,5056180	0	0	1	0	1
0,5314286	0	0	1	1	1
0,5136612	0	0	0	1	1
0,5297297	0	0	1	0	1
0,5310734	0	0	0	1	1
0,5397727	0	0	1	1	1
0,5257143	1	0	0	1	1
0,5096953	0	0	1	1	1
0,5474860	0	0	0	1	1
0,5406977	0	0	1	1	1
0,5523256	0	0	0	1	1
0,5599513	0	0	1	0	1
0,5619335	1	0	1	1	1
0,5426829	0	0	0	0	0
0,5390836	0	0	0	1	1
0,5187166	0	0	0	1	1
0,5248619	1	0	1	1	1
0,5819209	0	0	0	1	1
0,5755814	1	0	0	1	1
0,5433526	0	0	1	1	1
0,5828571	0	0	0	1	1
0,5555556	0	0	0	1	1
0,5804598	0	0	0	1	1
0,6024096	0	0	1	1	1
0,6114286	0	0	1	1	1
0,5882353	0	0	0	0	0
0,6329114	1	1	1	1	1
0,6136364	1	0	1	1	1
0,5697674	1	1	0	1	1
0,5885714	1	0	0	1	1
0,5142857	0	0	1	1	1
0,5565217	0	0	1	1	1
0,5321637	1	1	1	1	1
0,5152355	1	0	0	1	1

0,5562130	1	1	0	1	1
0,5542169	1	0	0	1	1
0,5417867	1	1	1	1	1
0,5439093	1	0	0	0	1
0,5517241	1	0	1	1	1
0,5329815	0	0	1	1	1
0,5549451	0	0	0	1	1
0,5423729	0	0	0	0	0
0,6033520	0	0	0	0	0
0,5941176	1	0	1	1	1
0,6111111	0	0	0	1	1
0,5755814	0	0	0	0	0
0,5936599	1	0	1	1	1
0,5838150	0	0	1	0	1
0,5810056	0	0	0	0	0
0,5507246	0	0	1	0	1
0,5722714	1	0	1	1	1
0,5696970	0	0	1	1	1
0,5628743	1	0	1	1	1
0,5459770	0	0	1	0	1
0,5502959	0	0	0	1	1
0,5630499	1	1	0	1	1
0,5674157	1	0	0	0	1
0,5280899	0	0	0	0	0
0,5657143	1	0	0	1	1
0,5511364	0	0	0	1	1
0,5828571	1	0	1	1	1
0,5872093	1	0	1	1	1
0,5355191	1	1	1	1	1
0,6011561	0	0	1	1	1
0,5776567	1	0	1	1	1
0,6011561	0	0	0	1	1
0,5729730	1	1	1	1	1
0,5568862	0	0	0	0	0
0,5828221	0	0	1	1	1
0,5454545	1	0	0	1	1
0,5344828	0	0	1	1	1
0,5698324	0	0	0	1	1
0,5900621	1	0	1	1	1
0,5604396	1	0	1	1	1
0,5698324	1	1	1	1	1
0,5769231	0	0	0	1	1
0,5895954	0	0	0	1	1
0,5647059	0	0	0	1	1
0,5523256	1	0	0	0	1
0,5705882	1	1	0	1	1
0,5465116	1	1	0	1	1
0,5806452	1	1	1	1	1
0,5681818	0	0	0	1	1
0,5789474	1	1	0	1	1

0,6049383	1	1	1	1	1
0,5800604	0	0	0	0	0
0,5393939	0	0	1	1	1
0,5485714	0	0	1	1	1
0,5277778	0	0	1	0	1
0,5565217	0	0	0	1	1
0,5647059	0	0	0	1	1
0,5289256	1	0	1	1	1
0,5416667	1	1	0	1	1
0,5391304	0	0	1	1	1
0,5454545	1	1	0	1	1
0,5810056	0	0	1	1	1
0,5562130	0	0	1	0	1
0,5459770	1	0	1	1	1
0,5714286	0	0	0	0	0
0,5473684	1	1	0	1	1
0,5730337	0	0	0	1	1
0,5762712	0	0	1	1	1
0,5683060	0	0	1	0	1
0,5780347	0	0	0	1	1
0,6149425	0	0	1	1	1
0,5643836	1	0	1	1	1
0,6121212	0	0	0	1	1
0,5780347	0	0	0	0	0
0,6069364	0	0	0	1	1
0,5939394	1	0	0	0	1
0,6024096	1	1	0	1	1
0,5416667	0	0	1	0	1
0,5397727	0	0	1	1	1
0,5040214	0	0	0	1	1
0,5542857	0	0	1	0	1
0,5689655	1	0	0	1	1
0,5348189	1	1	1	0	1
0,5215054	1	1	1	1	1
0,5764706	0	0	1	1	1
0,5654762	0	0	0	1	1
0,5875000	0	0	0	1	1
0,5780347	1	1	1	1	1
0,5511364	1	1	0	1	1
0,5444444	0	0	1	1	1
0,5649718	0	0	0	1	1
0,5581395	0	0	0	1	1
0,5593220	0	0	1	1	1
0,5895954	1	1	0	1	1
0,6149254	0	0	0	1	1
0,5988701	1	1	0	1	1
0,5608466	0	0	0	1	1
0,6144578	0	0	0	1	1
0,5833333	0	0	1	1	1
0,6079545	0	0	1	0	1

0,5057471	1	0	0	1	1
0,5272206	0	0	0	1	1
0,5280899	0	0	0	1	1
0,5416667	1	1	0	1	1
0,5840708	0	0	0	1	1
0,5937500	0	0	0	1	1
0,5561798	0	0	0	1	1
0,5786517	1	0	0	0	1
0,5775076	1	0	1	1	1
0,5917160	1	0	1	1	1
0,5945946	0	0	0	1	1
0,5838150	0	0	1	1	1
0,5681818	0	0	0	1	1
0,5655977	0	0	0	1	1
0,5597668	0	0	0	1	1
0,6045198	1	1	0	1	1
0,5882353	1	0	1	1	1
0,5617978	0	0	0	0	0
0,5639535	0	0	0	1	1
0,6250000	1	1	1	1	1
0,6277778	1	0	1	1	1
0,6424242	0	0	1	0	1
0,5706215	1	0	0	1	1
0,5161290	0	0	1	1	1
0,5497076	1	1	0	1	1
0,5508982	0	0	1	1	1
0,5542169	0	0	1	0	1
0,5251397	0	0	0	0	0
0,5405405	1	1	0	1	1
0,5356125	1	1	1	1	1
0,5545723	1	0	1	1	1
0,5777778	1	0	0	1	1
0,5600000	0	0	0	1	1
0,5833333	0	0	1	1	1
0,5517241	0	0	0	1	1
0,6034483	0	0	0	1	1
0,5852273	0	0	0	1	1
0,6057143	1	0	0	1	1
0,5449438	1	1	1	1	1
0,5470588	1	0	1	1	1
0,5523256	1	1	1	1	1
0,5611940	1	1	1	1	1
0,5357143	1	0	1	1	1
0,5427729	1	0	1	1	1
0,5212766	0	0	0	1	1
0,5449102	1	1	1	0	1
0,5722543	1	0	1	1	1
0,5771429	1	1	0	1	1
0,5367232	0	0	0	1	1
0,5993884	1	0	0	1	1

0,5696970	1	1	1	1	1
0,5730994	1	1	0	1	1
0,5875706	1	0	0	1	1
0,5524862	0	0	1	0	1
0,5976331	0	0	0	1	1
0,5500000	0	0	0	1	1
0,6094675	1	1	1	0	1
0,5670103	0	0	0	1	1
0,5625000	1	1	1	1	1
0,5771429	0	0	0	1	1
0,5895954	0	0	0	0	0
0,6386861	1	0	1	1	1
0,5942857	1	1	0	1	1
0,6627219	0	0	1	1	1
0,5487805	1	0	0	0	1
0,5280899	1	0	1	1	1
0,5502959	1	0	0	1	1
0,5142857	0	0	0	1	1
0,5637982	0	0	1	1	1
0,5546667	0	0	0	0	0
0,5689655	0	0	0	1	1
0,5941176	1	0	0	1	1
0,5917160	0	0	1	1	1
0,5617978	0	0	1	1	1
0,5657143	1	0	1	1	1
0,5842697	1	1	0	1	1
0,5828221	0	0	0	1	1
0,5574713	1	1	1	1	1
0,5397727	1	1	1	1	1
0,5730994	1	0	0	1	1
0,5764706	0	0	1	1	1
0,5454545	0	0	0	1	1
0,5469613	1	1	0	1	1
0,5630499	1	0	1	1	1
0,5868946	1	0	0	1	1
0,5813953	1	0	1	1	1
0,5810056	0	0	1	1	1
0,5696970	1	0	1	1	1
0,5523256	1	1	0	1	1
0,5274725	0	0	0	1	1
0,5722543	0	0	1	1	1
0,5480226	1	1	1	1	1
0,5588235	0	0	1	1	1
0,5818182	0	0	1	1	1
0,5581395	0	0	0	1	1
0,5698006	0	0	0	1	1
0,5964912	0	0	0	1	1
0,5529412	0	0	0	1	1
0,5852273	1	1	0	1	1
0,5714286	0	0	1	1	1

0,5745856	0	0	1	1	1
0,5792683	0	0	0	1	1
0,6389776	0	0	1	1	1
0,5166667	0	0	0	0	0
0,5628743	1	1	0	1	1
0,5114943	1	0	1	1	1
0,5555556	0	0	0	1	1
0,5889571	1	0	0	1	1
0,5632184	0	0	0	1	1
0,5497076	1	1	1	1	1
0,5595238	1	1	1	1	1
0,5813953	0	0	0	0	0
0,5555556	0	0	0	0	0
0,5965909	0	0	1	1	1
0,5497076	0	0	0	1	1
0,5376344	0	0	0	1	1
0,5696970	0	0	1	0	1
0,5777778	1	0	1	1	1
0,5988701	1	0	1	1	1
0,5857988	1	1	1	1	1
0,5714286	0	0	0	1	1
0,6107784	0	0	1	1	1
0,6121212	0	0	1	1	1
0,5642458	1	0	1	1	1
0,5810056	0	0	0	1	1
0,5348837	0	0	0	0	0
0,5277778	0	0	0	1	1
0,5722892	1	1	1	1	1
0,5517241	0	0	1	1	1
0,5438596	0	0	0	1	1
0,5555556	0	0	0	1	1
0,5889571	1	1	1	1	1
0,5833333	1	0	1	1	1
0,5813953	1	0	0	0	1
0,5810056	0	0	1	1	1
0,5642458	1	1	1	1	1
0,5604720	1	0	1	1	1
0,5561798	0	0	0	1	1
0,5771429	0	0	0	1	1
0,5879121	0	0	1	1	1
0,5722892	0	0	0	1	1
0,5780347	0	0	0	1	1
0,5536723	0	0	0	1	1
0,6385542	1	0	1	1	1
0,5659341	1	1	1	1	1
0,5271739	1	0	0	1	1
0,5337079	0	0	1	1	1
0,5632184	0	0	0	0	0
0,5654762	0	0	1	1	1
0,5609756	0	0	0	1	1

0,5585586	0	0	1	1	1
0,5535714	1	0	0	1	1
0,6000000	0	0	0	1	1
0,5508982	1	1	1	1	1
0,5777778	1	0	1	0	1
0,5941176	1	0	1	1	1
0,5882353	1	0	0	1	1
0,5722222	1	0	1	1	1
0,5819209	1	1	0	1	1
0,5771429	0	0	0	1	1
0,5517241	0	0	1	1	1
0,5714286	1	1	1	1	1
0,5649718	1	0	1	1	1
0,5828571	0	0	1	1	1
0,5823529	0	0	0	1	1
0,5344828	0	0	0	1	1
0,5314286	0	0	0	1	1
0,5449438	1	1	0	1	1
0,5294118	0	0	1	1	1
0,5593220	0	0	0	1	1
0,5459770	0	0	0	1	1
0,5340909	1	0	0	0	1
0,5632184	0	0	1	0	1
0,5748503	0	0	0	0	0
0,5780347	0	0	1	1	1
0,5555556	1	0	0	0	1
0,5969231	0	0	0	1	1
0,5623188	0	0	0	1	1
0,5941176	1	0	0	1	1
0,5584046	1	0	0	1	1
0,5714286	0	0	0	1	1
0,5862069	0	0	0	1	1
0,5862069	1	1	0	1	1
0,5808383	1	1	0	1	1
0,5919540	0	0	0	1	1
0,5606936	0	0	1	0	1
0,5722222	1	0	1	1	1
0,5872093	0	0	0	1	1
0,5689655	0	0	1	1	1
0,5287356	1	1	0	1	1
0,5459770	0	0	1	1	1
0,5310734	0	0	0	1	1
0,5602410	0	0	0	1	1
0,5475504	1	1	0	1	1
0,5616046	1	0	1	0	1
0,5280899	1	0	1	1	1
0,5428571	1	0	1	1	1
0,5789474	0	0	1	1	1
0,5672515	1	1	1	1	1
0,5748503	1	0	0	1	1

0,5795455	0	0	0	1	1
0,5885714	1	0	0	1	1
0,5789474	0	0	1	1	1
0,5500000	0	0	1	1	1
0,5976331	0	0	0	1	1
0,6114286	0	0	0	1	1
0,5730994	0	0	1	1	1
0,5828571	1	0	1	1	1
0,5706215	0	0	0	0	0
0,5763689	0	0	1	1	1
0,5994065	0	0	0	1	1
0,6437500	0	0	0	1	1
0,5813953	1	0	0	1	1
0,5810056	1	0	0	1	1
0,5142857	0	0	1	1	1
0,5135135	0	0	1	1	1
0,5227273	1	0	0	1	1
0,5376344	1	0	1	1	1
0,5841270	0	0	0	0	0
0,5657143	0	0	1	1	1
0,5513514	0	0	0	1	1
0,5804598	1	1	1	1	1
0,5697674	0	0	0	1	1
0,5449438	0	0	1	1	1
0,5823529	1	0	1	0	1
0,6049383	0	0	0	1	1
0,5508021	1	0	0	1	1
0,5639535	0	0	0	0	0
0,5892857	1	1	1	1	1
0,5523256	1	0	1	1	1
0,5989011	1	0	0	1	1
0,6227545	1	0	1	1	1
0,6024845	1	0	1	1	1
0,6005666	1	0	0	1	1
0,6477273	1	1	1	1	1
0,5621302	1	1	0	1	1
0,5621302	0	0	0	1	1
0,5454545	1	0	0	1	1
0,5964912	0	0	1	0	1
0,5862069	0	0	0	1	1
0,5159574	1	0	1	0	1
0,5878788	1	0	0	1	1
0,5689655	0	0	0	0	0
0,5795455	1	1	1	1	1
0,5824176	1	0	0	1	1
0,6190476	1	0	1	1	1
0,5962060	0	0	0	0	0
0,5248619	0	0	1	1	1
0,5705882	0	0	1	1	1
0,5277778	1	0	1	1	1

0,5444444	0	0	1	1	1
0,5497076	0	0	0	1	1
0,5764706	1	1	1	1	1
0,5878788	0	0	1	1	1
0,5268817	0	0	0	1	1
0,5549133	0	0	0	1	1
0,5635359	0	0	0	1	1
0,5662651	1	0	0	1	1
0,5224719	0	0	1	1	1
0,5113924	1	0	0	1	1
0,5414365	0	0	1	1	1
0,5846995	1	0	1	1	1
0,6231454	0	0	0	1	1
0,6312500	0	0	1	1	1
0,5885714	1	0	1	1	1
0,5956284	1	0	0	1	1
0,5921788	1	0	0	1	1
0,6250000	1	0	1	1	1
0,5465116	1	1	1	1	1
0,5571848	0	0	0	1	1
0,5527066	0	0	1	1	1
0,5333333	1	1	0	0	1
0,5739645	1	1	0	1	1
0,5368732	1	1	1	1	1
0,5680473	0	0	1	0	1
0,5549451	0	0	1	0	1
0,5639535	0	0	1	1	1
0,5595238	1	1	0	1	1
0,5542169	0	0	0	1	1
0,6036585	0	0	0	0	0
0,5919540	1	0	1	0	1
0,5909091	1	0	1	1	1
0,6219512	1	1	1	1	1
0,6011561	1	0	0	1	1
0,6125000	0	0	0	1	1
0,6607143	1	1	0	1	1
0,5294118	1	0	1	0	1
0,5367232	0	0	0	1	1
0,5269461	0	0	1	1	1
0,5582822	0	0	0	1	1
0,5113636	0	0	0	1	1
0,5530726	1	1	0	1	1
0,5617978	1	1	1	1	1
0,5798817	0	0	0	1	1
0,5696970	0	0	0	1	1
0,5722543	1	1	0	1	1
0,5470588	1	1	1	1	1
0,5731707	0	0	1	1	1
0,5819209	0	0	1	1	1
0,5297297	1	0	1	0	1

0,5847953	0	0	0	1	1
0,5895954	0	0	0	0	0
0,5988372	0	0	1	1	1
0,5714286	1	0	0	1	1
0,6057143	1	0	1	0	1
0,5675676	1	0	0	1	1
0,6123596	1	0	1	1	1
0,6153846	1	1	1	1	1
0,5391304	0	0	1	1	1
0,5388889	0	0	0	1	1
0,5497076	0	0	0	1	1
0,5705882	1	0	0	1	1
0,5792683	0	0	1	1	1
0,5755814	0	0	1	1	1
0,5549133	0	0	1	1	1
0,5657143	1	1	1	1	1
0,5642458	0	0	0	1	1
0,5470588	0	0	1	0	1
0,5683060	0	0	1	1	1
0,5647059	0	0	0	1	1
0,5804598	1	1	0	1	1
0,5705882	0	0	0	1	1
0,5705882	0	0	1	1	1
0,5367232	1	0	0	1	1
0,5540166	1	0	0	1	1
0,5842697	0	0	0	1	1
0,6158358	0	0	0	0	0
0,6250000	1	0	1	1	1
0,5813953	1	0	1	1	1
0,6181818	0	0	0	1	1
0,5535714	1	1	1	1	1
0,5549133	0	0	1	1	1
0,5421687	1	0	0	1	1
0,5714286	1	1	0	1	1
0,5352941	0	0	1	1	1
0,5732087	0	0	1	1	1
0,5722543	0	0	0	1	1
0,6104651	1	1	1	1	1
0,6005666	0	0	1	1	1
0,6342857	0	0	1	1	1
0,5000000	0	0	0	1	1
0,5654762	0	0	0	1	1
0,5535714	1	0	0	1	1
0,5810398	0	0	0	1	1
0,5480226	0	0	0	1	1
0,5697674	1	0	1	1	1
0,5964912	0	0	1	1	1
0,6130952	0	0	0	1	1
0,5798817	0	0	0	0	0
0,6112760	0	0	0	0	0

0,6121212	1	1	1	1	1
0,5745856	1	0	0	1	1
0,6117647	1	0	0	1	1
0,5754986	0	0	1	0	1
0,6140351	0	0	1	1	1
0,5976331	1	0	0	1	1
0,5977011	1	0	0	1	1
0,6000000	0	0	1	0	1
0,5989011	0	0	1	0	1
0,6363636	0	0	1	1	1
0,5588235	1	0	1	1	1
0,5391304	1	1	1	1	1
0,5578635	1	1	1	1	1
0,5911950	1	1	1	1	1
0,5758514	0	0	0	1	1
0,5706215	1	0	0	1	1
0,5812500	1	1	1	1	1
0,5611111	0	0	0	1	1
0,5952381	0	0	0	1	1
0,5434783	0	0	1	1	1
0,5459770	1	0	0	1	1
0,5604396	1	0	1	1	1
0,5975197	0	0	1	1	1
0,5942857	1	1	0	1	1
0,6424242	0	0	1	1	1
0,5303867	1	0	1	1	1
0,5367232	0	0	0	1	1
0,5459770	0	0	0	0	0
0,5524862	1	1	1	1	1
0,5505618	0	0	0	1	1
0,5748503	0	0	1	1	1
0,5617978	1	0	0	1	1
0,5773810	0	0	1	1	1
0,5593220	0	0	1	0	1
0,5798817	0	0	0	1	1
0,5513514	1	0	1	1	1
0,5975610	1	1	0	1	1
0,5843373	0	0	0	1	1
0,5847953	0	0	1	1	1
0,5953757	0	0	0	1	1
0,5987654	0	0	1	1	1
0,5511364	0	0	0	1	1
0,5952381	0	0	0	0	0
0,5562130	1	0	0	1	1
0,6058824	1	0	0	1	1
0,5977011	1	0	1	1	1
0,5911602	0	0	0	1	1
0,6097561	0	0	0	1	1
0,6079545	1	1	0	1	1
0,6445783	0	0	1	1	1

0,6045198	0	0	1	1	1
0,5443787	0	0	0	1	1
0,5738636	1	0	1	0	1
0,5568182	0	0	0	1	1
0,5433526	1	0	0	0	1
0,5649718	1	1	1	1	1
0,5731707	0	0	0	1	1
0,5586592	0	0	0	1	1
0,6000000	1	0	0	1	1
0,5568182	0	0	1	1	1
0,5942857	0	0	0	1	1
0,5739645	0	0	0	0	0
0,6058824	1	0	0	1	1
0,5941176	0	0	0	1	1
0,6071429	1	1	1	1	1
0,5966851	0	0	0	1	1
0,6163522	0	0	0	1	1
0,5168539	0	0	0	1	1
0,5714286	1	0	1	1	1
0,5567568	1	0	1	1	1
0,6220930	0	0	1	1	1
0,5843373	1	1	0	1	1
0,6176471	1	1	1	1	1
0,5521127	1	0	0	1	1
0,5581395	1	0	1	0	1
0,5697674	0	0	0	1	1
0,5623342	1	1	0	1	1
0,5739645	1	1	1	1	1
0,5847953	0	0	1	1	1
0,5898876	0	0	1	1	1
0,5934718	0	0	0	1	1
0,5842697	0	0	1	0	1
0,5898876	0	0	1	1	1
0,6011236	1	0	0	1	1
0,5942857	0	0	1	1	1
0,6000000	0	0	1	1	1
0,5287356	0	0	0	1	1
0,5352941	0	0	1	1	1
0,5654762	0	0	1	1	1
0,5657143	1	0	0	0	1
0,5697674	0	0	0	1	1
0,5681818	0	0	1	0	1
0,5833333	0	0	0	0	0
0,5738636	0	0	0	1	1
0,5885714	0	0	1	1	1
0,5771429	0	0	1	1	1
0,5430108	1	0	1	1	1
0,5852273	0	0	1	1	1
0,5942857	0	0	1	1	1
0,5852273	0	0	1	1	1

0,5911602	0	0	1	1	1
0,5892857	0	0	0	1	1
0,5919540	0	0	0	1	1
0,6265060	0	0	0	1	1
0,6045198	0	0	0	1	1
0,6097561	0	0	1	1	1
0,5680473	0	0	1	1	1
0,5548780	0	0	0	1	1
0,5523256	1	0	0	0	1
0,5714286	1	0	0	1	1
0,5698324	0	0	0	1	1
0,5628743	1	0	0	1	1
0,5359116	0	0	0	1	1
0,5801105	1	0	1	1	1
0,6100629	1	1	0	1	1
0,6385542	0	0	1	1	1
0,6264368	0	0	0	1	1
0,5555556	0	0	1	1	1
0,5378151	1	0	0	1	1
0,5517241	0	0	0	1	1
0,5548780	1	1	0	1	1
0,5588235	1	0	1	1	1
0,5714286	0	0	0	1	1
0,5644172	1	0	0	1	1
0,5406977	1	0	0	1	1
0,6000000	0	0	0	1	1
0,5762712	1	0	1	1	1
0,6000000	1	0	1	1	1
0,6220930	1	0	1	1	1
0,5244957	1	1	0	1	1
0,5561798	0	0	0	1	1
0,5555556	1	0	1	1	1
0,5485714	1	0	0	1	1
0,5838150	1	0	0	1	1
0,5580110	0	0	1	1	1
0,5857988	0	0	1	0	1
0,5875706	1	0	0	1	1
0,5976331	0	0	1	1	1
0,6022727	1	0	0	1	1
0,5963855	0	0	1	1	1
0,6011561	0	0	1	1	1
0,5491329	0	0	0	1	1
0,5310734	0	0	1	1	1
0,5459770	0	0	0	1	1
0,5823529	0	0	0	1	1
0,5722892	1	1	0	1	1
0,5921450	0	0	0	1	1
0,5714286	1	1	1	0	1
0,5593220	0	0	0	0	0
0,5542857	1	0	0	1	1

0,5705882	0	0	0	1	1
0,5838150	1	1	0	1	1
0,5838150	1	1	1	1	1
0,5838150	1	0	0	0	1
0,6337209	0	0	0	1	1
0,5271739	0	0	1	1	1
0,5766871	0	0	1	1	1
0,5474860	1	0	1	1	1
0,5569620	0	0	1	1	1
0,5800604	0	0	0	1	1
0,5562130	1	0	1	1	1
0,5962733	0	0	0	1	1
0,5852273	1	0	0	0	1
0,5568182	1	0	0	1	1
0,5600000	0	0	1	1	1
0,5875706	0	0	0	0	0
0,5833333	0	0	1	1	1
0,5842697	0	0	0	0	0
0,5885714	1	1	1	1	1
0,5611111	0	0	0	1	1
0,5879121	0	0	1	1	1
0,6094675	1	0	0	1	1
0,6117647	1	1	1	1	1
0,6144578	0	0	0	1	1
0,6046512	1	1	1	1	1
0,6158192	0	0	1	1	1
0,6845638	0	0	0	0	0
0,5449438	0	0	1	1	1
0,5469613	1	0	0	0	1
0,5823529	0	0	0	1	1
0,5565217	1	1	0	1	1
0,5878788	0	0	1	1	1
0,5840708	0	0	1	1	1
0,5845272	1	0	1	1	1
0,5838150	1	0	0	1	1
0,5857988	0	0	0	1	1
0,6358025	0	0	1	1	1
0,6114286	0	0	0	1	1
0,6325301	1	1	1	1	1
0,5609756	0	0	0	0	0
0,5625000	0	0	1	1	1
0,5555556	0	0	1	1	1
0,5500000	1	0	0	0	1
0,5639535	1	1	1	1	1
0,5823529	0	0	0	1	1
0,5591398	1	0	0	0	1
0,6097561	1	0	1	1	1
0,5852273	0	0	1	1	1
0,5932203	0	0	0	1	1
0,5857988	1	0	0	1	1

0,5944444	1	0	0	1	1
0,6091954	1	1	1	1	1
0,5714286	0	0	0	1	1
0,6104651	1	1	1	1	1
0,5994236	1	1	1	1	1
0,6091954	0	0	1	1	1
0,6285714	1	1	0	1	1
0,5433526	0	0	0	1	1
0,5903614	0	0	1	1	1
0,5680473	0	0	1	1	1
0,5689655	1	1	1	1	1
0,5862069	0	0	0	1	1
0,5505618	0	0	1	1	1
0,6049383	1	0	1	1	1
0,5574713	0	0	1	1	1
0,5568182	0	0	0	1	1
0,6097561	0	0	0	1	1
0,5639535	1	0	1	1	1
0,5777778	0	0	0	1	1
0,6220930	0	0	0	1	1
0,5988024	1	0	0	1	1
0,6337209	0	0	0	1	1
0,5730337	0	0	1	1	1
0,5852273	0	0	1	1	1
0,5764706	1	0	1	0	1
0,5917160	0	0	0	0	0
0,5942857	0	0	0	1	1
0,5810056	1	0	1	1	1
0,5617978	1	0	0	1	1
0,5941176	0	0	1	1	1
0,6057143	0	0	0	1	1
0,6091954	1	1	0	1	1
0,6022727	0	0	1	1	1
0,6250000	1	0	0	1	1
0,5697674	0	0	0	1	1
0,5771429	1	0	0	1	1
0,5665722	0	0	0	1	1
0,5489130	0	0	0	1	1
0,6006006	1	0	1	1	1
0,5806452	0	0	1	1	1
0,6023392	1	0	1	1	1
0,5806452	0	0	1	1	1
0,6024096	1	0	1	1	1
0,6144578	0	0	0	1	1
0,5977011	1	0	1	1	1
0,5164835	0	0	1	1	1
0,5161290	0	0	1	1	1
0,5491329	0	0	0	1	1
0,5439093	0	0	1	1	1
0,5645646	1	1	1	1	1

0,5777778	0	0	0	1	1
0,6000000	0	0	0	1	1
0,5977011	1	0	1	1	1
0,5847953	1	0	0	0	1
0,5357143	0	0	0	1	1
0,5731343	0	0	0	1	1
0,5487122	0	0	1	1	1
0,5907692	0	0	1	1	1
0,5952381	1	0	0	0	1
0,5726092	0	0	0	1	1
0,5942857	1	0	0	1	1
0,5795455	0	0	0	1	1
0,6096866	1	0	0	1	1
0,5224719	1	1	0	1	1
0,5722543	1	0	1	1	1
0,6000000	1	0	1	1	1
0,5976331	1	0	1	1	1
0,5824176	1	1	1	1	1
0,5681818	1	1	0	1	1
0,6077348	0	0	1	1	1
0,5906433	0	0	0	1	1
0,5310734	1	0	0	1	1
0,5773810	0	0	0	1	1
0,5628743	1	0	0	1	1
0,5730337	1	0	0	1	1
0,5779037	1	0	1	1	1
0,5689150	0	0	0	1	1
0,5698324	1	1	1	1	1
0,5959885	0	0	1	1	1
0,5802817	0	0	1	1	1
0,6104651	0	0	0	0	0
0,5747126	1	1	1	0	1
0,6023392	0	0	1	1	1
0,5941176	1	0	0	0	1
0,6000000	0	0	1	1	1
0,5789474	0	0	1	1	1
0,5600000	1	1	0	1	1
0,5730994	1	1	0	1	1
0,5780347	0	0	1	1	1
0,5771429	0	0	1	1	1
0,5771429	0	0	0	1	1
0,5666667	0	0	0	1	1
0,5875706	0	0	1	1	1
0,5810056	0	0	1	1	1
0,6000000	0	0	1	0	1
0,6235294	0	0	0	1	1
0,5359116	0	0	1	1	1
0,5900621	0	0	0	1	1
0,5988372	0	0	1	0	1
0,5977011	0	0	0	1	1

0,5945946	0	0	1	1	1
0,6198830	0	0	0	1	1
0,5481050	0	0	1	1	1
0,5602410	1	0	0	1	1
0,5950920	0	0	1	1	1
0,5842697	0	0	0	1	1
0,5474860	1	0	0	1	1
0,6011905	1	1	0	0	1
0,5548780	1	0	0	1	1
0,5963855	1	0	0	1	1
0,6204819	1	0	0	0	1
0,6196319	0	0	0	1	1
0,6190476	1	0	0	1	1
0,6058824	0	0	0	1	1
0,6005831	0	0	1	1	1
0,6300578	0	0	0	1	1
0,5471125	1	0	1	1	1
0,5028571	0	0	1	1	1
0,5555556	1	0	1	1	1
0,5802817	0	0	1	0	1
0,5862069	0	0	0	1	1
0,6097561	0	0	1	1	1
0,5878788	1	1	1	1	1
0,6011561	1	1	0	1	1
0,6296296	0	0	0	1	1
0,5913978	0	0	0	1	1
0,6069364	1	0	1	1	1
0,6130952	1	0	0	0	1
0,6264368	1	0	1	1	1
0,5955056	1	0	0	1	1
0,6162162	1	1	1	1	1
0,5406977	1	0	1	0	1
0,5614035	0	0	1	1	1
0,5818182	0	0	1	1	1
0,5885714	1	0	0	1	1
0,5738636	1	0	0	0	1
0,6011561	1	1	1	1	1
0,5847953	0	0	0	1	1
0,5930233	1	1	1	1	1
0,5752688	1	1	1	1	1
0,6369048	1	1	0	1	1
0,5747801	1	0	0	0	1
0,5530726	0	0	0	1	1
0,6000000	0	0	0	1	1
0,6136364	0	0	0	0	0
0,6380368	1	1	0	1	1
0,6588235	1	1	1	1	1
0,5562130	0	0	1	1	1
0,5609756	0	0	0	1	1
0,5952381	0	0	0	1	1

0,5899705	1	1	1	1	1
0,6046512	1	0	0	0	1
0,5277778	1	1	0	0	1
0,5705521	0	0	1	1	1
0,5993884	0	0	0	1	1
0,5932203	0	0	0	1	1
0,5524862	0	0	1	1	1
0,6666667	1	1	0	1	1
0,5909091	1	0	0	1	1
0,6196319	1	0	0	1	1
0,5714286	0	0	1	1	1
0,6298343	0	0	0	0	0
0,5515152	1	1	0	1	1
0,6011236	1	0	0	1	1
0,6034483	1	0	1	0	1
0,6114286	0	0	1	1	1
0,6257310	0	0	1	0	1
0,5780347	1	1	1	0	1
0,5590778	1	0	0	1	1
0,5988701	0	0	0	1	1
0,5665722	1	1	1	1	1
0,6257310	0	0	0	1	1
0,6049046	1	0	0	1	1
0,6162791	1	1	1	1	1
0,6467066	0	0	1	1	1
0,5833333	0	0	0	1	1
0,5397727	1	1	0	1	1
0,5388889	0	0	1	1	1
0,6005831	0	0	0	1	1
0,5878788	0	0	1	1	1
0,5879121	0	0	1	1	1
0,6167665	1	0	1	0	1
0,5891892	1	1	1	1	1
0,6250000	0	0	0	1	1
0,6227545	0	0	0	1	1
0,5260116	1	1	1	1	1
0,5586592	1	0	1	1	1
0,5698324	0	0	0	0	0
0,5819209	1	1	0	1	1
0,6250000	0	0	0	1	1
0,5898876	0	0	0	1	1
0,6184971	0	0	0	1	1
0,6176471	1	0	0	1	1
0,6172840	0	0	1	0	1
0,5202312	0	0	1	1	1
0,5662651	1	0	0	1	1
0,5898876	0	0	0	1	1
0,5670103	0	0	1	1	1
0,6045198	1	1	0	1	1
0,5945946	0	0	0	0	0

0,6300578	1	1	1	1	1
0,5357143	1	1	1	1	1
0,5747126	0	0	0	1	1
0,6411765	1	1	0	1	1
0,5714286	0	0	1	1	1
0,5617978	1	0	1	1	1
0,5882353	0	0	0	1	1
0,5917160	1	0	0	1	1
0,6352201	0	0	1	1	1
0,6179775	0	0	1	0	1
0,5869565	0	0	1	1	1
0,5783133	0	0	1	1	1
0,5679012	1	0	1	1	1
0,5789474	0	0	1	1	1
0,6181818	0	0	1	1	1
0,5681818	1	0	0	0	1
0,6094675	0	0	0	1	1
0,6184971	0	0	1	1	1
0,5856354	0	0	0	1	1
0,5872093	1	0	0	1	1
0,6531792	1	1	1	1	1
0,5581395	0	0	1	0	1
0,5755814	1	0	0	1	1
0,5971014	1	0	1	1	1
0,5977011	1	1	0	1	1
0,5988701	0	0	0	1	1
0,6084337	0	0	1	1	1
0,6010638	1	0	1	1	1
0,5953757	0	0	0	1	1
0,5757576	0	0	0	1	1
0,6000000	1	1	0	1	1
0,5975610	1	1	1	0	1
0,5988372	0	0	1	1	1
0,6257310	1	1	1	1	1
0,6067416	1	0	1	1	1
0,6300578	1	1	1	1	1
0,5706052	0	0	1	1	1
0,5977011	1	0	0	1	1
0,5389222	1	1	1	1	1
0,5193370	1	1	0	1	1
0,5705882	1	0	0	1	1
0,5965909	0	0	1	1	1
0,5988024	0	0	1	0	1
0,6337209	1	1	0	1	1
0,5773810	0	0	1	1	1
0,5698324	0	0	0	1	1
0,5917160	0	0	1	1	1
0,6107784	1	1	1	1	1
0,6144578	1	1	0	1	1
0,5977011	0	0	0	1	1

0,5959885	1	1	1	1	1
0,6213018	0	0	0	1	1
0,5647059	0	0	0	1	1
0,5944444	1	1	0	1	1
0,6034483	1	0	0	1	1
0,6000000	1	0	0	1	1
0,6273292	0	0	0	1	1
0,6162791	0	0	1	1	1
0,6420455	0	0	1	0	1
0,6089744	1	0	1	1	1
0,5806452	1	0	0	1	1
0,5714286	1	1	1	1	1
0,5828221	0	0	1	0	1
0,5748503	1	1	1	1	1
0,5652174	0	0	0	1	1
0,5775401	1	0	1	1	1
0,5847953	1	1	0	1	1
0,5786517	0	0	0	1	1
0,6140351	1	1	0	1	1
0,6011236	0	0	1	0	1
0,5491329	0	0	1	0	1
0,6162791	0	0	0	1	1
0,5787966	1	1	0	1	1
0,5639535	0	0	1	0	1
0,5898876	1	0	1	1	1
0,5988701	0	0	1	1	1
0,5297619	1	1	0	0	1
0,6300578	1	1	1	1	1
0,5739645	0	0	0	1	1
0,6062500	1	0	0	1	1
0,6067416	0	0	1	1	1
0,6352941	1	1	1	1	1
0,5314286	0	0	0	1	1
0,5833333	1	1	1	1	1
0,5329513	1	1	1	1	1
0,6374269	1	1	1	1	1
0,6606061	1	1	0	1	1
0,5263158	0	0	0	1	1
0,5470588	1	1	1	1	1
0,5939394	0	0	0	1	1
0,5439560	1	0	0	1	1
0,5755814	1	0	1	1	1
0,5028249	0	0	1	1	1
0,5416667	0	0	0	1	1
0,6347305	0	0	1	1	1
0,5491329	0	0	1	1	1
0,6054054	1	1	0	1	1
0,5375723	1	1	0	1	1
0,5290698	1	0	0	0	1
0,5416667	0	0	0	1	1

0,5862069	1	1	1	1	1
0,5542857	1	0	1	1	1
0,5722222	0	0	0	1	1
0,6250000	0	0	1	1	1
0,5956284	1	0	0	1	1
0,5558739	1	0	1	1	1
0,5605096	1	1	0	1	1
0,5232558	0	0	0	0	0
0,5813953	0	0	0	1	1
0,6058824	1	0	0	1	1
0,5232558	1	0	0	1	1
0,6428571	1	0	0	1	1
0,5113636	0	0	0	1	1
0,5705882	0	0	0	1	1
0,6309524	0	0	0	1	1
0,5885714	0	0	1	1	1
0,5900621	0	0	0	0	0
0,5474860	1	1	1	1	1
0,5418994	1	0	0	1	1
0,6250000	0	0	1	1	1
0,5885714	1	1	0	1	1
0,5536723	0	0	0	1	1
0,5664740	0	0	1	1	1
0,5647059	1	0	1	1	1
0,5977654	0	0	0	1	1
0,6918239	0	0	0	1	1
0,5317919	1	0	0	0	1
0,5654762	1	0	1	1	1
0,5561798	0	0	0	1	1
0,5055556	1	0	0	0	1
0,5268817	0	0	0	1	1
0,5505618	0	0	1	1	1
0,5621302	0	0	1	1	1
0,4820513	1	0	0	0	1
0,5500000	0	0	1	1	1
0,6024096	0	0	0	1	1
0,6171429	0	0	1	1	1
0,5771429	1	0	1	1	1
0,6190476	1	1	1	1	1
0,5580110	0	0	1	1	1
0,5517241	1	0	1	1	1
0,6153846	0	0	1	1	1
0,5280899	1	1	1	1	1
0,5917160	0	0	1	1	1
0,5202312	0	0	0	0	0
0,6303030	0	0	0	1	1
0,5432099	0	0	0	1	1
0,5555556	0	0	1	0	1
0,5649718	0	0	0	1	1
0,5977011	0	0	0	1	1

0,5388889	0	0	0	1	1
0,5606936	1	0	1	1	1
0,5689655	1	1	1	1	1
0,5898876	0	0	0	0	0
0,6250000	0	0	0	0	0
0,5842697	1	0	0	1	1
0,5494505	1	1	1	1	1
0,5748503	0	0	0	1	1
0,5780347	1	0	1	1	1
0,5857988	1	0	1	1	1
0,5909091	0	0	1	1	1
0,5586592	1	0	1	1	1
0,6277778	0	0	0	1	1
0,5914634	0	0	1	1	1
0,5505618	0	0	1	1	1
0,6117647	0	0	0	1	1
0,6626506	1	1	1	1	1
0,6011561	1	1	1	1	1
0,6460674	1	1	1	1	1
0,5549133	1	0	0	1	1
0,6136364	1	0	1	0	1
0,5889213	1	0	0	1	1
0,6666667	0	0	0	0	0
0,5415385	1	1	1	1	1
0,5600000	1	0	1	0	1
0,5266272	1	0	0	1	1
0,5402299	0	0	0	1	1
0,5895954	0	0	0	1	1
0,6775956	1	0	0	1	1
0,6347305	0	0	1	1	1
0,5421687	1	1	0	0	1
0,6508876	1	0	1	1	1
0,6707317	1	1	1	1	1
0,6058824	0	0	0	1	1
0,6287425	1	0	1	1	1
0,6804734	0	0	1	1	1
0,6946108	1	1	1	1	1
0,5389222	1	0	0	0	1
0,6470588	1	0	1	1	1
0,5882353	1	0	0	1	1
0,6055556	1	1	1	1	1
0,6235294	0	0	0	1	1
0,6052632	1	1	1	1	1
0,5123967	1	1	1	1	1
0,5902579	0	0	1	1	1
0,5485714	0	0	1	0	1
0,5706215	0	0	0	1	1
0,6279683	0	0	1	0	1
0,5737705	0	0	0	1	1
0,5977011	1	1	1	1	1

0,6193182	0	0	1	1	1
0,6363636	1	0	0	1	1
0,5491329	0	0	1	1	1
0,5722543	0	0	1	1	1
0,6130952	1	0	1	1	1
0,5838509	0	0	0	1	1
0,6171429	1	0	1	1	1
0,5697674	0	0	1	1	1
0,6073620	0	0	0	1	1
0,5882353	1	0	1	1	1
0,5898876	0	0	0	1	1
0,6358696	1	0	1	1	1
0,6079545	0	0	1	1	1
0,6257669	0	0	1	1	1
0,6021505	1	0	1	1	1
0,5521127	1	0	0	1	1
0,5868263	0	0	1	1	1
0,5857988	1	1	0	1	1
0,5406977	0	0	1	1	1
0,5771429	0	0	1	1	1
0,5786517	0	0	1	1	1
0,6193182	0	0	1	1	1
0,6257310	1	0	0	1	1
0,5597668	0	0	0	1	1
0,6097561	0	0	0	0	0
0,6214689	1	0	1	1	1
0,5212766	0	0	0	1	1
0,5722380	1	1	1	1	1
0,6337209	0	0	1	1	1
0,5930233	1	0	1	1	1
0,5738636	0	0	0	1	1
0,6424242	0	0	0	0	0
0,6534091	0	0	1	1	1
0,6611111	1	1	1	1	1
0,5474860	1	0	1	1	1
0,6049383	0	0	0	1	1
0,5835694	0	0	0	0	0
0,5956284	0	0	0	1	1
0,6181818	0	0	1	1	1
0,6220930	1	0	1	1	1
0,5588235	0	0	0	1	1
0,5600000	0	0	0	1	1
0,5847953	1	1	1	1	1
0,5965909	0	0	0	1	1
0,5828571	0	0	0	0	0
0,5649718	0	0	1	1	1
0,5872093	0	0	0	1	1
0,5862069	1	0	0	1	1
0,6261981	1	1	1	1	1
0,6242424	1	0	1	1	1

0,5418994	0	0	1	1	1
0,5696970	1	1	1	1	1
0,6084337	0	0	1	1	1
0,5675676	0	0	1	1	1
0,5729167	1	0	0	1	1
0,6055556	1	0	1	1	1
0,6358382	1	1	1	1	1
0,5505618	1	0	0	1	1
0,5664740	1	0	1	1	1
0,6035503	0	0	0	0	0
0,6211180	1	0	1	1	1
0,5956284	1	0	1	1	1
0,6071429	0	0	1	1	1
0,6130952	0	0	1	1	1
0,7192982	1	1	0	1	1
0,5611111	1	0	0	1	1
0,5762712	0	0	1	1	1
0,6011561	1	0	0	1	1
0,6463415	0	0	1	1	1
0,5797101	1	0	1	1	1
0,5573770	1	0	1	1	1
0,5804598	1	1	1	1	1
0,6000000	0	0	1	1	1
0,6058824	1	0	1	1	1
0,6217009	1	1	1	1	1
0,6107784	0	0	0	1	1
0,6117647	1	0	1	1	1
0,6297376	0	0	1	1	1
0,5913978	1	0	0	1	1
0,6867470	0	0	0	1	1
0,5945946	0	0	0	1	1
0,6125000	1	0	0	1	1
0,6289308	0	0	1	1	1
0,5895954	0	0	0	1	1
0,6035503	1	1	1	1	1
0,6184971	0	0	0	1	1
0,6235294	0	0	1	1	1
0,5621302	0	0	1	1	1
0,6130952	1	0	1	1	1
0,6121212	0	0	1	1	1
0,6264368	0	0	1	1	1
0,5938375	1	0	1	1	1
0,6707317	1	1	1	1	1
0,6589595	0	0	0	1	1
0,6111111	0	0	0	1	1
0,6068111	1	1	0	1	1
0,5906433	1	1	1	1	1
0,6167665	1	0	0	1	1
0,5964912	0	0	1	0	1
0,5580110	0	0	1	0	1

0,6353591	0	0	1	1	1
0,5988701	0	0	1	1	1
0,6034483	0	0	0	1	1
0,6094675	0	0	0	0	0
0,6327684	0	0	1	1	1
0,6123596	0	0	1	1	1
0,6022727	1	0	1	1	1
0,6369048	1	1	0	1	1
0,5614035	1	0	0	1	1
0,5945946	1	0	0	1	1
0,5534247	0	0	1	1	1
0,5808383	0	0	0	0	0
0,5659341	1	0	0	1	1
0,5964912	1	0	1	0	1
0,5988372	1	1	1	1	1
0,6000000	0	0	0	1	1
0,6117647	0	0	0	1	1
0,6506024	0	0	1	1	1
0,5872093	0	0	0	1	1
0,6125000	0	0	1	1	1
0,5944444	1	1	1	1	1
0,6117647	0	0	0	1	1
0,6204819	0	0	1	1	1
0,6390533	1	0	1	1	1
0,5623188	0	0	0	1	1
0,5571031	0	0	1	1	1
0,5852273	0	0	1	0	1
0,6011561	0	0	1	0	1
0,6388889	1	0	0	1	1
0,6198830	1	0	1	1	1
0,5454545	0	0	0	1	1
0,5739645	0	0	1	1	1
0,5963855	0	0	0	1	1
0,6079545	1	1	1	1	1
0,6411765	1	1	1	1	1
0,6250000	0	0	1	1	1
0,6201117	1	1	1	1	1
0,6507463	0	0	1	1	1
0,5260274	0	0	1	1	1
0,5584046	1	0	0	1	1
0,6549708	1	1	1	1	1
0,6057143	0	0	0	1	1
0,6196319	0	0	0	1	1
0,6118980	0	0	1	1	1
0,6176471	0	0	0	1	1
0,7037037	0	0	0	1	1
0,5375723	0	0	0	1	1
0,5439560	0	0	1	1	1
0,6077348	1	0	1	1	1
0,5824176	1	0	1	1	1

0,6219512	1	0	1	1	1
0,6000000	0	0	0	1	1
0,6123596	1	1	1	0	1
0,6031746	0	0	0	1	1
0,6098901	1	0	0	1	1
0,6685714	1	0	0	1	1
0,5786517	1	0	0	1	1
0,5617978	0	0	1	1	1
0,6057143	0	0	0	1	1
0,5748503	0	0	0	1	1
0,6011905	1	1	0	1	1
0,5952381	1	0	0	1	1
0,5982405	0	0	1	1	1
0,5938375	0	0	0	0	0
0,6057143	1	1	1	1	1
0,6011561	0	0	0	1	1
0,6213018	1	0	0	0	1
0,6063830	0	0	0	1	1
0,5952381	1	1	1	1	1
0,5675676	0	0	0	1	1
0,6107784	1	1	0	1	1
0,6000000	0	0	0	1	1
0,5798817	0	0	1	1	1
0,5847953	1	0	1	1	1
0,5819209	0	0	0	1	1
0,5795455	1	1	0	1	1
0,6101695	1	0	1	1	1
0,6803519	1	1	0	1	1
0,6264368	1	0	0	1	1
0,6627907	1	0	0	1	1
0,6529412	1	0	0	0	1
0,6058824	0	0	0	1	1
0,6227545	0	0	0	1	1
0,6077348	1	1	0	1	1
0,5892857	1	0	1	1	1
0,5988372	0	0	1	0	1
0,5666667	0	0	0	1	1
0,6167665	0	0	0	1	1
0,6046512	0	0	1	1	1
0,5852273	0	0	0	1	1
0,6964286	0	0	0	1	1
0,6162162	0	0	1	1	1
0,5274725	0	0	0	0	0
0,5319149	1	0	1	1	1
0,5838150	0	0	1	1	1
0,6034483	1	1	1	1	1
0,6069712	1	0	0	1	1
0,6390533	1	1	0	1	1
0,5955056	0	0	0	1	1
0,5681818	0	0	1	1	1

0,5906433	0	0	1	1	1
0,5823529	0	0	0	1	1
0,5795455	0	0	1	1	1
0,6610644	1	0	1	1	1
0,5989011	0	0	0	1	1
0,6136364	1	0	0	1	1
0,6477273	0	0	1	1	1
0,6032609	0	0	0	1	1
0,5798817	0	0	1	1	1
0,6104651	0	0	0	1	1
0,5930233	1	0	1	1	1
0,5909091	0	0	1	1	1
0,6046512	1	0	1	1	1
0,6057143	0	0	0	1	1
0,5389222	0	0	0	1	1
0,5647059	1	1	1	1	1
0,5730337	1	0	1	1	1
0,5604396	0	0	1	1	1
0,5585106	0	0	1	1	1
0,5769231	0	0	0	1	1
0,6611570	0	0	1	1	1
0,6444444	1	1	1	0	1
0,6023392	1	0	0	1	1
0,5927978	0	0	0	0	0
0,6034483	1	1	1	1	1
0,5898876	0	0	1	0	1
0,6047904	1	1	0	1	1
0,6089552	1	1	1	1	1
0,6067416	0	0	1	0	1
0,6402439	1	0	0	1	1
0,6075269	0	0	0	1	1
0,6818182	1	1	1	1	1
0,6540541	1	1	1	1	1
0,5739645	0	0	0	1	1
0,6153846	1	0	1	1	1
0,5844504	0	0	0	1	1
0,6232295	1	0	1	1	1
0,6094675	1	0	1	1	1
0,6312684	0	0	1	1	1
0,6130952	0	0	0	0	0
0,6526946	0	0	0	1	1
0,6285714	0	0	0	1	1
0,6235294	1	0	1	1	1
0,6315789	1	0	0	1	1
0,5714286	1	0	1	1	1
0,5828571	0	0	1	1	1
0,6000000	1	1	1	1	1
0,6227545	1	1	0	1	1
0,5988701	1	0	1	1	1
0,5919540	0	0	1	1	1

0,6292835	0	0	1	1	1
0,5853659	1	0	0	0	1
0,5988024	0	0	1	0	1
0,6206897	1	1	0	1	1
0,6114286	0	0	0	1	1
0,6467066	1	0	0	1	1
0,6547619	1	0	0	1	1
0,6589595	1	1	1	1	1
0,5994065	1	0	1	1	1
0,5977011	0	0	1	1	1
0,6196319	1	1	1	1	1
0,6256983	1	0	1	1	1
0,6153846	0	0	1	1	1
0,6069364	0	0	1	1	1
0,6321839	1	0	0	0	1
0,6298343	1	0	1	1	1
0,5639535	1	0	1	1	1
0,5976331	0	0	0	1	1
0,6158192	1	1	1	1	1
0,6470588	0	0	1	1	1
0,5919540	0	0	0	1	1
0,5911602	0	0	1	0	1
0,6220930	1	0	0	1	1
0,6167665	1	1	1	1	1
0,6213018	1	1	1	1	1
0,6294118	1	1	1	1	1
0,6077348	0	0	0	1	1
0,6268657	1	1	0	1	1
0,6428571	1	0	0	1	1
0,6428571	1	0	1	1	1
0,6510264	0	0	0	0	0
0,6627907	1	1	1	1	1
0,5875706	1	1	1	1	1
0,5769231	0	0	1	1	1
0,5810056	0	0	0	1	1
0,6081871	1	1	0	0	1
0,6547619	1	1	1	1	1
0,5804598	1	0	1	1	1
0,5830904	0	0	1	1	1
0,5868263	1	0	0	0	1
0,6878613	0	0	1	1	1
0,6107784	0	0	1	0	1
0,6470588	0	0	1	1	1
0,6402439	0	0	0	1	1
0,5197740	0	0	0	1	1
0,5668449	0	0	1	1	1
0,5513196	0	0	1	1	1
0,5491329	0	0	0	1	1
0,5878788	1	0	1	1	1
0,6395349	0	0	1	1	1

0,6079545	0	0	1	1	1
0,6132597	1	0	0	1	1
0,5937500	0	0	1	1	1
0,6193182	1	1	1	1	1
0,7142857	0	0	0	1	1
0,6842105	1	1	0	1	1
0,6257669	1	0	0	1	1
0,5872093	1	1	1	1	1
0,5714286	1	1	1	1	1
0,6171429	0	0	1	0	1
0,5882353	1	0	0	1	1
0,6171429	0	0	0	1	1
0,5977011	1	1	0	1	1
0,5801105	0	0	0	1	1
0,6666667	1	0	1	0	1
0,5921788	1	1	1	1	1
0,6198830	1	0	1	1	1
0,6184971	1	0	1	1	1
0,6566265	1	0	1	1	1
0,6569767	0	0	0	1	1
0,6514286	1	0	0	0	1
0,5647059	0	0	0	1	1
0,6358382	0	0	1	1	1
0,5842697	1	1	1	1	1
0,6086957	1	0	1	0	1
0,5963855	0	0	1	1	1
0,5919540	0	0	1	1	1
0,6172840	0	0	1	1	1
0,6114286	0	0	0	1	1
0,6321839	1	0	1	1	1
0,6306818	1	0	1	1	1
0,6079545	0	0	0	1	1
0,6190476	0	0	1	1	1
0,6627907	0	0	1	1	1
0,6032609	1	1	1	1	1
0,6378378	1	1	1	1	1
0,5828571	0	0	1	0	1
0,6424242	1	0	0	1	1
0,5988372	0	0	0	1	1
0,6703911	0	0	1	1	1
0,6069364	0	0	1	1	1
0,5864198	1	1	0	1	1
0,6172840	1	0	1	1	1
0,6071429	1	1	1	1	1
0,5745856	1	1	0	1	1
0,5777778	0	0	1	1	1
0,5913043	1	0	1	1	1
0,6079545	0	0	1	1	1
0,6484848	0	0	1	1	1
0,6224784	0	0	0	1	1

0,6574586	1	0	0	1	1
0,6161616	0	0	0	1	1
0,5269122	0	0	0	1	1
0,5611111	0	0	0	1	1
0,5953757	0	0	0	1	1
0,6045198	1	1	1	1	1
0,5902579	1	0	1	1	1
0,6271186	1	1	1	1	1
0,6000000	0	0	1	1	1
0,6079545	1	1	0	1	1
0,6153846	1	1	1	1	1
0,6337209	1	1	1	1	1
0,7048193	1	1	1	1	1
0,6023392	1	1	0	1	1
0,6257669	0	0	1	0	1
0,6000000	0	0	0	1	1
0,6116208	1	1	1	0	1
0,6176471	1	0	1	1	1
0,6379310	1	1	1	1	1
0,6646707	1	1	1	1	1
0,6511628	0	0	0	1	1
0,6592179	1	0	1	1	1
0,5542857	1	0	1	1	1
0,5930233	1	0	0	1	1
0,6111111	1	1	1	1	1
0,5989011	1	1	1	0	1
0,7218935	1	1	1	1	1
0,6287425	0	0	0	0	0
0,6706949	1	1	1	1	1
0,5763689	0	0	1	1	1
0,5934718	1	0	0	0	1
0,6686747	0	0	0	1	1
0,6181818	1	0	0	1	1
0,5863014	1	0	1	1	1
0,6055556	1	0	1	1	1
0,6058824	1	0	0	1	1
0,6312500	1	0	0	0	1
0,5932203	0	0	1	1	1
0,6111111	1	1	0	1	1
0,6585366	1	1	0	1	1
0,6686391	1	0	1	1	1
0,6704545	0	0	1	1	1
0,6109510	1	1	1	1	1
0,5798817	1	1	0	1	1
0,5847953	0	0	0	1	1
0,6285714	1	0	1	1	1
0,6279070	1	0	1	1	1
0,5882353	1	0	0	1	1
0,5519126	1	0	0	0	1
0,5868263	0	0	1	1	1

0,5795455	0	0	0	0	0
0,5801105	1	0	0	1	1
0,6524390	0	0	0	0	0
0,7142857	1	1	1	1	1
0,6112760	1	0	1	1	1
0,6820809	0	0	0	1	1
0,5815217	0	0	1	1	1
0,6190476	0	0	1	1	1
0,6385542	0	0	0	1	1
0,5511364	1	0	1	0	1
0,5617978	1	1	1	1	1
0,5617978	0	0	1	1	1
0,5786517	0	0	1	1	1
0,5971014	0	0	1	1	1
0,5909091	1	0	1	1	1
0,6189112	1	1	0	1	1
0,5928144	1	0	1	1	1
0,5847953	0	0	0	1	1
0,5681818	1	0	0	1	1
0,6035503	0	0	1	1	1
0,6158192	1	0	1	1	1
0,6022727	1	0	1	1	1
0,6312849	1	0	1	1	1
0,6321839	0	0	1	1	1
0,6312500	1	0	0	1	1
0,5824176	0	0	0	1	1
0,6184971	1	0	0	1	1
0,6264368	0	0	1	1	1
0,6292135	1	1	1	1	1
0,6494253	0	0	1	1	1
0,7058824	0	0	0	1	1
0,6457143	1	1	1	1	1
0,6285714	1	0	1	1	1
0,5675676	1	1	1	1	1
0,6369048	1	0	0	1	1
0,6117647	1	1	1	1	1
0,6453488	0	0	0	1	1
0,6027397	0	0	1	1	1
0,5771429	1	0	0	1	1
0,5833333	1	0	0	0	1
0,5901639	1	0	0	0	1
0,6136364	1	0	0	1	1
0,6474359	1	0	1	1	1
0,6748466	0	0	1	1	1
0,6705202	1	1	0	1	1
0,6964286	1	1	0	1	1
0,5738636	1	0	1	0	1
0,6890244	0	0	1	1	1
0,6227545	1	1	1	1	1
0,5921788	1	0	1	1	1

0,6171429	1	1	1	0	1
0,6189112	1	1	0	1	1
0,6111111	1	0	1	1	1
0,6123596	1	0	0	0	1
0,6149425	1	0	0	1	1
0,6091954	1	1	0	1	1
0,6294118	1	1	1	1	1
0,6951220	1	1	1	1	1
0,6369048	0	0	1	1	1
0,6239554	1	1	0	1	1
0,5696970	1	0	1	1	1
0,6250000	0	0	1	1	1
0,6441718	1	0	1	1	1
0,6099707	1	0	1	1	1
0,6287425	0	0	0	1	1
0,7068966	1	1	1	1	1
0,6108108	1	0	1	1	1
0,6242775	0	0	1	1	1
0,6437500	1	0	1	1	1
0,6432432	1	0	0	1	1
0,6136364	0	0	1	0	1
0,6511628	1	0	0	1	1
0,6369048	0	0	1	0	1
0,6800000	1	0	1	1	1
0,5600000	0	0	1	1	1
0,6069364	0	0	1	0	1
0,6728395	0	0	0	1	1
0,6117647	1	0	1	1	1
0,5964912	1	0	1	1	1
0,6325301	1	0	1	1	1
0,6488095	1	1	0	1	1
0,6402266	1	0	1	1	1
0,6306818	1	0	1	1	1
0,6127168	1	1	0	1	1
0,6077348	1	0	1	1	1
0,6648352	0	0	1	1	1
0,6171429	0	0	0	1	1
0,6534091	1	0	0	1	1
0,6415094	0	0	1	0	1
0,6867470	1	0	0	1	1
0,6590909	0	0	1	1	1
0,5696594	1	0	1	1	1
0,6287425	0	0	0	1	1
0,5895954	1	0	1	1	1
0,6047904	1	1	1	1	1
0,5738636	0	0	1	1	1
0,6117647	0	0	0	0	0
0,6117647	0	0	0	1	1
0,6927711	1	0	1	1	1
0,6420455	1	0	1	1	1

0,5967742	1	0	0	1	1
0,6470588	1	0	1	1	1
0,6436782	0	0	0	1	1
0,6242775	1	1	1	1	1
0,6279070	0	0	0	1	1
0,6666667	1	1	1	1	1
0,5485714	0	0	0	1	1
0,6179775	1	1	0	1	1
0,6497175	1	1	1	1	1
0,6277778	1	0	0	1	1
0,5810056	0	0	0	1	1
0,6309524	1	1	1	1	1
0,6204819	0	0	1	1	1
0,6347305	0	0	0	1	1
0,6378378	0	0	1	1	1
0,6491228	0	0	0	1	1
0,5802817	0	0	0	1	1
0,5714286	0	0	1	1	1
0,6363636	0	0	0	1	1
0,6369048	0	0	1	1	1
0,6312849	0	0	1	1	1
0,5888889	0	0	0	1	1
0,6081871	1	1	1	1	1
0,6424242	1	0	0	1	1
0,6081871	1	0	0	1	1
0,6242775	0	0	1	1	1
0,6432749	1	0	0	0	1
0,6741573	1	0	1	1	1
0,5722543	0	0	1	1	1
0,5952381	1	1	0	1	1
0,6432749	1	1	1	1	1
0,6162791	1	1	1	1	1
0,6491228	1	1	0	1	1
0,6463415	1	1	1	1	1
0,6516854	1	1	0	1	1
0,6086957	1	1	0	1	1
0,6506024	0	0	1	1	1
0,6484848	1	0	1	1	1
0,6723164	0	0	1	1	1
0,5786517	1	0	1	0	1
0,5882353	0	0	0	1	1
0,5852273	0	0	0	0	0
0,5846995	1	1	0	1	1
0,6227545	0	0	0	1	1
0,6586826	0	0	0	1	1
0,5965909	0	0	0	1	1
0,6529412	0	0	1	0	1
0,6235955	1	0	1	1	1
0,6213018	0	0	0	1	1
0,6488095	1	1	1	1	1

0,6725146	0	0	1	1	1
0,6460674	0	0	0	0	0
0,5642458	1	0	1	1	1
0,6214689	0	0	0	1	1
0,6526946	0	0	0	0	0
0,5917160	1	1	0	1	1
0,6000000	1	0	1	1	1
0,5706522	1	1	1	1	1
0,6162791	1	1	0	1	1
0,6467066	1	1	1	1	1
0,6802326	0	0	1	1	1
0,6857143	0	0	1	1	1
0,5828571	0	0	1	1	1
0,6214689	0	0	0	1	1
0,5953757	1	1	1	1	1
0,6066066	1	0	1	1	1
0,6545455	1	1	0	0	1
0,6369048	1	0	1	1	1
0,6407186	1	0	0	1	1
0,6649215	1	0	1	1	1
0,6106443	0	0	1	1	1
0,6167665	0	0	0	1	1
0,6139241	0	0	0	1	1
0,5953757	0	0	1	1	1
0,6463415	0	0	0	1	1
0,6217009	1	0	0	1	1
0,6117647	0	0	1	1	1
0,5977011	1	0	0	1	1
0,6282421	1	1	0	1	1
0,6153846	1	1	1	1	1
0,6473988	1	1	0	1	1
0,6321839	1	0	1	1	1
0,6824926	0	0	0	1	1
0,6352941	1	0	0	1	1
0,5842697	1	1	1	1	1
0,5895954	1	0	1	1	1
0,5895317	0	0	1	1	1
0,7160494	1	0	1	1	1
0,6590258	0	0	1	1	1
0,6011905	0	0	0	1	1
0,6264368	1	1	1	1	1
0,6381766	0	0	0	1	1
0,6079545	1	1	1	1	1
0,6586103	0	0	0	1	1
0,6363636	0	0	0	0	0
0,6243094	1	0	1	1	1
0,6583851	0	0	1	1	1
0,6848485	1	1	1	1	1
0,6627219	0	0	0	1	1
0,6158537	1	1	1	1	1

0,6000000	0	0	0	1	1
0,5953757	1	1	1	1	1
0,6686747	1	1	1	1	1
0,6069364	1	1	1	1	1
0,6235294	0	0	1	0	1
0,6341463	1	1	0	1	1
0,6189112	0	0	1	1	1
0,7126437	1	0	1	1	1
0,6563467	1	0	0	1	1
0,6243094	1	0	1	1	1
0,5906433	0	0	1	0	1
0,5895954	0	0	1	1	1
0,6331361	1	1	1	1	1
0,6449704	1	1	1	1	1
0,6439628	0	0	1	1	1
0,7176471	1	0	0	1	1
0,5913978	1	0	1	1	1
0,6190476	1	0	1	1	1
0,6229508	1	1	1	1	1
0,6062323	0	0	0	0	0
0,6294118	1	0	1	1	1
0,6647399	1	0	1	1	1
0,5722222	1	1	0	1	1
0,5706215	0	0	1	1	1
0,5791045	1	1	1	1	1
0,6058824	0	0	1	1	1
0,6091954	1	0	0	1	1
0,6528190	1	0	0	1	1
0,6581470	0	0	0	1	1
0,6279070	0	0	1	1	1
0,6071429	1	0	0	1	1
0,6171429	1	0	1	1	1
0,6764706	1	0	1	1	1
0,6222222	1	0	1	1	1
0,6369048	1	0	0	1	1
0,6432749	1	0	1	1	1
0,6534091	0	0	1	1	1
0,6473684	1	0	1	1	1
0,6000000	0	0	0	0	0
0,6424242	0	0	0	0	0
0,5977011	1	0	1	1	1
0,6067416	1	1	1	1	1
0,6055556	1	0	1	1	1
0,7028571	0	0	0	1	1
0,6666667	0	0	1	1	1
0,7438017	1	1	1	1	1
0,5795455	0	0	1	1	1
0,5706215	1	0	1	1	1
0,6629834	1	0	0	1	1
0,5816024	0	0	1	1	1

0,5714286	0	0	0	1	1
0,6379310	1	0	1	1	1
0,6744186	1	1	1	1	1
0,6744186	1	0	1	1	1
0,6294118	0	0	0	1	1
0,6628571	0	0	0	1	1
0,6075269	1	1	1	1	1
0,5682451	0	0	1	1	1
0,5930233	1	0	1	1	1
0,6074499	1	0	0	1	1
0,5801105	1	0	1	1	1
0,6197183	0	0	0	1	1
0,6167147	0	0	0	1	1
0,6065574	1	0	1	1	1
0,6202899	0	0	0	1	1
0,6432749	0	0	1	1	1
0,6584615	1	0	1	1	1
0,5876011	0	0	1	1	1
0,6206897	0	0	0	1	1
0,6441718	1	0	1	1	1
0,6769231	1	0	1	1	1
0,6127168	0	0	1	1	1
0,6890244	0	0	0	1	1
0,6516854	1	0	0	1	1
0,6687500	0	0	1	1	1
0,6035503	1	0	1	1	1
0,6094675	0	0	1	1	1
0,6162791	1	1	0	1	1
0,5941176	1	0	0	1	1
0,6363636	1	0	0	1	1
0,6228571	0	0	0	1	1
0,6117647	0	0	1	1	1
0,6193182	0	0	1	1	1
0,6331361	1	0	1	1	1
0,6374269	1	0	1	1	1
0,6612022	1	0	0	1	1
0,5689150	1	1	1	1	1
0,6179775	1	0	1	1	1
0,6395349	1	0	1	1	1
0,5921788	1	0	0	1	1
0,6257310	0	0	1	0	1
0,6645963	1	0	1	1	1
0,6130952	0	0	1	1	1
0,6508876	1	0	1	1	1
0,6792453	1	0	1	1	1
0,6705882	1	1	1	1	1
0,5491329	0	0	1	1	1
0,6341463	0	0	0	1	1
0,5909091	1	1	1	1	1
0,6257310	0	0	1	0	1

0,6728395	0	0	1	1	1
0,6829268	1	0	0	1	1
0,6271186	0	0	1	1	1
0,6800000	0	0	1	1	1
0,6875000	1	0	1	1	1
0,6334311	0	0	1	1	1
0,6214689	0	0	0	1	1
0,6184971	1	0	0	0	1
0,6331361	0	0	1	1	1
0,6111111	0	0	1	1	1
0,6920821	1	0	1	1	1
0,6555556	1	0	1	1	1
0,5780347	1	0	0	1	1
0,6758242	0	0	0	1	1
0,6250000	0	0	0	1	1
0,6819484	1	0	1	1	1
0,6190476	1	0	1	1	1
0,6445783	0	0	0	1	1
0,6606061	0	0	1	1	1
0,6046512	0	0	1	1	1
0,6179775	0	0	0	1	1
0,5857988	0	0	1	1	1
0,6250000	0	0	0	1	1
0,6549708	1	0	1	1	1
0,6094183	0	0	0	1	1
0,6477987	0	0	0	1	1
0,6385542	0	0	0	1	1
0,6380368	0	0	0	1	1
0,6375000	0	0	1	1	1
0,6686047	1	0	1	1	1
0,6485014	0	0	0	1	1
0,5964912	1	0	1	1	1
0,6592179	1	0	1	1	1
0,6666667	0	0	1	1	1
0,6187845	1	0	0	1	1
0,6181818	0	0	1	1	1
0,6705202	0	0	1	1	1
0,6222222	1	0	1	1	1
0,6477273	1	0	1	1	1
0,6280488	0	0	1	1	1
0,6514286	1	1	1	0	1
0,6500000	1	0	0	1	1
0,6726190	1	1	1	1	1
0,5952381	0	0	0	1	1
0,5819209	1	0	0	0	1
0,6298343	0	0	1	1	1
0,5919540	0	0	0	1	1
0,6545455	1	1	1	1	1
0,6242038	1	0	0	1	1
0,6279070	1	1	1	1	1

0,6626506	1	1	1	1	1
0,6529412	1	0	1	1	1
0,5909091	0	0	1	0	1
0,6626506	0	0	1	1	1
0,6607143	1	1	1	1	1
0,6035503	0	0	1	0	1
0,6104651	0	0	1	1	1
0,6264368	1	0	0	1	1
0,6488095	1	0	0	1	1
0,6941176	0	0	1	1	1
0,6988636	1	1	0	0	1
0,6453488	0	0	0	1	1
0,6666667	1	1	0	1	1
0,5771429	1	1	0	1	1
0,6294118	0	0	0	1	1
0,6845238	1	0	1	1	1
0,6547619	1	0	0	1	1
0,6875000	0	0	1	1	1
0,6234568	1	1	0	1	1
0,7093023	1	0	1	1	1
0,6242775	0	0	1	1	1
0,6256983	0	0	1	0	1
0,6300578	0	0	0	1	1
0,6131805	1	1	1	1	1
0,6514286	0	0	0	1	1
0,6648352	1	0	0	1	1
0,6705882	0	0	1	1	1
0,6590909	0	0	1	1	1
0,6280488	0	0	1	0	1
0,6171429	0	0	1	1	1
0,6878307	1	1	0	1	1
0,6790123	1	0	1	1	1
0,6567164	0	0	1	1	1
0,6777778	1	0	0	1	1
0,6721311	1	0	0	1	1
0,6250000	1	1	1	1	1
0,6488095	0	0	0	1	1
0,6890244	0	0	1	1	1
0,6440678	1	0	1	1	1
0,6812500	0	0	1	1	1
0,6167665	1	0	0	1	1
0,6400000	0	0	1	0	1
0,6174863	1	0	0	1	1
0,6436782	0	0	0	0	0
0,6467066	1	1	0	1	1
0,6058824	1	0	1	0	1
0,6202899	1	0	0	0	1
0,6969697	1	0	0	1	1
0,6395349	1	1	1	1	1
0,7005988	0	0	0	1	1

0,5642458	0	0	0	1	1
0,5842697	0	0	0	1	1
0,6229508	0	0	0	1	1
0,5961003	1	0	1	1	1
0,6520376	1	1	1	1	1
0,6706587	1	0	1	0	1
0,6149425	1	0	1	1	1
0,6331361	0	0	1	0	1
0,5744681	0	0	0	1	1
0,6623681	0	0	1	1	1
0,6467066	0	0	0	1	1
0,6628571	0	0	0	1	1
0,6214689	1	0	0	0	1
0,6611111	0	0	1	1	1
0,6366197	0	0	1	1	1
0,6863905	1	0	0	1	1
0,6923077	1	0	1	1	1
0,6748466	0	0	0	1	1
0,5771429	0	0	1	1	1
0,6686930	0	0	1	1	1
0,6611111	0	0	1	1	1
0,7073171	1	1	1	1	1
0,6107784	1	1	1	1	1
0,6315789	1	1	1	1	1
0,6285714	0	0	1	1	1
0,6666667	0	0	1	1	1
0,6397695	1	1	0	1	1
0,6432749	1	0	0	1	1
0,6723164	1	1	1	1	1
0,6590909	1	1	0	1	1
0,5975610	1	1	1	1	1
0,6242775	0	0	0	1	1
0,5953757	0	0	0	1	1
0,6227545	1	0	1	1	1
0,6104651	0	0	1	1	1
0,6303030	1	0	0	1	1
0,6101695	1	0	1	1	1
0,6219512	1	0	1	1	1
0,6607143	1	0	1	1	1
0,6626506	1	1	1	1	1
0,6235955	0	0	0	1	1
0,7500000	1	1	1	1	1
0,6745562	1	0	0	1	1
0,6686747	1	1	1	1	1
0,7294118	0	0	0	1	1
0,5947368	0	0	0	1	1
0,6555556	1	0	1	1	1
0,6646707	1	0	0	1	1
0,6871508	0	0	1	0	1
0,6132597	1	1	1	1	1

0,6213018	0	0	1	0	1
0,6956522	0	0	1	1	1
0,6256983	0	0	1	1	1
0,6802326	0	0	1	1	1
0,6568915	1	1	1	1	1
0,7005988	1	0	1	1	1
0,6071429	1	1	0	1	1
0,5915493	0	0	1	1	1
0,6534091	0	0	0	1	1
0,6568047	1	1	1	1	1
0,6034483	1	0	1	1	1
0,6529412	1	1	0	1	1
0,6449704	0	0	1	1	1
0,6000000	1	0	1	1	1
0,6081871	1	1	0	0	1
0,6315789	1	0	1	1	1
0,6407186	0	0	1	1	1
0,6832298	0	0	1	1	1
0,6569767	0	0	0	1	1
0,6285714	0	0	1	1	1
0,6395349	1	0	0	1	1
0,7643678	1	1	1	1	1
0,5722222	0	0	1	1	1
0,6275660	1	1	1	1	1
0,6705882	0	0	0	1	1
0,6807229	0	0	1	1	1
0,6206897	1	0	1	1	1
0,6250000	0	0	0	1	1
0,6379310	0	0	0	1	1
0,6046512	1	0	1	1	1
0,6845238	0	0	0	1	1
0,6243094	0	0	1	1	1
0,7215909	0	0	1	1	1
0,6053412	1	0	1	1	1
0,6725146	1	0	1	1	1
0,6268657	1	0	0	1	1
0,6016260	0	0	0	1	1
0,6804734	0	0	1	1	1
0,6358382	0	0	1	1	1
0,6855346	1	1	1	1	1
0,6946108	1	1	1	1	1
0,7558140	1	1	1	1	1
0,6214689	0	0	1	1	1
0,6222222	1	1	0	1	1
0,6128134	0	0	1	1	1
0,6860465	0	0	0	1	1
0,6629213	1	1	1	1	1
0,6407186	1	1	1	1	1
0,7093023	1	1	0	1	1
0,6588921	0	0	1	1	1

0,5885714	1	0	1	1	1
0,6422018	1	1	0	1	1
0,6235294	1	1	0	1	1
0,6564417	1	0	1	1	1
0,6511628	0	0	1	1	1
0,6809816	0	0	1	1	1
0,6764706	0	0	1	1	1
0,6842105	1	1	1	1	1
0,6352941	1	1	1	0	1
0,6628895	0	0	1	1	1
0,6759777	0	0	1	1	1
0,7058824	1	1	1	1	1
0,6551724	1	0	1	0	1
0,6782609	1	0	1	1	1
0,6627907	0	0	0	1	1
0,6728395	1	1	0	1	1
0,6432749	1	0	1	1	1
0,6404494	1	1	1	1	1
0,6904762	0	0	0	1	1
0,5865922	1	0	1	1	1
0,6426426	0	0	1	1	1
0,6420455	0	0	0	1	1
0,6516854	1	0	0	1	1
0,7072464	1	0	1	1	1
0,6946108	1	0	1	1	1
0,7005988	0	0	1	1	1
0,6666667	0	0	1	1	1
0,6781609	0	0	0	1	1
0,6473988	1	0	1	1	1
0,6626506	1	0	1	1	1
0,7251462	1	0	1	1	1
0,7022472	1	1	1	1	1
0,6340058	0	0	1	1	1
0,6627219	0	0	1	1	1
0,6685552	1	0	1	1	1
0,7425150	0	0	1	1	1
0,7297297	0	0	1	1	1
0,6096866	1	0	1	1	1
0,6097561	1	1	0	1	1
0,6900585	1	0	1	1	1
0,6592179	1	0	1	1	1
0,6497175	1	0	0	1	1
0,6815642	1	0	1	1	1
0,6807229	0	0	1	1	1
0,6368421	0	0	0	1	1
0,7028571	0	0	1	1	1
0,7016575	0	0	1	1	1
0,7283237	0	0	0	1	1
0,6472303	0	0	0	1	1
0,6467391	1	0	1	1	1

0,6910112	0	0	0	1	1
0,6512968	1	0	1	1	1
0,6337209	1	1	1	1	1
0,5988372	0	0	0	1	1
0,6770186	1	1	0	1	1
0,5976331	0	0	1	1	1
0,5828571	1	0	1	1	1
0,6043956	1	1	1	1	1
0,7356322	1	1	0	1	1
0,6449704	1	1	1	1	1
0,7415730	0	0	1	1	1
0,6829268	1	1	1	1	1
0,6309524	1	0	1	1	1
0,6229508	1	0	1	1	1
0,6149425	1	0	1	1	1
0,6508876	1	0	0	1	1
0,6839080	0	0	0	1	1
0,7556818	1	1	1	1	1
0,7378049	0	0	1	1	1
0,5459770	1	1	1	1	1
0,6882353	0	0	0	1	1
0,5882353	0	0	0	0	0
0,6267806	1	1	1	1	1
0,5932203	0	0	0	1	1
0,6358382	0	0	1	1	1
0,6184971	0	0	0	1	1
0,7192982	1	0	1	1	1
0,6457143	1	1	1	1	1
0,6627907	1	0	1	1	1
0,6666667	0	0	1	1	1
0,6609195	0	0	0	1	1
0,6420455	0	0	1	1	1
0,7267442	1	1	1	1	1
0,6279070	0	0	0	1	1
0,6428571	1	0	0	1	1
0,6220930	0	0	1	1	1
0,6744186	1	0	1	1	1
0,6536313	1	0	0	1	1
0,6363636	0	0	0	1	1
0,6923077	0	0	1	1	1
0,6702128	1	1	1	1	1
0,6510264	0	0	1	1	1
0,6250000	0	0	1	1	1
0,6358382	0	0	0	1	1
0,6726190	0	0	1	1	1
0,6374269	0	0	0	1	1
0,6071429	1	0	1	1	1
0,6272189	1	1	0	1	1
0,6397695	1	0	1	1	1
0,6184971	1	1	1	1	1

0,6666667	0	0	0	1	1
0,6395349	1	0	1	1	1
0,6067416	1	1	0	1	1
0,6802326	1	0	1	1	1
0,6444444	0	0	1	1	1
0,6555556	0	0	0	1	1
0,6363636	0	0	1	1	1
0,6220930	1	1	0	1	1
0,6927374	1	0	1	1	1
0,6400000	0	0	1	1	1
0,6250000	0	0	0	1	1
0,6823529	1	0	1	1	1
0,6627907	1	1	1	1	1
0,6842105	0	0	0	1	1
0,5964912	0	0	1	1	1
0,6609195	0	0	0	1	1
0,7100592	1	0	0	1	1
0,7028571	1	0	1	1	1
0,6807229	0	0	0	1	1
0,6721311	1	1	1	1	1
0,6318841	0	0	0	0	0
0,5838150	1	0	1	1	1
0,6705882	0	0	0	1	1
0,6424581	0	0	1	0	1
0,6863905	1	1	0	1	1
0,7065868	1	0	1	1	1
0,6171429	0	0	1	1	1
0,6264368	0	0	1	1	1
0,6968839	1	0	1	1	1
0,6607143	1	1	0	1	1
0,7185629	0	0	1	1	1
0,6325301	0	0	1	0	1
0,6341463	0	0	1	1	1
0,7176471	1	0	1	0	1
0,6470588	0	0	1	0	1
0,7134503	1	0	1	1	1
0,6627907	1	0	1	1	1
0,6067416	1	0	1	1	1
0,6166667	1	0	0	0	1
0,5988372	0	0	0	1	1
0,7200000	1	0	1	1	1
0,6420455	1	0	0	1	1
0,6882353	1	0	1	1	1
0,6923077	1	1	1	1	1
0,7230321	0	0	0	1	1
0,6607143	1	1	1	1	1
0,6551724	0	0	1	1	1
0,6272189	1	0	0	1	1
0,6158537	1	1	1	1	1
0,6294118	1	1	0	1	1

0,6666667	0	0	1	1	1
0,6592179	0	0	1	1	1
0,6331361	1	1	1	1	1
0,6111111	1	0	1	1	1
0,6011236	0	0	0	1	1
0,6457143	0	0	0	0	0
0,6145251	0	0	0	1	1
0,6802326	0	0	1	1	1
0,6742857	1	1	0	1	1
0,6516854	1	1	1	1	1
0,6250000	1	0	1	1	1
0,6193182	0	0	0	1	1
0,6842105	1	0	1	0	1
0,6264368	1	0	1	1	1
0,6724138	1	0	1	1	1
0,7126437	0	0	1	1	1
0,6927711	1	0	1	1	1
0,7090909	0	0	0	1	1
0,6071429	1	0	1	1	1
0,6592179	1	0	1	1	1
0,6851852	1	0	0	1	1
0,6457143	0	0	0	1	1
0,7613636	1	1	1	1	1
0,5856354	1	0	1	1	1
0,6573034	1	0	0	1	1
0,6823529	1	1	1	1	1
0,6608187	1	0	0	1	1
0,6521739	0	0	1	1	1
0,7272727	1	1	1	1	1
0,6784661	1	1	1	1	1
0,6140351	0	0	1	1	1
0,7070064	0	0	1	1	1
0,6348315	1	0	1	1	1
0,5714286	0	0	0	1	1
0,5878788	1	0	0	1	1
0,6306818	0	0	1	1	1
0,6626506	0	0	0	1	1
0,6964286	0	0	0	1	1
0,6802326	1	0	1	1	1
0,7125749	1	1	0	1	1
0,6818182	0	0	0	1	1
0,6703911	1	1	1	0	1
0,6352941	0	0	0	1	1
0,7256098	0	0	1	1	1
0,7058824	0	0	1	1	1
0,6266667	0	0	1	1	1
0,6228571	1	1	1	1	1
0,6355685	1	0	1	1	1
0,7252747	1	0	1	1	1
0,6352941	0	0	1	1	1

0,7151163	1	0	0	1	1
0,7102273	1	0	1	1	1
0,5808383	0	0	1	1	1
0,7356322	1	1	1	1	1
0,7380952	1	1	1	1	1
0,6036585	0	0	1	1	1
0,6105919	1	0	1	1	1
0,6845238	1	0	1	1	1
0,6914286	0	0	1	1	1
0,5529412	0	0	0	1	1
0,7151163	1	1	1	1	1
0,6795580	0	0	0	1	1
0,6779661	1	0	1	1	1
0,5611940	1	0	0	1	1
0,7085714	1	0	0	1	1
0,6809816	1	1	1	1	1
0,7288630	1	0	0	0	1
0,6041056	0	0	0	1	1
0,6703911	0	0	1	1	1
0,7578348	1	1	0	1	1
0,6954023	0	0	0	1	1
0,6971429	1	0	0	1	1
0,6742857	0	0	1	1	1
0,5773810	1	0	0	0	1
0,6120219	0	0	1	1	1
0,6823529	0	0	0	1	1
0,7159091	0	0	1	1	1
0,6779661	1	1	1	1	1
0,5730337	1	1	0	1	1
0,6303725	1	1	1	1	1
0,6592179	1	0	0	1	1
0,6477273	1	0	1	1	1
0,6666667	1	0	1	1	1
0,6473988	0	0	1	1	1
0,6988636	1	0	1	1	1
0,6084656	1	1	0	1	1
0,6842105	1	1	1	0	1
0,7202381	0	0	1	1	1
0,5666667	0	0	0	1	1
0,5600000	1	0	0	1	1
0,5930233	0	0	0	0	0
0,6135693	0	0	1	1	1
0,6666667	0	0	0	1	1
0,6195652	1	0	0	1	1
0,6830601	1	0	0	1	1
0,6352941	1	1	1	1	1
0,6395349	0	0	0	1	1
0,6855524	0	0	1	1	1
0,6395349	1	1	1	1	1
0,6611111	0	0	0	1	1

0,6780627	1	1	1	1	1
0,6511628	1	0	1	0	1
0,6117647	1	0	0	1	1
0,6428571	0	0	0	1	1
0,6812500	0	0	1	0	1
0,7257143	0	0	0	1	1
0,5988701	0	0	0	1	1
0,6724138	0	0	1	1	1
0,6294118	1	1	1	1	1
0,7000000	0	0	0	1	1
0,6380368	1	0	1	1	1
0,7185629	1	0	1	1	1
0,5690608	1	0	1	1	1
0,5976331	1	0	0	1	1
0,6993865	0	0	0	1	1
0,7000000	0	0	1	1	1
0,7247191	0	0	0	1	1
0,6767372	1	0	1	1	1
0,6785714	0	0	1	1	1
0,7160494	0	0	0	1	1
0,7356322	1	1	1	1	1
0,6627907	0	0	1	1	1
0,6235294	1	0	1	1	1
0,5795455	1	0	1	1	1
0,6536313	1	1	1	1	1
0,6647399	1	1	1	1	1
0,6347305	1	1	1	0	1
0,6839080	1	0	1	1	1
0,6145251	1	0	0	1	1
0,7125749	1	1	1	1	1
0,5930233	1	1	1	1	1
0,6892655	0	0	1	1	1
0,7654321	1	1	0	1	1
0,6588235	1	1	0	1	1
0,6685393	0	0	0	1	1
0,6235955	0	0	1	1	1
0,5775401	1	0	1	1	1
0,7844311	0	0	1	1	1
0,5722222	1	0	1	1	1
0,6779661	0	0	0	1	1
0,7164179	0	0	1	1	1
0,6114286	0	0	1	1	1
0,7560976	1	0	1	1	1
0,6586826	1	0	1	1	1
0,6894410	1	0	0	1	1
0,7283951	0	0	1	1	1
0,6629213	1	1	1	1	1
0,6666667	1	0	1	1	1
0,6518987	1	0	1	1	1
0,6369048	1	1	1	1	1

0,6722222	1	0	0	1	1
0,6646707	0	0	1	1	1
0,6264368	1	1	1	1	1
0,6459948	1	0	0	1	1
0,6455331	0	0	1	1	1
0,6809816	1	1	1	1	1
0,6411765	0	0	1	1	1
0,7100592	1	1	1	1	1
0,7479675	0	0	1	1	1
0,6589595	1	1	0	1	1
0,7151515	1	1	1	1	1
0,7052023	1	1	1	1	1
0,6795580	0	0	0	1	1
0,5454545	1	1	0	0	1
0,6363636	1	1	0	1	1
0,7575758	1	1	1	1	1
0,7543860	0	0	1	1	1
0,5813953	0	0	0	1	1
0,7234043	0	0	0	0	0
0,7083333	1	1	0	1	1
0,8255814	1	1	1	1	1
0,7784091	1	1	1	1	1
0,7528090	1	0	0	1	1
0,7396450	1	0	1	1	1
0,8588957	1	1	1	1	1
0,6976744	1	0	0	1	1
0,7426901	0	0	1	1	1
0,7251462	1	1	1	1	1
0,7058824	0	0	1	1	1
0,6954023	0	0	1	1	1
0,7251462	0	0	1	1	1
0,7471264	1	1	1	1	1
0,7283237	0	0	1	1	1
0,7428571	0	0	1	1	1
0,7352941	1	1	1	1	1
0,7102273	0	0	0	1	1
0,7000000	1	0	1	1	1
0,7664671	0	0	0	1	1
0,8011869	0	0	1	1	1
0,8208092	1	0	1	1	1
0,7485380	1	1	1	1	1
0,7965116	0	0	1	1	1
0,7966102	0	0	1	1	1
0,7428571	1	0	1	1	1
0,7443182	0	0	1	1	1
0,6884273	1	1	1	1	1
0,7674419	0	0	1	1	1
0,7551622	1	0	1	1	1
0,8301887	1	0	0	1	1
0,8192771	1	1	1	1	1
